# Bi(III)-Catalyzed Enantioselective Allylation Reactions of Ketimines

**DOI:** 10.1016/j.isci.2019.06.006

**Published:** 2019-06-11

**Authors:** Jie Wang, Qingxia Zhang, Biying Zhou, Chen Yang, Xin Li, Jin-Pei Cheng

**Affiliations:** 1 State Key Laboratory of Elemento-Organic Chemistry, College of Chemistry, Nankai University, Tianjin 300071, China

**Keywords:** Catalysis, Organic Synthesis, Organic Reaction

## Abstract

Chiral homoallylic amines not only are found in pharmaceutically relevant compounds but also serve as versatile building blocks for chemical synthesis. However, catalytic allylation of ketimines with allylboronates, an attractive approach to synthesize chiral homoallylic amine scaffolds remain scarce. Herein, we develop a highly enantioselective allylation of isatin-derived ketimines with boron allylation reagents catalyzed by a Bi(OAc)_3_-chiral phosphoric acid catalyst system. The reactions are remarkably efficient and mild, most of which were completed in less than an hour at room temperature with only 1/2 mol% (Bi(OAc)_3_/CPA) catalyst loading. A wide range of chiral 3-allyl 3-aminooxindoles were obtained in excellent yields and enantioselectivities. The synthetic utility was demonstrated by efficient formal synthesis of (+)-AG-041R and (−)-psychotriasine. Preliminary mechanism was studied by control experiments and theoretical calculations.

## Highlights

• Asymmetric allylation of ketimines
• Bi(OAc)_3_-chiral phosphoric acid catalyst
• Downstream synthetic transformations

## Graphical Abstract

**Figure:**
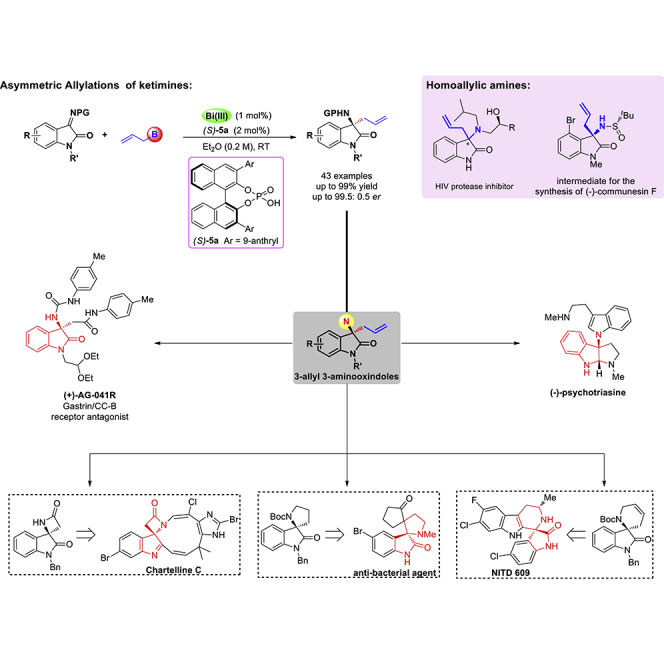

## Introduction

**Scheme 1:**
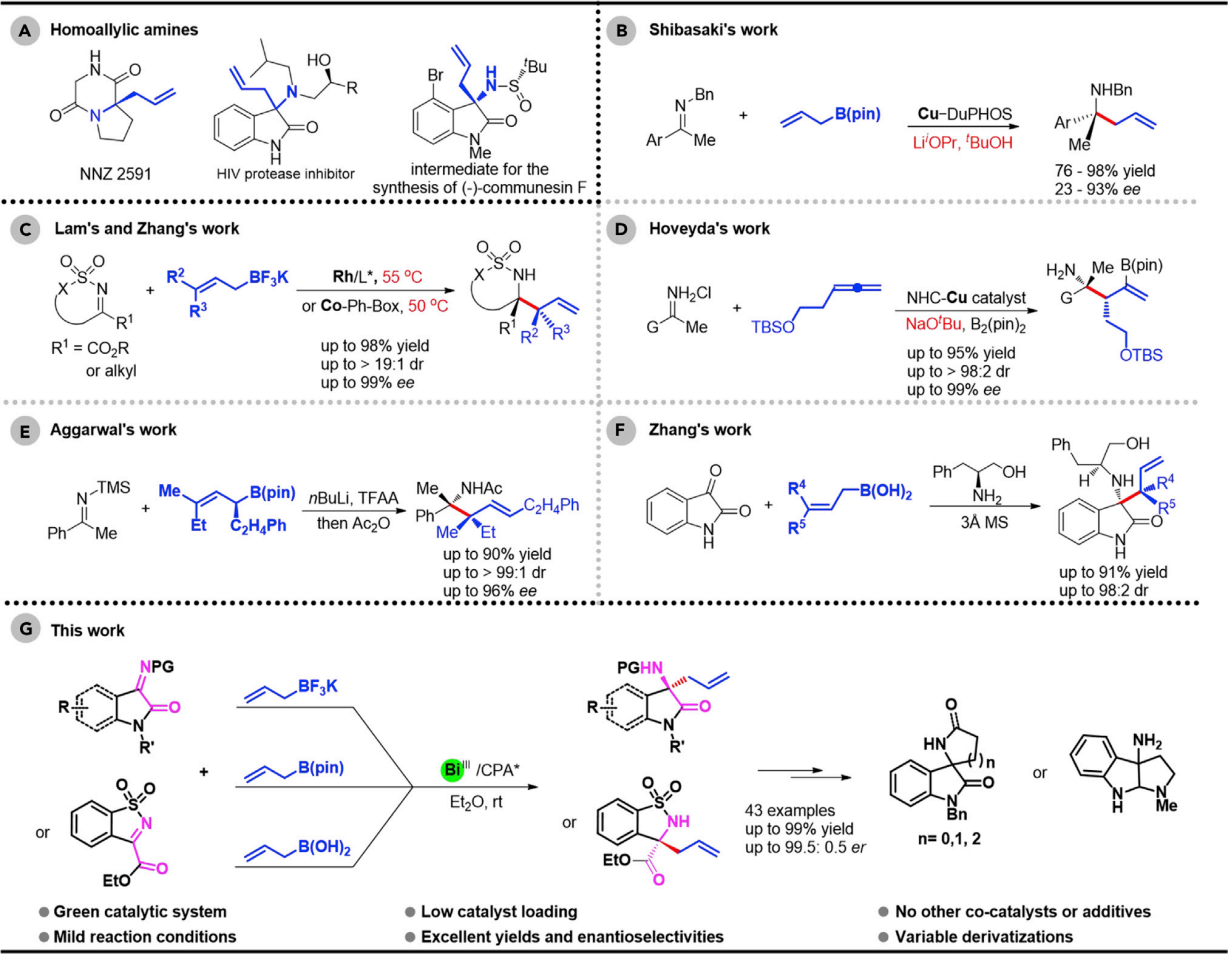
Construction of Chiral Homoallylic Amines through Addition of Allylboronates to Ketimines (A) Examples of biologically active homoallylamines. (B) Cu-catalyzed addition of allylboronates to ketimines. (C) Rh- or Co-catalyzed addition of allylboronates to ketimines. (D) Cu-catalyzed three-component reaction of N–H ketimines. (E) Addition of chiral allylboronates to ketimine. (F) Addition of allylboronates to ketimines controlled by chiral reagent. (G) Bi-catalyzed addition of allylboronates to ketimines.

Chiral homoallylic amines not only are widely found in natural products and pharmaceutically relevant compounds (Guan et al., 2003, Ghosh et al., 2006) but also serve as versatile building blocks for chemical synthesis (Scheme 1A) (Sirasani and Andrade, 2011, Lathrop et al., 2016). Therefore, the asymmetric synthesis of chiral homoallylic amine scaffolds is of great interest in the organic chemistry community (Kumar et al., 2016, Wan et al., 2017). In this context, the asymmetric addition reaction of allylboronates to imines has been recognized as one of the most efficient methods for the construction of chiral homoallylic amines (Kennedy and Hall, 2003, Yus et al., 2011, Huo et al., 2014). Compared with the additions of allylboronates to aldimines (Lou et al., 2007, Lou and Schaus, 2008, Silverio et al., 2013, Wu et al., 2014, Jiang et al., 2017a, Jiang et al., 2017b, Jiang and Schaus, 2017), the corresponding asymmetric allylation of ketimines remains scarce, probably owing to the low reactivity of ketimines. Pioneering enantioselective allylation of acyclic ketimines with allylboronates by using DuPHOS-CuF catalyst has been demonstrated in 2006 by Shibasaki group (Scheme 1B) (Wada et al., 2006). In addition, Rh (and Co)-catalyzed enantioselective additions of potassium allyltrifluoroborates to cyclic N-sulfonyl α-ketiminoesters were also reported (Scheme 1C) (Luo et al., 2012, Hepburn et al., 2013, Hepburn and Lam, 2014, Wu et al., 2018). Very recently, Hoveyda reported NHC-CuCl complex-catalyzed highly stereoselective additions of versatile allyl groups to N-H ketimines (Scheme 1D) (Jang et al., 2017). Other methods involve using enantiomerically pure boron allylation reagent (Scheme 1E) (Chen and Aggarwal, 2014) or chiral inducing amine alcohol reagent (Scheme 1F) (Tan et al., 2017). Despite the mentioned achievements, several limitations, including high catalyst loading, long reaction time, harsh reaction conditions, and limited substrates, remain vast challenges to this field. Furthermore, such endeavors have been relying on either the utilization of canonical transition metal catalysis or stoichiometric chiral reagent. In consequence, the discovery of an efficient catalyst system that could enable the allylation of ketimines by allylboronate reagents in a more efficient and stereoselective fashion would provide access to chiral homoallylic amines in a sustainable manner.

Over the past few decades, chiral Lewis acid catalysis, a significant approach to obtain optically active compounds, had been well developed (Yamamoto, 2000, Yamamoto and Futatsugi, 2005, Yamamoto and Ishihara, 2008, Liu et al., 2011, Liu et al., 2014, Lv and Luo, 2013, Mlynarski, 2017). Although rare-earth metals, the first-row transition metals, and boron-type compounds are the most popular Lewis acid catalysts, the chiral alkaline-earth metal-based catalysts have also attracted ever-growing interest for meeting the needs of green sustainable chemical synthesis (Hatano et al., 2010, Zhang et al., 2011, Zheng et al., 2011, Li et al., 2013). Bismuth compounds, due to their low toxicity and non-corrosiveness, have always been considered as suitable for designing environmentally benign catalysts (Bothwell et al., 2011, Salvador et al., 2012, Ollevier, 2013, Ondet et al., 2017). However, the asymmetric bismuth catalysis remains a relatively unexplored field (Wada et al., 1997, Kobayashi et al., 2005, Kobayashi and Ogawa, 2006, Koch and Peters, 2007, Koch and Peters, 2011, Lassauque et al., 2009, Mahajan et al., 2011, Li et al., 2012, Kitanosono et al., 2013, Isomura et al., 2019). Thus, the development of efficient catalytic transformations using chiral bismuth system is highly meaningful and desirable.

Chiral 3-amino-2-oxindole is an important structural motif in medicinally relevant compounds (Zhou et al., 2010, Singh and Desta, 2012, Cao et al., 2018). Especially, the homoallylic aminooxindole derivatives not only act directly as an inhibitor of HIV-1 protease (Scheme 1A) but also can be converted into aminooxindole frameworks presented in alkaloids (Scheme 3B and 3C). In 2013, Nakamura demonstrated the enantioselective allylation of isatin-derived ketimines catalyzed by Pd-pincer-complexes and AgF under strict reaction conditions (−30°C) (Nakamura et al., 2013). In 2016, Cai group reported an enantioselective In(OTf)_3_-catalyzed allylation of ketimines derived from isatins with highly toxic allyltributyltin; however, this method is not suitable for the substrates with electron-withdrawing groups (Chen and Cai, 2016). Herein, we report a Bi(III)-catalyzed asymmetric allylation of isatin-derived ketimines with allylboronates under rather mild reaction conditions (Scheme 1G). A wide range of chiral 3-allyl 3-aminooxindoles were smoothly obtained in excellent yields with exceptional stereocontrol to forge the quaternary stereogenic carbon centers (Scheme 1G).

## Results and Discussion

### Optimization of the Reaction Conditions

Binaphthols have been proved to be efficient catalysts for the reactions of boronate with ketones (Lou et al., 2006, Barnett et al., 2009, Alam et al., 2015) and aldimines (Lou et al., 2007, Lou and Schaus, 2008, Jiang et al., 2017a, Jiang et al., 2017b, Jang et al., 2017); we initially attempted the reaction of the isatin-derived *N*-Boc-protected ketimine **1a** and allylboronic acid pinacol ester **2a** with binaphthol **4**, yet catalyst **4** could not promote this reaction (Table 1, entry 1). Then we turned our attention to chiral phosphoric acids, which have also been considered as good catalysts to realize the allylboration of aldehydes (Jain and Antilla, 2010). Although chiral phosphoric acid (*S*)-**5a** indeed catalyzed the reaction to give product **3a** with 85.9: 14.1 *er*, only 17% yield was obtained after 48h (Table 1, entry 2). The reactivity is obviously unsatisfactory. We suspected that the Brønsted acidity of chiral phosphoric acid is not strong enough to simultaneously activate ketimine **1a** and allylboronate **2a**. Inspired by Luo's asymmetric binary acid catalysis (Lv et al., 2011, Lv et al., 2013, Hashimoto et al., 2013, Hatano et al., 2015, Wang et al., 2017, Zhang et al., 2017a) and the bismuth catalyzed allylation of *para*-quinone with allylboronate **2a** developed by our group (Zhang et al., 2017b), we proposed that this transformation was likely to be promoted by the BiX_3_-chiral phosphoric acid catalyst system and the use of chiral phosphoric acid could ensure the stereochemistry of this process. Gratifyingly, in the presence of *(S)*-**5a** and Bi(OAc)_3_, the model reaction gave product **3a** in quantitative yield with 87.9: 12.1 *er* (Table 1, entry 3). We then examined other *(S)-BINOL* chiral phosphoric acid with Bi(OAc)_3_, but no better results were achieved (Table 1, entries 4–8). Screening of solvents (Table 1, entries 9–15) revealed that the reaction was favored in Et_2_O (Table 1, entry 15). When catalyst loading was lowered to 1 mol% Bi(OAc)_3_ and 2 mol% *(S)*-**5a**, the yield (96%) and enantioselectivity (99.1: 0.9 *er*) essentially remained the same in comparison with those with high catalyst loading (Table 1, entry 16). The counter anions of Bi(III) and different Lewis acids were also investigated in the model reaction. The use of other bismuth salts resulted in either low reactivities or poor stereoselectivities (Table 1, entries 17–22). Exploring other metal acetates, including Sc(III), In(III), and Y(III), almost all showed poor catalytic activities (Table 1, entries 23–28). Thus, the optimal reaction conditions were finally determined to be 1 mol% Bi(OAc)_3_ and 2 mol% *(S)*-**5a** in Et_2_O (0.2 M) at room temperature (Table 1, entry 16).

**Table 1:** Reaction Optimization


Entry	LA	CPA	Solvent	Time	Yield^a^/%	*er*^b^
1^c^	–	**4**	DCM	64 h	n.r.	–
2^c^	–	**5a**	DCM	48 h	17	85.9:14.1
3	Bi(OAc)_3_	**5a**	CHCl_3_	20 min	99	87.9:12.1
4	Bi(OAc)_3_	**5b**	CHCl_3_	25 min	88	61.7:38.3
5	Bi(OAc)_3_	**5c**	CHCl_3_	40 min	98	75.3:24.7
6	Bi(OAc)_3_	**5d**	CHCl_3_	25 min	95	55.1:44.9
7	Bi(OAc)_3_	**5e**	CHCl_3_	25 min	92	50.4:49.6
8	Bi(OAc)_3_	**6a**	CHCl_3_	80 min	99	17.0:83.0
9	Bi(OAc)_3_	**5a**	DCM	20 min	89	86.0:14.0
10	Bi(OAc)_3_	**5a**	Toluene	15 min	98	97.7:2.3
11	Bi(OAc)_3_	**5a**	EA	15 min	96	98.3:1.7
12	Bi(OAc)_3_	**5a**	CH_3_CN	50 min	99	88.2:11.8
13	Bi(OAc)_3_	**5a**	THF	75 min	93	97.0:3.0
14	Bi(OAc)_3_	**5a**	Dioxane	45 min	99	96.6:3.4
15	Bi(OAc)_3_	**5a**	Et_2_O	20 min	99	99.2:0.8
16^d^	Bi(OAc)_3_	**5a**	Et_2_O	35 min	96	99.1:0.9
17^d^	Bi(OTf)_3_	**5a**	Et_2_O	24 h	30	57.0:43.0
18^d^	BiCl_3_	**5a**	Et_2_O	24 h	27	53.9:46.1
19^d^	BiBr_3_	**5a**	Et_2_O	24 h	81	52.6:47.4
20^d^	BiI_3_	**5a**	Et_2_O	24 h	92	65.5:34.5
21^d^	Bi(OH)_3_	**5a**	Et_2_O	24 h	20	94.8:5.2
22^d^	Bi(O^*i*^Pr)_3_	**5a**	Et_2_O	60 h	94	94.9:5.1
23^d^	Sc(OAc)_3_	**5a**	Et_2_O	72 h	<5	–
24^d^	In(OAc)_3_	**5a**	Et_2_O	72 h	31	55.4:44.6
25^d^	Cu(OAc)_2_	**5a**	Et_2_O	52 h	trace	–
26^d^	AgOAc	**5a**	Et_2_O	25 h	12	55.9:44.1
27^d^	Y(OAc)_3_	**5a**	Et_2_O	25 h	trace	–
28^d^	La(OAc)_3_	**5a**	Et_2_O	48 h	12	69.0:31.0

The reactions were carried out with **1a** (0.1 mmol), **2a** (0.12 mmol), Bi(OAc)_3_ (2 mol%), and CPA (3 mol%) in 0.5 mL solvent at room temperature.

a Yield of isolated products.

b Determined by HPLC analysis.

c The reactions were carried out with **1a** (0.1 mmol), **2a** (0.12 mmol), 10 mol% catalyst in 0.5 mL DCM at room temperature.

d The reactions were carried out with **1a** (0.2 mmol), **2a** (0.24 mmol), Bi(OAc)_3_ (1 mol%), and *(S)*-**5a** (2 mol%) in 1.0 mL Et_2_O at room temperature.

### Substrate Scope

**Figure 1:**
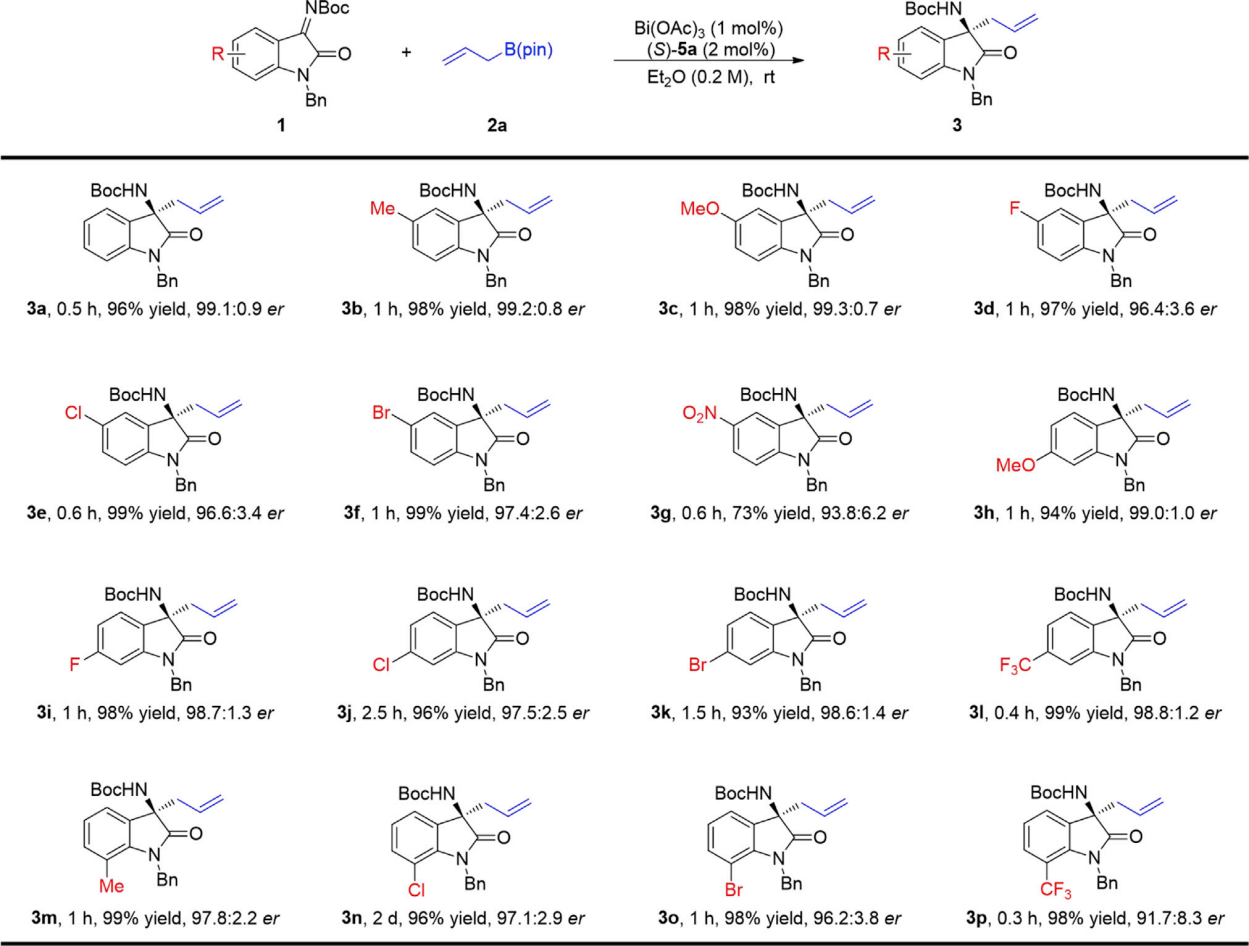
Scope of Substituents on the Phenyl Ring The reactions were carried out with **1** (0.2 mmol), **2a** (0.24 mmol), Bi(OAc)_3_ (1 mol%), and (*S*)-**5a** (2 mol%) in 1.0 mL Et_2_O at room temperature. The absolute configuration of the product was determined by X-ray analysis of **10**. Isolated yields. The *er* values were determined by HPLC analysis.

We then explored the substrate scope of the allylation of isatin-derived ketimines under the optimal reaction conditions. We first investigated the substituents on the phenyl ring of the isatin. As shown in Figure 1, this protocol is amenable to most of *N*-Boc-protected ketimines derived from *N*-benzylisatins bearing electron-donating or electron-withdrawing substituents and halogen atoms on the phenyl ring, leading to chiral 3-allyl 3-aminooxindole products (Figures 1, **3a**-**3p**) in high yields (73%–99%) with good to excellent enantioselectivities (91.7: 8.3–99.3: 0.7 *er*). However, electron-withdrawing substituents on the C5 and C7 of the phenyl ring led to reduced stereoselectivities (Figure 1, **3g** and **3p**).

**Figure 2:**
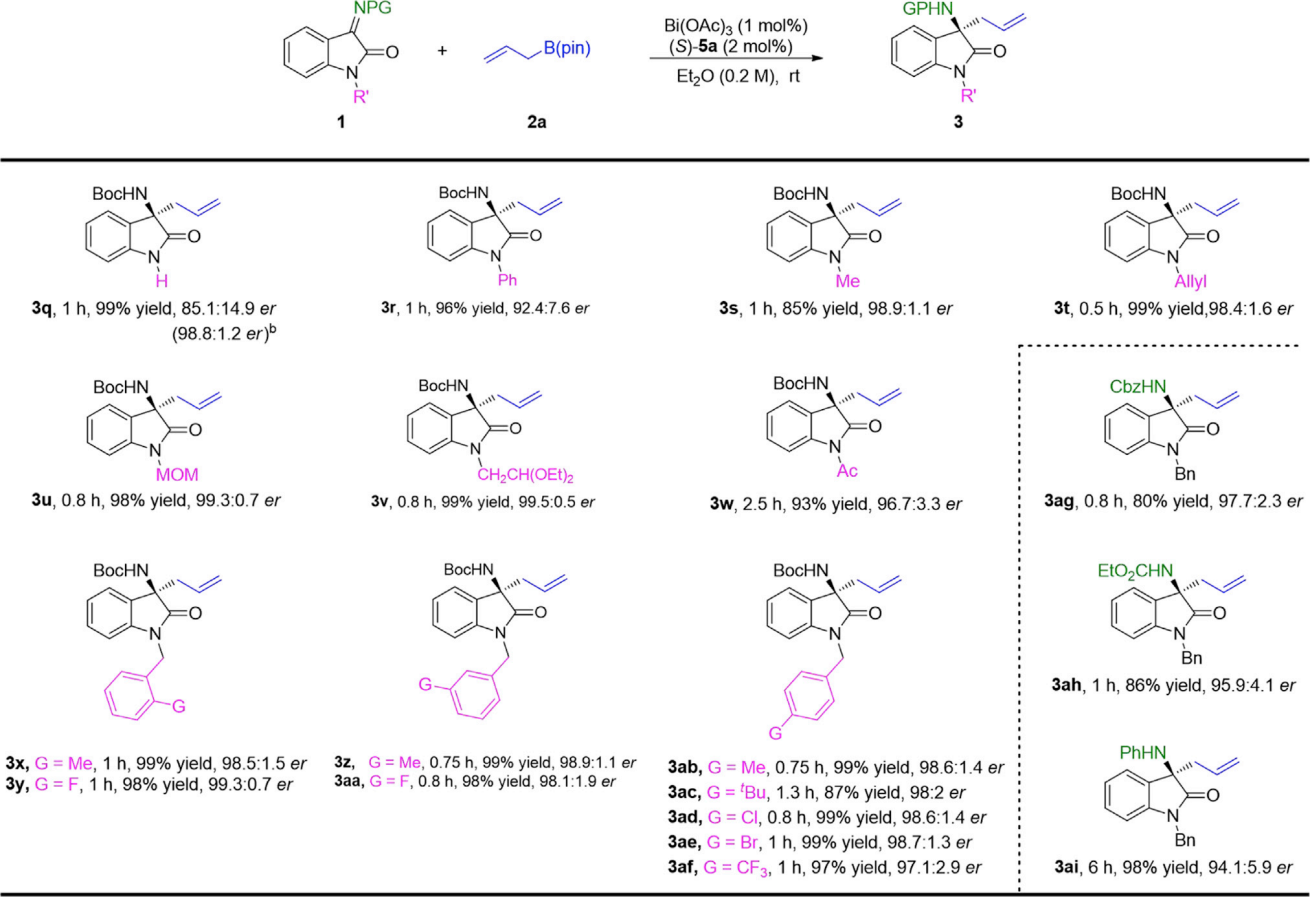
Scope of Protecting Group at the N1-Position and Other N-Substituted Ketimines The reactions were carried out with **1** (0.2 mmol), **2a** (0.24 mmol), Bi(OAc)_3_ (1 mol%), and *(S)*-**5a** (2 mol%) in 1.0 mL Et_2_O at room temperature. Isolated yields. The *er* values were determined by HPLC analysis. The *er* in bracket was afforded after recrystallization from ethyl acetate/*n*-pentane.

Subsequently, the effect of the protecting group at the N1-position were examined (Figure 2). To our delight, the expected product **3q** was afforded from ketimine **1q** without protecting group on the N1-atom in 99% yield and 85.1: 14.9 *er*. An elevated 98.8: 1.2 *er* was obtained after one single recrystallization from ethyl acetate/*n*-pentane. In addition, isatin-derived ketimines with phenyl, acetyl, alkyl (R’ = Me, allyl, methoxymethyl or CH_2_CH(OEt)_2_) at the N1-position, were also efficiently transformed into the corresponding allylic products (**3r**-**3v**) with good to excellent enantioselectivities (92.4: 7.6–99.5: 0.5 *er*). When the substituents at the N1-position of ketimines **1** were substituted benzyl groups, we found that the electron effect or the steric hindrance had almost no effect on the reaction results (Figure 2, **3x**-**3z** and **3aa**–**3af**).

Furthermore, other *N*-alkoxycarbonyl ketimines can also react with **2a** and give the products (**3ag** and **3ah**) in good yields and excellent enantioselectivities (Figure 2). And *N*-phenyl ketimine **1ai** could also be transformed into the corresponding allylation product **3ai** under the optimal conditions in excellent yield (98%) with good enantioselectivity (94.1:5.9 *er*).

**Scheme 2:**
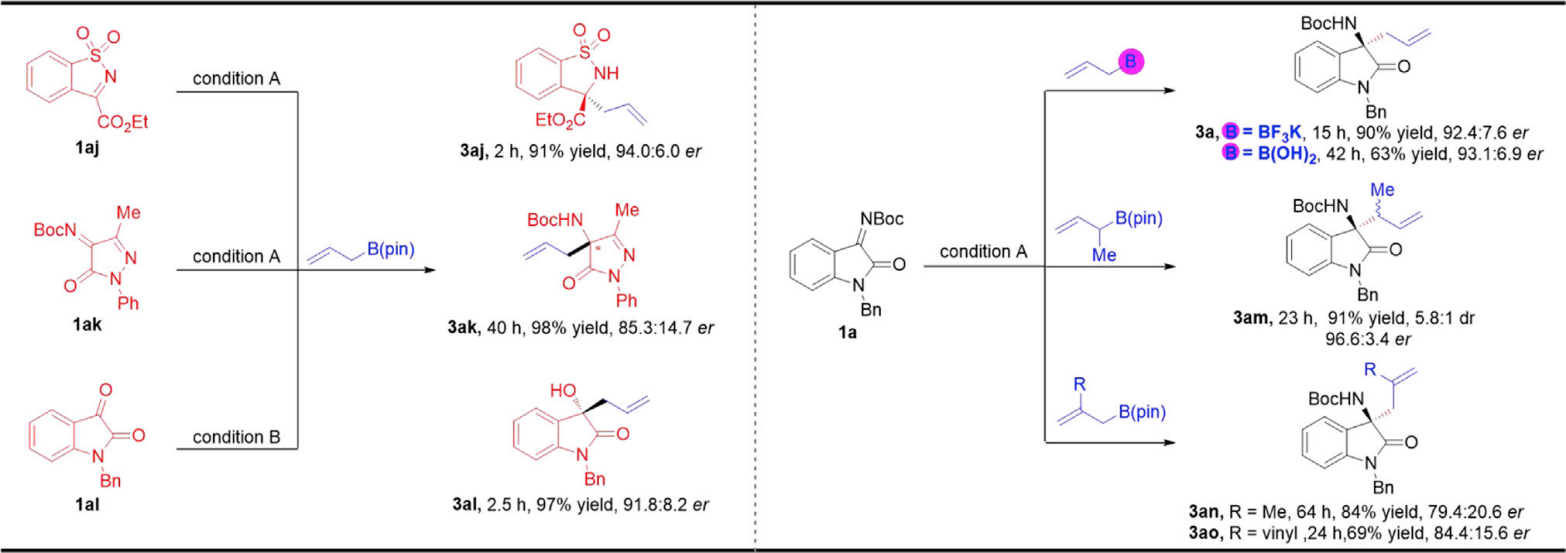
Other Ketimine Skeletons and Scope of Allyl Boron Reagent Condition A: **1** (0.2 mmol), **2a** (0.24 mmol), Bi(OAc)_3_ (1 mol%), and *(S)*-**5a** (2 mol%) in 1.0 mL Et_2_O at room temperature; Condition B: **1al** (0.2 mmol), **2a** (0.24 mmol), Bi(OAc)_3_ (1 mol%), and *(S)*-**6a** (2 mol%) in 1.0 mL cyclohexane at room temperature. Isolated yields. The *er* values were determined by high-performance liquid chromatography (HPLC) analysis.

To expand the scope of this Bi(OAc)_3_/CPA catalyzed asymmetric allylation method, some other ketimines were also investigated (Scheme 2). To our delight, not only the cyclic *N*-sulfonyl α-ketiminoester **1aj** but also the **N**-Boc ketimine **1ak** derived from pyrazolin-5-one could work smoothly under the optimal conditions and give the desired products **3aj** (Wu et al., 2018) and **3ak** in excellent yields with good enantioselectivities. In addition, this catalytic system was also proved to be suitable for the asymmetric allylation of isatin (Scheme 2) (Itoh et al., 2009).

Further exploration of the substrate scope was focused on the allyl boron reagent (Scheme 2). When potassium allyltrifluoroborate and allyl boric acid were used, the corresponding product **3a** was obtained with 92.4:7.6 *er* and 93.1:6.9 *er*, respectively. It should be noted that this Bi(OAc)_3_/CPA catalytic system is applicable to a variety of boron allylation reagent, whereas previous reports are often limited to the particular one. The α-addition product **3am** (91% yield, 5.8:1 d.r., and 96.6:3.4 *er*) resulted in the reaction of **1a** and **2d**. When β-methyl branch allylboronic acid pinacol ester reacted with **1a** under the optimal conditions, the desired product **3an** was obtained in good yield (84%) with depressed enantioselectivity (79.4:20.6 *er*). Moreover, the reaction of pinacolyl isoprenylboronate and ketimine **1a** could also give the desired product **3ao** in good yield (69%) and moderate enantioselectivity (84.4:15.6 *er*).

### Large-Scale Reaction and Synthetic Applications

**Scheme 3:**
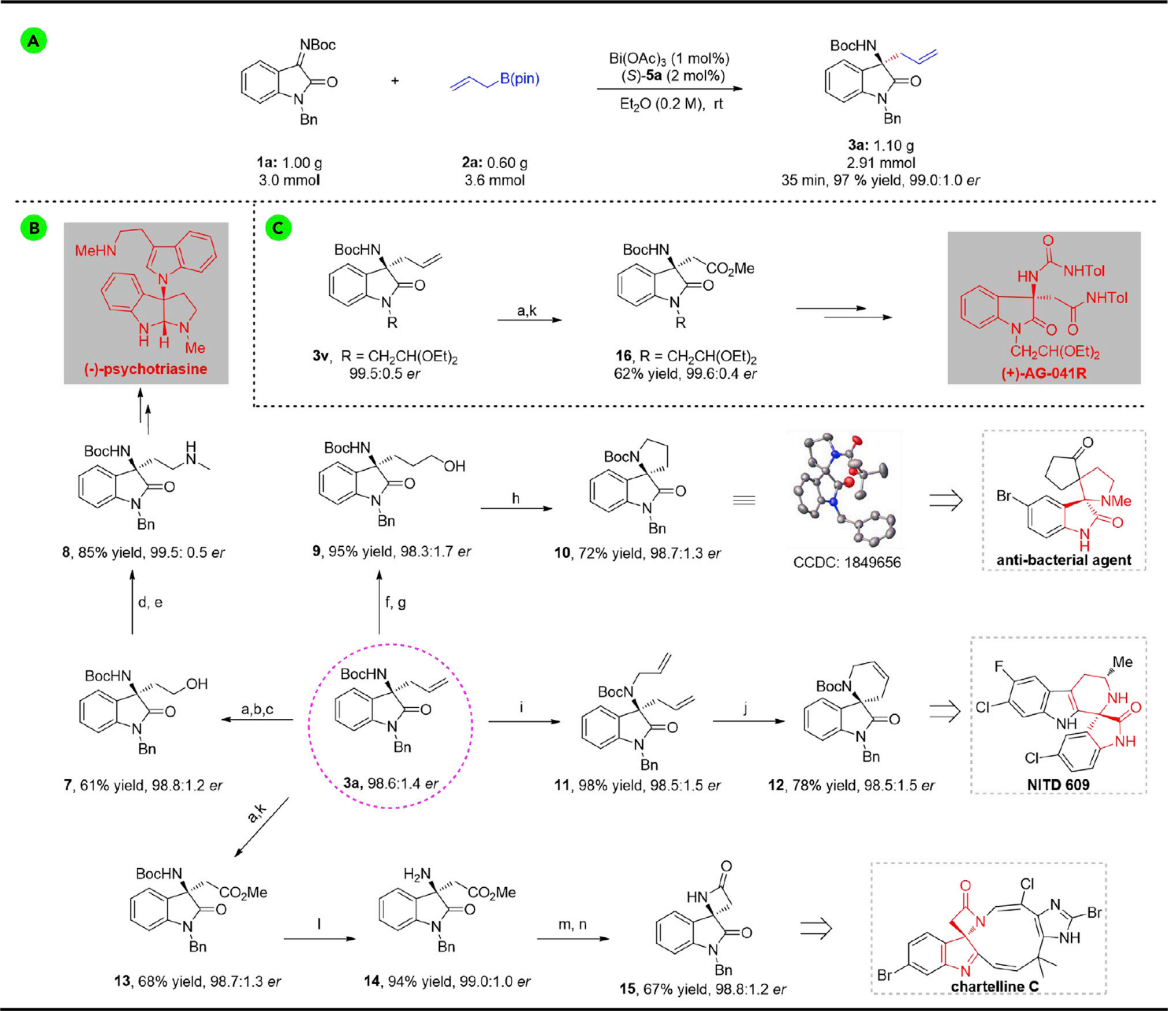
Large-Scale Reaction and Transformations of the Products (A) The gram-scale reaction. (B) The versatile transforms of 3a. (C) The formal synthesis of (+)-AG-041R. Reagents and conditions: (a) KMnO_4_, NaIO_4_, H_2_O, room temperature, 2 d; (b) Et_3_N, ClCO_2_Et, THF, −10°C, 1 h; (c) NaBH_4_, H_2_O, 0°C to room temperature, 4 h; (d) DMP, NaHCO_3_, DCM, room temperature, 1 h; (e) CH_3_NH_2_⋅HCl, Et_3_N, MgSO_4_, MeOH, room temperature, overnight, then NaBH_4_, 0°C; (f) 9-BBN, THF, 0°C to room temperature, 24 h; (g) AcONa, H_2_O_2_ (30% aq.), 0°C to room temperature, 5 h; (h) Ph_3_P, DEAD, DCM, 0°C to room temperature, overnight; (i) NaH, DMF, Allyl bromide, room temperature, 30 min; (j) Grubbs second, toluene, 60°C, 20 min; (k) MeI, Cs_2_CO_3_, CH_3_CN, room temperature, 8 h; (l) TFA, DCM, room temperature, 3 h; (m) 2 M NaOH (aq.), MeOH, 2 h; (n) MsCl, NaHCO_3_, CH_3_CN, 80°C, 18 h.

To probe the efficiency of current asymmetric allylation strategy in preparative synthesis, a gram-scale reaction of **1a** and **2a** was investigated under optimal reaction conditions. To our delight, the corresponding product **3a** was obtained without any loss of the enantioselectivity (Scheme 3A). To illustrate the applicability of our method in organic synthesis, the product was applied to synthesize some pharmaceuticals and *N*-containing heterocyclic oxindole compounds. Firstly, as shown in Scheme 3B, the allylation product **3a** underwent complete oxidation and reduction to give the compound **7**. Compound **7** can be oxidized to an aldehyde intermediate and provided key compound **8** by reductive amination, which can be converted to (−)-psychotriasine (Dai et al., 2017). Compound **3a** underwent hydroboration-oxidation followed by an intramolecular Mitsunobu reaction to afford spirocyclic amine **10** (98.7:1.3 *er*). The N-allylation of **3a** can also offer product **11** in high yield, and its ring-closing metathesis gave spirocyclic amine **12** in high yield with maintained *er* value by using Grubbs second catalyst. In addition, the β-amino ester **13** was afforded by oxidation of **3a** followed by esterification. Boc removal followed by cyclization led to spiro-β-lactam **15** in 67% yield and 98.8:1.2 *er*. Thereafter, oxidation of **3v** followed by an esterification afforded compound **16** without any loss of enantioselectivity (99.6:0.4 *er*). And the compound **16** could be transformed into (+)-AG-041R, which is a potent gastrin/CCK-B receptor antagonist (Scheme 3C) (Sato et al., 2009).

### Mechanistic Considerations

We performed control experiments to investigate whether bismuth acetate and chiral phosphoric acid work in a synergic manner on the activity and enantioselectivity of the asymmetric allylation (Table 2). The reaction proceeded smoothly in the presence of Bi(OAc)_3_ and gave a racemic product (Table 2, entry 1). When only chiral phosphoric acid *(S)*-**5a** existed, 14% yield and 62.4: 37.6 *er* could be achieved in 48 h (Table 2, entry 2). Considering that the hydrolysis of Bi(OAc)_3_ produces acetic acid, we performed the reaction under the condition of only 2 mol% AcOH, and 12% racemic product could be given (Table 2, entry 3). If adding 3 mol% *(S)*-**5a** on the basis of condition C, we could afford the product in 17% yield with 71.6: 28.4 *er* in 48 h (Table 2, entry 4). Therefore, the effect of Lewis acid's hydrolysis on the reaction results could be excluded. These experimental results demonstrated that the reactivity and stereoselectivity should be controlled by Bi(OAc)_3_ and chiral phosphoric acid together.

**Table 2:** Control Experiments


Entry	Conditions	Results
1	Only 2 mol% Bi(OAc)_3_	1.5 h, 99% yield, rac
2	Only 3 mol% (*S*)-**5a**	48 h, 14% yield, 62.4:37.6 *er*
3	Only 2 mol% AcOH	48 h, 12% yield, rac
4	2 mol% AcOH +3 mol% (*S*)-**5a**	48 h, 17% yield, 71.6:28.4 *er*

**Figure 3:**
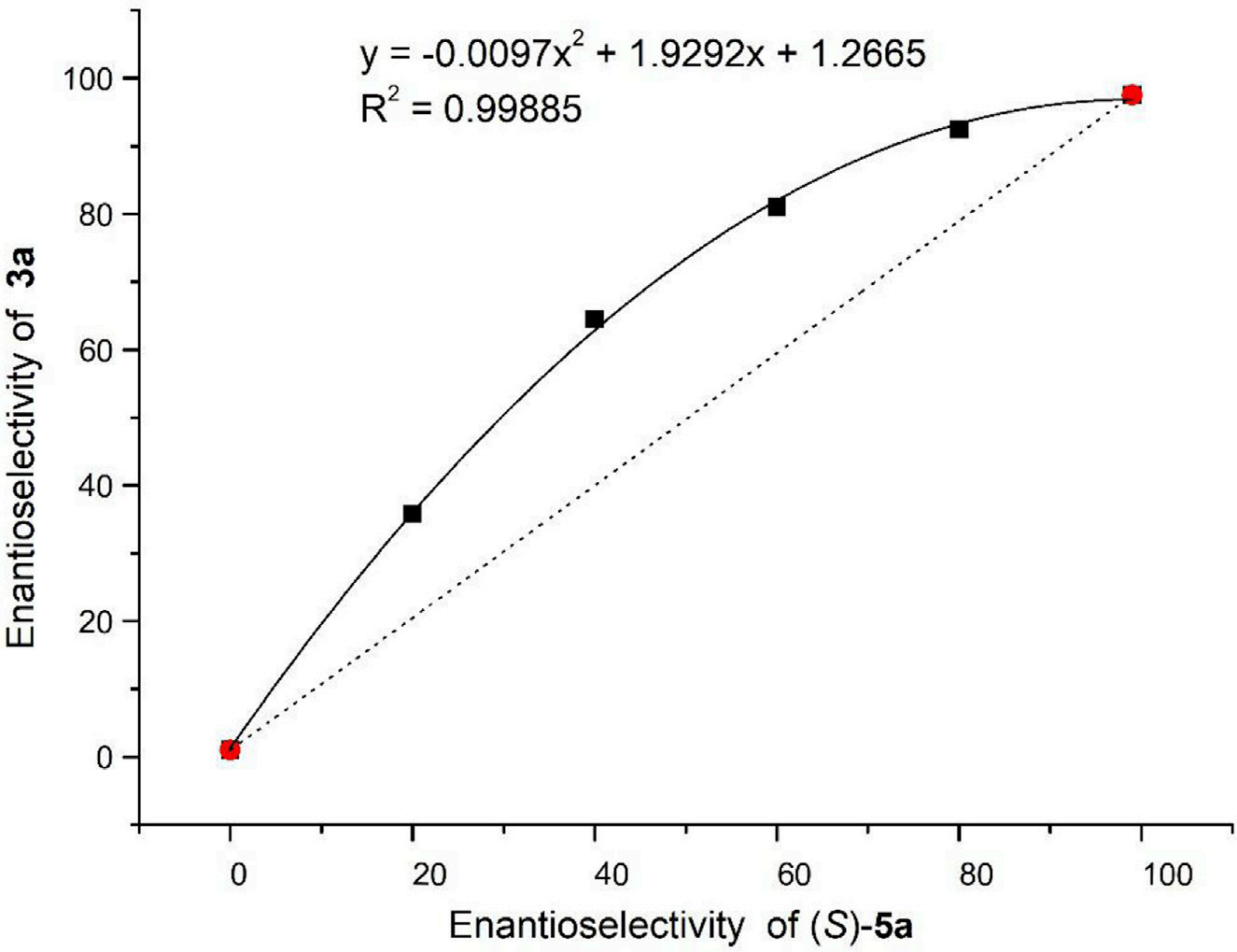
Nonlinear Effect Experiment For the major diastereomer, determined by HPLC analysis on a chiral stationary phase, averaged over two runs (see also Table S3).

Preliminary experiments were conducted to illustrate the mechanism of the Bi(OAc)_3_/CPA catalytic system. ESI-MS experiment (cationic mode) gave two peaks m/z 1027.27 and 1728.45 corresponding to **5a**⋅Bi(OAc)_2_ and (**5a**)_2_⋅Bi(OAc)_2_ (for details, see Supplemental Information). A positive nonlinear effect between the catalyst's *er* value and product's *er* value was observed under optimal reaction conditions (Figure 3) (Liu et al., 2011, Wang et al., 2017), which indicates that more than one molecule of the chiral acid *(S)*-**5a** is likely to be involved in the transition state of the enantio-differentiating step. The α-selectivity was observed with 1-methylallylboronic acid pinacol ester (Scheme 2, substrate scope part); thus, we speculated that the reaction should occur through a B-to-Bi transmetalation process (Chakrabarti et al., 2010).

**Figure 4:**
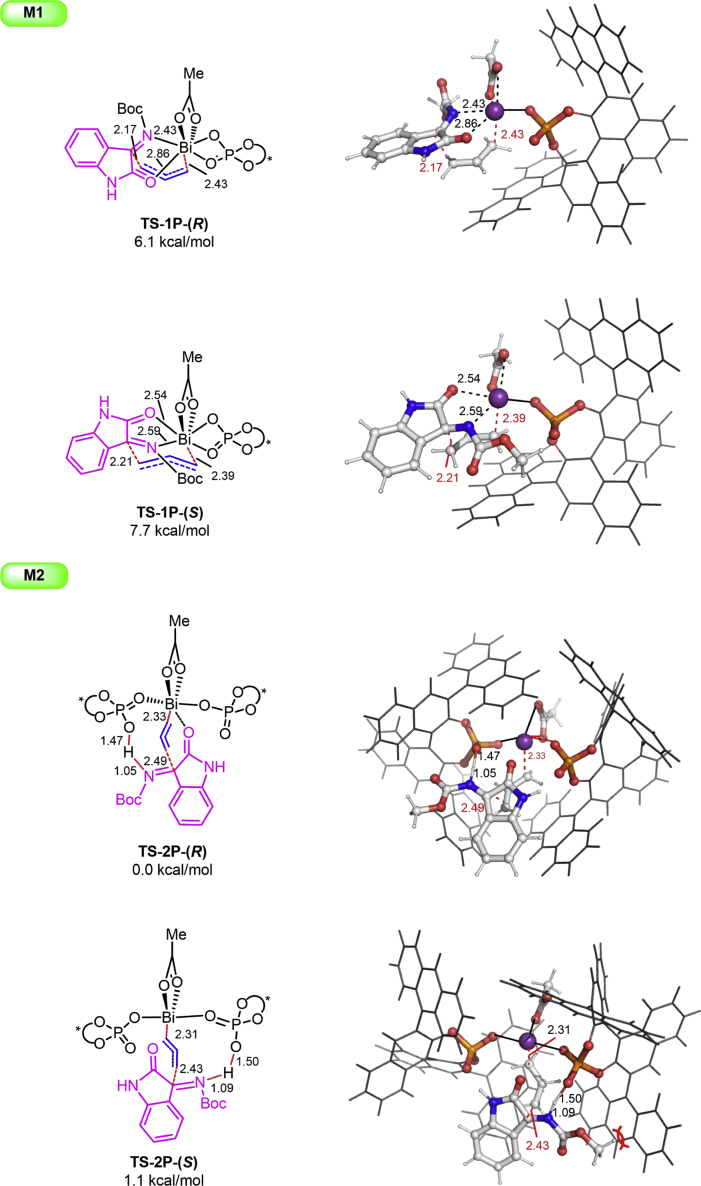
Transition State Structures and Relative Free Energies (in kcal/mol) See also Figures S167–S170; and Tables S3 and S4–S10.

The mechanism of the catalytic system has been further investigated by theoretical calculations (for computational details, see Supplemental Information). Two mechanistic possibilities that differ by the coordination number were considered. A single CPA ligand is present in **M1**, whereas two chiral ligands are present in **M2** (Figure 4). Mechanism **M1** can be discarded based on the large energy barrier (at least 6.1 kcal/mol unfavorable) in which the single CPA served as a typical anionic ligand. Two CPA ligands perform different roles in **M2**, in which one serves as a typical anionic ligand and the other performs as a neutral ligand and acid catalyst simultaneously. We have examined different relative orientations of substrate **1q** and Bi-allyl species (details in the Supplemental Information), and the most stable **TSs** corresponding to the structure was shown (**M2** in Figure 4). On examination of **TS-2p-(R)**, we found that the C=O group of the ketimine is coordinated with the Bi and the C=N group is activated by the proton of phosphoric acid simultaneously. In the most stable **TS-2P-(R)**, substrate **1q** is oriented with the bulky Boc group into an open quadrant of the catalyst and **TS-2P-(S)** with the bulky Boc group toward the catalyst lying 1.1 kcal/mol above the most stable **TS**. Calculations predict 86.5: 13.5 *er* for the *(R)*-product, which is consistent well with experimental 85.1: 14.9 *er*.

### Limitations of Study

The reaction only gave poor yield (30%) and poor enantioselectivity (57.0:43.0 *er*) with the widely used Bi(OTf)_3_ instead of Bi(OAc)_3_ (Table 1, entry 17).

### Conclusion

In summary, we have developed a highly efficient and enantioselective asymmetric allylation of isatin-derived ketimines with allylboronates promoted by a binary acid system containing bismuth acetate and chiral phosphoric acid. As far as we know, this is the first successful application of the catalyst system of Bi(III) Lewis acid and chiral phosphoric acid in asymmetric catalysis. This is an unreported catalytic system in asymmetric allylation of ketimines. As a result, a series of chiral 3-allyl 3-aminooxindoles were obtained in excellent yields (up to 99%) and enantioselectivities (up to 99.5: 0.5 *er*). The synthetic utility was demonstrated not only by formal synthesis of (+)-AG-041R and (−)-psychotriasine but also by the transformation of the allylation products into valuable chiral 3-spirocyclic oxindoles. Preliminary mechanism study by control experiments and theoretical calculations shows that two chiral phosphoric acids, in which one serves as an anionic ligand and the other performs as a neutral ligand and acid catalyst simultaneously, have participated in this allylation strategy. We anticipate that this work will provide a broad prospect for the future application of bismuth in asymmetric catalysis.

## Methods

All methods can be found in the accompanying Transparent Methods supplemental file.

## Data and Software Availability

The crystallography data have been deposited at the Cambridge Crystallographic Data Center (CCDC) under accession number CCDC: 1849656 (**10**) and can be obtained free of charge from www.ccdc.cam.ac.uk/getstructures.

## Supplemental Information

Document S1. Transparent Methods, Figures S1–S170, Tables S1–S3, and Data S1 and S2

## References

[bib1] Alam, R., Vollgraff, T., Eriksson, L. and Szabo, K.J.. (2015). Synthesis of adjacent quaternary stereocenters by catalytic asymmetric allylboration. J. Am. Chem. Soc. 137, 11262-11265. 10.1021/jacs.5b07498 26316158

[bib2] Barnett, D.S., Moquist, P.N. and Schaus, S.E.. (2009). The mechanism and an improved asymmetric allylboration of ketones catalyzed by chiral biphenols. Angew. Chem. Int. Ed. 48, 8679-8682. 10.1002/anie.200904715 PMC2819187 19816902

[bib3] Bothwell, J.M., Krabbez, S.W. and Mohan, R.S.. (2011). Applications of bismuth(III) compounds in organic synthesis. Chem. Soc. Rev. 40, 4649-4707. 10.1039/c0cs00206b 21589974

[bib4] Cao, Z.-Y., Zhou, F. and Zhou, J.. (2018). Development of synthetic methodologies via catalytic enantioselective synthesis of 3,3-disubstituted oxindoles. Acc. Chem. Res. 51, 1443-1454. 10.1021/acs.accounts.8b00097 29808678

[bib5] Chakrabarti, A., Konishi, H., Yamaguchi, M., Schneider, U. and Kobayashi, S.. (2010). Indium(I)-catalyzed asymmetric allylation, crotylation, and α-chloroallylation of hydrazones with rare constitutional and high configurational selectivities. Angew. Chem. Int. Ed. 49, 1838-1841. 10.1002/anie.200906308 20135658

[bib6] Chen, J.L.-Y. and Aggarwal, V.K.. (2014). Highly diastereoselective and enantiospecific allylation of ketones and imines using borinic esters: contiguous quaternary stereogenic centers. Angew. Chem. Int. Ed. 53, 10992-10996. 10.1002/anie.201407127 25156948

[bib7] Chen, T. and Cai, C.. (2016). Imidazolylpyridine-In(OTf)3 catalyzed enantioselective allylation of ketimines derived from isatins. Org. Biomol. Chem. 14, 5019-5022. 10.1039/c6ob00551a 27193447

[bib8] Dai, J., Xiong, D., Yuan, T., Liu, J., Chen, T. and Shao, Z.. (2017). Chiral primary amine catalysis for asymmetric Mannich reactions of aldehydes with ketimines: stereoselectivity and reactivity. Angew. Chem. Int. Ed. 56, 12697-12701. 10.1002/anie.201706304 28786162

[bib9] Ghosh, A.K., Schiltz, G., Perali, R.S., Leshchenko, S., Kay, S., Walters, D.E., Koh, Y., Maeda, K. and Mitsuya, H.. (2006). Design and synthesis of novel HIV-1 protease inhibitors incorporating oxyindoles as the P’2-ligands. Bioorg. Med. Chem. Lett. 16, 1869-1873. 10.1016/j.bmcl.2006.01.011 16480871

[bib10] Guan, J., Mathai, S., Harris, P., Wen, J.-Y., Zhang, R., Brimble, M. and Gluckman, P.. (2003). Peripheral administration of a novel diketopiperazine, NNZ 2591, prevents brain injury and improves somatosensory-motor function following hypoxia-ischemia in adult rats. Neuropharmacology. 53, 749-762. 10.1016/j.neuropharm.2007.08.010 17904590

[bib11] Hashimoto, T., Galvez, A.O. and Maruoka, K.. (2013). In situ assembled boronate ester assisted chiral carboxylic acid catalyzed asymmetric trans- aziridinations. J. Am. Chem. Soc. 135, 17667-17670. 10.1021/ja407764u 24199743

[bib12] Hatano, M., Moriyama, K., Maki, T. and Ishihara, K.. (2010). Which is the actual catalyst: chiral phosphoric acid or chiral calcium phosphate. Angew. Chem. Int. Ed. 49, 3823 -3826. 10.1002/anie.201000824 20408153

[bib13] Hatano, M., Goto, Y., Izumiseki, A., Akakura, M. and Ishihara, K.. (2015). Boron tribromide-assisted chiral phosphoric acid catalyst for a highly enantioselective Diels-Alder reaction of 1, 2- dihydropyridines. J. Am. Chem. Soc. 137, 13472-13475. 10.1021/jacs.5b08693 26457929

[bib14] Hepburn, H.B. and Lam, H.W.. (2014). The isomerization of allylrhodium intermediates in the rhodium-catalyzed nucleophilic allylation of cyclic imines. Angew. Chem. Int. Ed. 53, 11605-11610. 10.1002/anie.201407233 PMC4497600 25205604

[bib15] Hepburn, H.B., Chotsaeng, N., Luo, Y. and Lam, H.W.. (2013). Enantioselective rhodium-catalyzed allylation of cyclic imines with potassium allyltrifluoroborates. Synthesis. 45, 2649-2661.

[bib16] Huo, H.X., Duvall, J.R., Huang, M.Y. and Hong, R.. (2014). Catalytic asymmetric allylation of carbonyl compounds and imines with allylic boronates. Org. Chem. Front. 1, 303-320.

[bib17] Isomura, M., Petrone, D.A. and Carreira, E.M.. (2019). Coordination-induced stereocontrol over carbocations: asymmetric reductive deoxygenation of racemic tertiary alcohols. J. Am. Chem. Soc. 141, 4738−4748. 10.1021/jacs.9b00862 30785741

[bib18] The absolute configuration of the product is determined by comparison with the data in the literature: Itoh, J., Han, S.B. andKrische, M.J. (2009). Enantioselective allylation, crotylation, and reverse prenylation of substituted isatins: iridium-catalyzed C-C bond- forming transfer hydrogenation. Angew. Chem. Int. Ed. 48, 6313-6316. 10.1002/anie.200902328 PMC2846777 19606435

[bib19] Jain, P. and Antilla, J.C.. (2010). Chiral Bronsted acid-catalyzed allylboration of aldehydes. J. Am. Chem. Soc. 132, 11884-11886. 10.1021/ja104956s PMC2928988 20690662

[bib20] Jang, H., Romiti, F., Torker, S. and Hoveyda, A.H.. (2017). Catalytic diastereo- and enantioselective additions of versatile allyl groups to N-H ketimines. Nat. Chem. 9, 1269-1275. 10.1038/nchem.2816 PMC5726442 29168479

[bib21] Jiang, Y. and Schaus, S.E.. (2017). Asymmetric petasis borono-mannich allylation reactions catalyzed by chiral biphenols. Angew. Chem. Int. Ed. 56, 1544-1548. 10.1002/anie.201611332 PMC5716625 28052567

[bib22] Jiang, Y., Diagne, A.B., Thomson, R.J. and Schaus, S.E.. (2017a). Enantioselective synthesis of allenes by catalytic traceless petasis reactions. J. Am. Chem. Soc. 139, 1998-2005. 10.1021/jacs.6b11937 PMC5716636 28121128

[bib23] Jiang, Y., Thomson, R.J. and Schaus, S.E.. (2017b). Asymmetric traceless petasis borono-mannich reactions of enals: reductive transposition of allylic diazenes. Angew. Chem. Int. Ed. 56, 16631-16635. 10.1002/anie.201708784 PMC5739942 29110383

[bib24] Kennedy, J.W.J. and Hall, D.G.. (2003). Recent advances in the activation of boron and silicon reagents for stereocontrolled allylation reactions. Angew. Chem. Int. Ed. 42, 4732-4739. 10.1002/anie.200301632 14562340

[bib25] Kitanosono, T., Ollevier, T. and Kobayashi, S.. (2013). Iron- and bismuth-catalyzed asymmetric Mukaiyama aldol reactions in aqueous media. Chem. Asian J. 8, 3051-3062. 10.1002/asia.201301149 24101589

[bib26] Kobayashi, S. and Ogawa, C.. (2006). New entries to water-compatible Lewis acids. Chem. Eur. J. 12, 5954-5960. 10.1002/chem.200600385 16773666

[bib27] Kobayashi, S., Ogino, T., Shimizu, H., Ishikawa, S., Hamada, T. and Manabe, K.. (2005). Bismuth triflate-chiral bipyridine complexes as water-compatible chiral Lewis acids. Org. Lett. 7, 4729-4731. 10.1021/ol051965w 16209521

[bib28] Koch, F.M. and Peters, R.. (2007). Catalytic enantio- and diastereoselective formation of β-sultones: ring-strained precursors for enantioenriched β-hydroxysulfonyl derivatives. Angew. Chem. Int. Ed. 46, 2685-2689. 10.1002/anie.200604796 17330913

[bib29] Koch, F.M. and Peters, R.. (2011). Lewis acid/base catalyzed [2+2]-cycloaddition of sulfenes and aldehydes: a versatile entry to chiral sulfonyl and sulfinyl derivatives. Chem. Eur. J. 17, 3679-3692. 10.1002/chem.201003542 21365709

[bib30] Kumar, D., Vemula, S.R., Balasubramanian, N. and Cook, G.R.. (2016). Indium-mediated stereoselective allylation. Acc. Chem. Res. 49, 2169-2178. 10.1021/acs.accounts.6b00362 27700084

[bib31] Lassauque, N., Francio, G. and Leitner, W.. (2009). Nickel-catalyzed asymmetric hydrovinylation using Lewis acid activation. Eur. J. Org. Chem. 2009, 3199-3202.

[bib32] Lathrop, S.P., Pompeo, M., Chang, W.T. and Movassaghi, M.. (2016). Convergent and biomimetic enantioselective total synthesis of (−) - Communesin F. J. Am. Chem. Soc. 138, 7763-7769. 10.1021/jacs.6b04072 PMC4944760 27244250

[bib33] Li, Z., Plancq, B. and Ollevier, T.. (2012). Bismuth triflate-catalyzed asymmetric allylation of aromatic aldehydes. Chem. Eur. J. 18, 3144-3147. 10.1002/chem.201103646 22337608

[bib34] Li, G., Liang, T., Wojtas, L. and Antilla, J.C.. (2013). An asymmetric Diels-Alder reaction catalyzed by chiral phosphate magnesium complexes: highly enantioselective synthesis of chiral spirooxindoles. Angew. Chem. Int. Ed. 52, 4628 -4632. 10.1002/anie.201209295 23519960

[bib35] Liu, X., Lin, L. and Feng, X.. (2011). Chiral N, N’-dioxides: new ligands and organocatalysts for catalytic asymmetric reactions. Acc. Chem. Res. 44, 574-587. 10.1021/ar200015s 21702458

[bib36] Liu, X., Lin, L. and Feng, X.. (2014). Chiral N, N’-dioxide ligands: synthesis, coordination chemistry and asymmetric catalysis. Org. Chem. Front. 1, 298-302.

[bib37] Lou, S. and Schaus, S.E.. (2008). Asymmetric petasis reactions catalyzed by chiral biphenols. J. Am. Chem. Soc. 130, 6922-6923. 10.1021/ja8018934 PMC2440570 18459782

[bib38] Lou, S., Moquist, P.N. and Schaus, S.E.. (2006). Asymmetric allylboration of ketones catalyzed by chiral diols. J. Am. Chem. Soc.. 128, 12660-12661. 10.1021/ja0651308 17002355

[bib39] Lou, S., Moquist, P.N. and Schaus, S.E.. (2007). Asymmetric allylboration of acyl imines catalyzed by chiral diols. J. Am. Chem. Soc. 129, 15398-15404. 10.1021/ja075204v PMC2638762 18020334

[bib40] Luo, Y., Hepburn, H.B., Chotsaeng, N. and Lam, H.W.. (2012). Enantioselective rhodium-catalyzed nucleophilic allylation of cyclic imines with allylboron reagents. Angew. Chem. Int. Ed. 51, 8309-8313. 10.1002/anie.201204004 22786686

[bib41] Lv, J. and Luo, S.. (2013). Asymmetric binary acid catalysis: chiral phosphoric acid as dual ligand and acid. Chem. Commun.. 49, 847-858. 10.1039/c2cc34288j 23150877

[bib42] Lv, J., Zhang, L., Zhou, Y., Nie, Z., Luo, S. and Cheng, J.-P.. (2011). Asymmetric binary acid catalysis: a regioselectivity switch between enantioselective 1, 2- and 1, 4- addition through different counteranions of InIII. Angew. Chem. Int. Ed. 50, 6610-6614. 10.1002/anie.201101254 21648033

[bib43] Lv, J., Zhang, L., Luo, S. and Cheng, J.-P.. (2013). Switchable diastereoselectivity in enantioselective [4+2] cycloadditions with simple olefins by asymmetric binary acid catalysis. Angew. Chem. Int. Ed. 52, 9786-9790. 10.1002/anie.201304561 23897700

[bib44] Mahajan, N., Koul, S. and Razdan, T.K.. (2011). Bismuth triflate- L(-)- proline catalyzed synthesis of chiral 2, 5- diaryl-2, 3- dihydropyrano[2, 3-b]quinolin- 4- ones. J. Heterocyclic Chem. 48, 1302-1307.

[bib45] Mlynarski, J.. (2017). Chiral Lewis Acids in Organic Synthesis (Wiley-VCH, Weinheim, Germany).

[bib46] Nakamura, S., Hyodo, K., Nakamura, M., Nakane, D. and Masuda, H.. (2013). Catalytic enantioselective allylation of ketimines by using palladium pincer complexes with chiral bis(imidazoline)s. Chem. Eur. J. 19, 7304-7309. 10.1002/chem.201300685 23633426

[bib47] Ollevier, T.. (2013). New trends in bismuth-catalyzed synthetic transformations. Org. Biomol. Chem. 11, 2740-2755. 10.1039/c3ob26537d 23380745

[bib48] Ondet, P., Lemiere, G. and Duñach, E.. (2017). Cyclisations catalysed by bismuth(III) triflate. Eur. J. Org. Chem. 2017 761-780.

[bib49] Salvador, J.A.R., Figueiredo, S.A.C., Pinto, R.M.A. and Silvestre, S.M.. (2012). Bismuth compounds in medicinal chemistry. Future Med. Chem. 4, 1495-1523. 10.4155/fmc.12.95 22857536

[bib50] Sato, S., Shibuya, M., Kanoh, N. and Iwabuchi, Y.. (2009). An expedient route to a potent gastrin/CCK-B receptor antagonist (+)-AG-041R. J. Org. Chem. 74, 7522-7524. 10.1021/jo901352u 19719158

[bib51] Silverio, D.L., Torker, S., Pilyugina, T., Vieira, E.M., Snapper, M.L., Haeffner, F. and Hoveyda, A.H.. (2013). Simple organic molecules as catalysts for enantioselective synthesis of amines and alcohols. Nature. 494, 216-221. 10.1038/nature11844 PMC3576146 23407537

[bib52] Singh, G.S. and Desta, Z.Y.. (2012). Isatins as privileged molecules in design and synthesis of spiro-fused cyclic frameworks. Chem. Rev. 112, 6104 -6155. 10.1021/cr300135y 22950860

[bib53] Sirasani, G. and Andrade, R.B.. (2011). Total synthesis of (−) - leuconicine a and B. Org. Lett. 13, 4736-4737. 10.1021/ol202056w 21812452

[bib54] Tan, Q., Wang, X., Xiong, Y., Zhao, Z., Li, L., Tang, P. and Zhang, M.. (2017). Chiral amino alcohol accelerated and stereocontrolled allylboration of iminoisatins: highly efficient construction of adjacent quaternary stereogenic centers. Angew. Chem. Int. Ed. 56, 4829-4833. 10.1002/anie.201700581 28338268

[bib55] Wada, M., Takahashi, T., Domae, T., Fukuma, T., Miyoshi, N. and Smith, K.. (1997). Asymmetric trimethylsilylcyanation of aldehydes utilizing chiral bismuth compounds. A frontier in bismuth mediated synthetic reactions. Tetrahedron Asymmetry. 8, 3939-3946.

[bib56] Wada, R., Shibuguchi, T., Makino, S., Oisaki, K., Kanai, M. and Shibasaki, M.. (2006). Catalytic enantioselective allylation of ketoimines. J. Am. Chem. Soc. 128, 7687-7691. 10.1021/ja061510h 16756326

[bib57] Wan, L., Tian, L., Liu, J. and Niu, D.. (2017). Iridium-catalyzed asymmetric umpolung allylation of N-fluorenyl imines to prepare 1, 4-disubstituted homoallylic amines. Synlett. 28, 2051-2056.

[bib58] Wang, L., Lv, L., Zhang, J. and Luo, S.. (2017). Catalytic regio- and enantioselective [4+2] annulation reactions of non-activated allenes by a chiral cationic indium complex. Angew. Chem. Int. Ed. 56, 10867-10871. 10.1002/anie.201704020 28707819

[bib59] Wu, H., Haeffner, F. and Hoveyda, A.H.. (2014). An efficient, practical, and enantioselective method for synthesis of homoallenylamides catalyzed by an aminoalcohol-derived, boron-based catalyst. J. Am. Chem. Soc. 136, 3780-3783. 10.1021/ja500374p PMC3985786 24588835

[bib60] Wu, L., Shao, Q., Yang, G. and Zhang, W.. (2018). Cobalt-catalyzed asymmetric allylation of cyclic ketimines. Chem. Eur. J. 24, 1241-1245. 10.1002/chem.201704760 29120070

[bib61] Yamamoto, H.. (2000). Lewis Acids in Organic Synthesis (Wiley-VCH, Weinheim, Germany).

[bib62] Yamamoto, H. and Futatsugi, K.. (2005). “Designer acids”: combined acid catalysis for asymmetric synthesis. Angew. Chem. Int. Ed. 44, 1924-1942. 10.1002/anie.200460394 15770618

[bib63] Yamamoto, H. and Ishihara, K.. (2008). Acid Catalysis in Modern Organic Synthesis (Wiley-VCH, Weinheim, Germany).

[bib64] Yus, M., Gonzalez-Gomez, J.C. and Foubelo, F.. (2011). Catalytic enantioselective allylation of carbonyl compounds and imines. Chem. Rev. 111, 7774-7854. 10.1021/cr1004474 21923136

[bib65] Zhang, Z., Zheng, W. and Antilla, J.C.. (2011). Highly enantioselective catalytic benzoyloxylation of 3-aryloxindoles using chiral VAPOL calcium phosphate. Angew. Chem. Int. Ed. 50, 1135 -1138. 10.1002/anie.201006595 PMC3115553 21268212

[bib66] Zhang, L., Zhang, J., Ma, J., Cheng, D.-J. and Tan, B.. (2017a). Highly atroposelective synthesis of arylpyrroles by catalytic asymmetric Paal-Knorr reaction. J. Am. Chem. Soc. 139, 1714-1717. 10.1021/jacs.6b09634 28106384

[bib67] Zhang, Z.-P., Dong, N. and Li, X.. (2017b). Bismuth-catalyzed allylation of para-quinone methides. Chem. Commun. (Camb.) 53, 1301-1304. 10.1039/c6cc06605d 28070583

[bib68] Zheng, W., Zhang, Z., Kaplan, M.J. and Antilla, J.C.. (2011). Chiral calcium VAPOL phosphate mediated asymmetric chlorination and Michael reactions of 3-substituted oxindoles. J. Am. Chem. Soc. 133, 3339-3341. 10.1021/ja109824x PMC3103939 21341790

[bib69] Zhou, F., Liu, Y.-L. and Zhou, J.. (2010). Catalytic asymmetric synthesis of oxindoles bearing a tetrasubstituted stereocenter at the C-3 position. Adv. Synth. Catal. 352, 1381 -1407.

