# Supplementary material for: Bi(III)-Catalyzed Enantioselective Allylation Reactions of Ketimines
Source: iScience. 2019 Jun 11;16:511–23. doi: 10.1016/j.isci.2019.06.006 (PMC6593186; doi:10.1016/j.isci.2019.06.006)
Supplement: Document S1. Transparent Methods, Figures S1–S170, Tables S1–S3, and Data S1 and S2 [file mmc1.pdf]

ISCI, Volume *16*

Supplemental Information

Bi(III)-Catalyzed Enantioselective Allylation Reactions of Ketimines

Jie Wang, Qingxia Zhang, Biying Zhou, Chen Yang, Xin Li, and Jin-Pei Cheng

Supplementary Figures

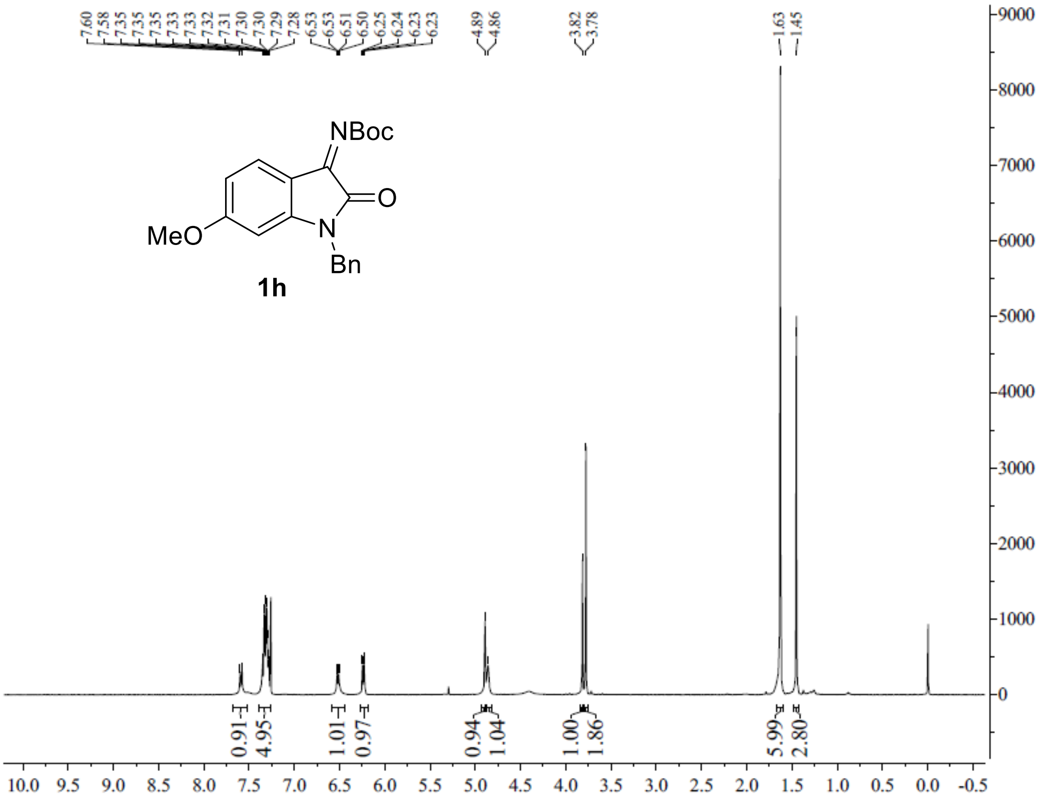

Figure S1. ^{1}H NMR spectrum of **1h**, related to **Figure 1**.

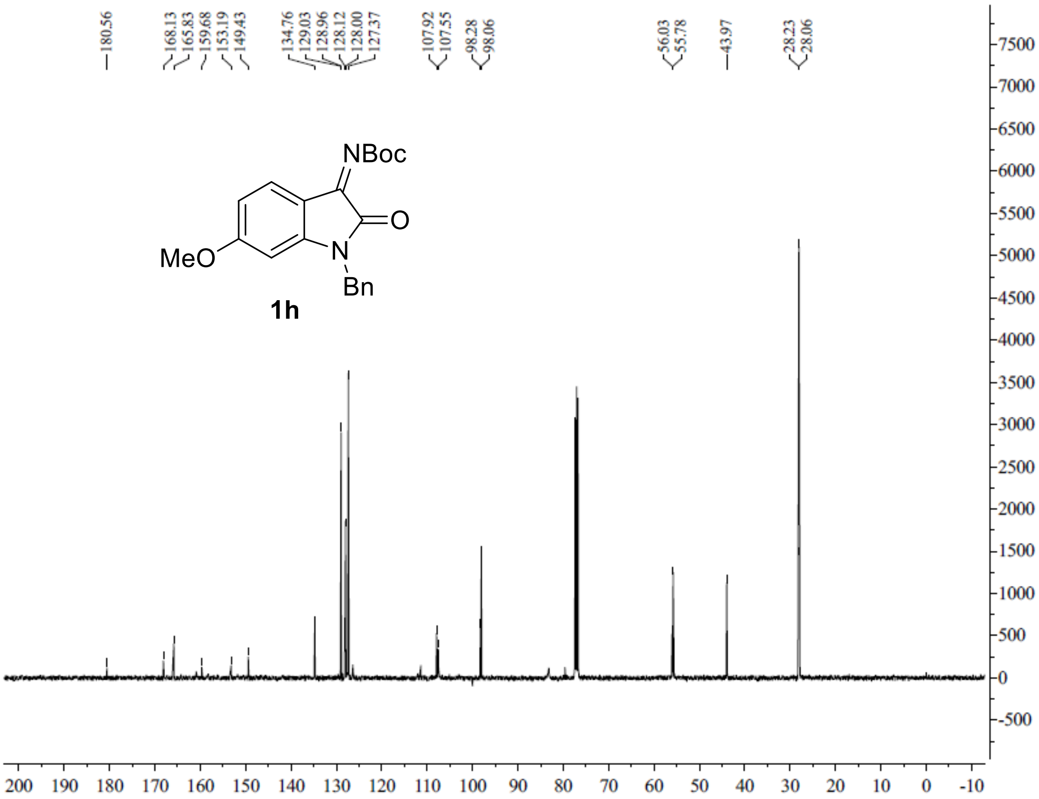

Figure S2. ^{13}C NMR spectrum of **1h**, related to **Figure 1.**

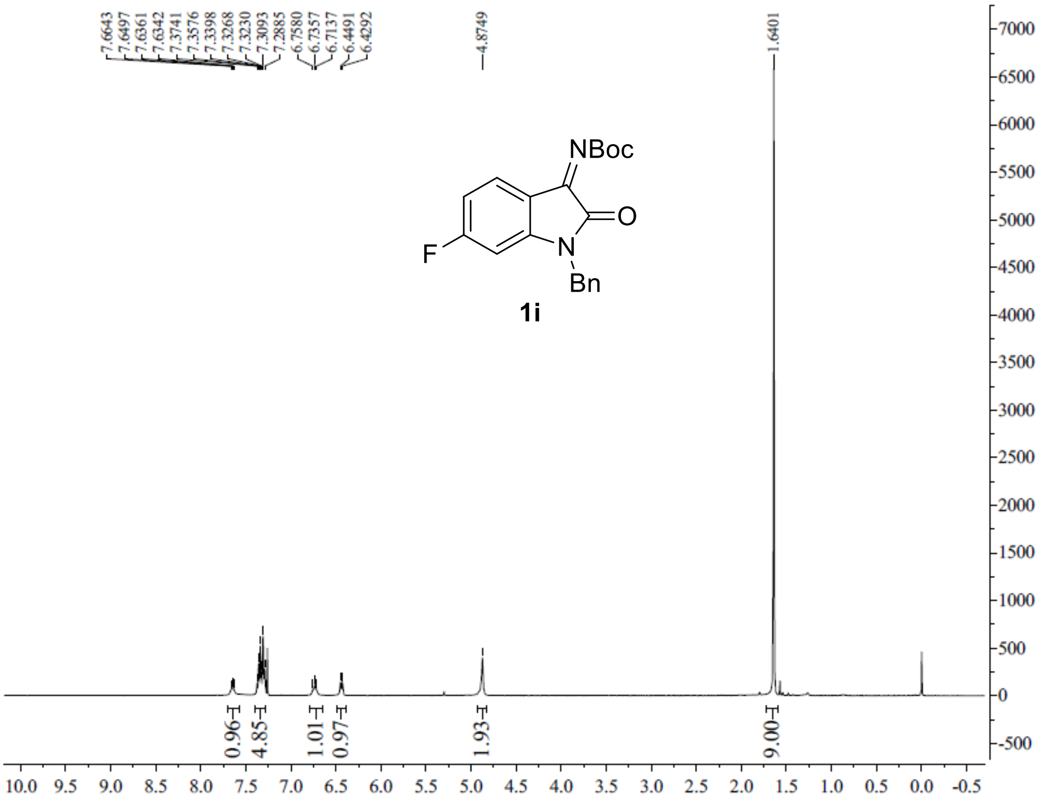

Figure S3. ^{1}H NMR spectrum of **1i**, related to **Figure 1**.

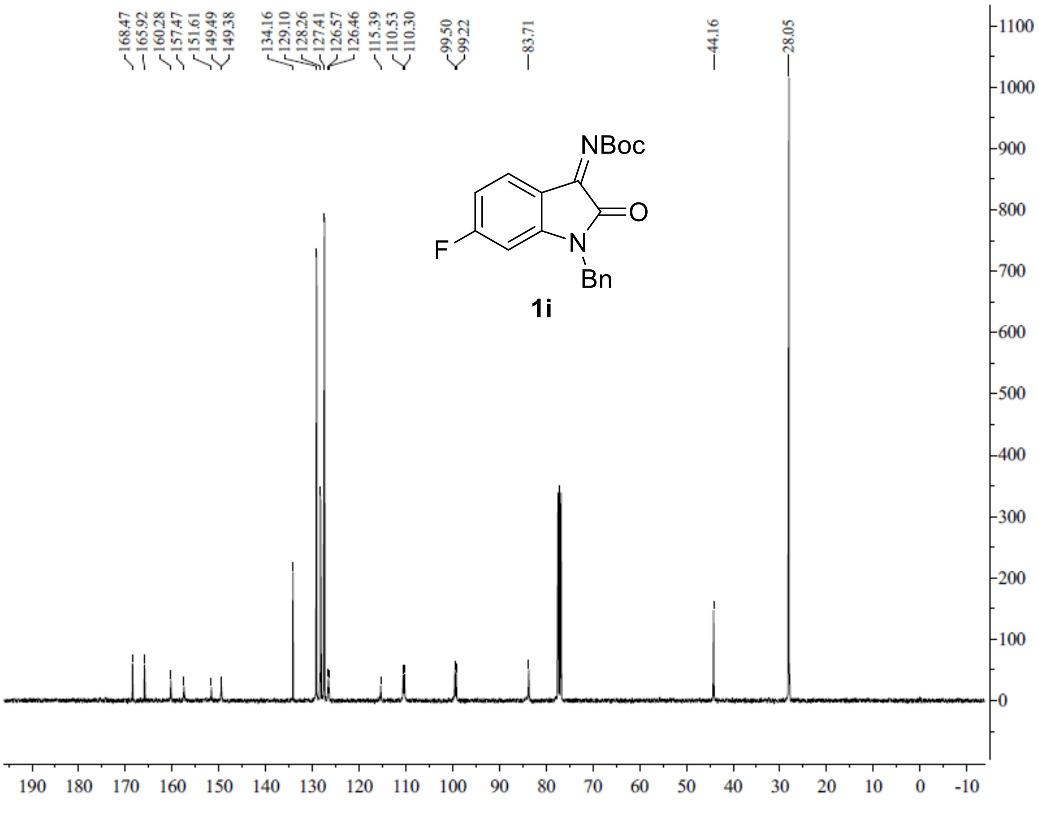

Figure S4. ^{13}C NMR spectrum of **1i**, related to **Figure 1.**

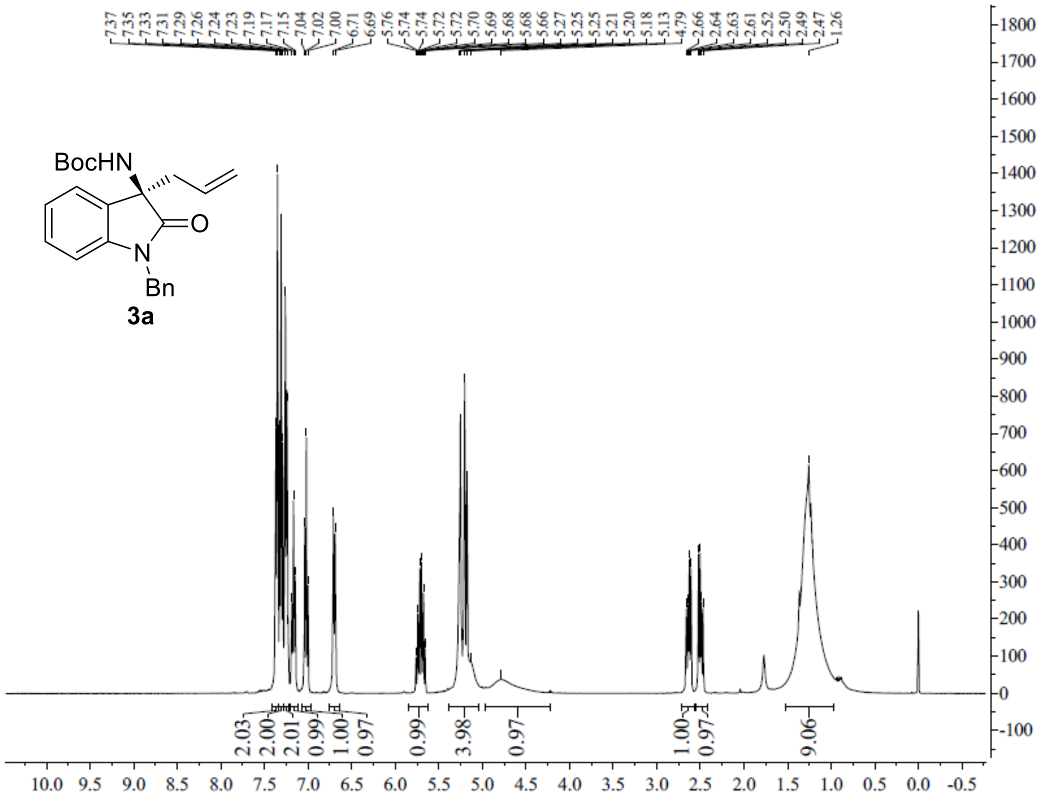

Figure S5. ^{1}H NMR spectrum of **3a**, related to **Figure 1**.

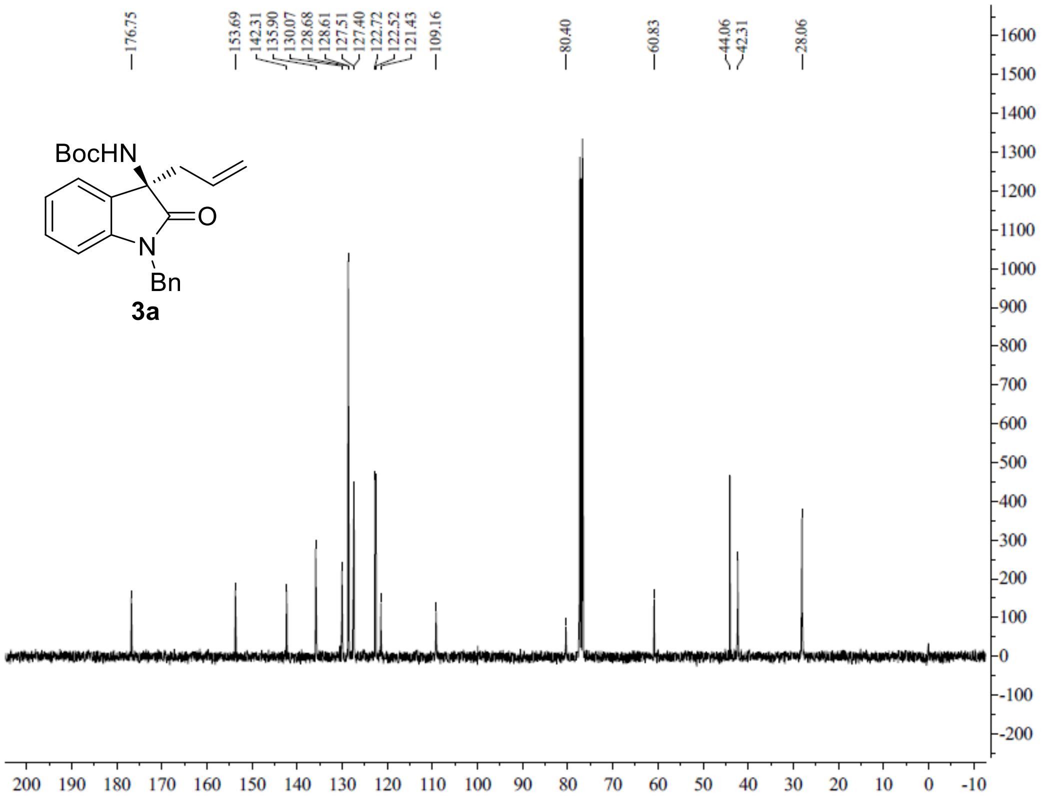

Figure S6. ^{13}C NMR spectrum of **3a**, related to **Figure 1.**

<Chromatogram>

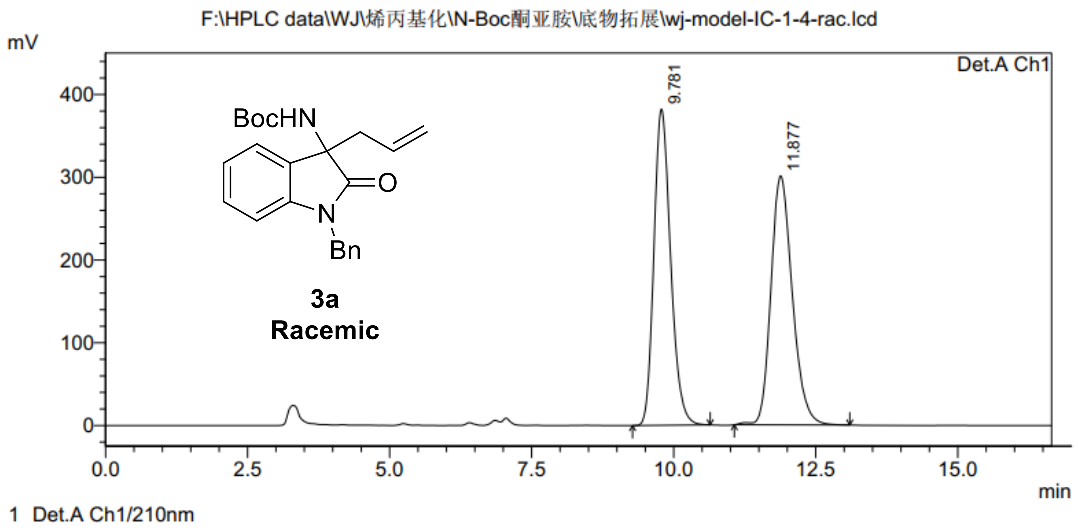

PeakTable

Detector A Ch1 210nm

Peak#	Ret. Time	Area	Height	Area %	Height %
1	9.781	7831520	382177	49.829	55.942
2	11.877	7885251	300990	50.171	44.058
Total		15716771	683166	100.000	100.000

<Chromatogram>

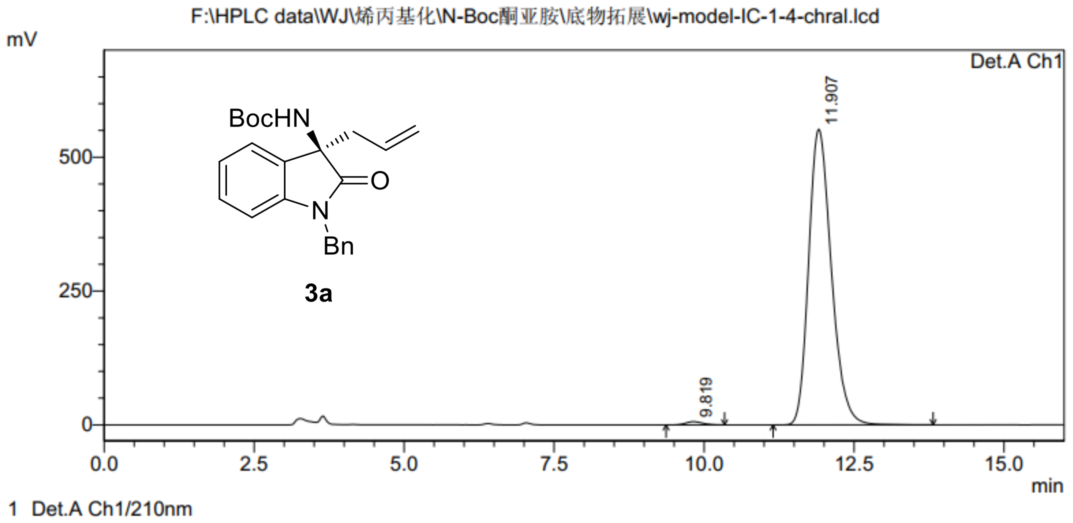

PeakTable

Detector A Ch1 210nm

Peak#	Ret. Time	Area	Height	Area %	Height %
1	9.819	119600	6031	0.816	1.080
2	11.907	14538536	552328	99.184	98.920
Total		14658136	558359	100.000	100.000

Figure S7. HPLC spectrum of **3a**, related to **Figure 1.**

<Chromatogram>

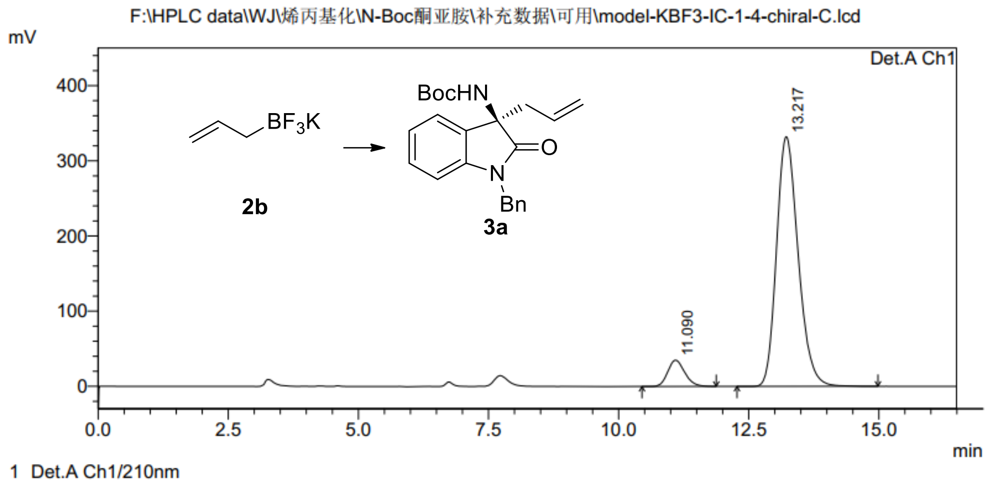

PeakTable

Detector A Ch1 210nm

Peak#	Ret. Time	Area	Height	Area %	Height %
1	11.090	793018	34977	7.641	9.527
2	13.217	9585727	332141	92.359	90.473
Total		10378746	367118	100.000	100.000

Figure S8. HPLC spectrum of **3a**, related to **Scheme 2.**

<Chromatogram>

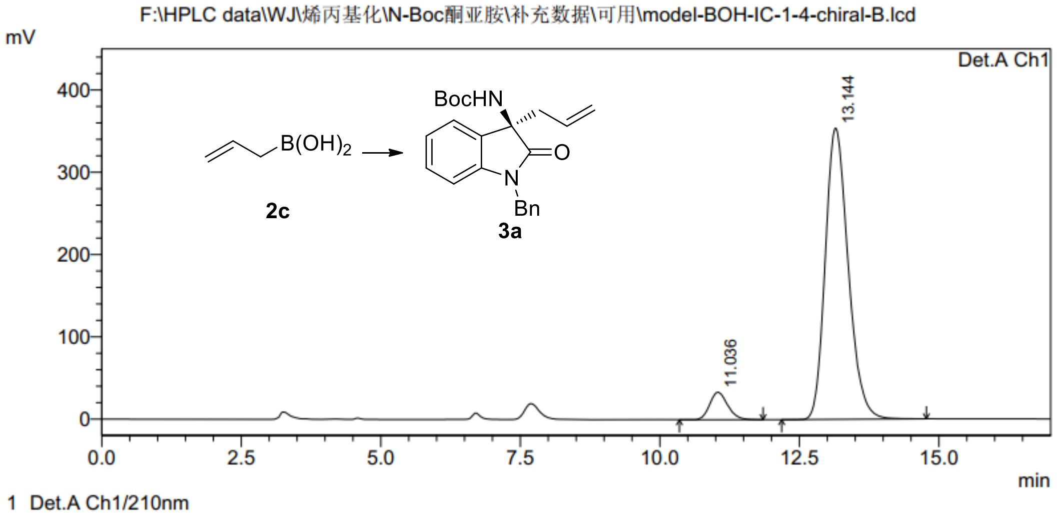

PeakTable

Detector A Ch1 210nm

Peak#	Ret. Time	Area	Height	Area %	Height %
1	11.036	750656	33476	6.912	8.638
2	13.144	10109786	354061	93.088	91.362
Total		10860441	387537	100.000	100.000

Figure S9. HPLC spectrum of **3a**, related to **Scheme 2.**

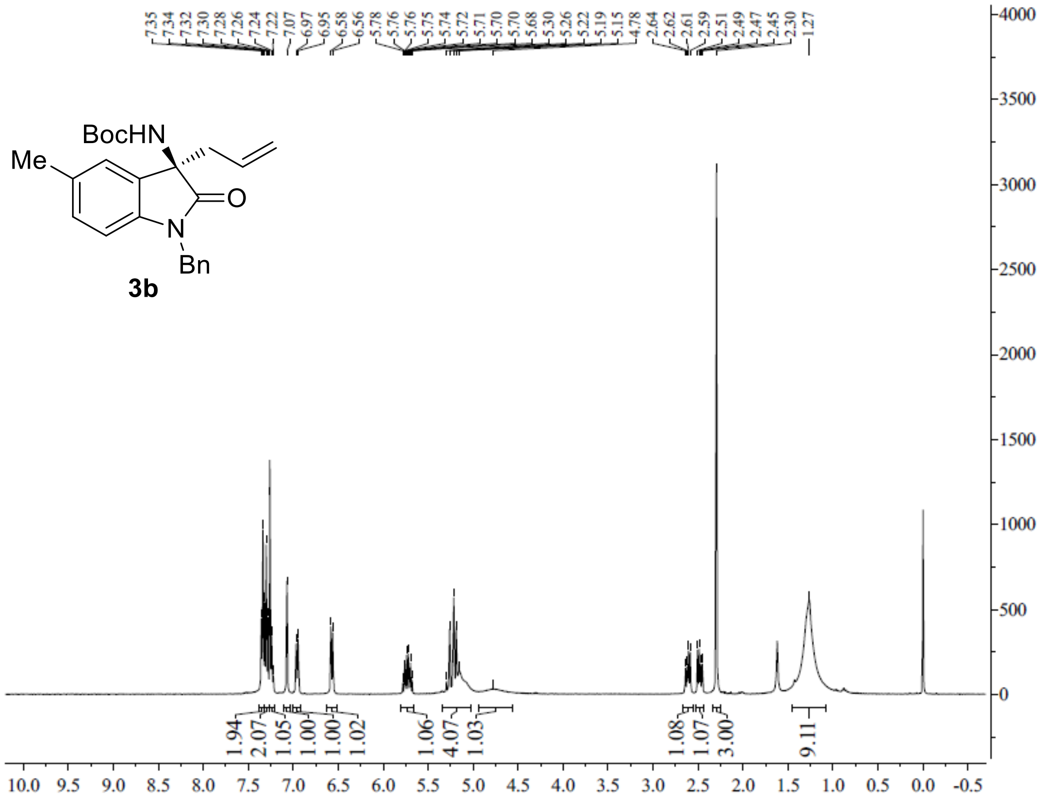

Figure S10. ^{1}H NMR spectrum of **3b**, related to **Figure 1**.

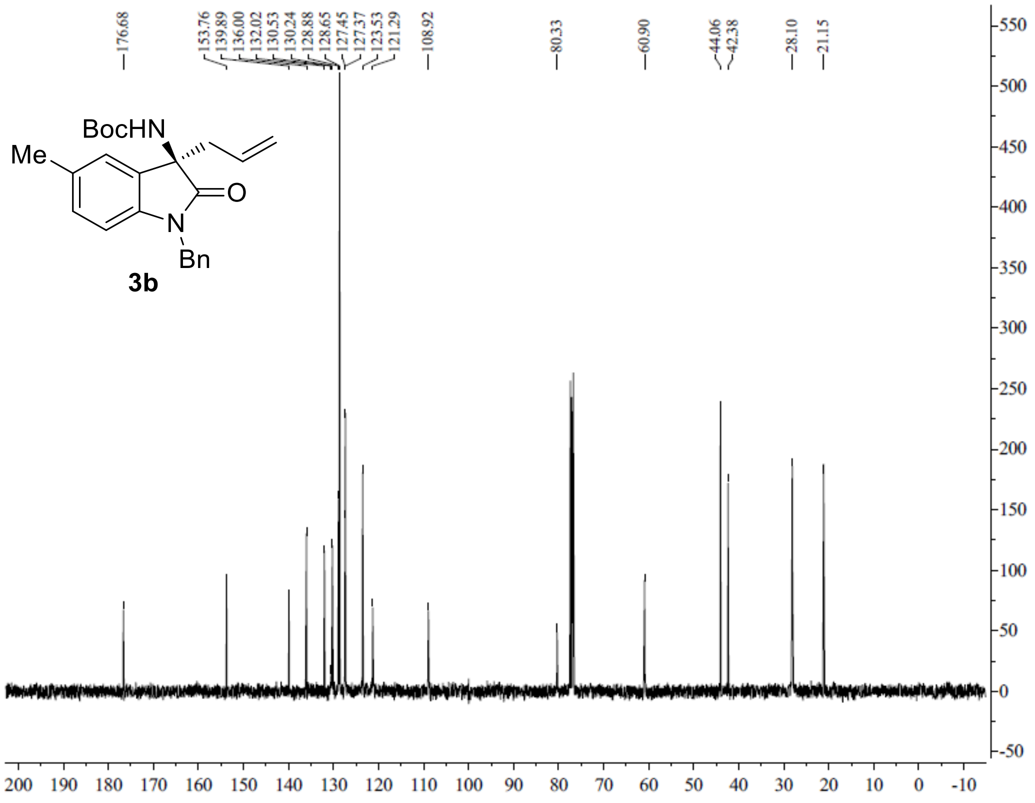

Figure S11. ^{13}C NMR spectrum of **3b**, related to **Figure 1.**

<Chromatogram>

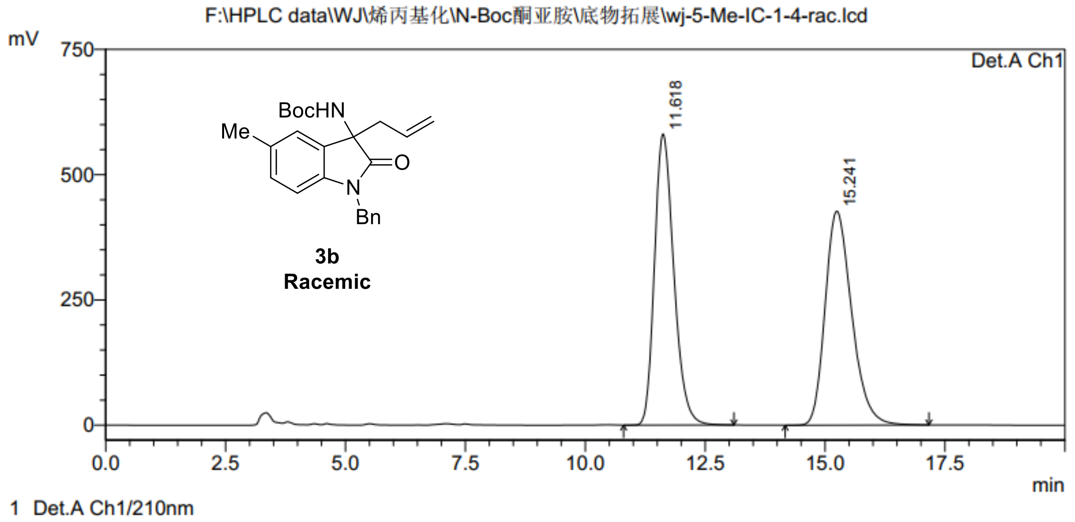

PeakTable

Detector A Ch1 210nm

Peak#	Ret. Time	Area	Height	Area %	Height %
1	11.618	16282441	581059	49.728	57.647
2	15.241	16460497	426907	50.272	42.353
Total		32742938	1007966	100.000	100.000

<Chromatogram>

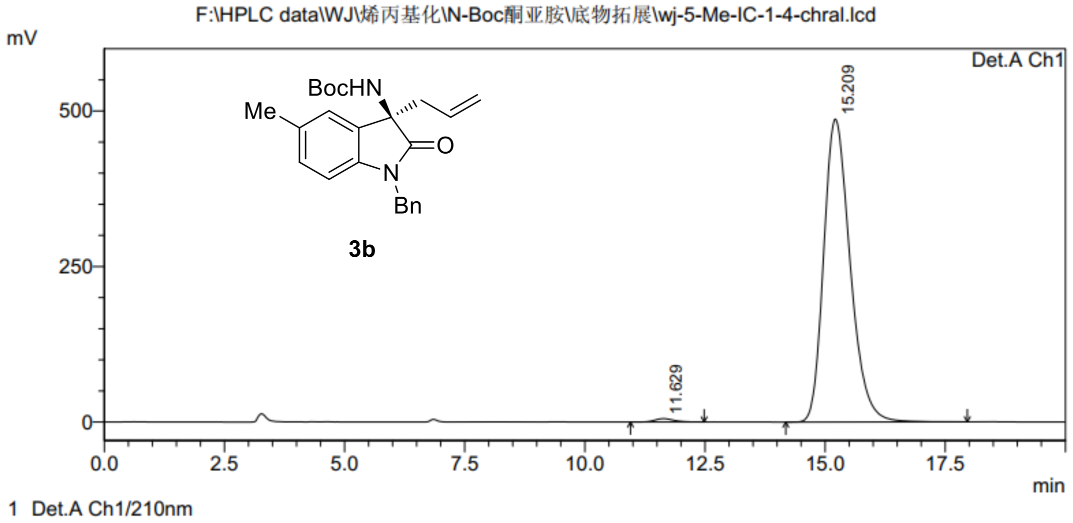

PeakTable

Detector A Ch1 210nm

Peak#	Ret. Time	Area	Height	Area %	Height %
1	11.629	146686	5430	0.781	1.103
2	15.209	18644249	486870	99.219	98.897
Total		18790934	492300	100.000	100.000

Figure S12. HPLC spectrum of **3b**, related to **Figure 1.**

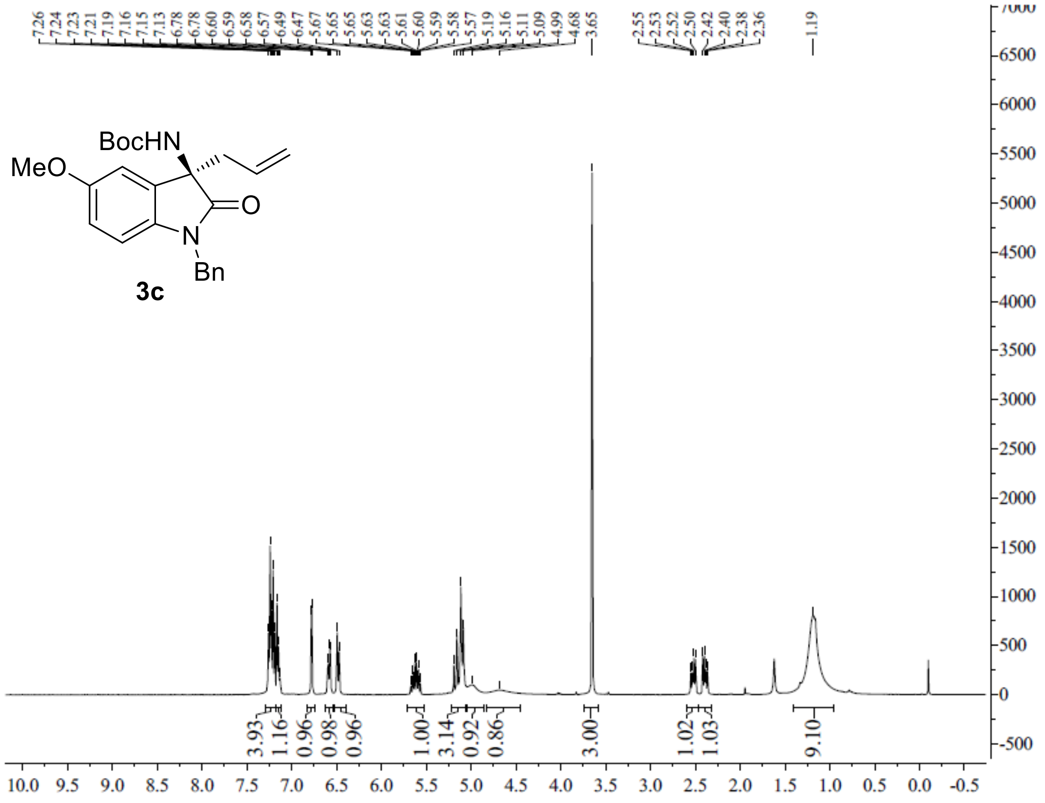

Figure S13. ^{1}H NMR spectrum of **3c**, related to **Figure 1**.

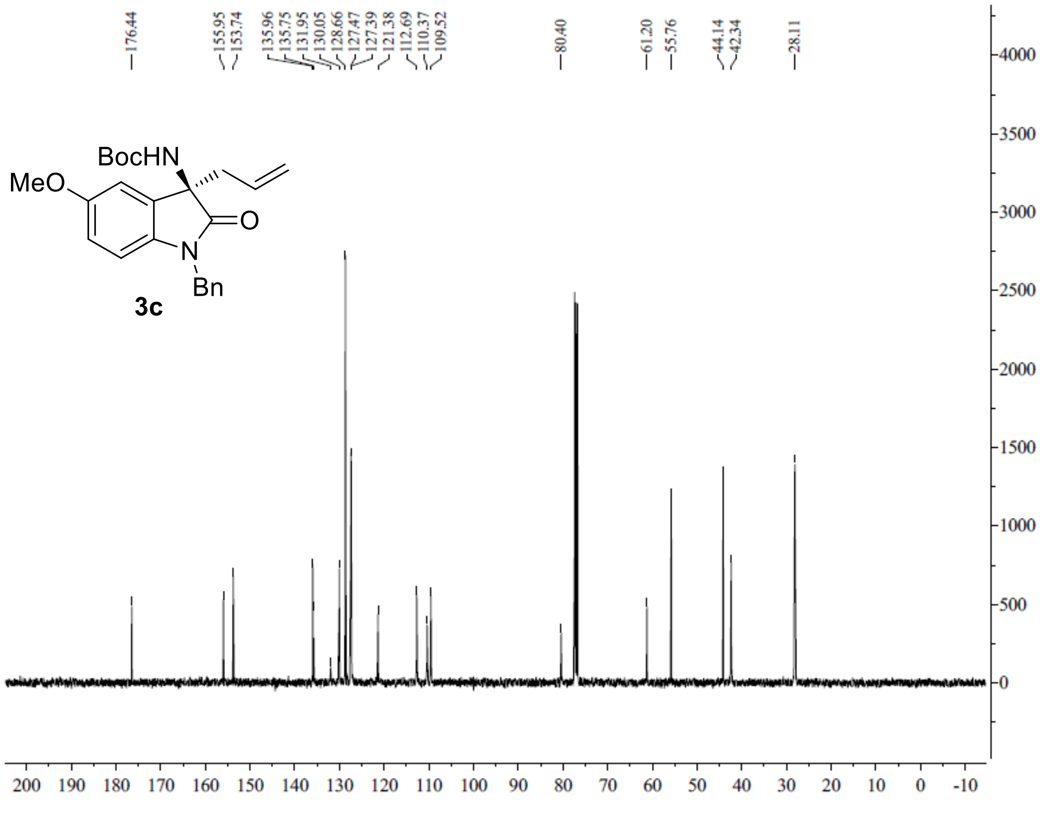

Figure S14. ^{13}C NMR spectrum of **3c**, related to **Figure 1.**

<Chromatogram>

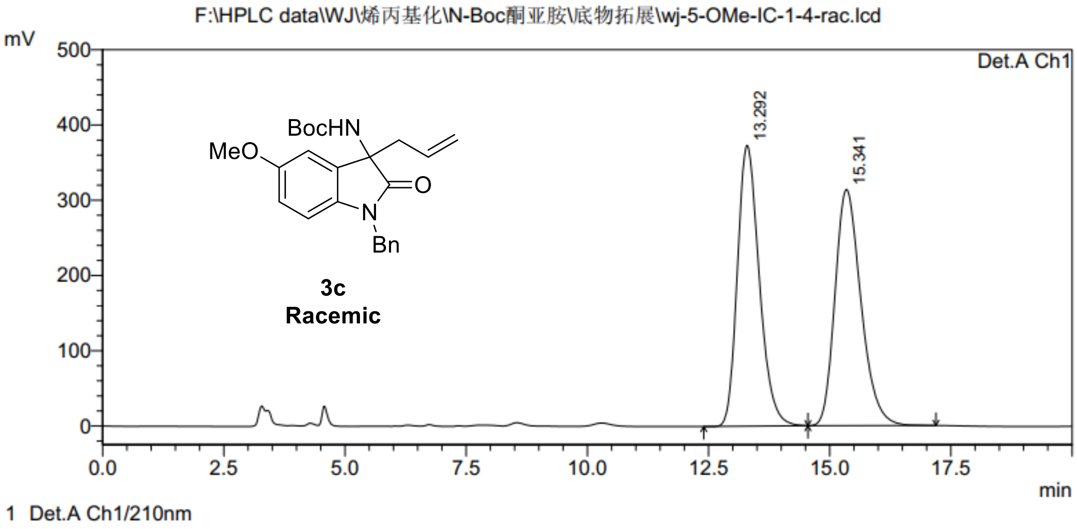

PeakTable

Detector A Ch1 210nm

Peak#	Ret. Time	Area	Height	Area %	Height %
1	13.292	11716455	373135	50.059	54.318
2	15.341	11688663	313816	49.941	45.682
Total		23405118	686951	100.000	100.000

<Chromatogram>

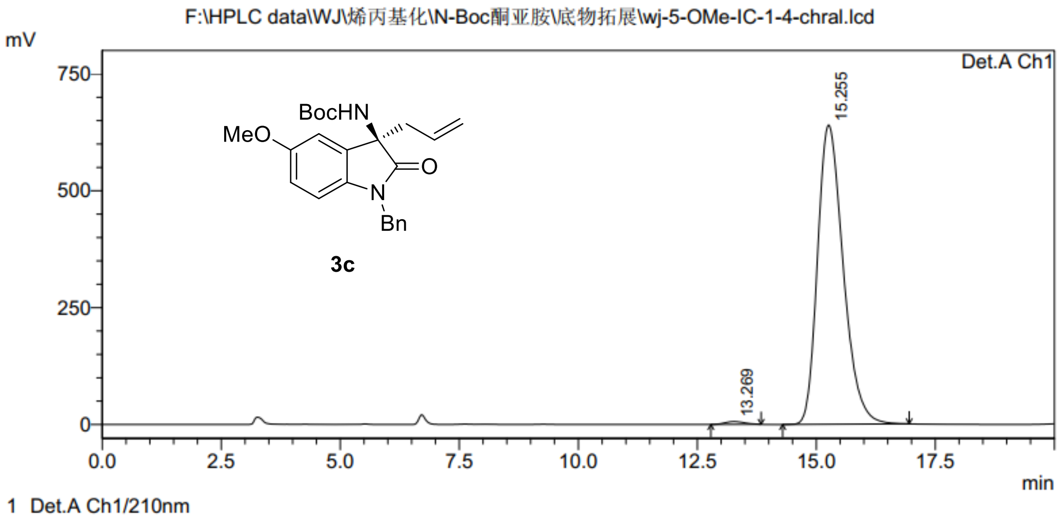

PeakTable

Detector A Ch1 210nm

Peak#	Ret. Time	Area	Height	Area %	Height %
1	13.269	173506	6057	0.718	0.937
2	15.255	23984030	640040	99.282	99.063
Total		24157535	646096	100.000	100.000

Figure S15. HPLC spectrum of **3c**, related to **Figure 1.**

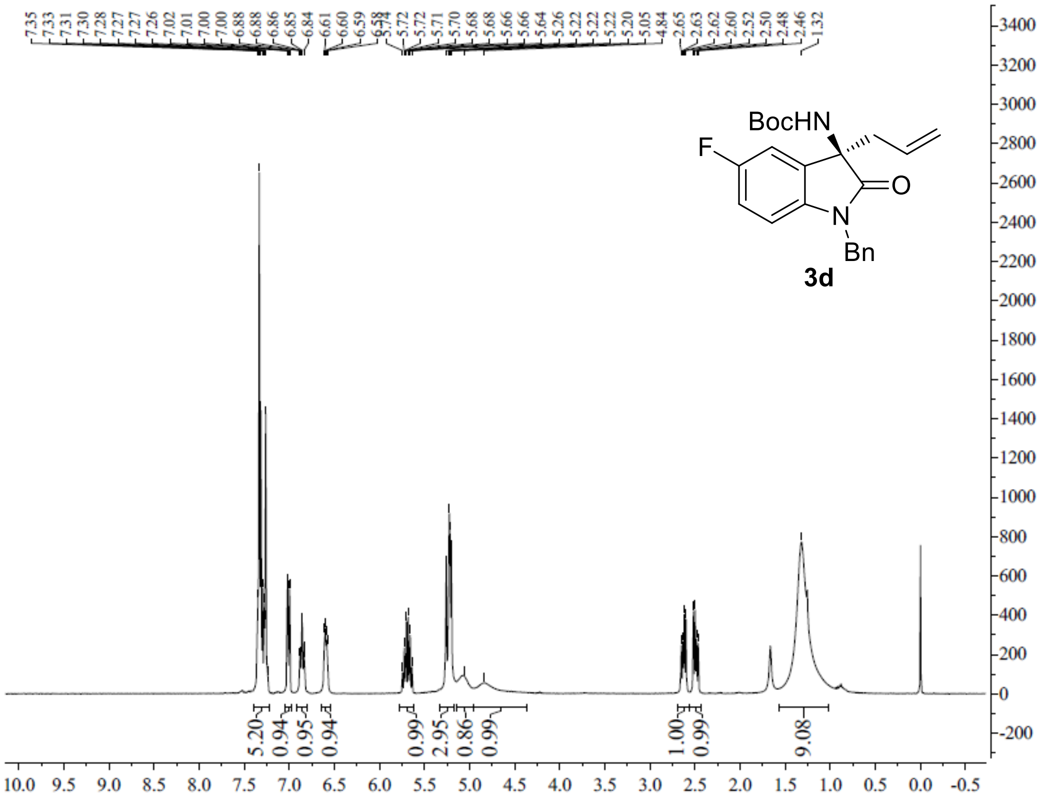

Figure S16. ^{1}H NMR spectrum of **3d**, related to **Figure 1**.

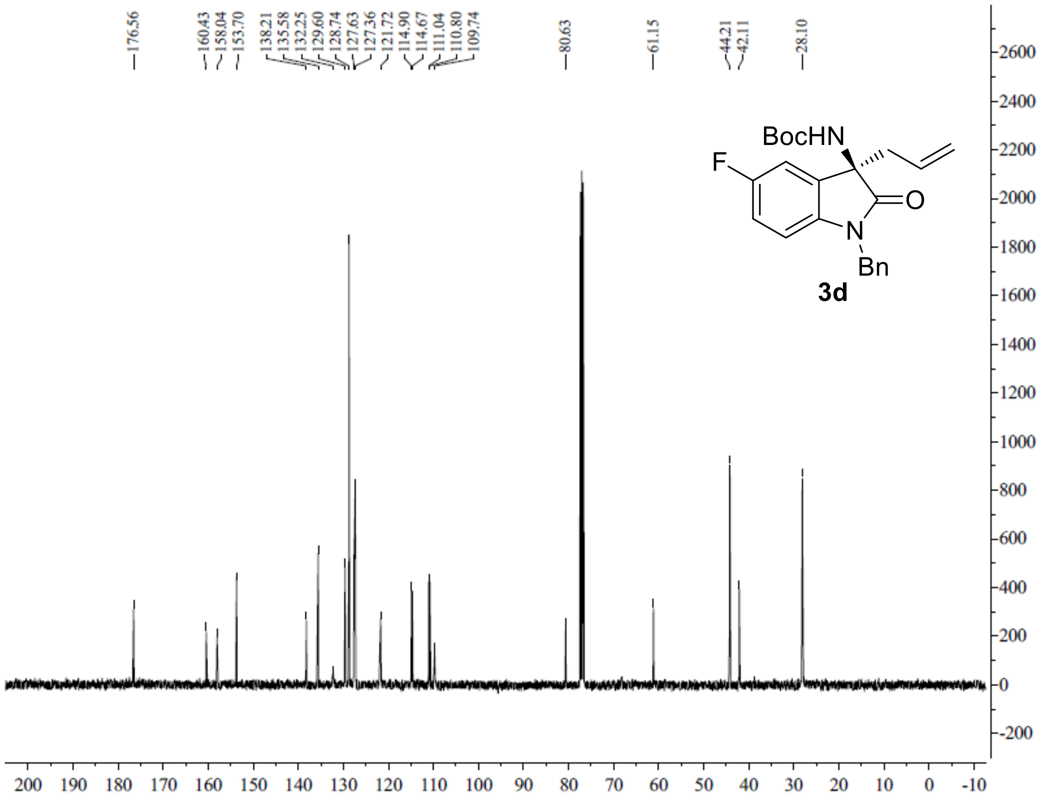

Figure S17. ^{13}C NMR spectrum of **3d**, related to **Figure 1.**

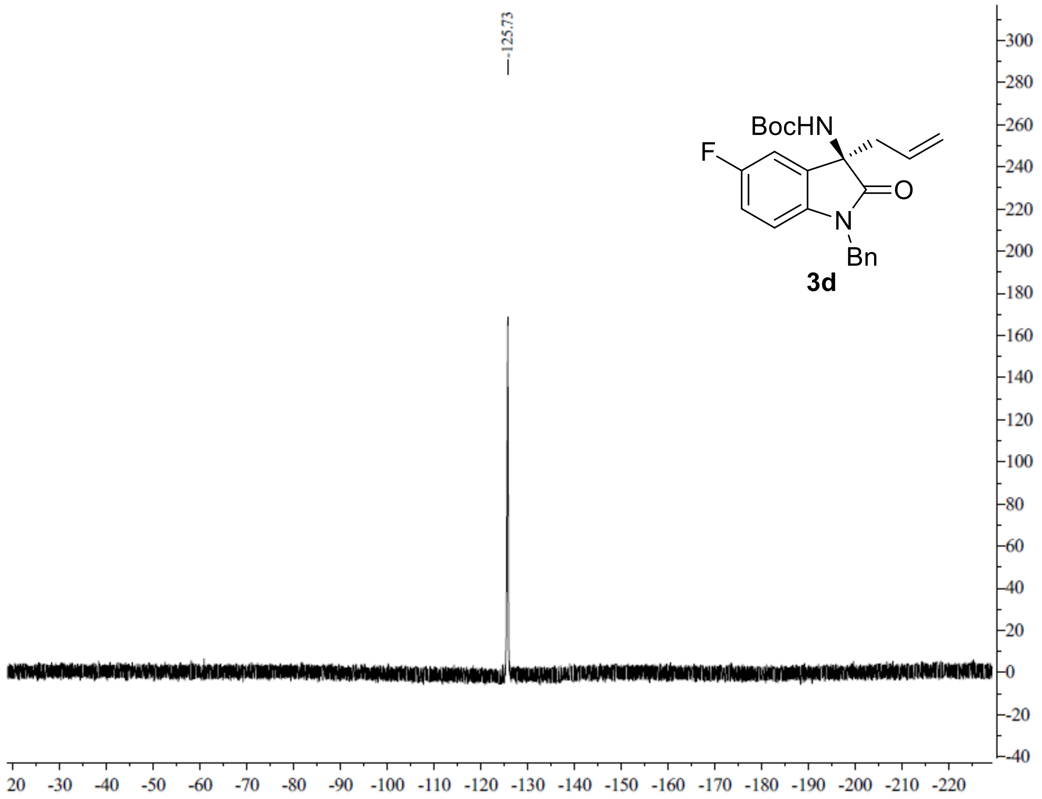

Figure S18. ^{19}F NMR spectrum of **3d**, related to **Figure 1.**

<Chromatogram>

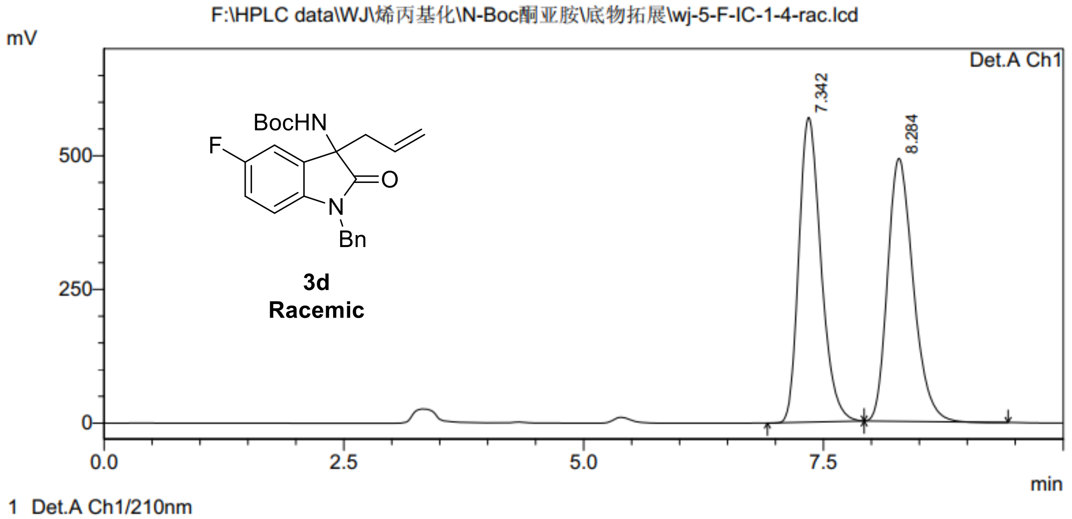

PeakTable

Detector A Ch1 210nm

Peak#	Ret. Time	Area	Height	Area %	Height %
1	7.342	9137475	569876	49.964	53.685
2	8.284	9150762	491647	50.036	46.315
Total		18288237	1061523	100.000	100.000

<Chromatogram>

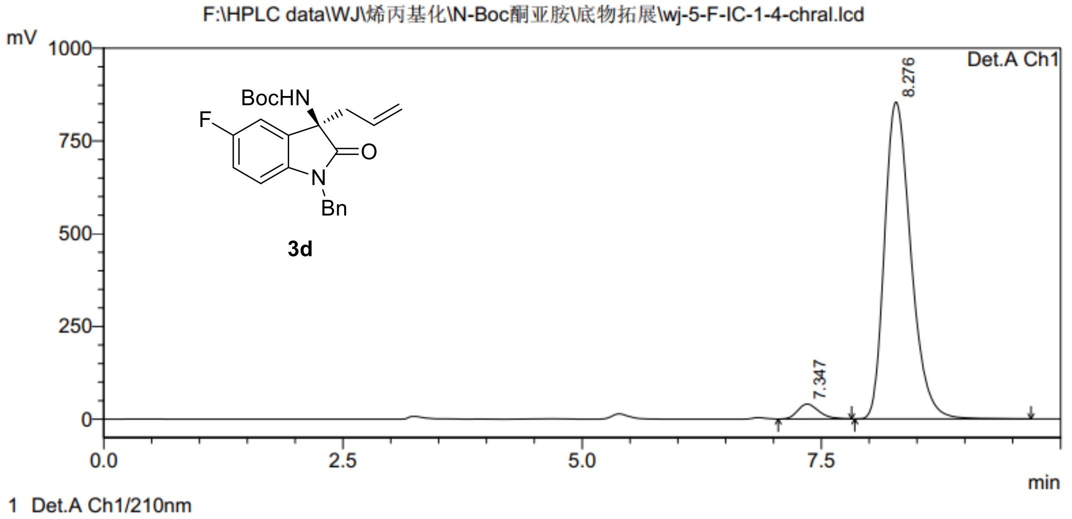

PeakTable

Detector A Ch1 210nm

Peak#	Ret. Time	Area	Height	Area %	Height %
1	7.347	617290	40215	3.633	4.495
2	8.276	16372288	854517	96.367	95.505
Total		16989577	894732	100.000	100.000

Figure S19. HPLC spectrum of **3d**, related to **Figure 1.**

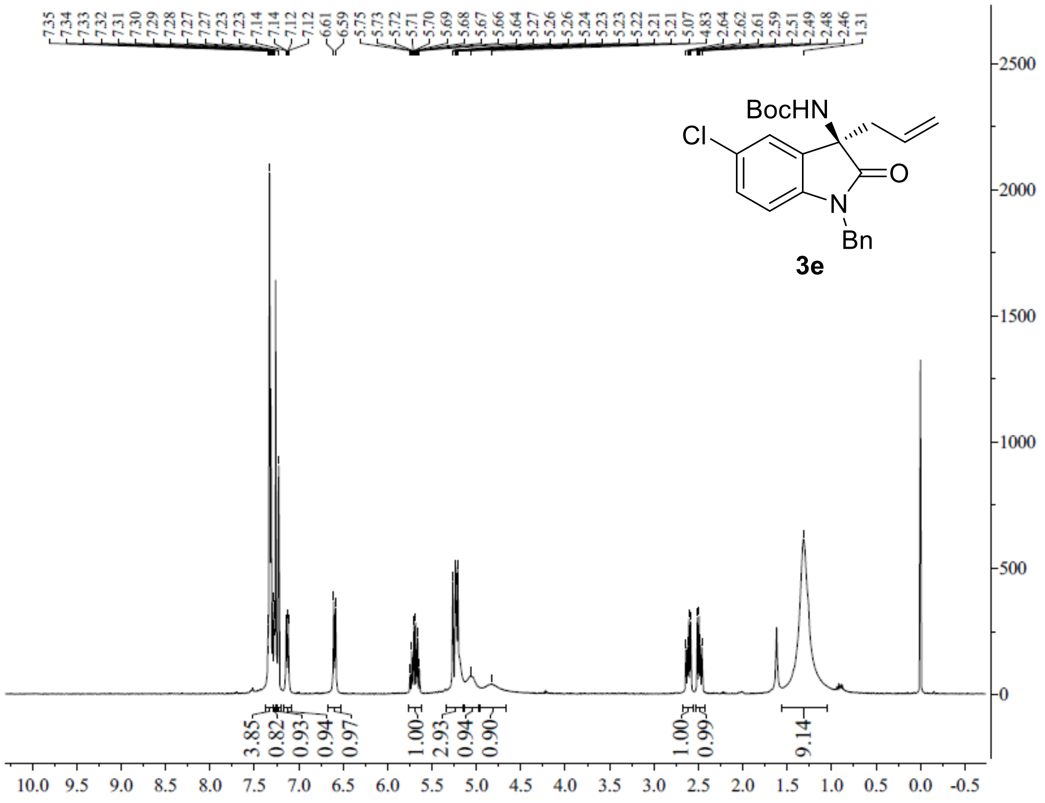

Figure S20. ^{1}H NMR spectrum of **3e**, related to **Figure 1**.

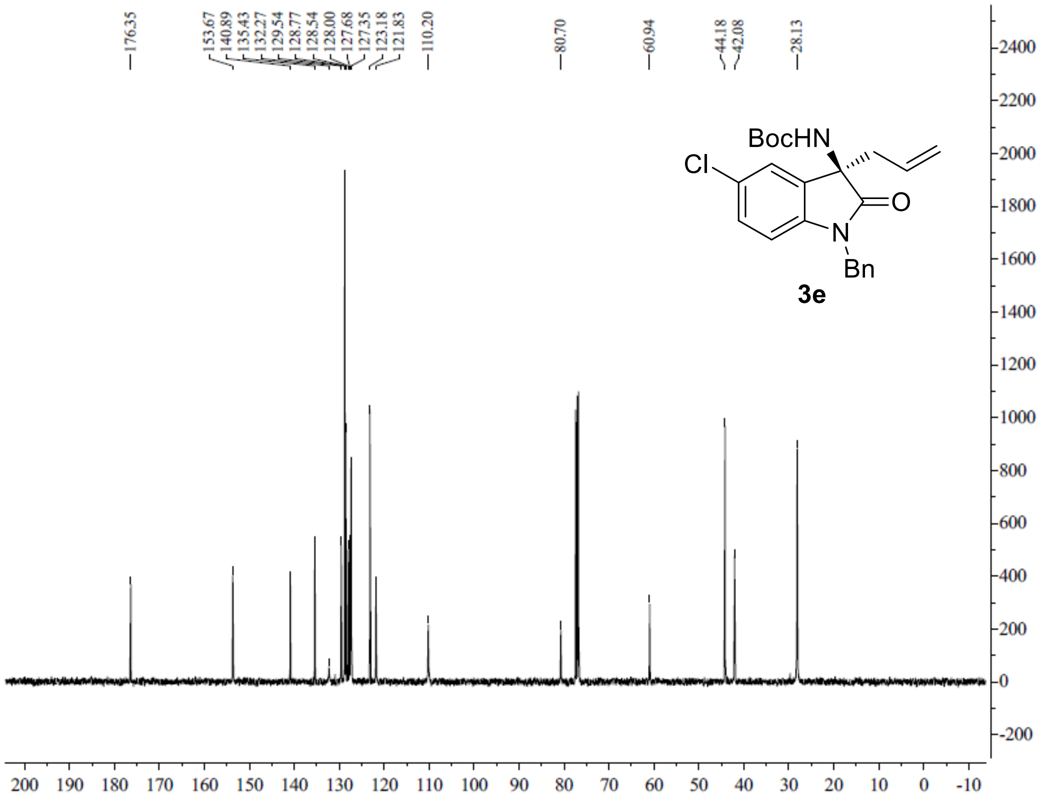

Figure S21. ^{13}C NMR spectrum of **3e**, related to **Figure 1.**

<Chromatogram>

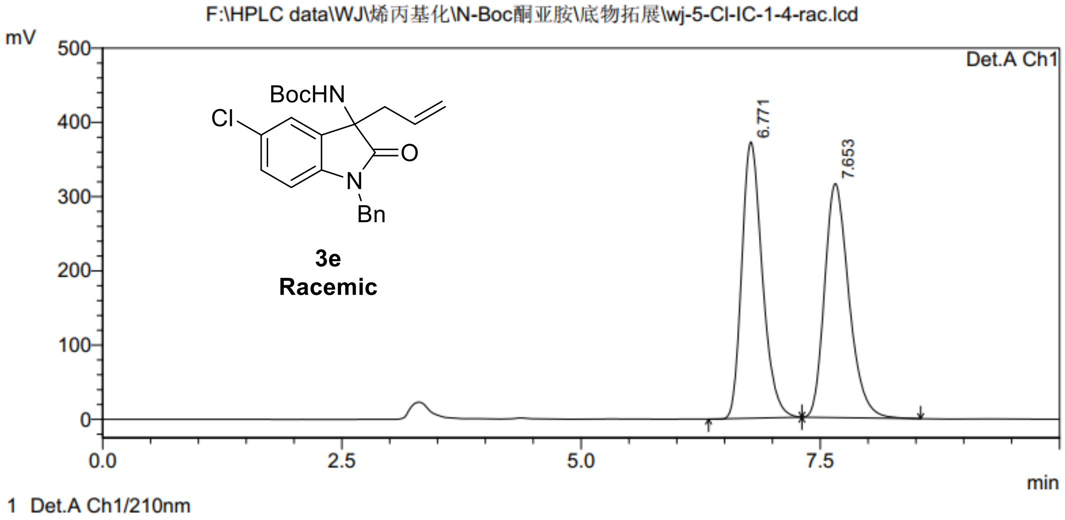

PeakTable

Detector A Ch1 210nm

Peak#	Ret. Time	Area	Height	Area %	Height %
1	6.771	5545149	372312	49.864	54.146
2	7.653	5575393	315292	50.136	45.854
Total		11120541	687604	100.000	100.000

<Chromatogram>

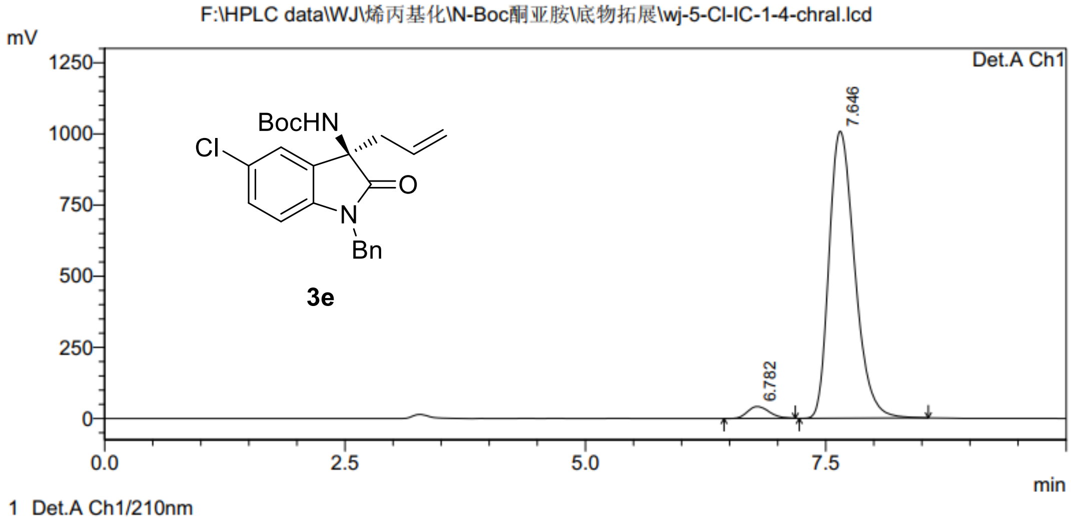

PeakTable

Detector A Ch1 210nm

Peak#	Ret. Time	Area	Height	Area %	Height %
1	6.782	655125	41787	3.381	3.978
2	7.646	18721443	1008681	96.619	96.022
Total		19376568	1050468	100.000	100.000

Figure S22. HPLC spectrum of **3e**, related to **Figure 1.**

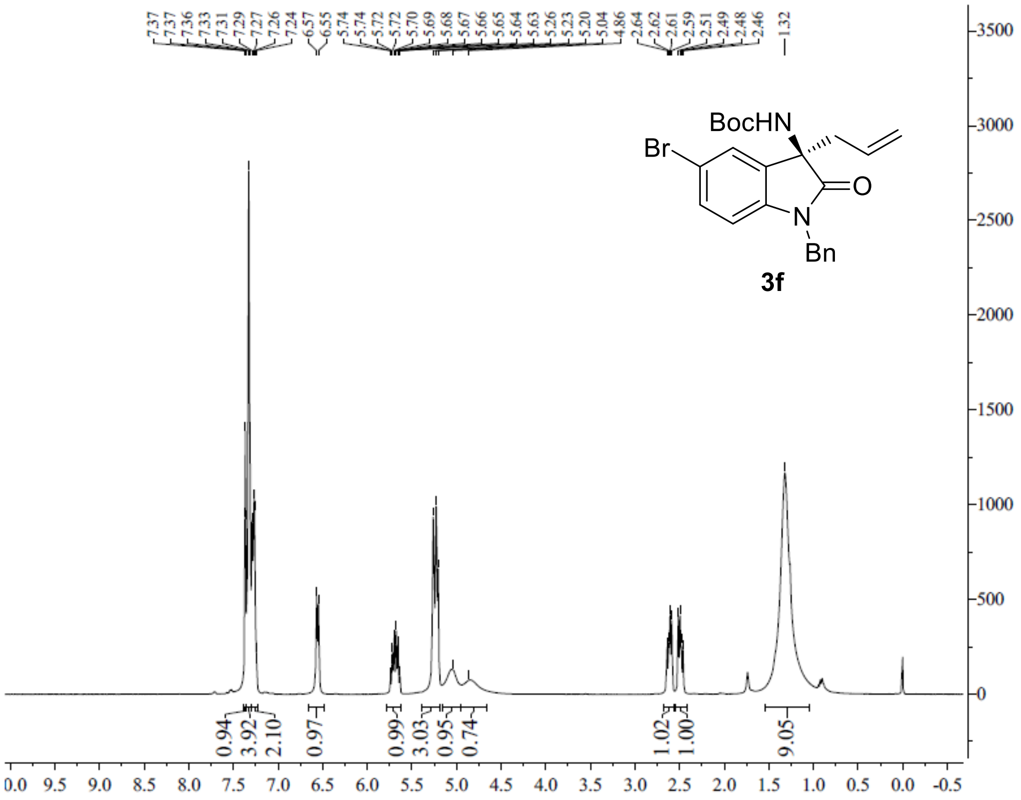

Figure S23. ^{1}H NMR spectrum of **3f**, related to **Figure 1**.

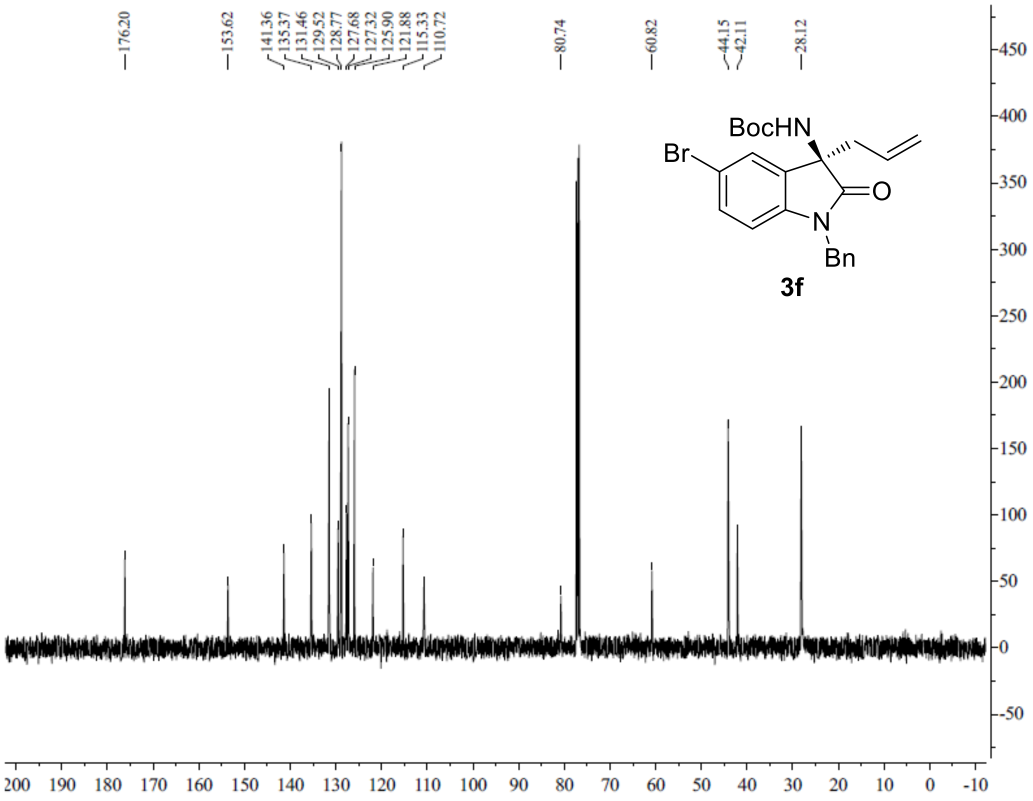

Figure S24. ^{13}C NMR spectrum of **3f**, related to **Figure 1.**

<Chromatogram>

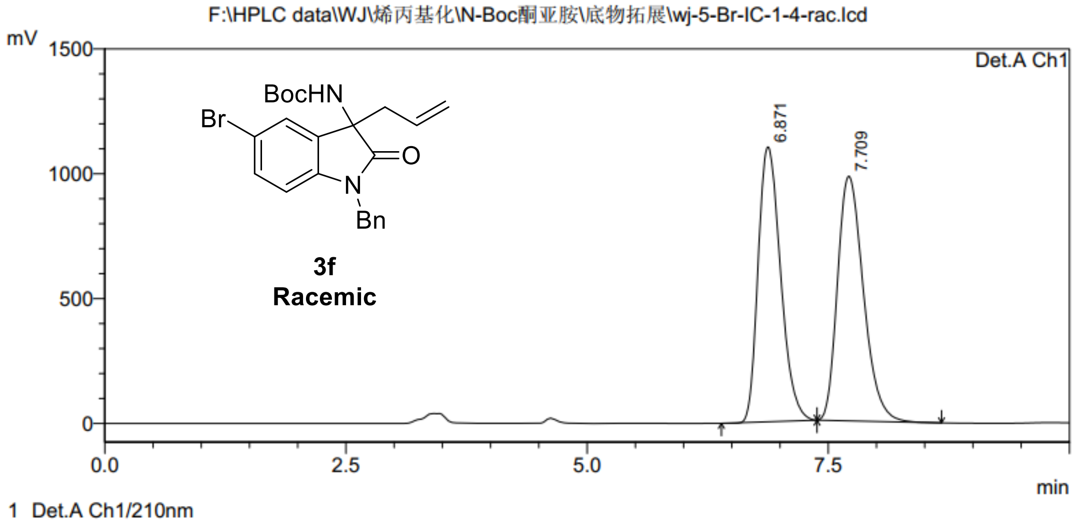

PeakTable

Detector A Ch1 210nm

Peak#	Ret. Time	Area	Height	Area %	Height %
1	6.871	17830706	1101053	49.348	52.910
2	7.709	18301868	979940	50.652	47.090
Total		36132573	2080993	100.000	100.000

<Chromatogram>

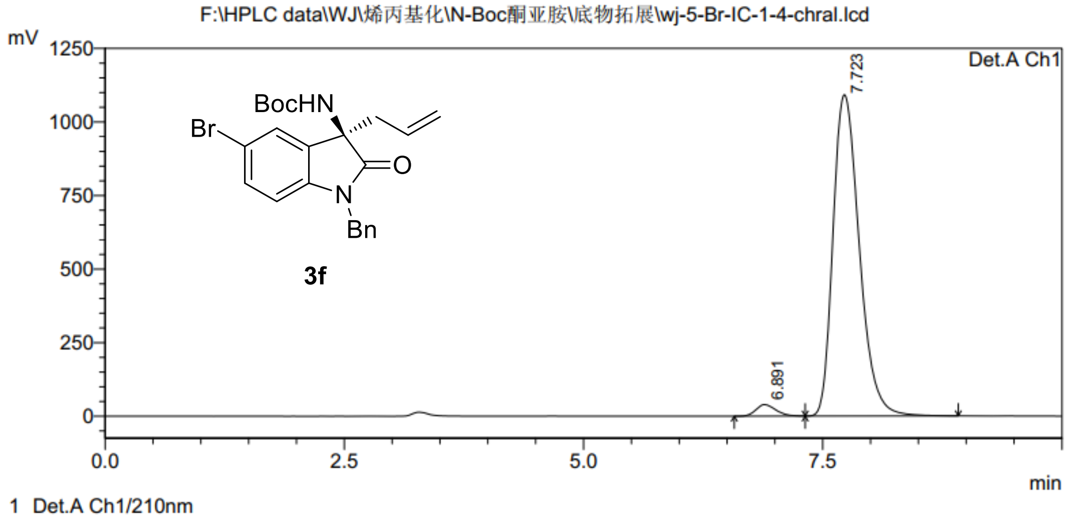

PeakTable

Detector A Ch1 210nm

Peak#	Ret. Time	Area	Height	Area %	Height %
1	6.891	566074	39649	2.640	3.503
2	7.723	20873260	1092125	97.360	96.497
Total		21439334	1131775	100.000	100.000

Figure S25. HPLC spectrum of **3f**, related to **Figure 1.**

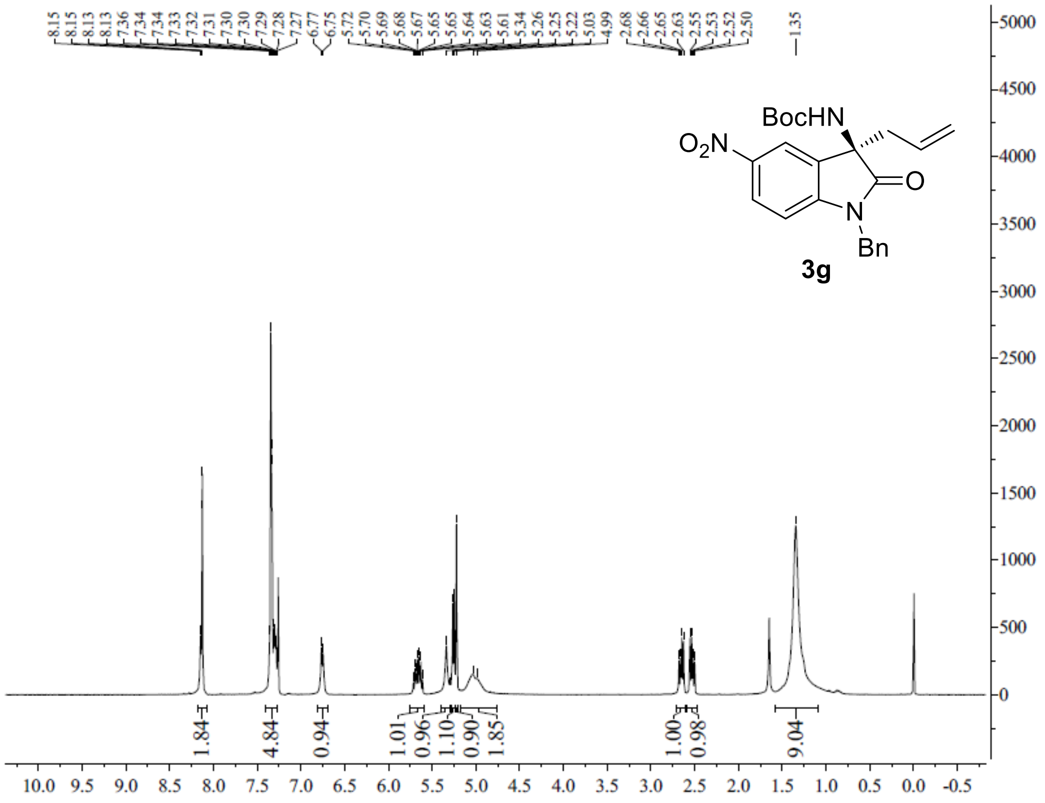

Figure S26. ^{1}H NMR spectrum of **3g**, related to **Figure 1**.

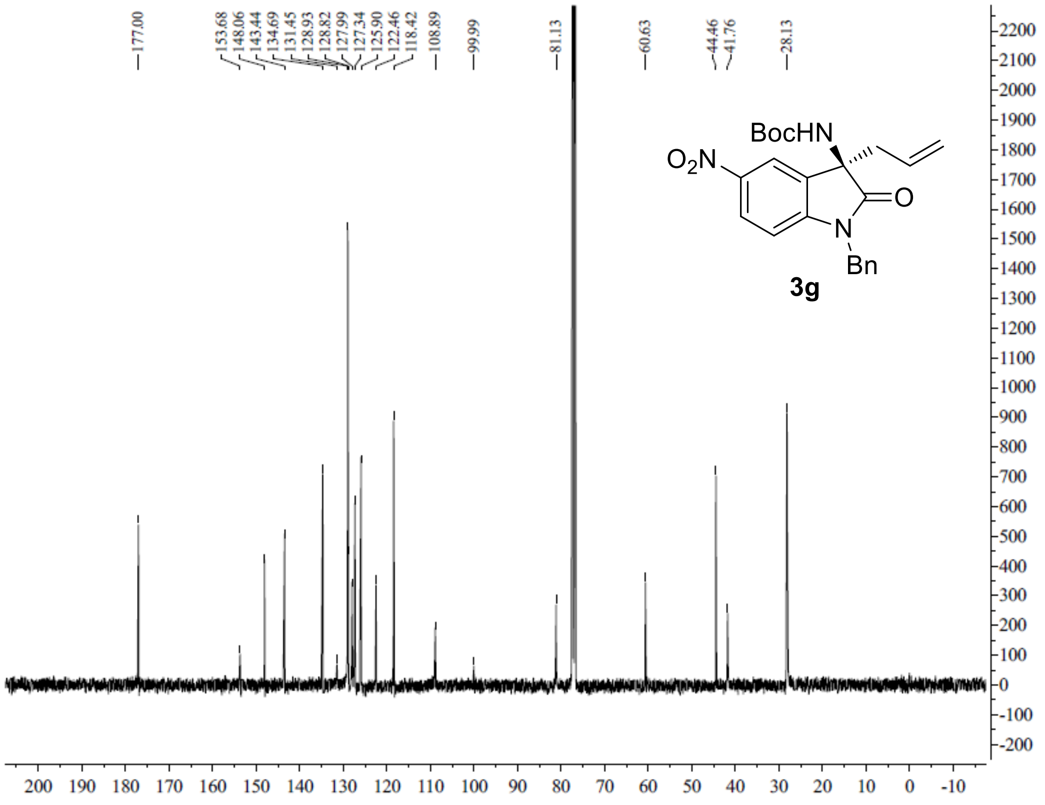

Figure S27. ^{13}C NMR spectrum of **3g**, related to **Figure 1.**

<Chromatogram>

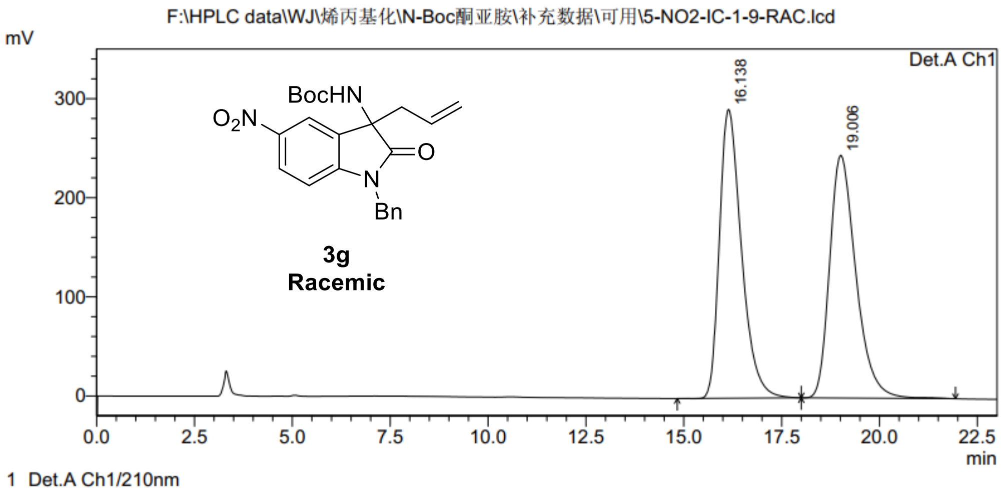

PeakTable

Detector A Ch1 210nm

Peak#	Ret. Time	Area	Height	Area %	Height %
1	16.138	11397732	291417	49.907	54.334
2	19.006	11440113	244925	50.093	45.666
Total		22837845	536341	100.000	100.000

<Chromatogram>

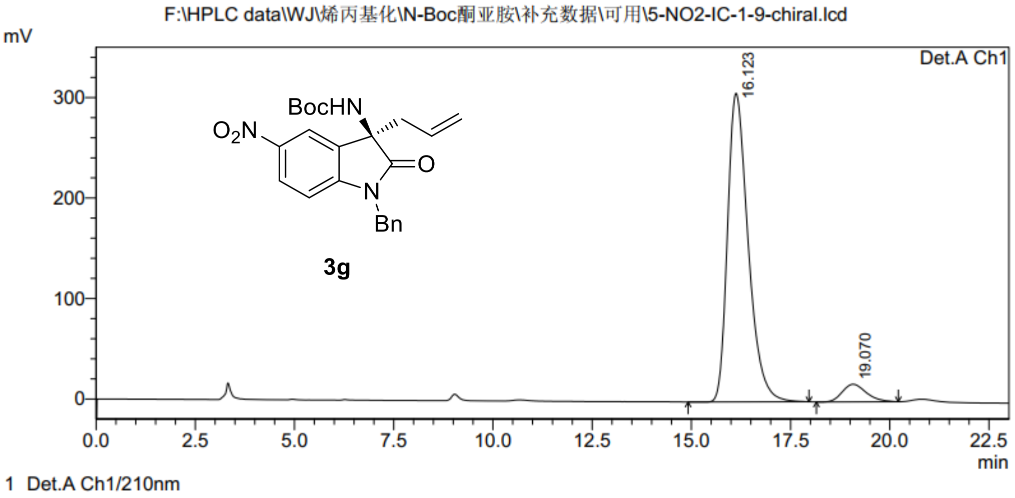

PeakTable

Detector A Ch1 210nm

Peak#	Ret. Time	Area	Height	Area %	Height %
1	16.123	11103910	307135	93.768	94.612
2	19.070	737944	17491	6.232	5.388
Total		11841853	324626	100.000	100.000

Figure S28. HPLC spectrum of **3g**, related to **Figure 1.**

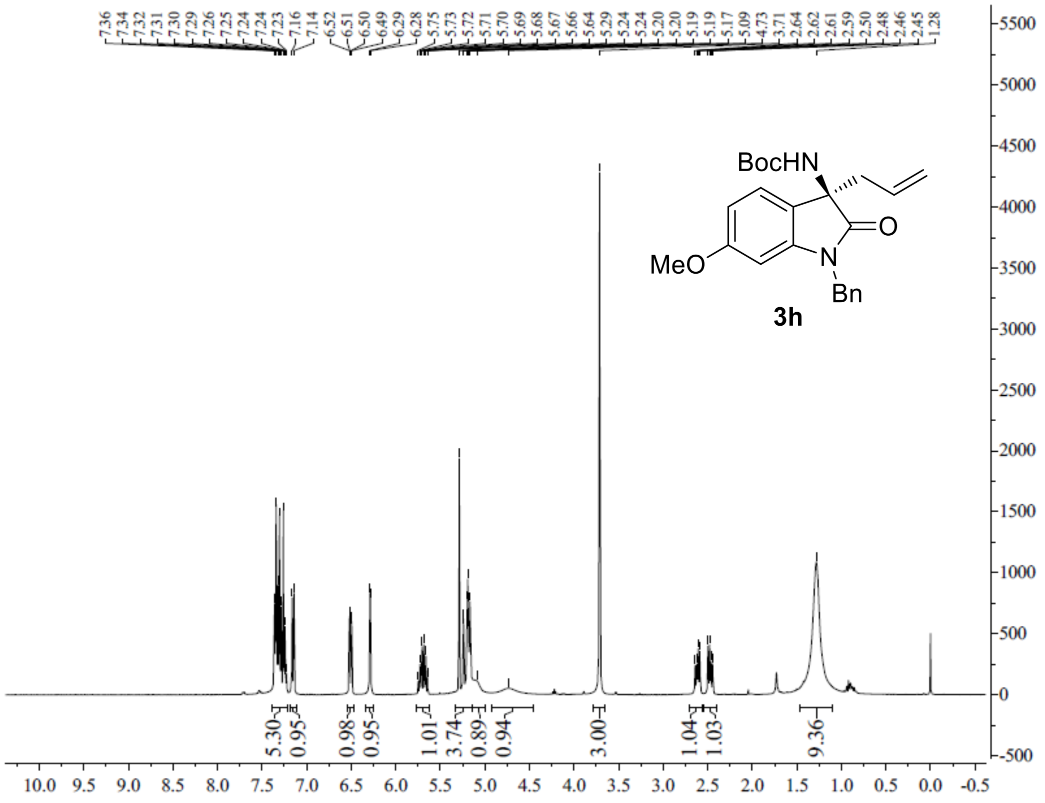

Figure S29. ^{1}H NMR spectrum of **3h**, related to **Figure 1**.

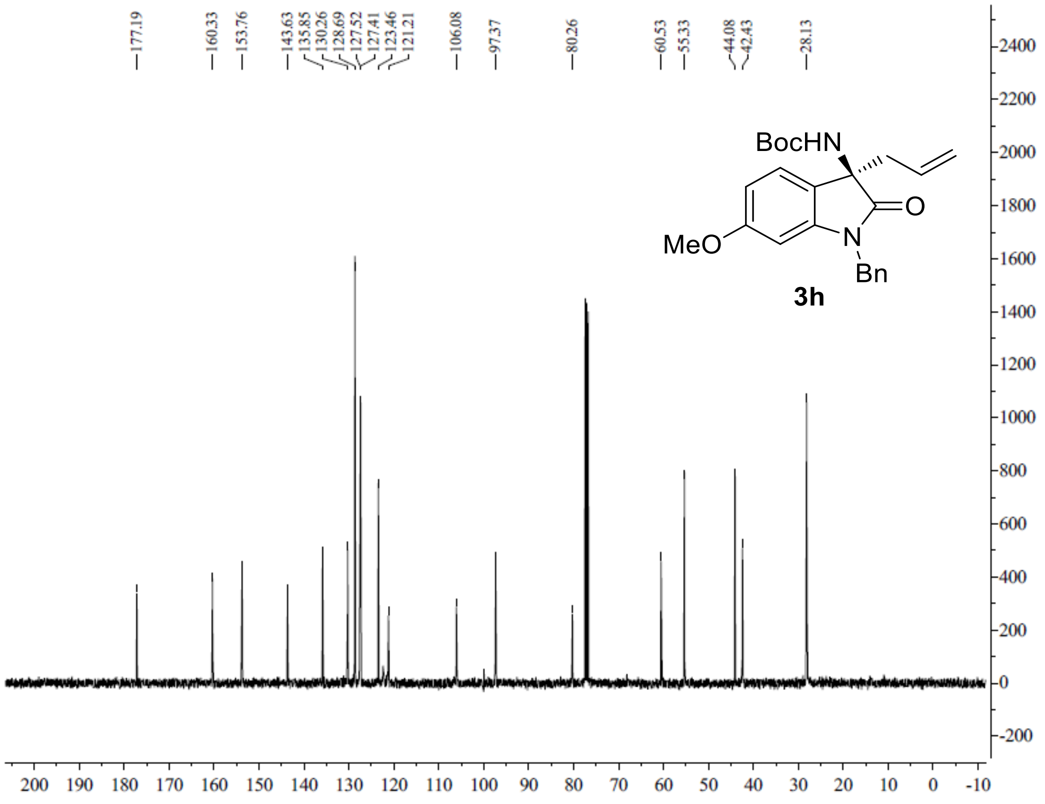

Figure S30. ^{13}C NMR spectrum of **3h**, related to **Figure 1.**

<Chromatogram>

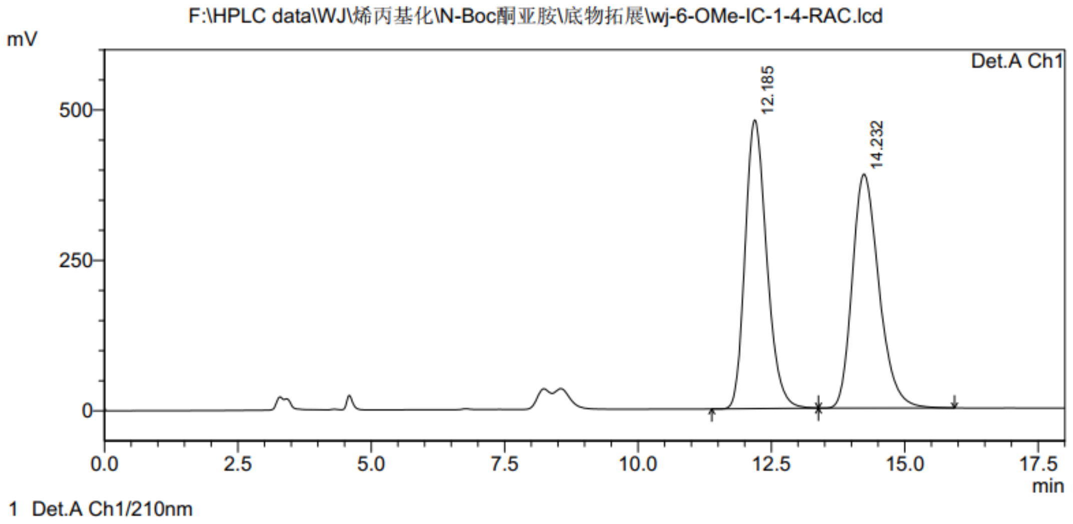

PeakTable

Detector A Ch1 210nm

Peak#	Ret. Time	Area	Height	Area %	Height %
1	12.185	13505010	479790	49.984	55.237
2	14.232	13513505	388806	50.016	44.763
Total		27018516	868595	100.000	100.000

<Chromatogram>

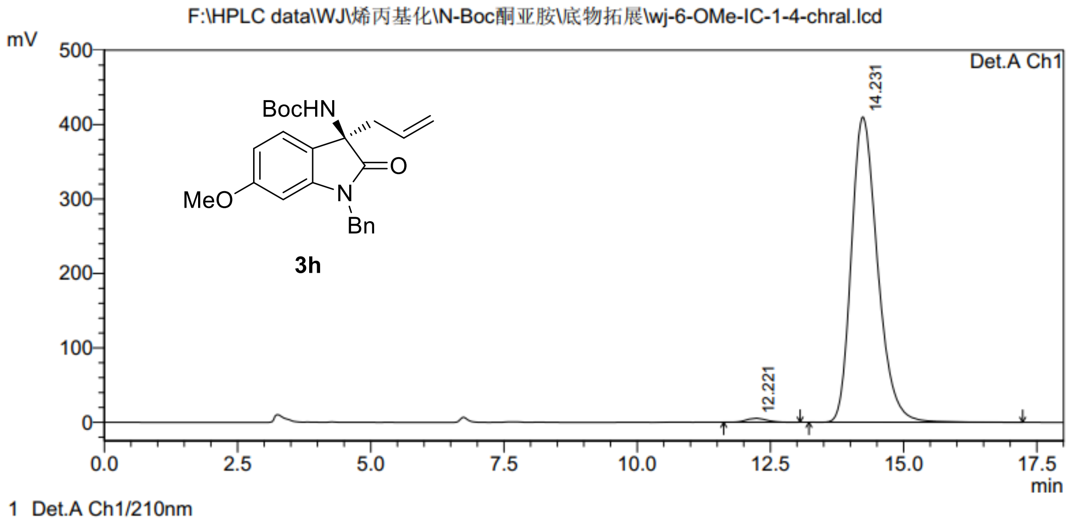

PeakTable

Detector A Ch1 210nm

Peak#	Ret. Time	Area	Height	Area %	Height %
1	12.221	135115	5318	0.953	1.280
2	14.231	14036166	410217	99.047	98.720
Total		14171282	415535	100.000	100.000

Figure S31. HPLC spectrum of **3h**, related to **Figure 1.**

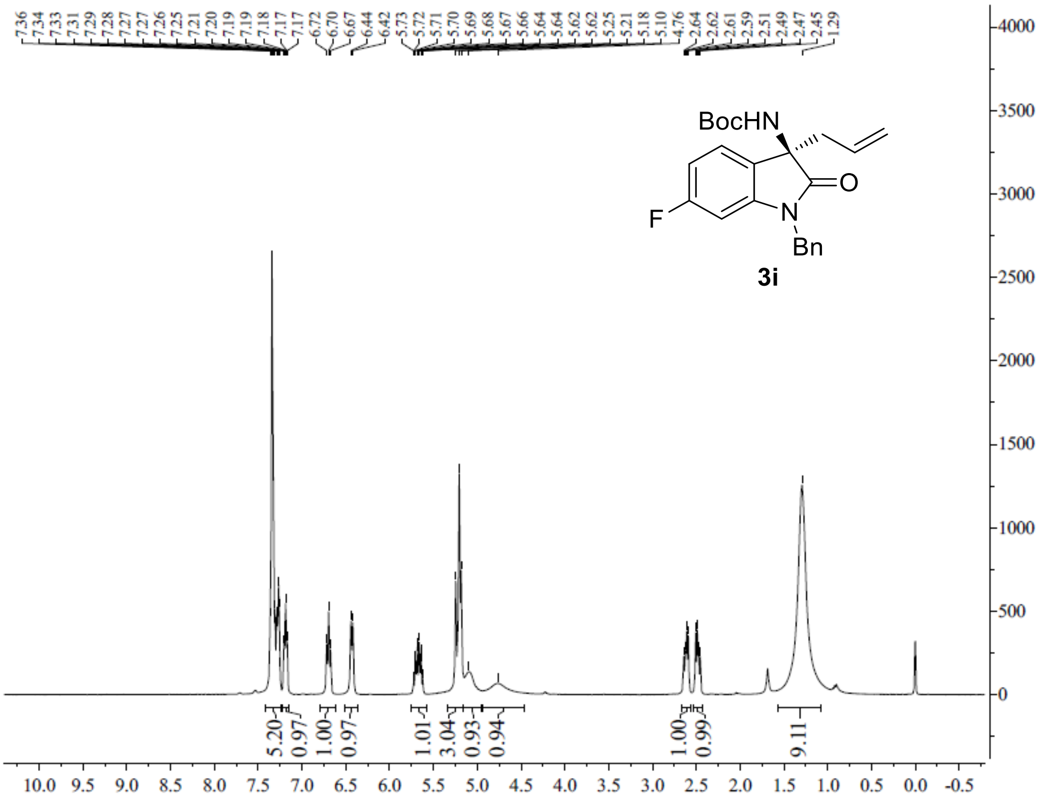

Figure S32. ^{1}H NMR spectrum of **3i**, related to **Figure 1**.

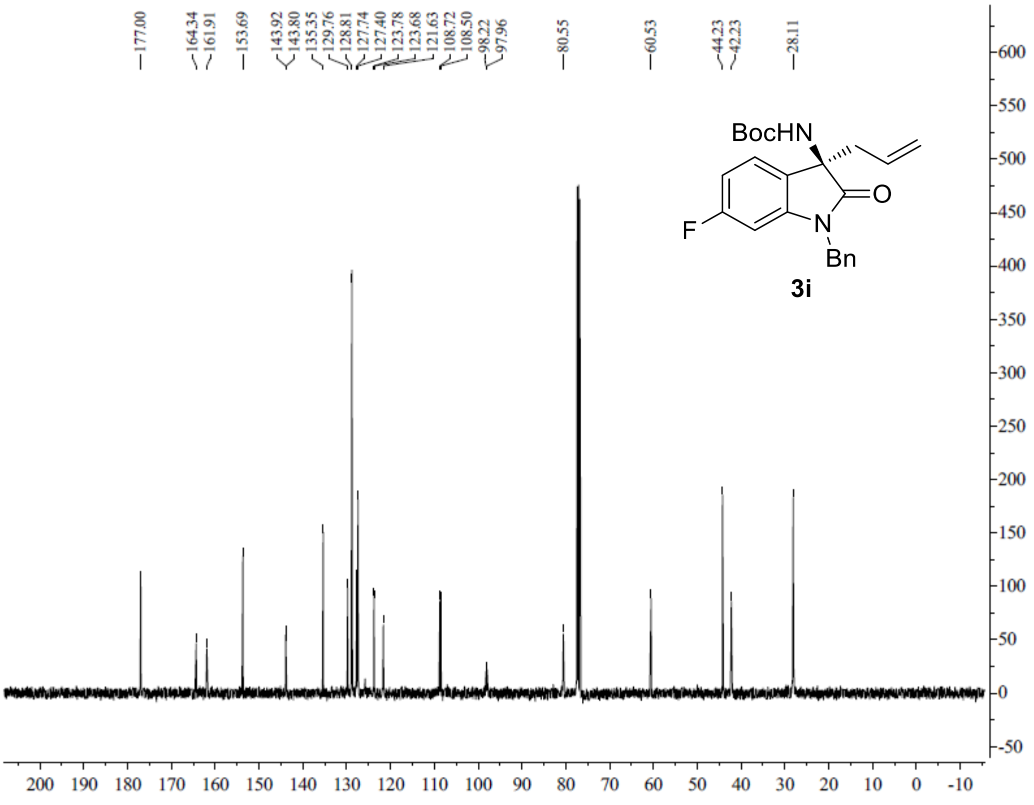

Figure S33. ^{13}C NMR spectrum of **3i**, related to **Figure 1.**

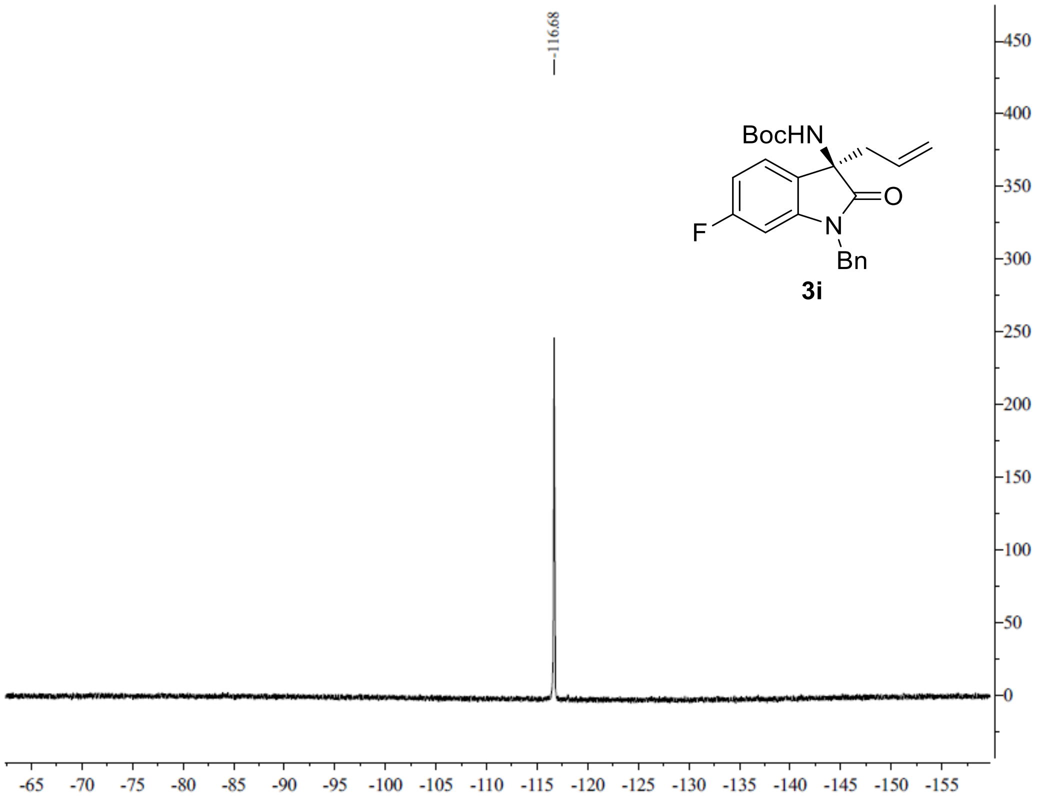

Figure S34. ^{19}F NMR spectrum of **3i**, related to **Figure 1.**

<Chromatogram>

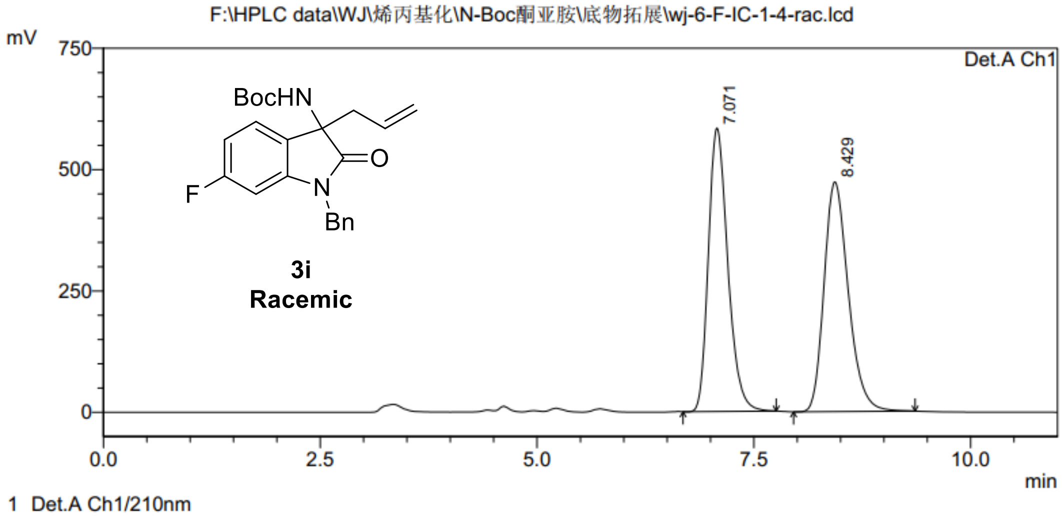

PeakTable

Detector A Ch1 210nm

Peak#	Ret. Time	Area	Height	Area %	Height %
1	7.071	9091672	583929	49.713	55.233
2	8.429	9196519	473286	50.287	44.767
Total		18288191	1057215	100.000	100.000

<Chromatogram>

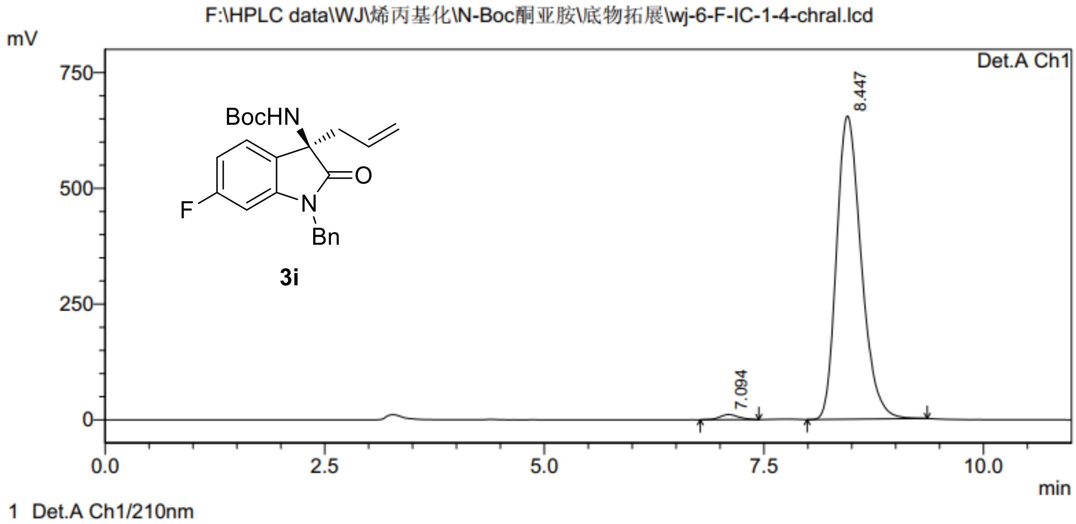

PeakTable

Detector A Ch1 210nm

Peak#	Ret. Time	Area	Height	Area %	Height %
1	7.094	166174	11228	1.284	1.685
2	8.447	12780136	655072	98.716	98.315
Total		12946310	666300	100.000	100.000

Figure S35. HPLC spectrum of **3i**, related to **Figure 1.**

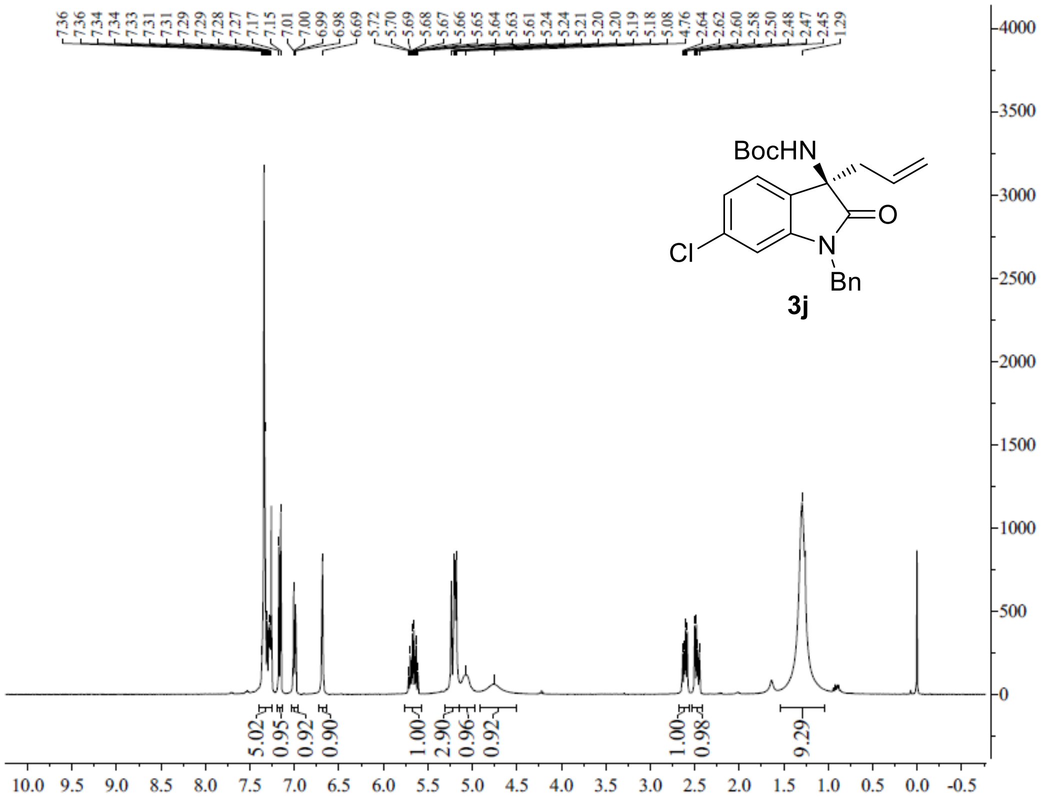

Figure S36. ^{1}H NMR spectrum of **3j**, related to **Figure 1**.

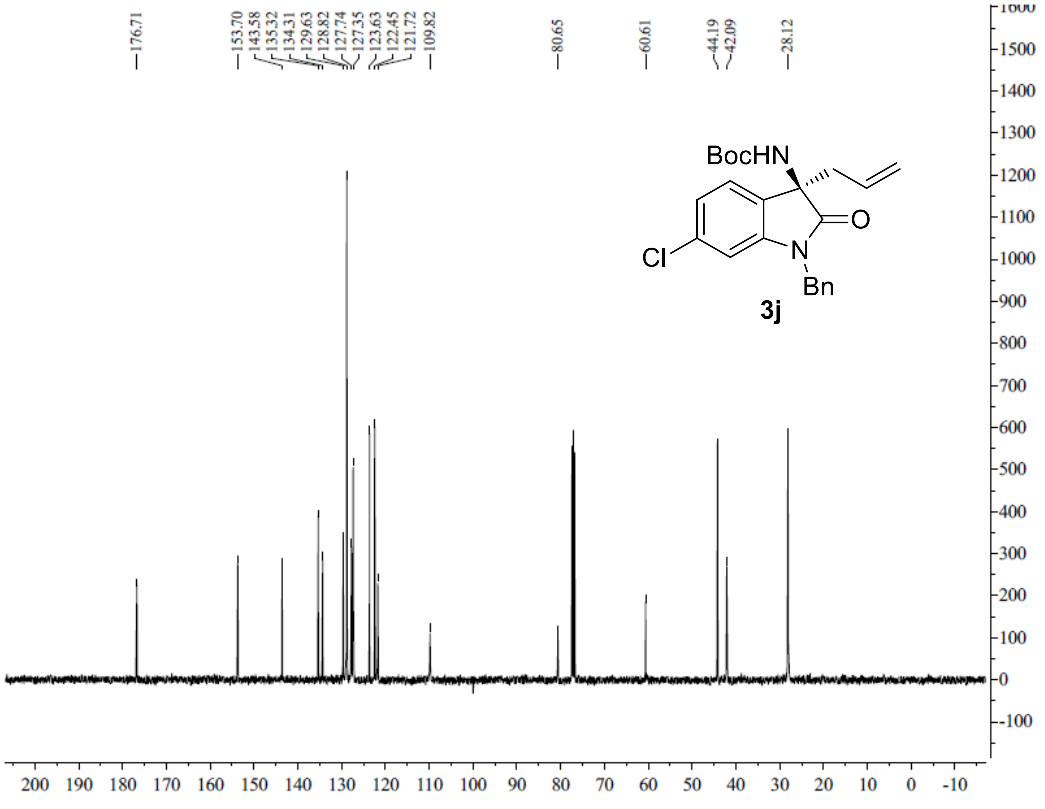

Figure S37. ^{13}C NMR spectrum of **3j**, related to **Figure 1.**

<Chromatogram>

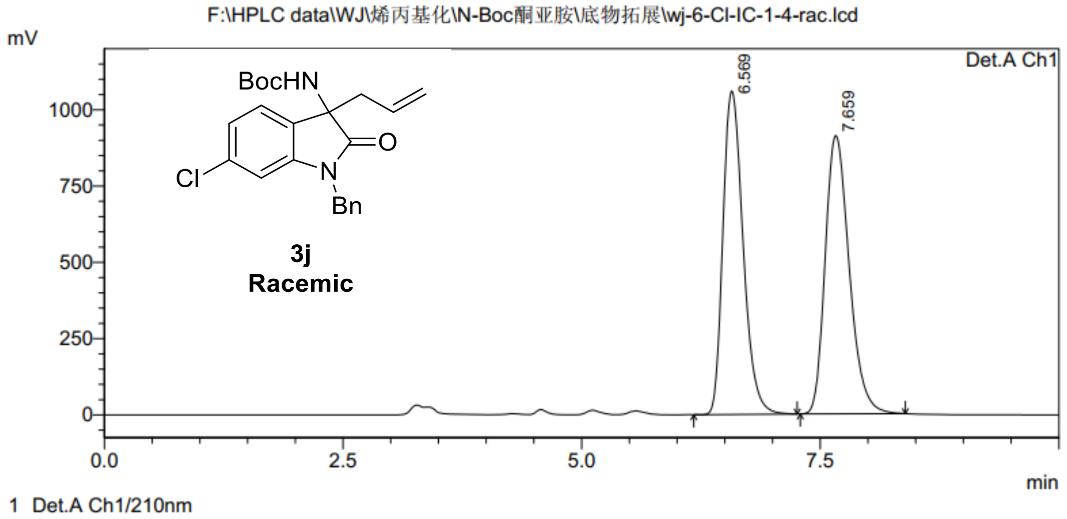

PeakTable

Detector A Ch1 210nm

Peak#	Ret. Time	Area	Height	Area %	Height %
1	6.569	15593579	1060371	49.377	53.754
2	7.659	15987190	912266	50.623	46.246
Total		31580768	1972637	100.000	100.000

<Chromatogram>

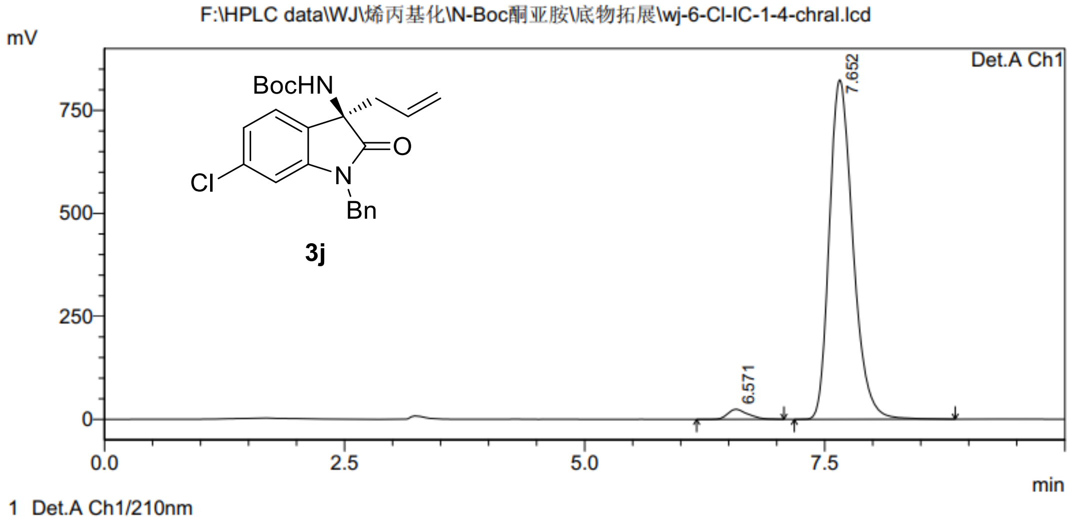

PeakTable

Detector A Ch1 210nm

Peak#	Ret. Time	Area	Height	Area %	Height %
1	6.571	355176	24346	2.461	2.870
2	7.652	14076376	824049	97.539	97.130
Total		14431552	848394	100.000	100.000

Figure S38. HPLC spectrum of **3j**, related to **Figure 1.**

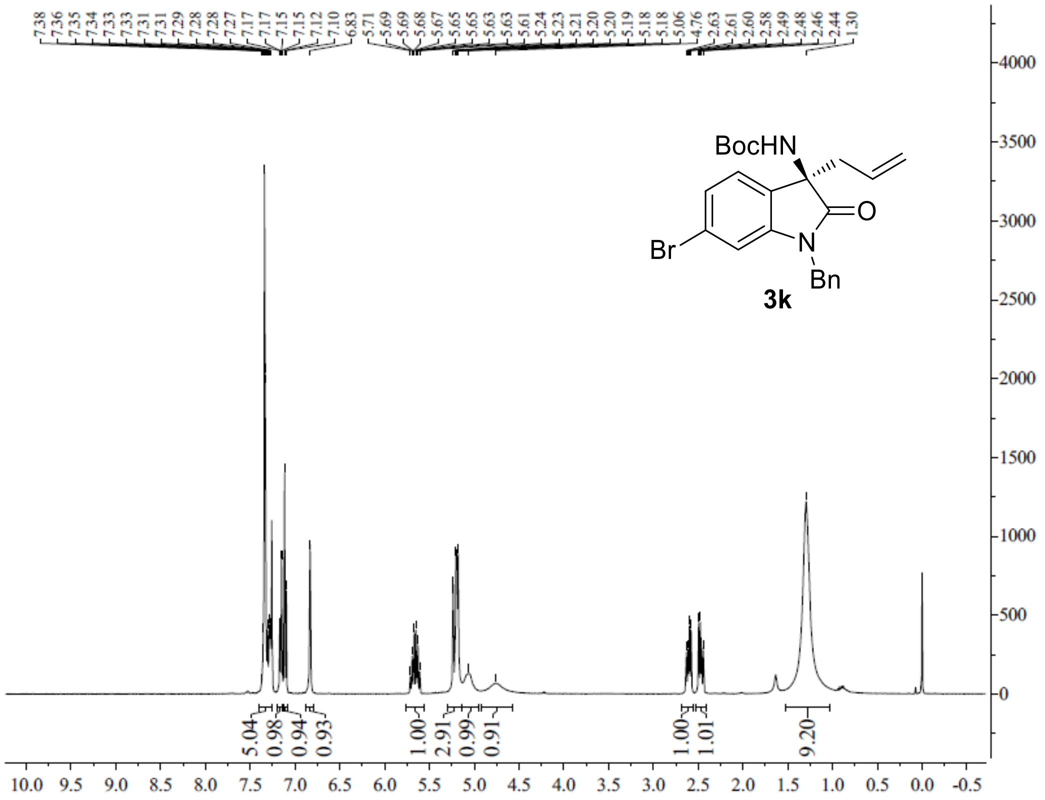

Figure S39. ^{1}H NMR spectrum of **3k**, related to **Figure 1**.

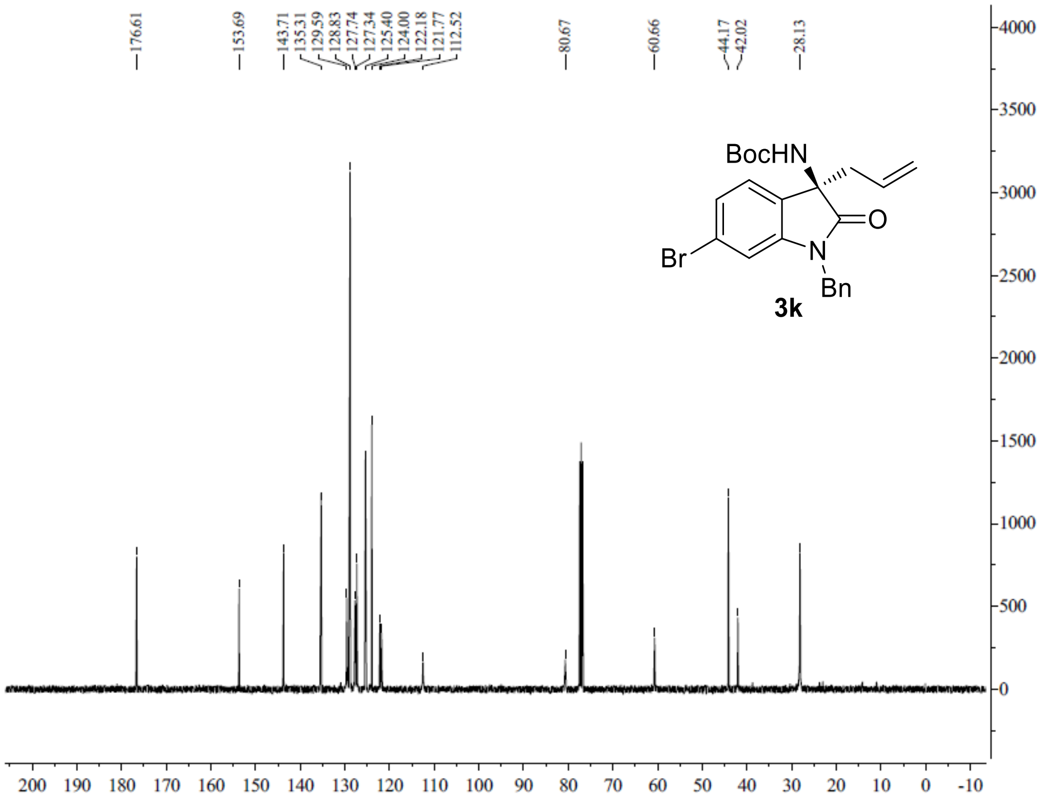

Figure S40. ^{13}C NMR spectrum of **3k**, related to **Figure 1.**

<Chromatogram>

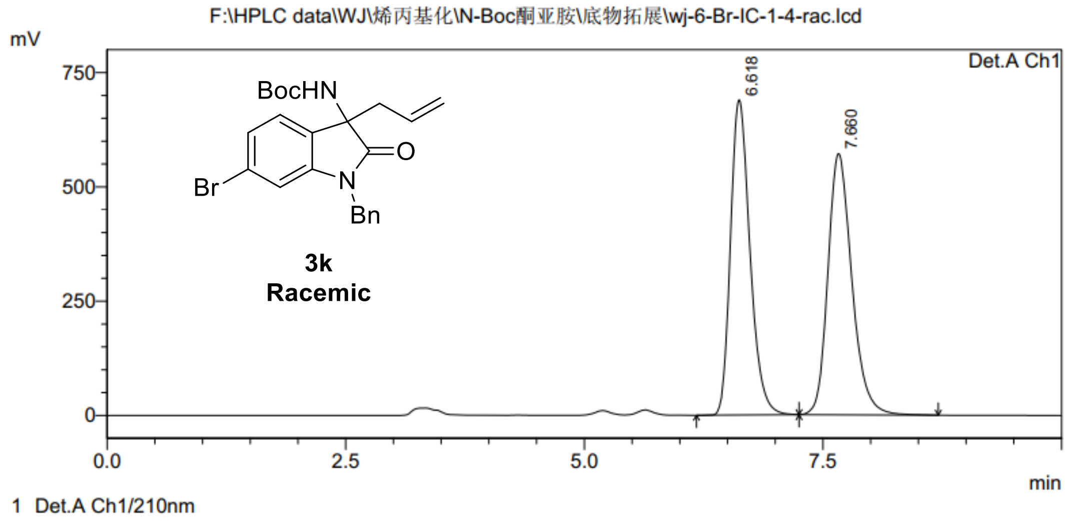

PeakTable

Detector A Ch1 210nm

Peak#	Ret. Time	Area	Height	Area %	Height %
1	6.618	9766618	689284	49.458	54.676
2	7.660	9980695	571382	50.542	45.324
Total		19747313	1260666	100.000	100.000

<Chromatogram>

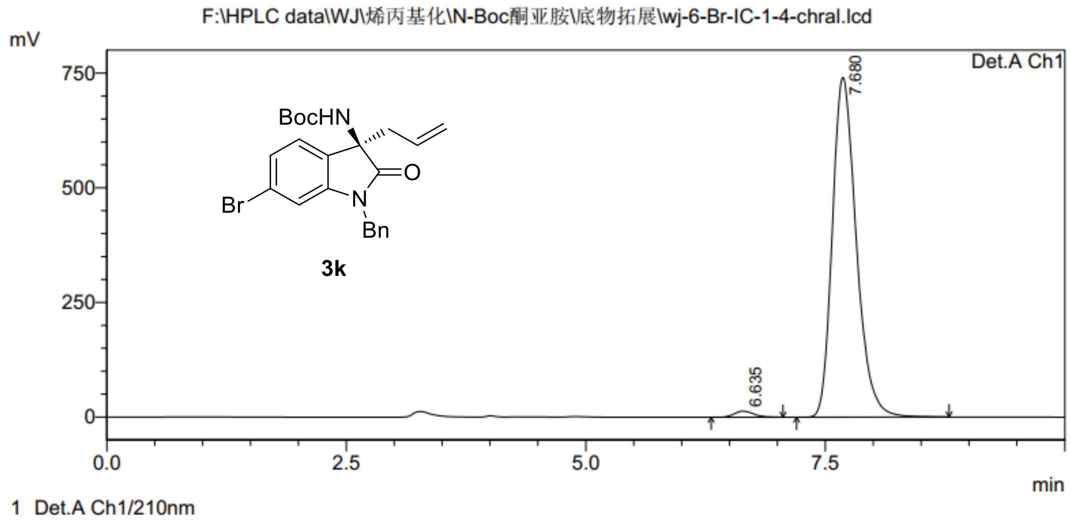

PeakTable

Detector A Ch1 210nm

Peak#	Ret. Time	Area	Height	Area %	Height %
1	6.635	184844	13503	1.414	1.790
2	7.680	12885529	740900	98.586	98.210
Total		13070373	754403	100.000	100.000

Figure S41. HPLC spectrum of **3k**, related to **Figure 1.**

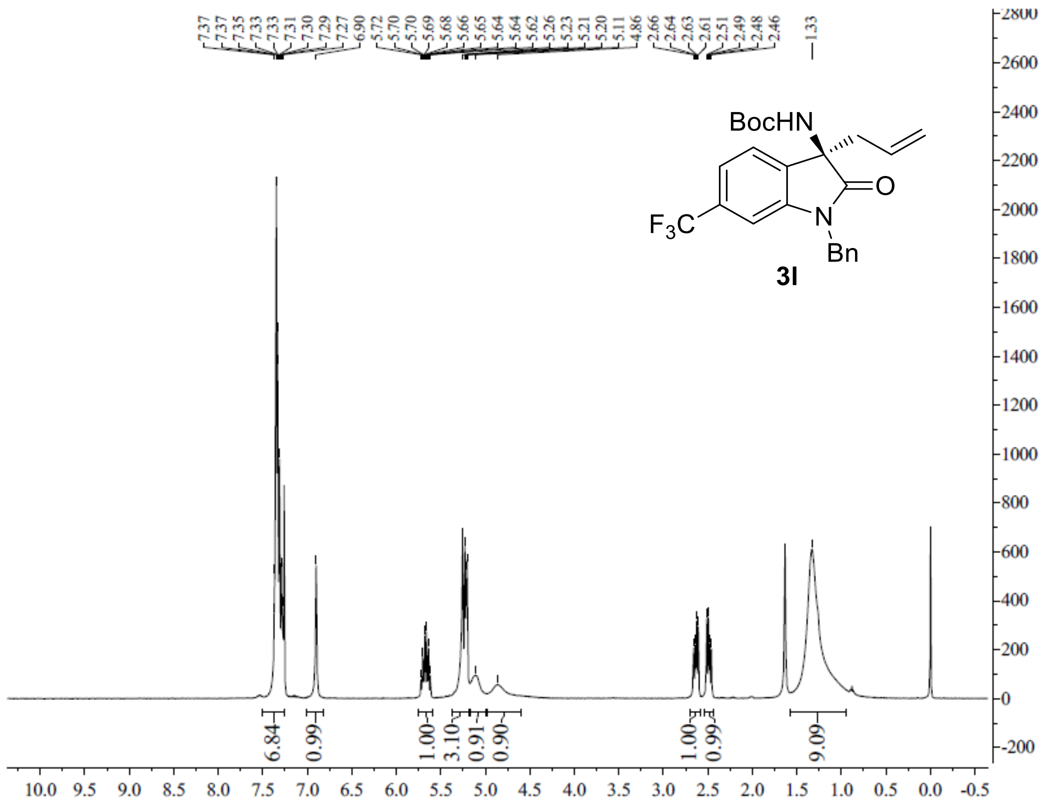

Figure S42. ^{1}H NMR spectrum of **3l**, related to **Figure 1**.

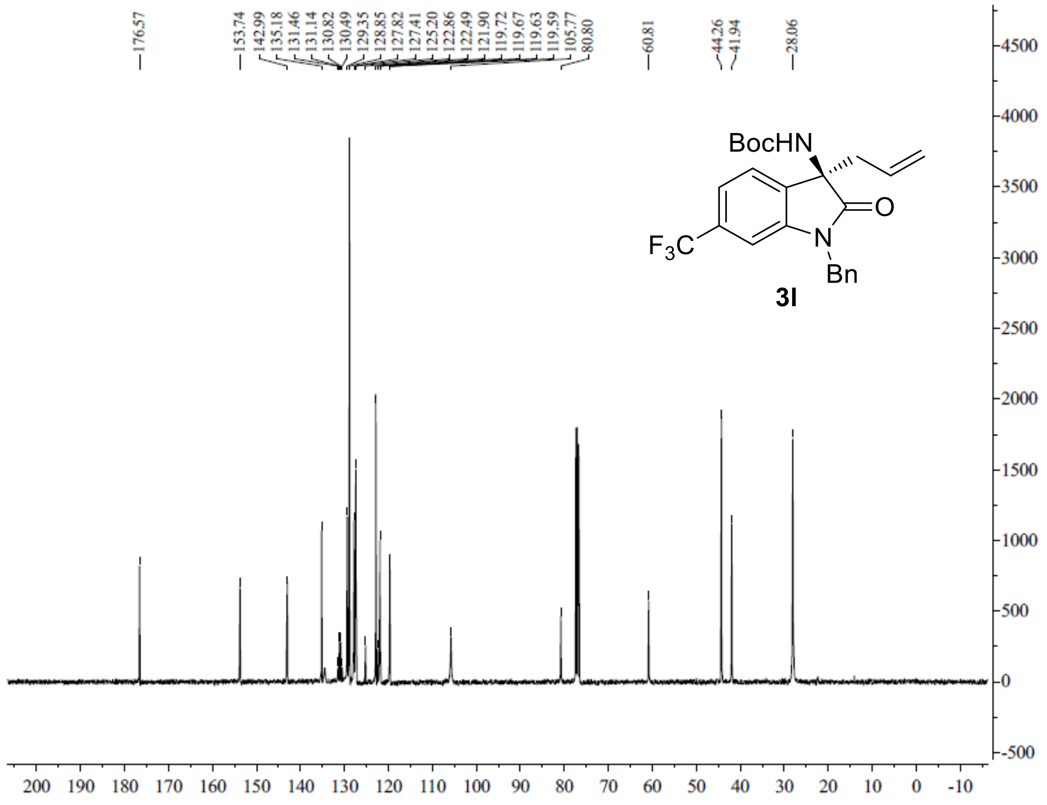

Figure S43. ^{13}C NMR spectrum of **3l**, related to **Figure 1.**

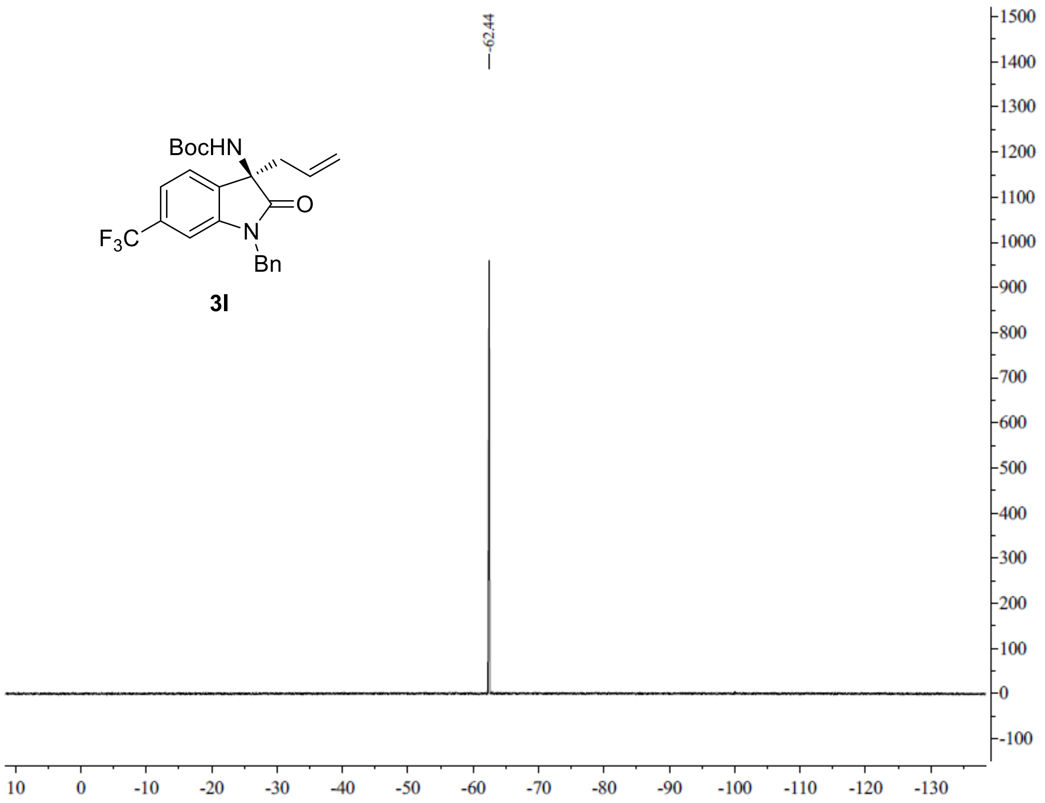

Figure S44. ^{19}F NMR spectrum of **3l**, related to **Figure 1.**

<Chromatogram>

F:\HPLC data\WJ\烯丙基化\N-Boc酮亚胺\补充数据\可用\6-CF3-IC-1-4-rac.lcd

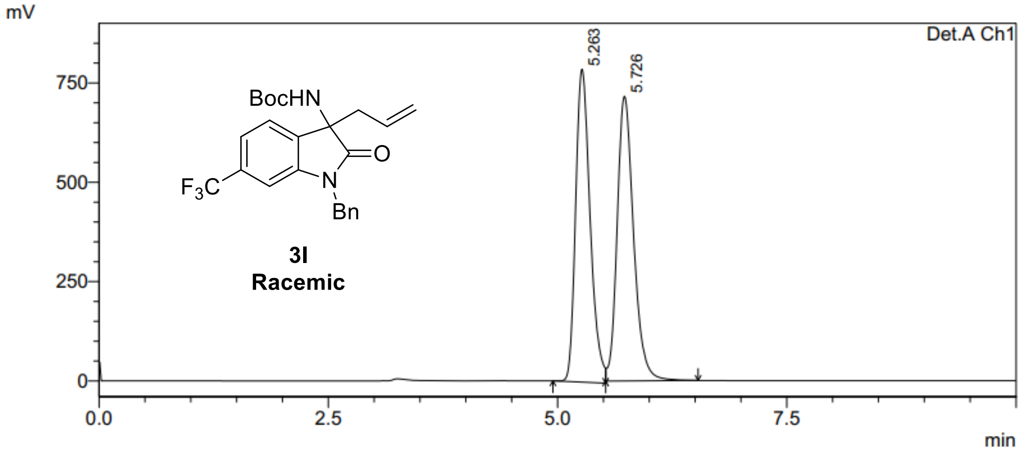

1 Det.A Ch1/210nm

PeakTable

Detector A Ch1 210nm

Peak#	Ret. Time	Area	Height	Area %	Height %
1	5.263	8666069	787903	49.257	52.381
2	5.726	8927584	716275	50.743	47.619
Total		17593652	1504177	100.000	100.000

<Chromatogram>

F:\HPLC data\WJ\烯丙基化\N-Boc酮亚胺\补充数据\可用\6-CF3-IC-1-4-chiral.lcd

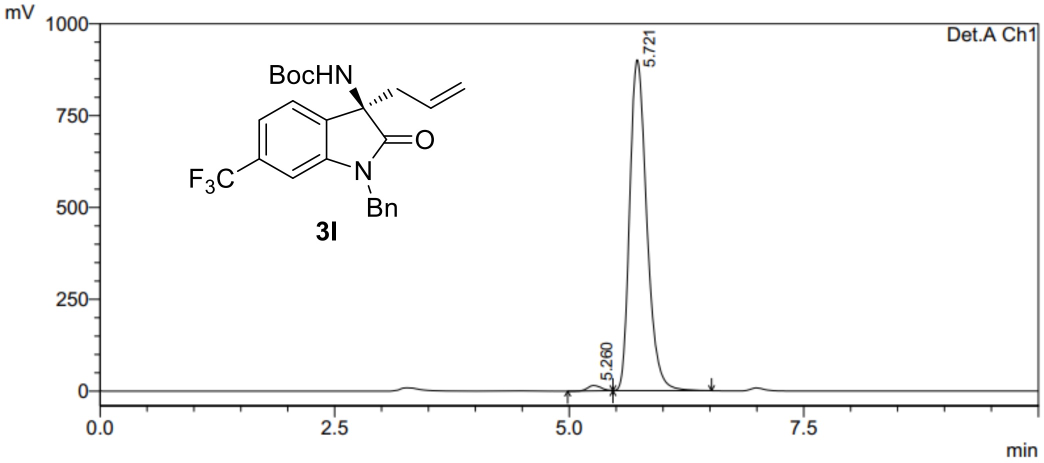

1 Det.A Ch1/210nm

PeakTable

Detector A Ch1 210nm

Peak#	Ret. Time	Area	Height	Area %	Height %
1	5.260	140188	14672	1.217	1.603
2	5.721	11382069	900525	98.783	98.397
Total		11522257	915196	100.000	100.000

Figure S45. HPLC spectrum of **3l**, related to **Figure 1.**

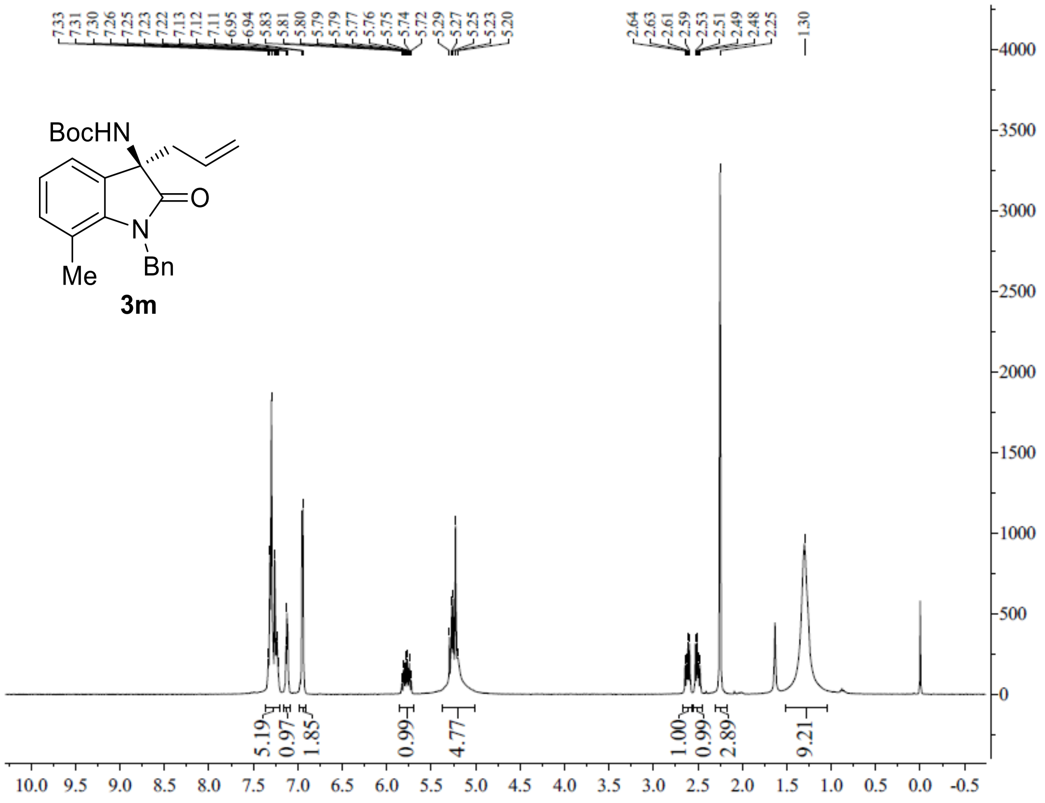

Figure S46. ^{1}H NMR spectrum of **3m**, related to **Figure 1**.

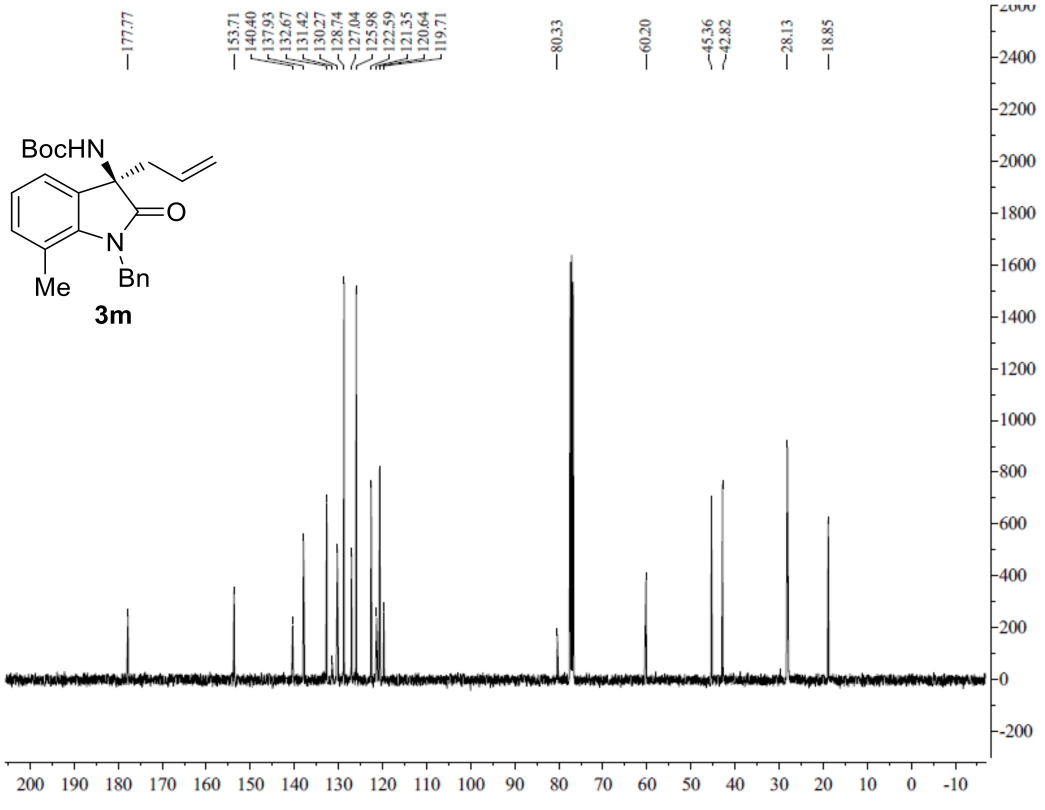

Figure S47. ^{13}C NMR spectrum of **3m**, related to **Figure 1.**

<Chromatogram>

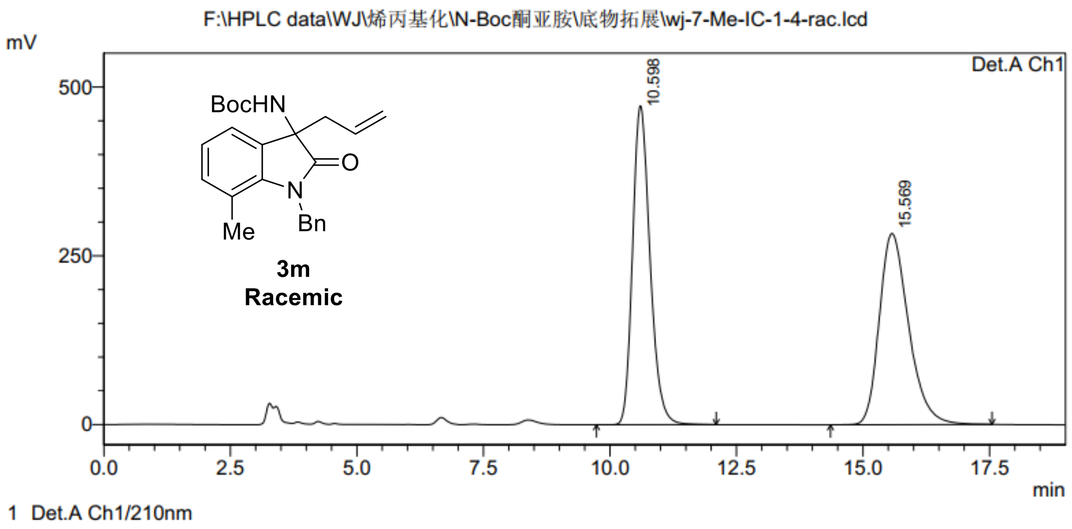

PeakTable

Detector A Ch1 210nm

Peak#	Ret. Time	Area	Height	Area %	Height %
1	10.598	11329291	472367	49.839	62.522
2	15.569	11402366	283156	50.161	37.478
Total		22731657	755523	100.000	100.000

<Chromatogram>

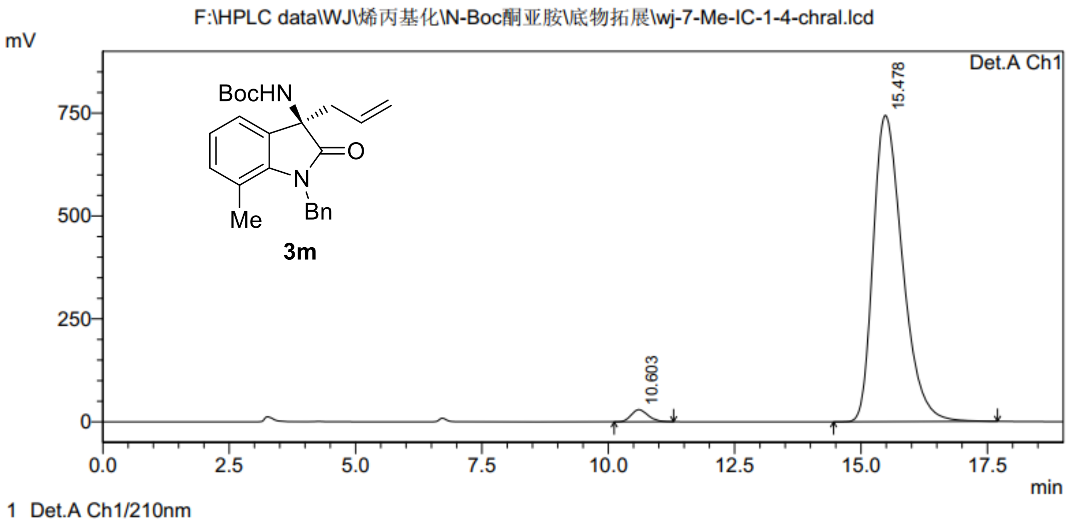

PeakTable

Detector A Ch1 210nm

Peak#	Ret. Time	Area	Height	Area %	Height %
1	10.603	683820	29574	2.223	3.821
2	15.478	30080164	744467	97.777	96.179
Total		30763984	774042	100.000	100.000

Figure S48. HPLC spectrum of **3m**, related to **Figure 1.**

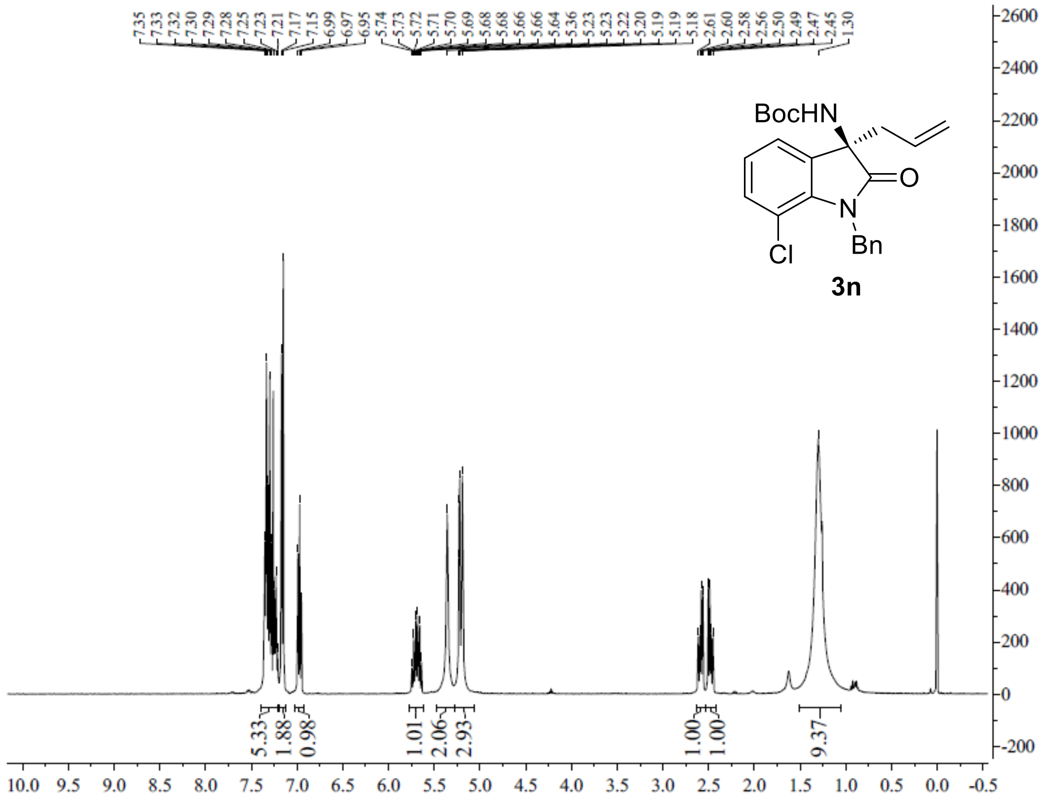

Figure S49. ^{1}H NMR spectrum of **3n**, related to **Figure 1**.

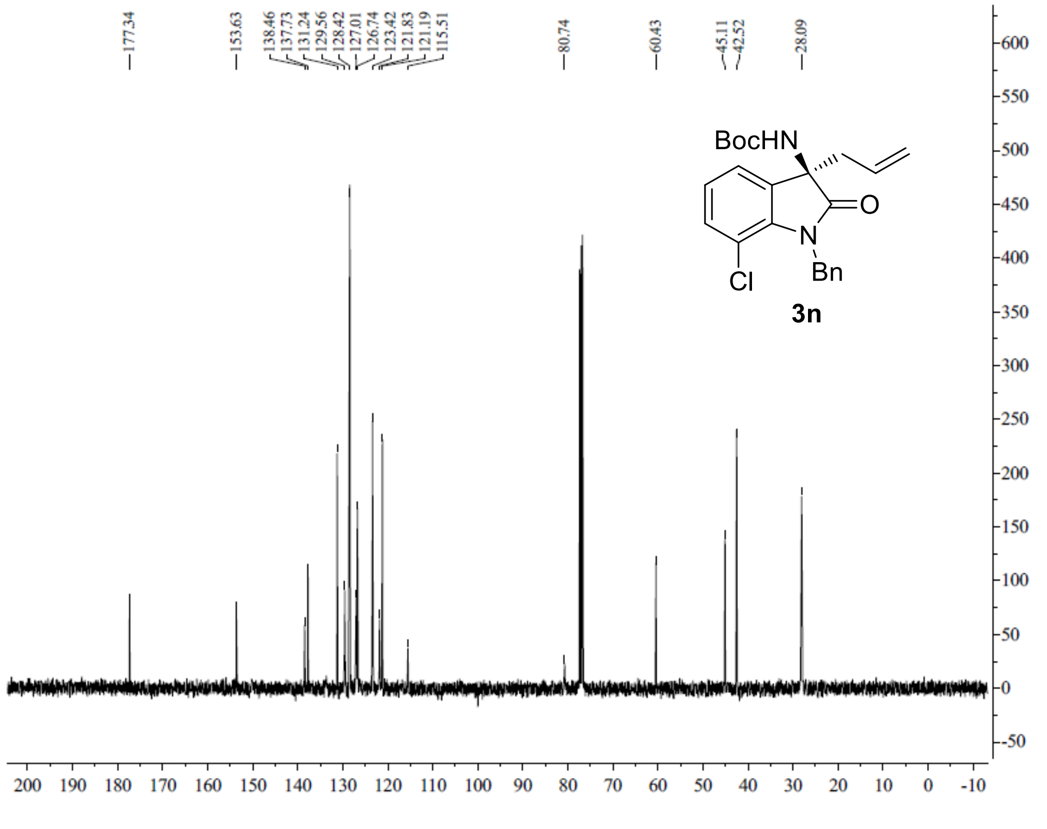

Figure S50. ^{13}C NMR spectrum of **3n**, related to **Figure 1.**

<Chromatogram>

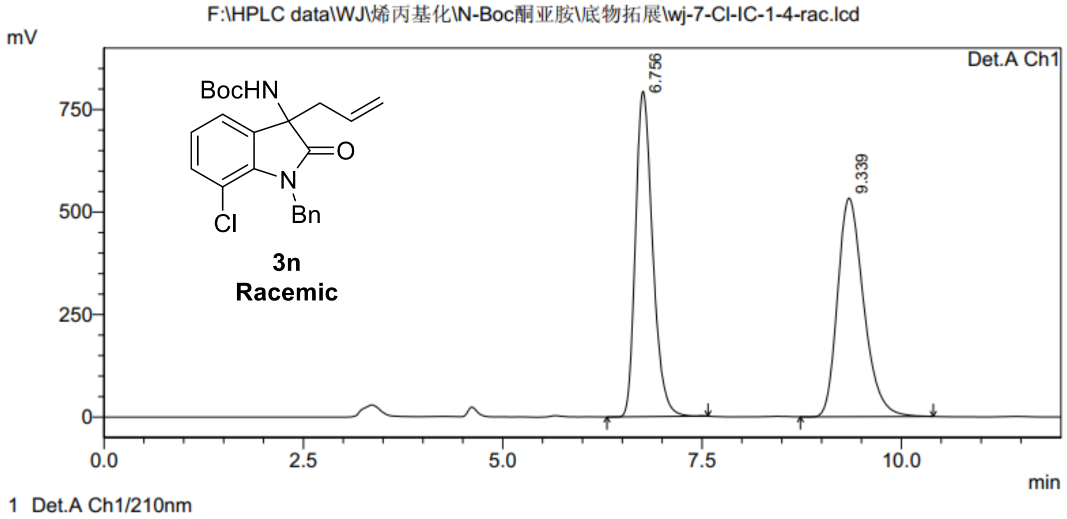

PeakTable

Detector A Ch1 210nm

Peak#	Ret. Time	Area	Height	Area %	Height %
1	6.756	11877544	793799	49.478	59.810
2	9.339	12128051	533413	50.522	40.190
Total		24005595	1327212	100.000	100.000

<Chromatogram>

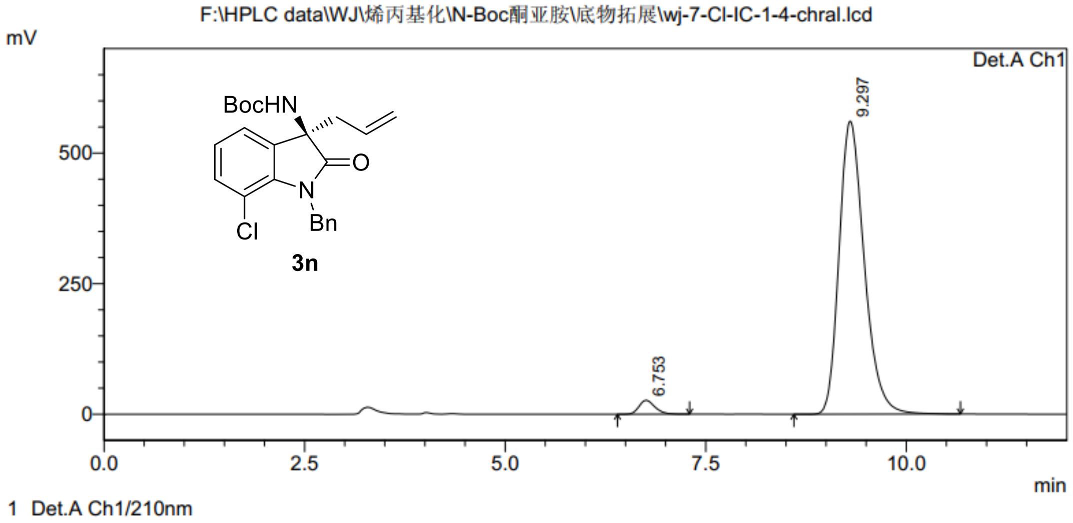

PeakTable

Detector A Ch1 210nm

Peak#	Ret. Time	Area	Height	Area %	Height %
1	6.753	366443	26687	2.907	4.542
2	9.297	12239114	560867	97.093	95.458
Total		12605557	587553	100.000	100.000

Figure S51. HPLC spectrum of **3n**, related to **Figure 1.**

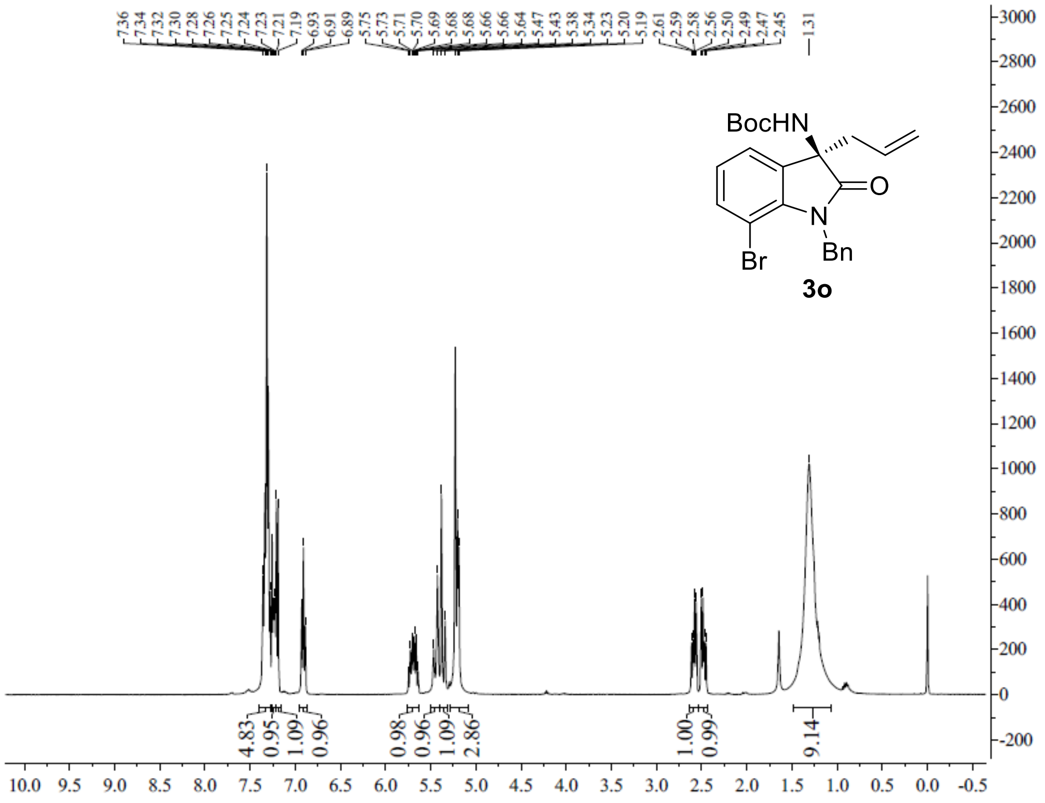

Figure S52. ^{1}H NMR spectrum of **3o**, related to **Figure 1**.

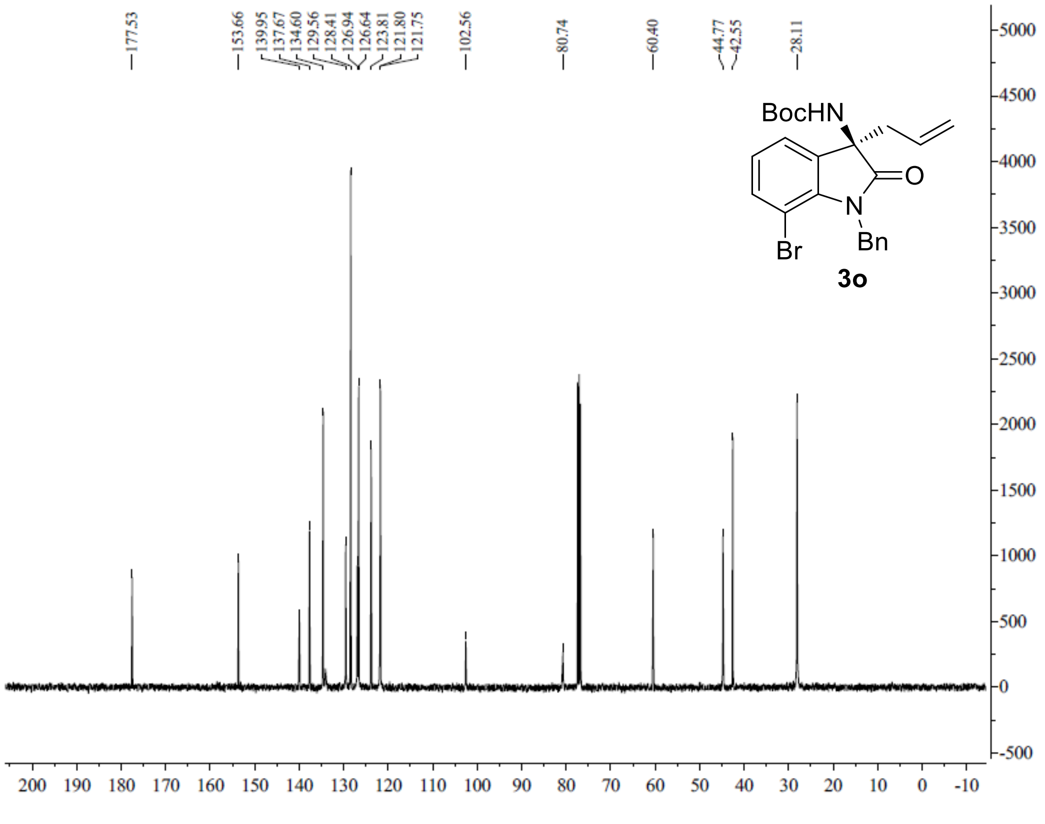

Figure S53. ^{13}C NMR spectrum of **3o**, related to **Figure 1.**

<Chromatogram>

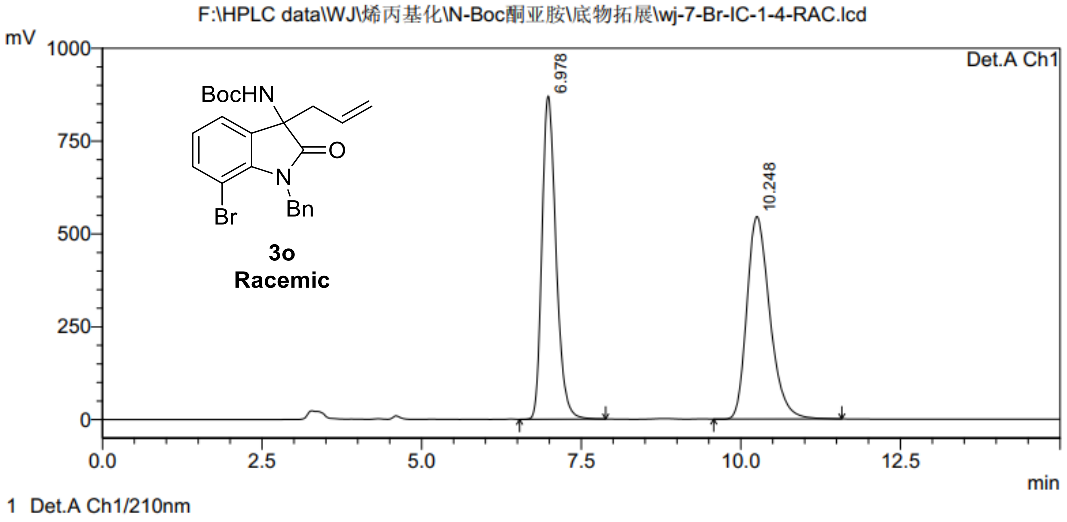

PeakTable

Detector A Ch1 210nm

Peak#	Ret. Time	Area	Height	Area %	Height %
1	6.978	13342936	870718	49.425	61.474
2	10.248	13653647	545678	50.575	38.526
Total		26996583	1416396	100.000	100.000

<Chromatogram>

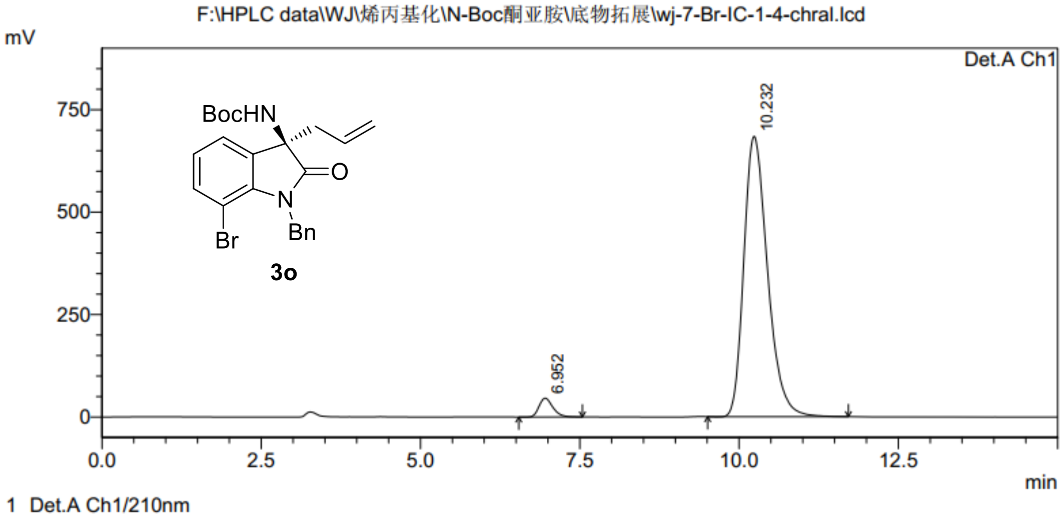

PeakTable

Detector A Ch1 210nm

Peak#	Ret. Time	Area	Height	Area %	Height %
1	6.952	692023	45979	3.764	6.297
2	10.232	17692340	684203	96.236	93.703
Total		18384363	730182	100.000	100.000

Figure S54. HPLC spectrum of **3o**, related to **Figure 1.**

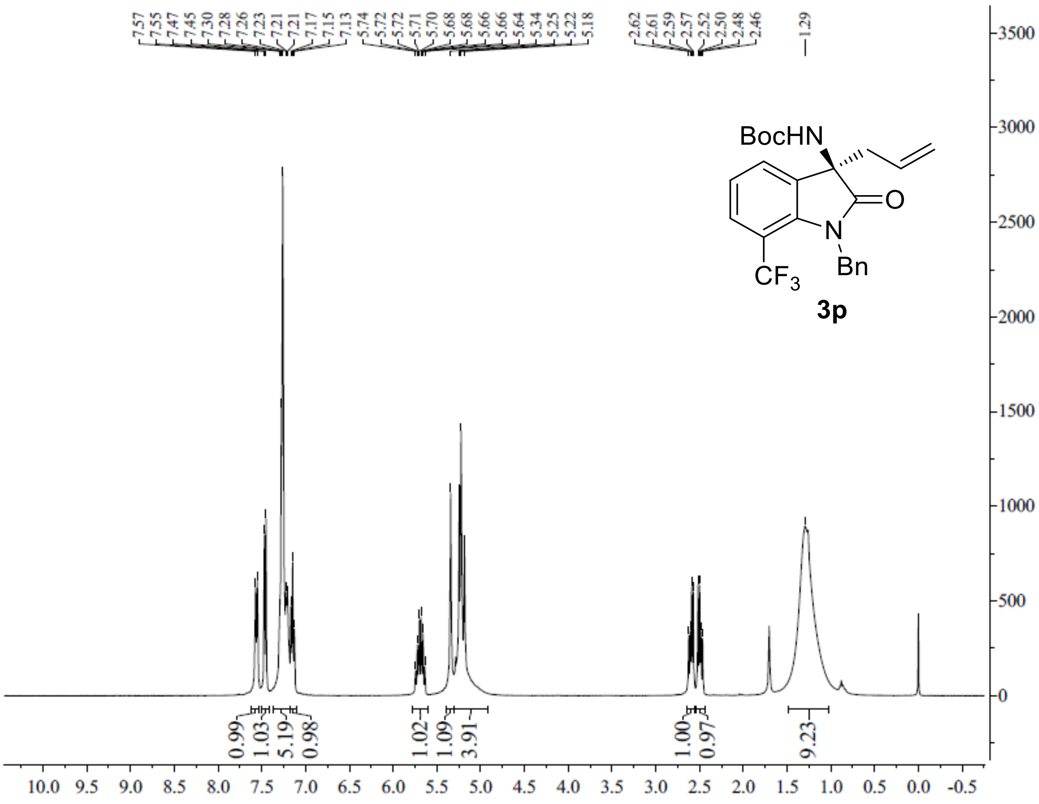

Figure S55. ^{1}H NMR spectrum of **3p**, related to **Figure 1**.

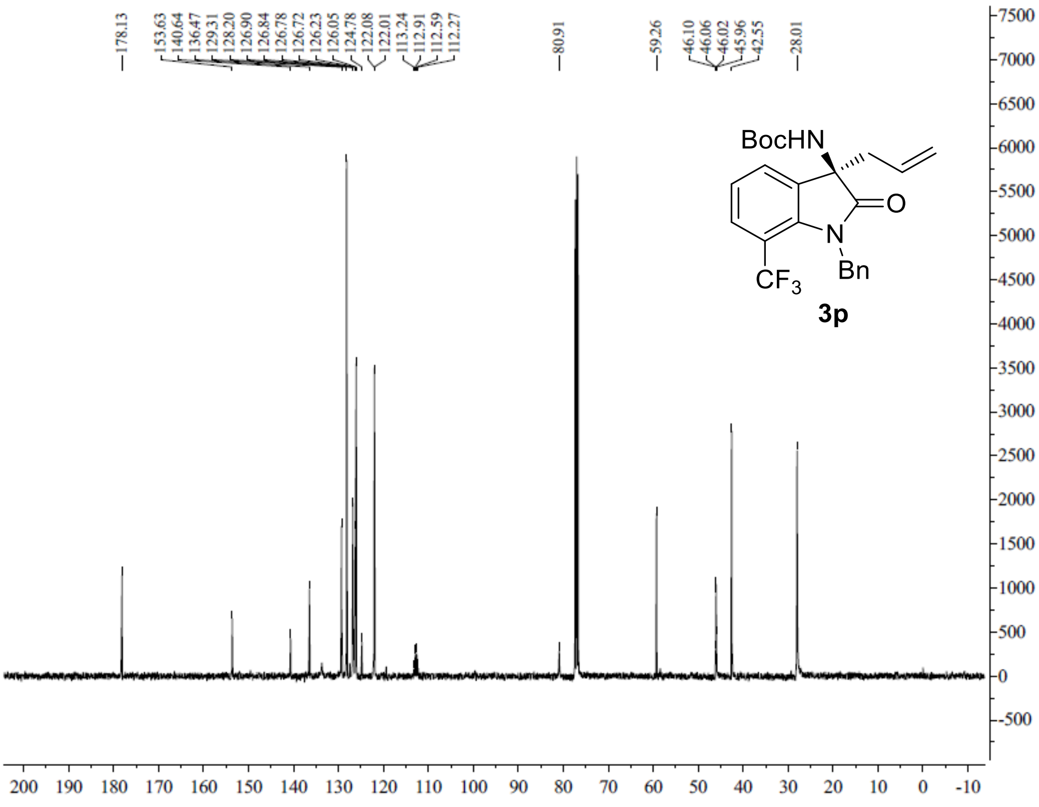

Figure S56. ^{13}C NMR spectrum of **3p**, related to **Figure 1.**

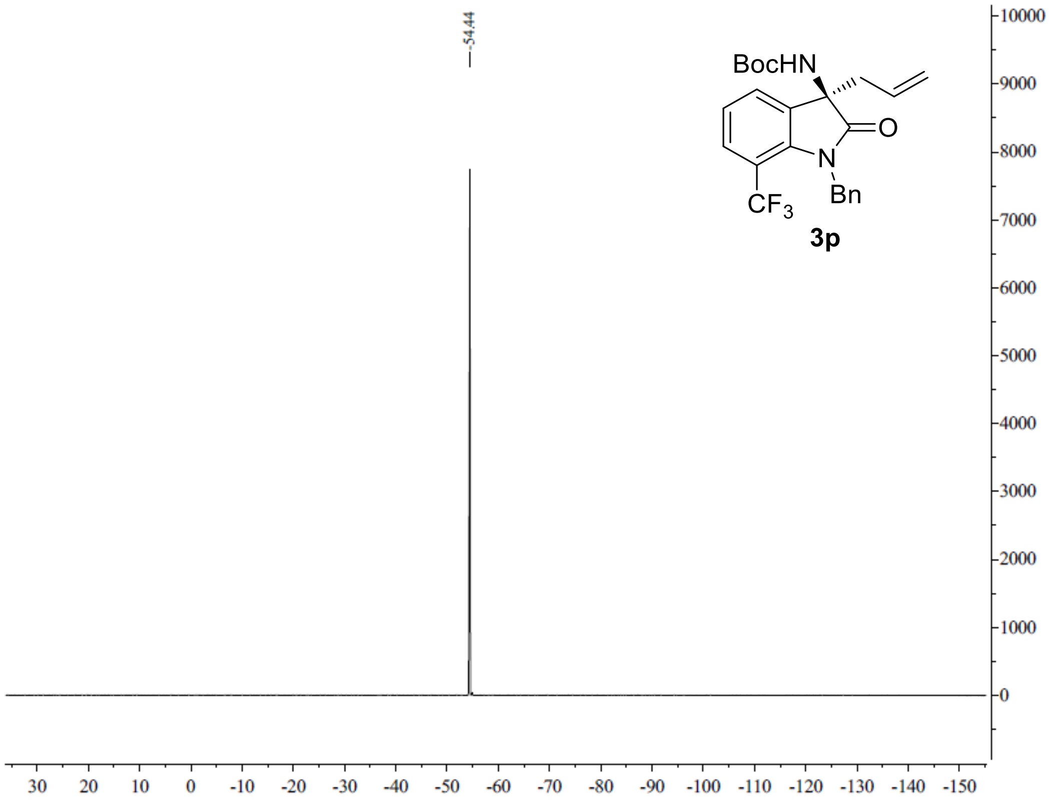

Figure S57. ^{19}F NMR spectrum of **3p**, related to **Figure 1.**

<Chromatogram>

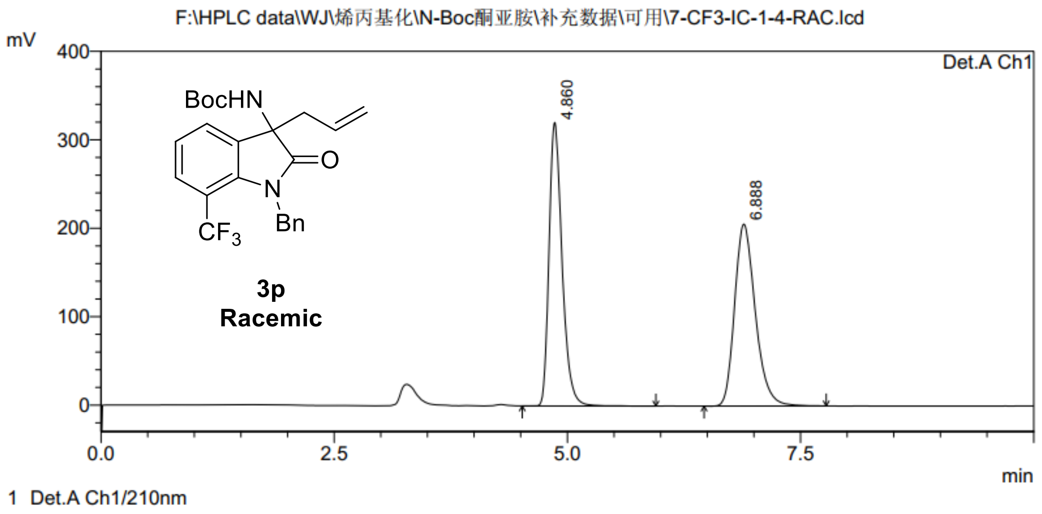

PeakTable

Detector A Ch1 210nm

Peak#	Ret. Time	Area	Height	Area %	Height %
1	4.860	3109314	320668	49.465	60.905
2	6.888	3176594	205840	50.535	39.095
Total		6285908	526508	100.000	100.000

<Chromatogram>

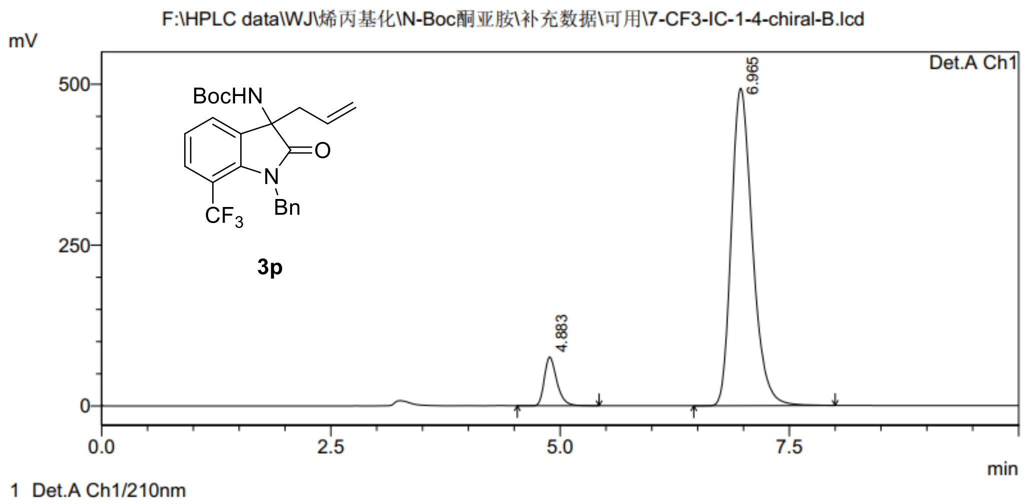

PeakTable

Detector A Ch1 210nm

Peak#	Ret. Time	Area	Height	Area %	Height %
1	4.883	714726	75606	8.325	13.292
2	6.965	7870098	493203	91.675	86.708
Total		8584824	568809	100.000	100.000

Figure S58. HPLC spectrum of **3p**, related to **Figure 1.**

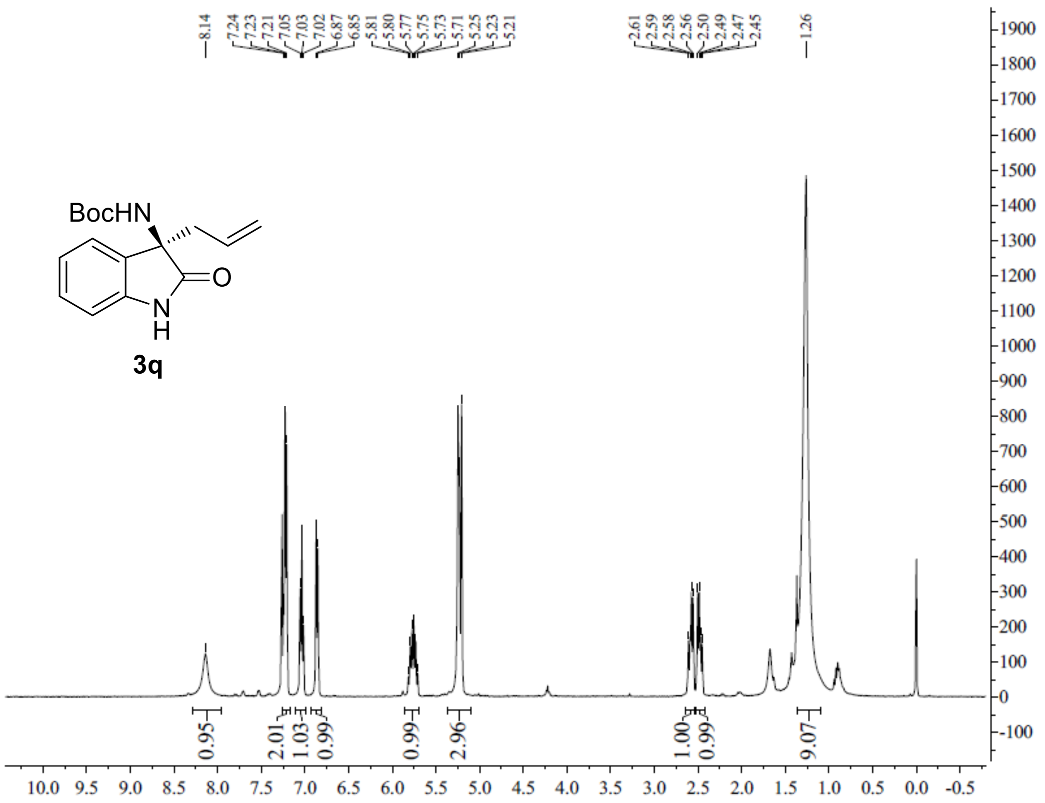

Figure S59. ^{1}H NMR spectrum of **3q**, related to **Figure 2**.

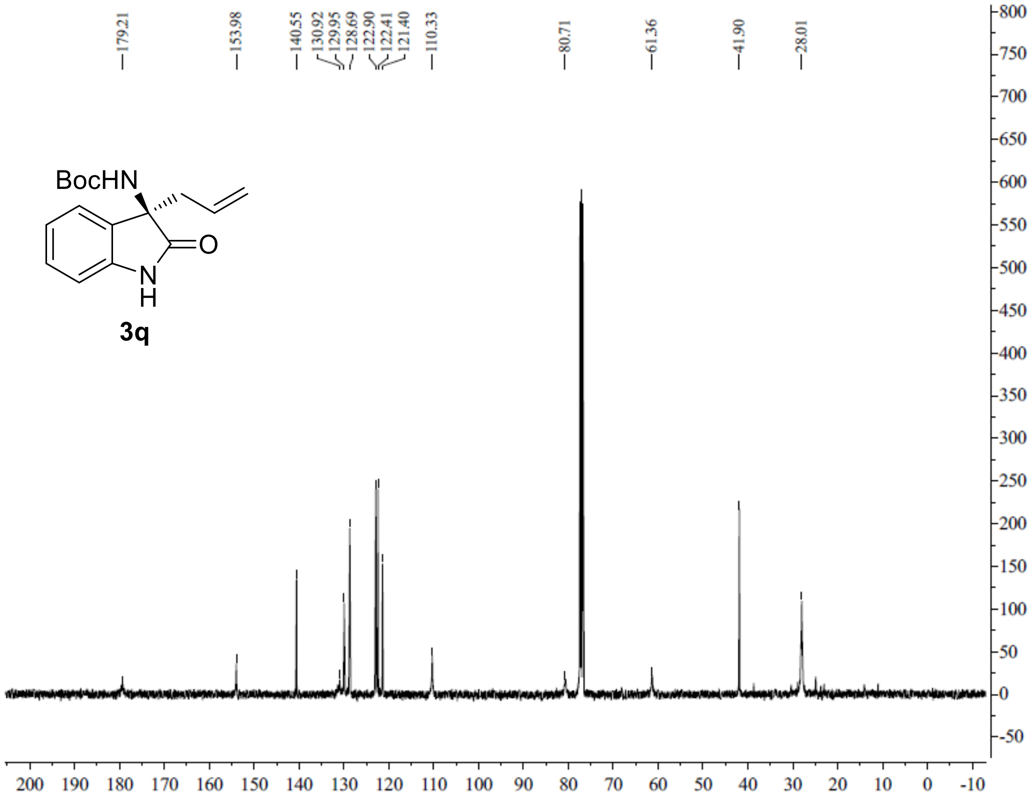

Figure S60. ^{13}C NMR spectrum of **3q**, related to **Figure 2.**

<Chromatogram>

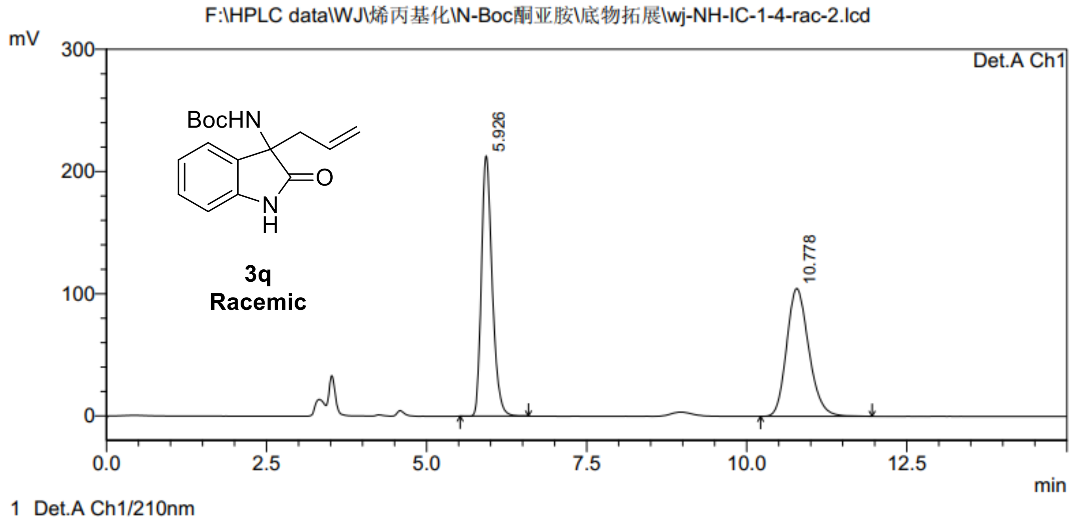

PeakTable

Detector A Ch1 210nm

Peak#	Ret. Time	Area	Height	Area %	Height %
1	5.926	2487104	212975	49.704	67.071
2	10.778	2516701	104563	50.296	32.929
Total		5003805	317538	100.000	100.000

<Chromatogram>

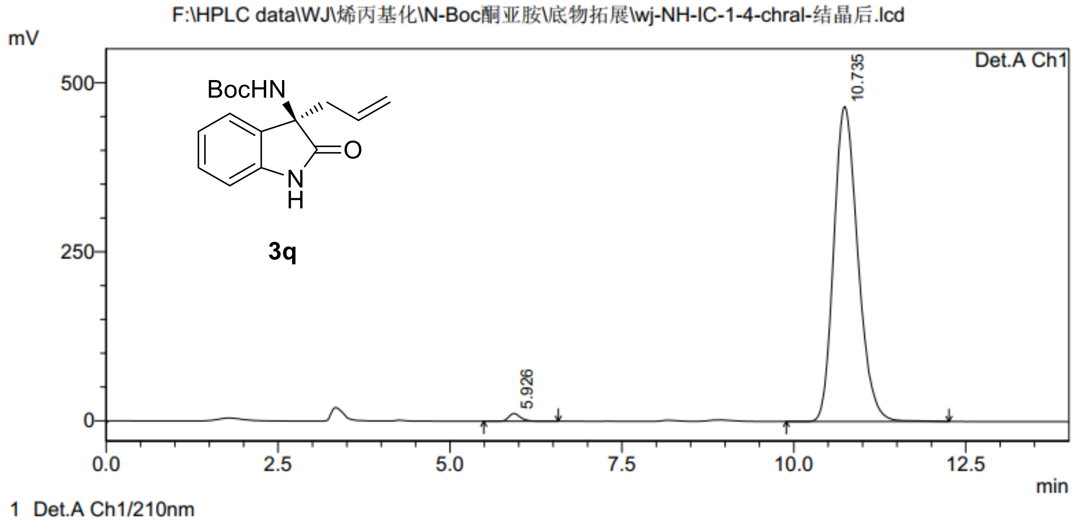

PeakTable

Detector A Ch1 210nm

Peak#	Ret. Time	Area	Height	Area %	Height %
1	5.926	136242	11445	1.188	2.399
2	10.735	11329605	465556	98.812	97.601
Total		11465847	477002	100.000	100.000

Figure S61. HPLC spectrum of **3q**, related to **Figure 2.**

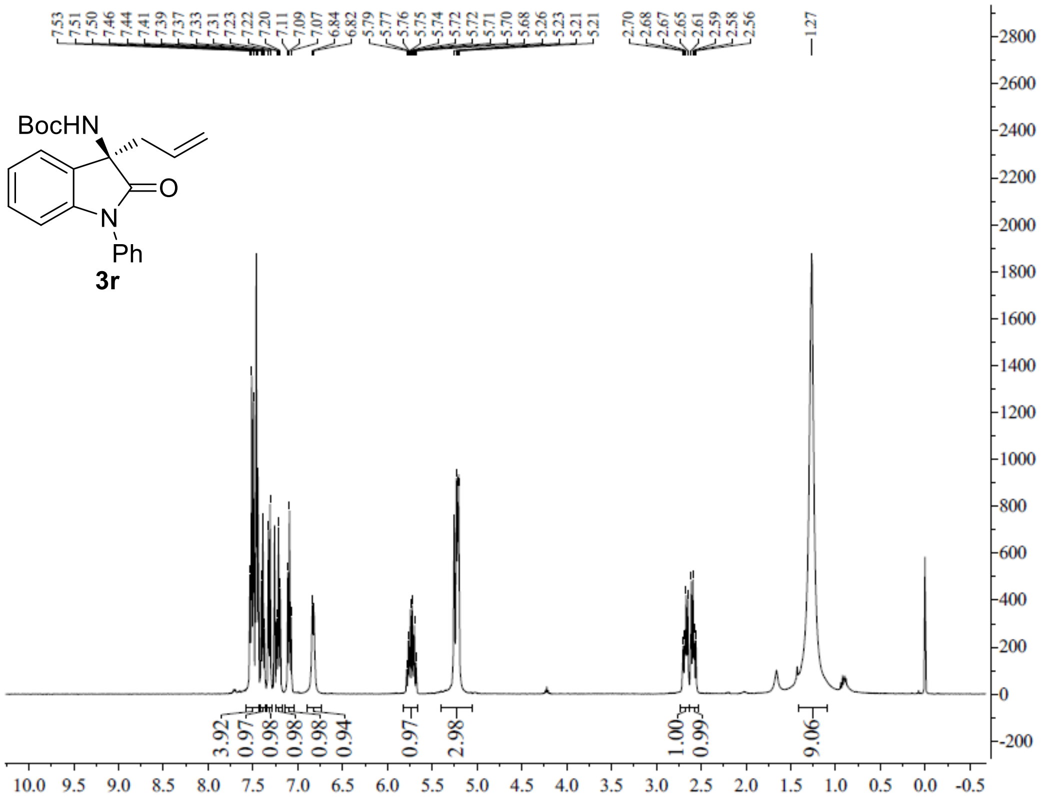

Figure S62. ^{1}H NMR spectrum of **3r**, related to **Figure 2**.

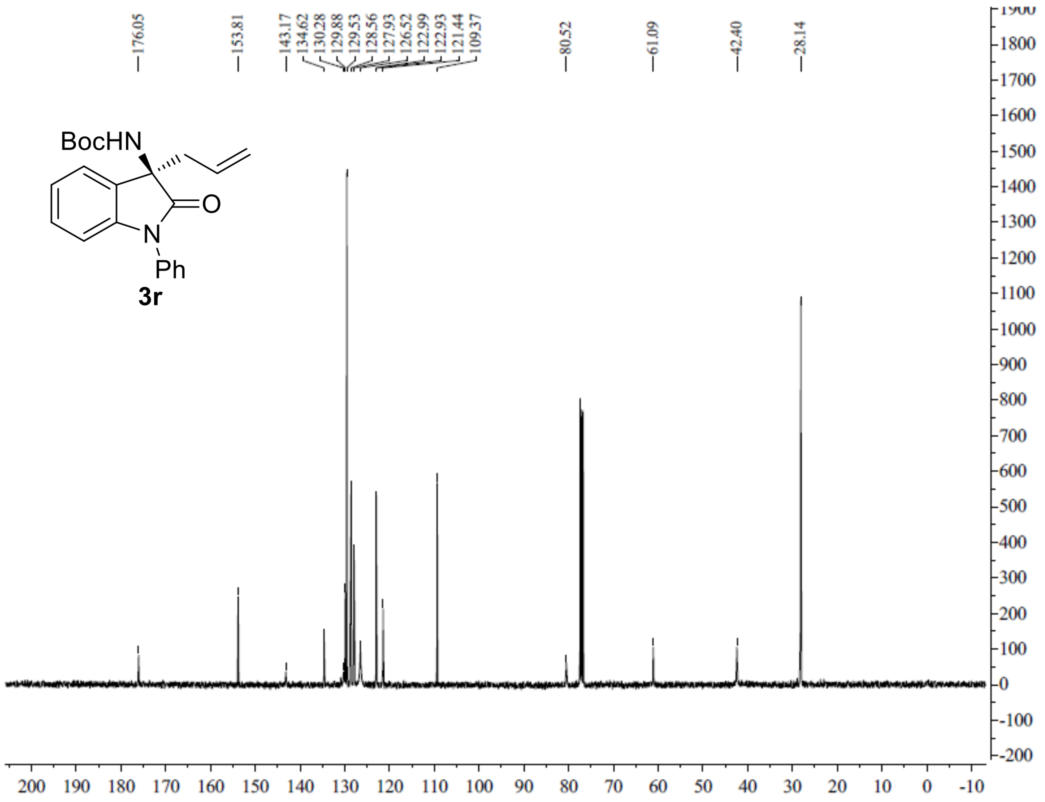

Figure S63. ^{13}C NMR spectrum of **3r**, related to **Figure 2.**

<Chromatogram>

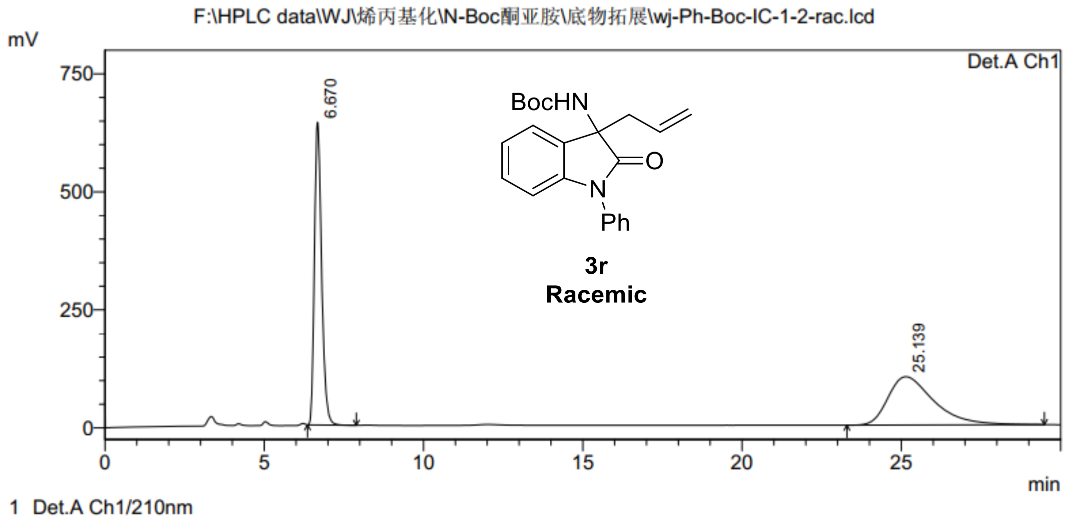

PeakTable

Detector A Ch1 210nm

Peak#	Ret. Time	Area	Height	Area %	Height %
1	6.670	10051746	641513	49.578	86.247
2	25.139	10223011	102292	50.422	13.753
Total		20274757	743805	100.000	100.000

<Chromatogram>

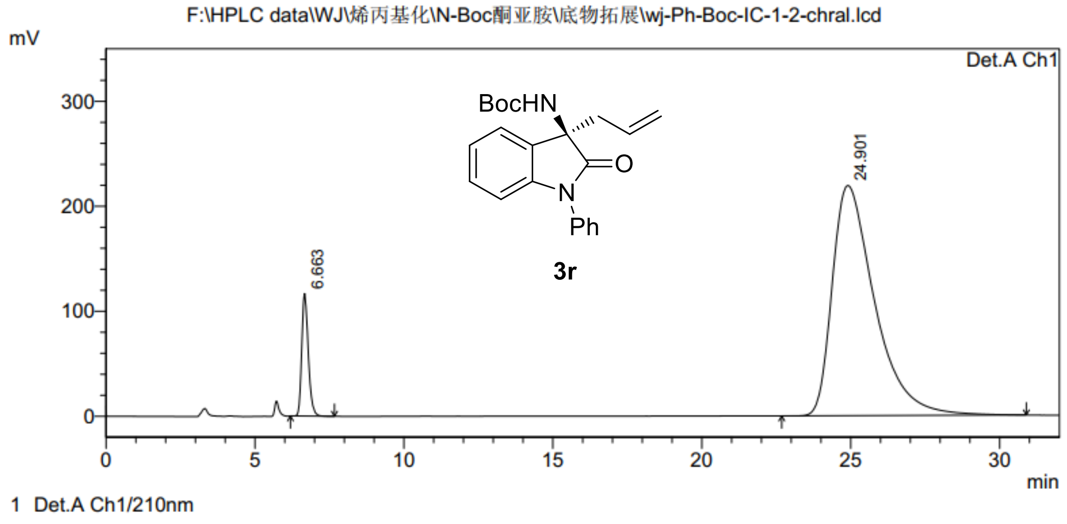

PeakTable

Detector A Ch1 210nm

Peak#	Ret. Time	Area	Height	Area %	Height %
1	6.663	1775713	116668	7.568	34.723
2	24.901	21687300	219324	92.432	65.277
Total		23463013	335991	100.000	100.000

Figure S64. HPLC spectrum of **3r**, related to **Figure 2.**

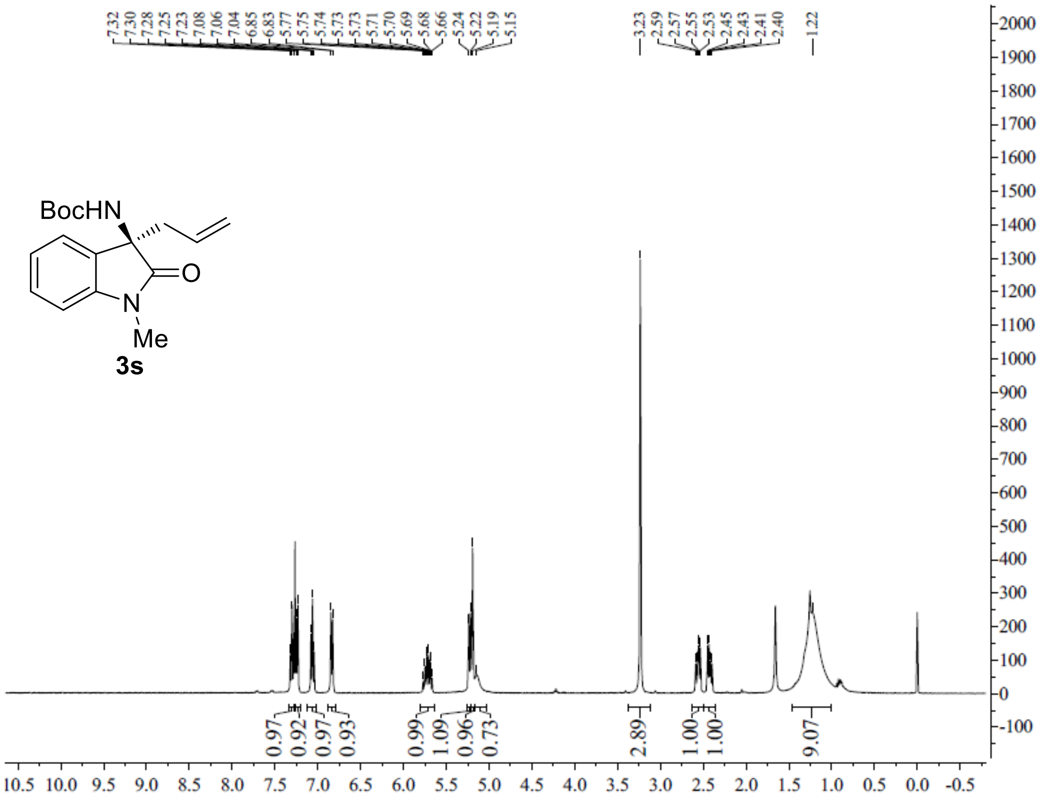

Figure S65. ^{1}H NMR spectrum of **3**s, related to **Figure 2**.

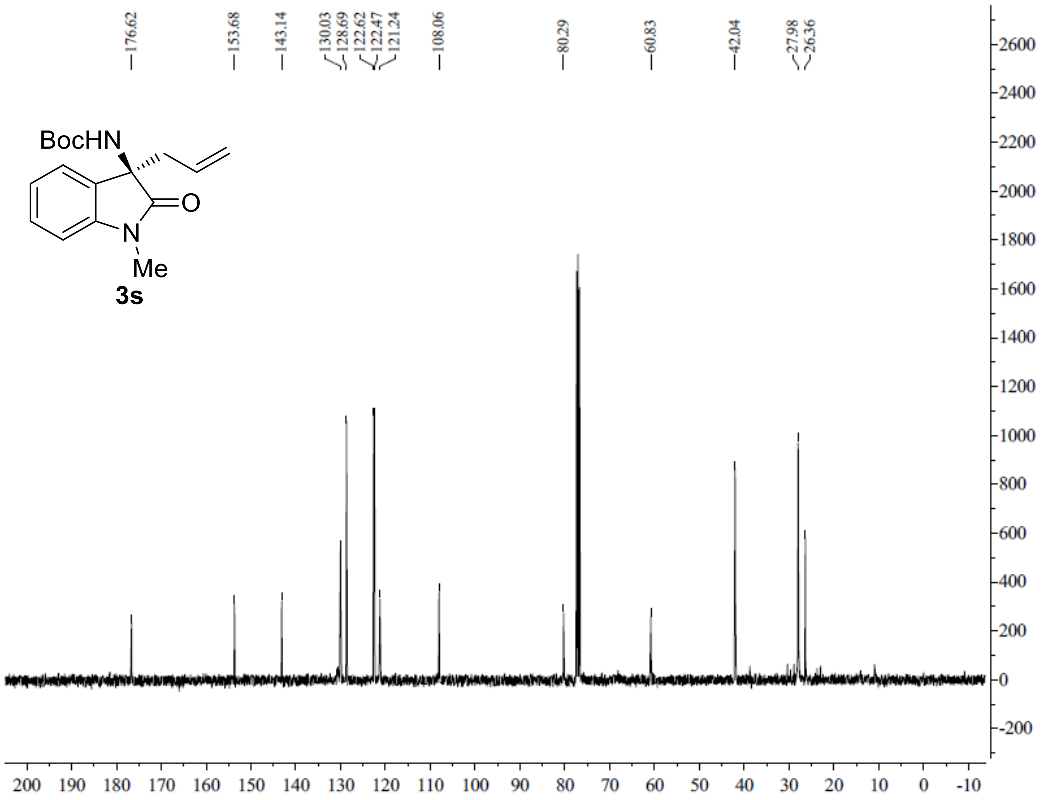

Figure S66. ^{13}C NMR spectrum of **3s**, related to **Figure 2.**

<Chromatogram>

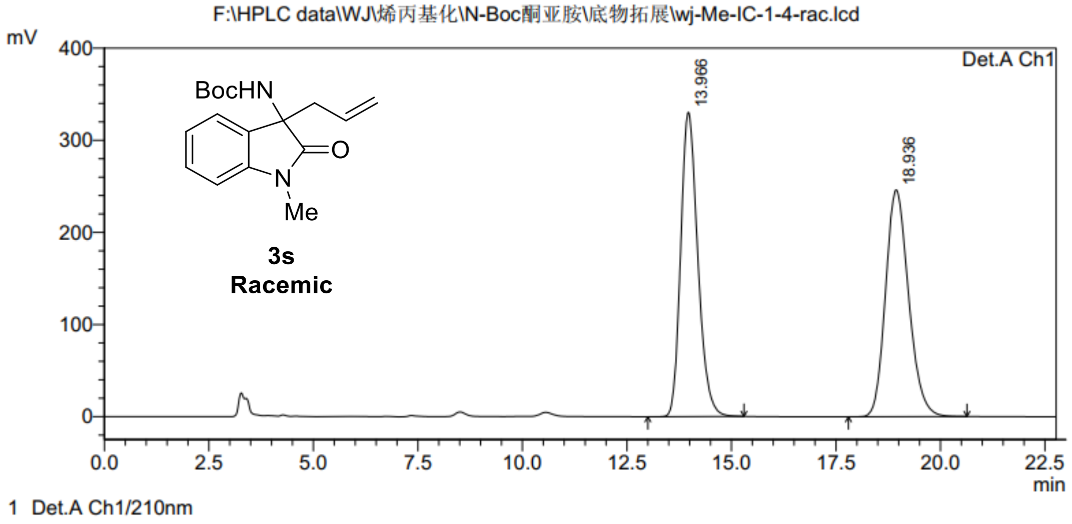

PeakTable

Detector A Ch1 210nm

Peak#	Ret. Time	Area	Height	Area %	Height %
1	13.966	9376697	330401	49.801	57.300
2	18.936	9451764	246211	50.199	42.700
Total		18828461	576613	100.000	100.000

<Chromatogram>

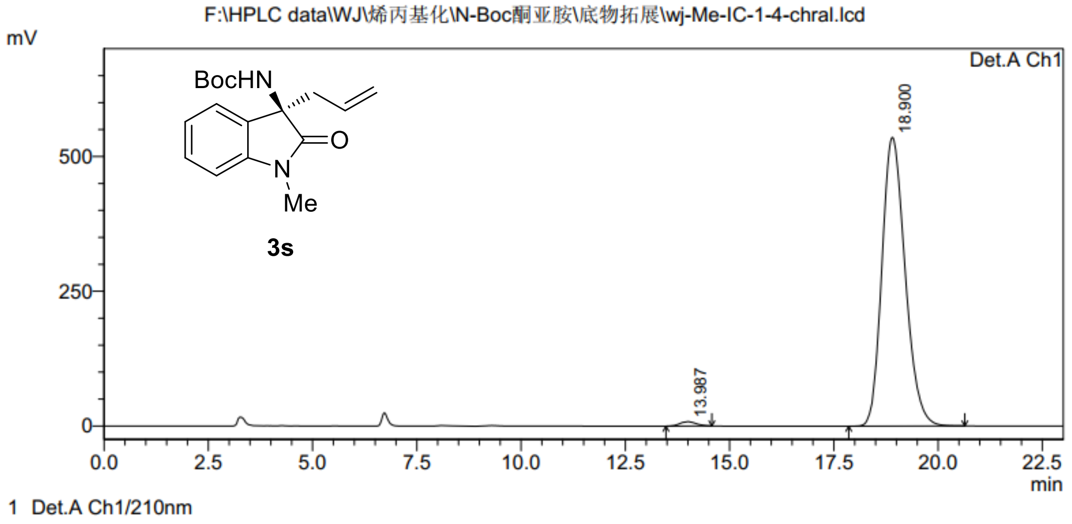

PeakTable

Detector A Ch1 210nm

Peak#	Ret. Time	Area	Height	Area %	Height %
1	13.987	221752	8251	1.057	1.517
2	18.900	20758482	535615	98.943	98.483
Total		20980234	543866	100.000	100.000

Figure S67. HPLC spectrum of **3s**, related to **Figure 2.**

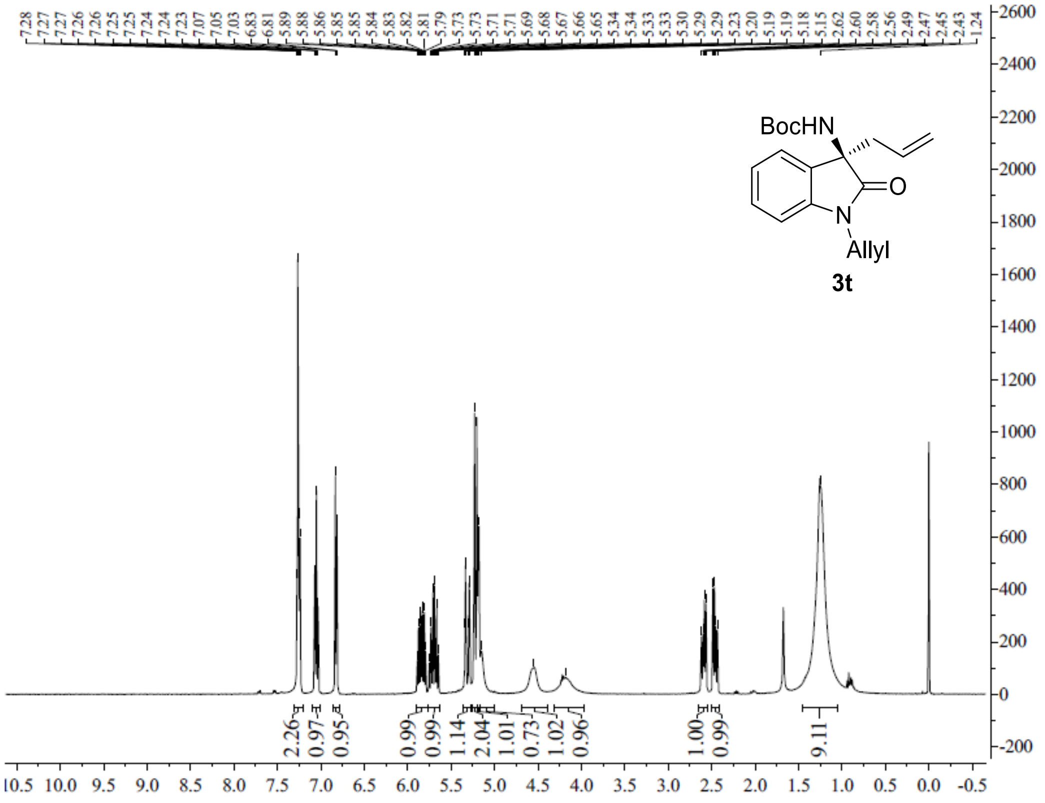

Figure S68. ^{1}H NMR spectrum of **3t**, related to **Figure 2**.

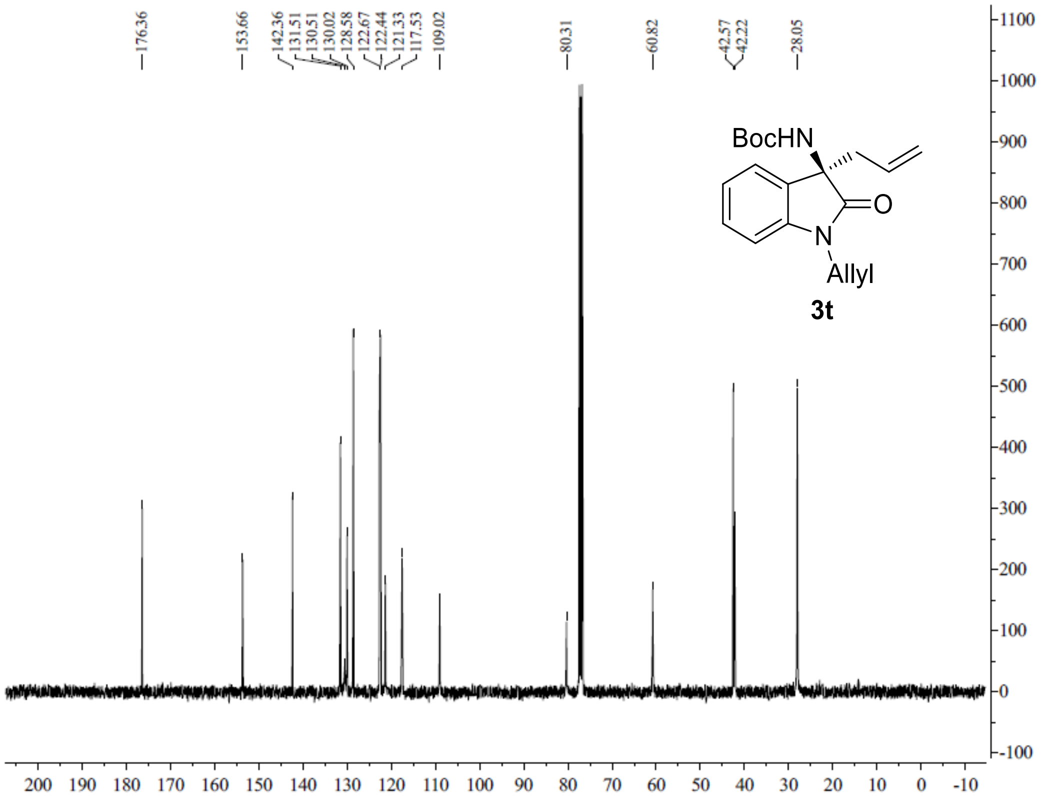

Figure S69. ^{13}C NMR spectrum of **3t**, related to **Figure 2.**

<Chromatogram>

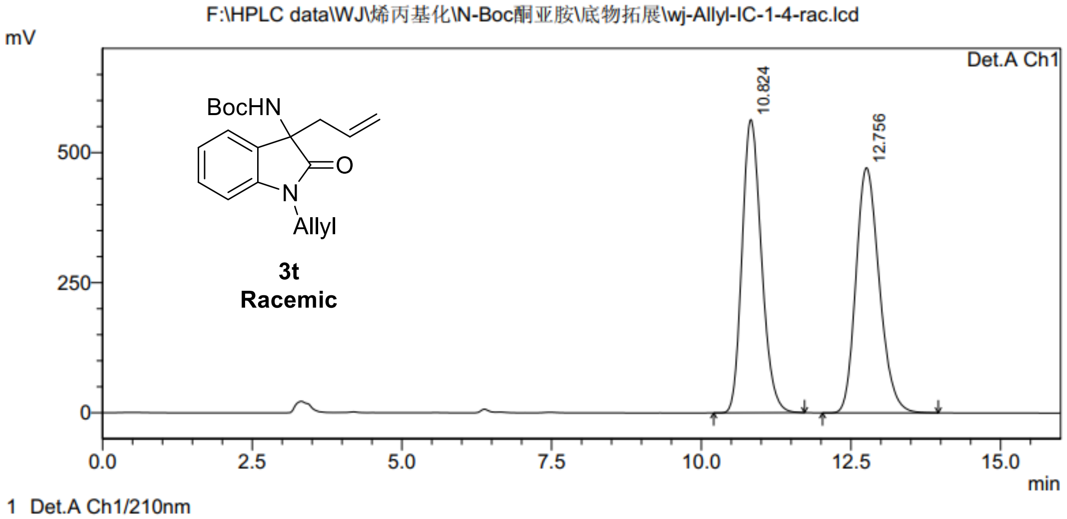

PeakTable

Detector A Ch1 210nm

Peak#	Ret. Time	Area	Height	Area %	Height %
1	10.824	12682484	563429	49.797	54.468
2	12.756	12786043	471000	50.203	45.532
Total		25468527	1034430	100.000	100.000

<Chromatogram>

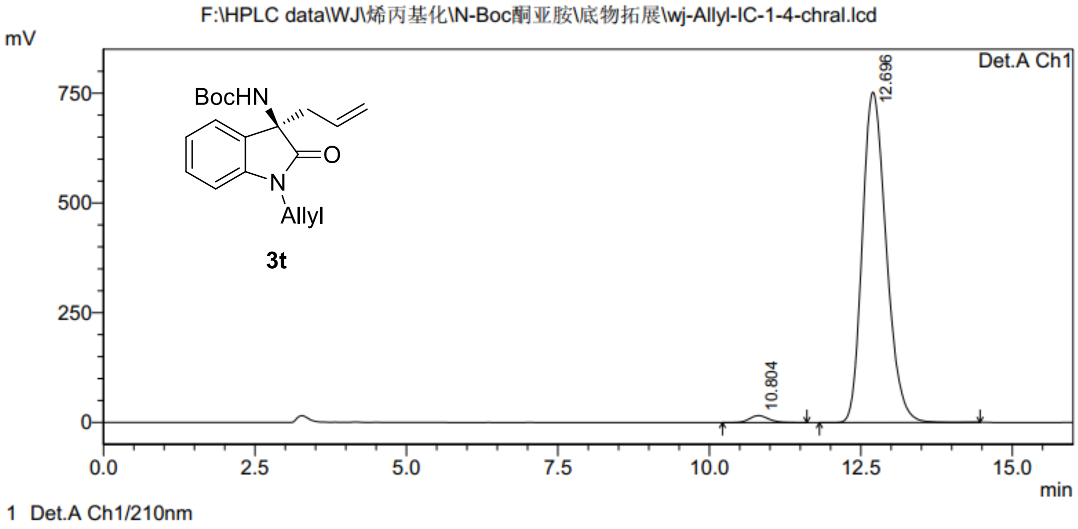

PeakTable

Detector A Ch1 210nm

Peak#	Ret. Time	Area	Height	Area %	Height %
1	10.804	346412	15870	1.648	2.065
2	12.696	20678600	752587	98.352	97.935
Total		21025012	768457	100.000	100.000

Figure S70. HPLC spectrum of **3t**, related to **Figure 2.**

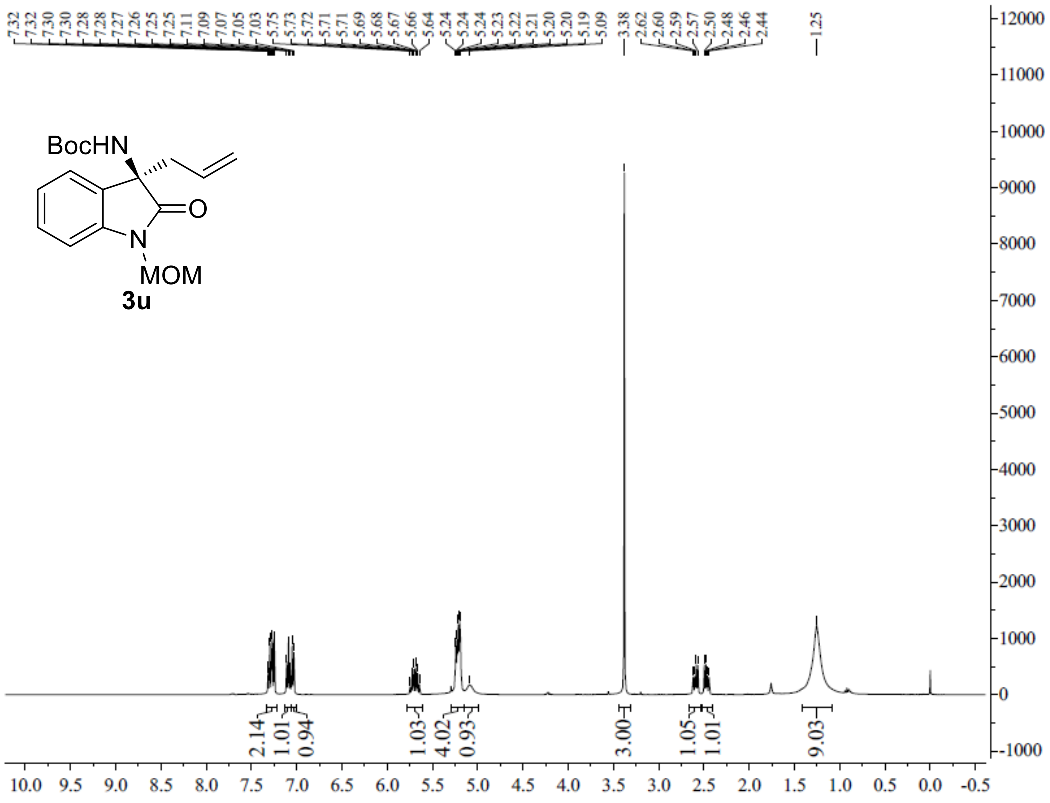

Figure S71. ^{1}H NMR spectrum of **3**u, related to **Figure 2**.

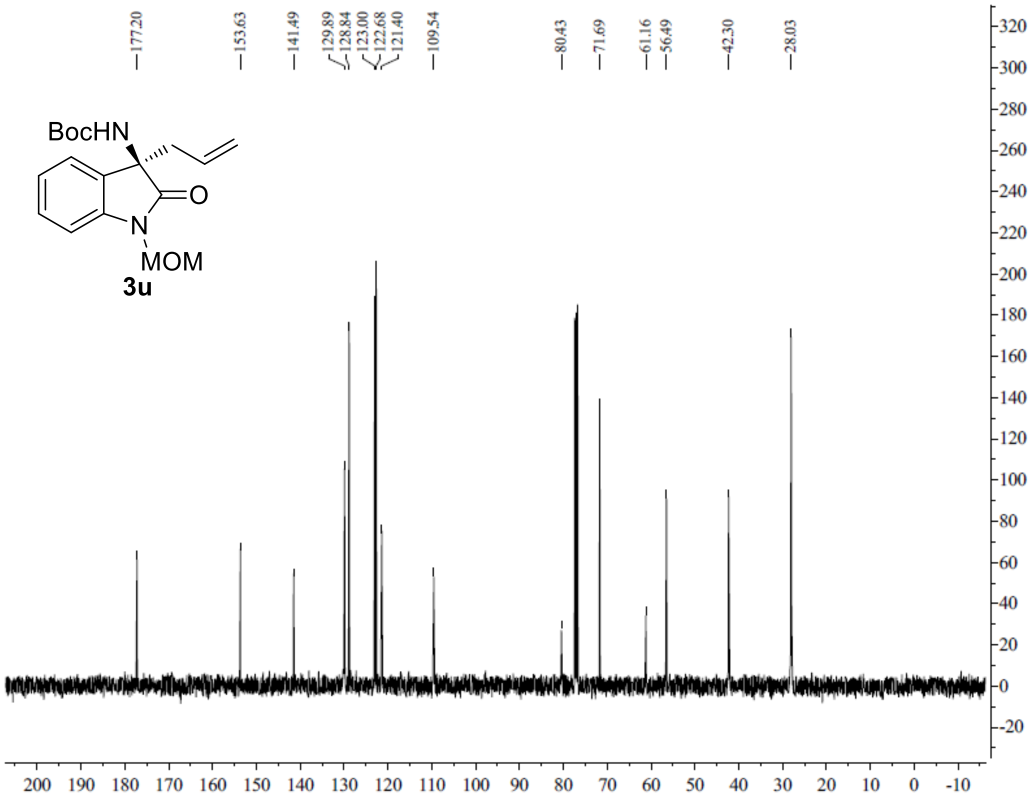

Figure S72. ^{13}C NMR spectrum of **3u**, related to **Figure 2.**

<Chromatogram>

F:\HPLC data\WJ\烯丙基化\N-Boc酮亚胺\底物拓展\wj-MOM-AD-1-4-rac.lcd

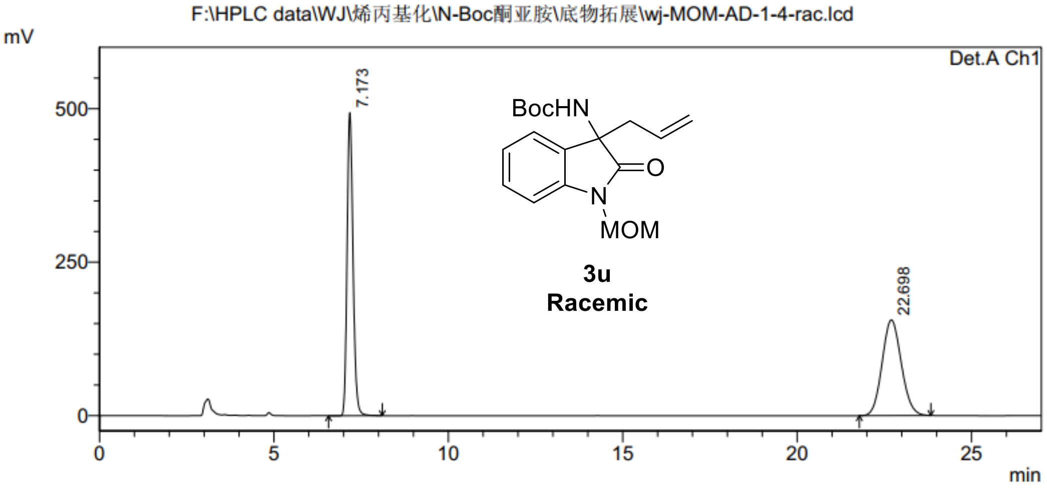

1 Det.A Ch1/210nm

PeakTable

Detector A Ch1 210nm

Peak#	Ret. Time	Area	Height	Area %	Height %
1	7.173	5828639	493506	49.414	75.986
2	22.698	5966816	155963	50.586	24.014
Total		11795455	649469	100.000	100.000

<Chromatogram>

F:\HPLC data\WJ\烯丙基化\N-Boc酮亚胺\底物拓展\wj-MOM-AD-1-4-chral.lcd

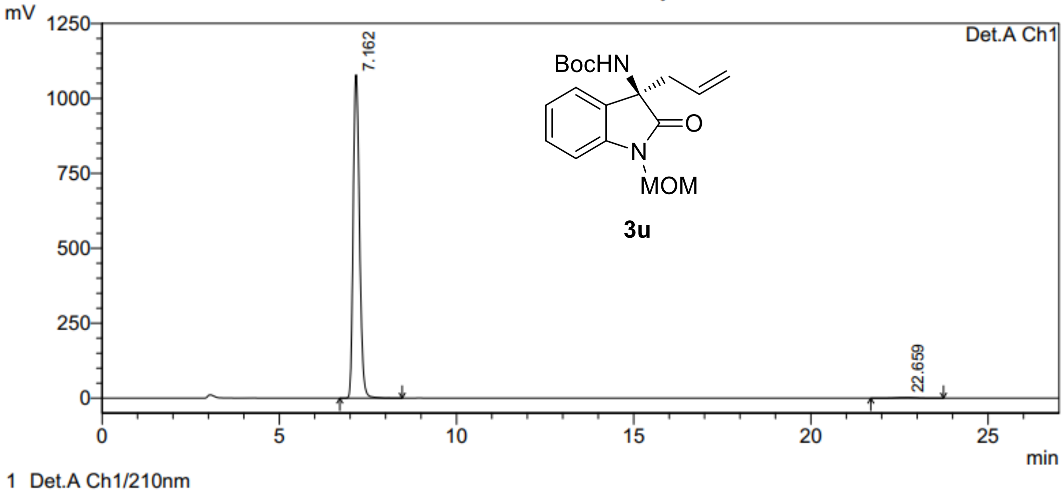

1 Det.A Ch1/210nm

PeakTable

Detector A Ch1 210nm

Peak#	Ret. Time	Area	Height	Area %	Height %
1	7.162	13336097	1078850	99.334	99.784
2	22.659	89412	2341	0.666	0.216
Total		13425509	1081191	100.000	100.000

Figure S73. HPLC spectrum of **3u**, related to **Figure 2.**

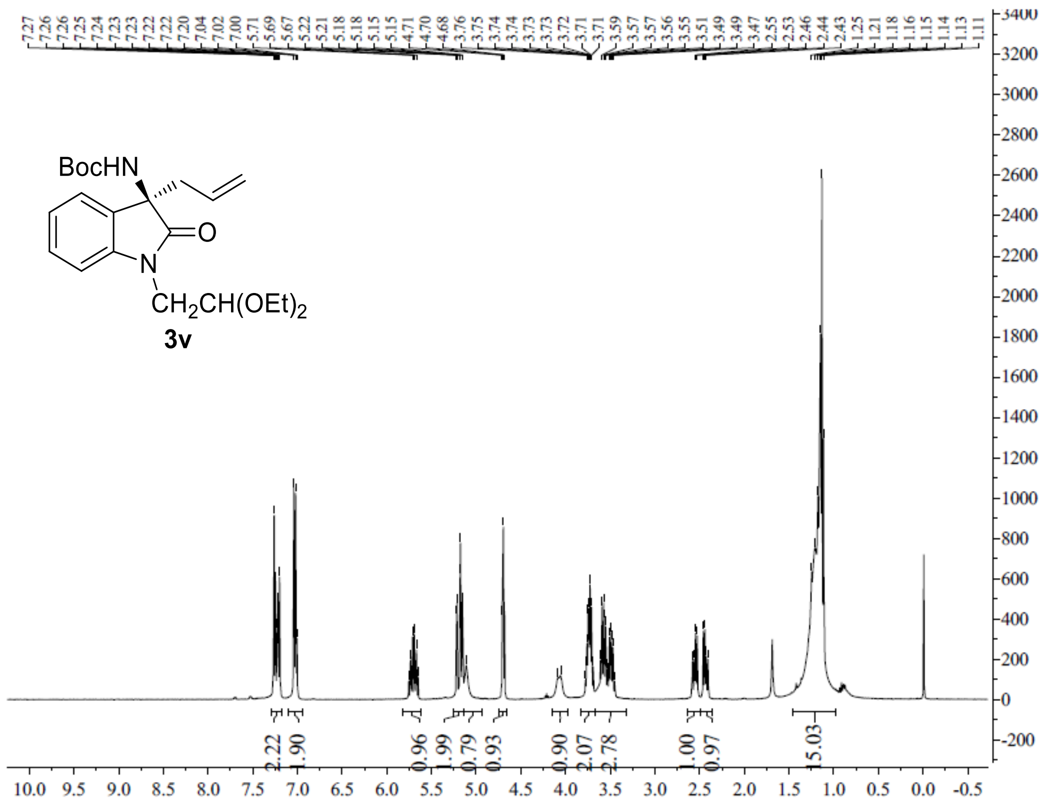

Figure S74. ^{1}H NMR spectrum of **3v**, related to **Figure 2**.

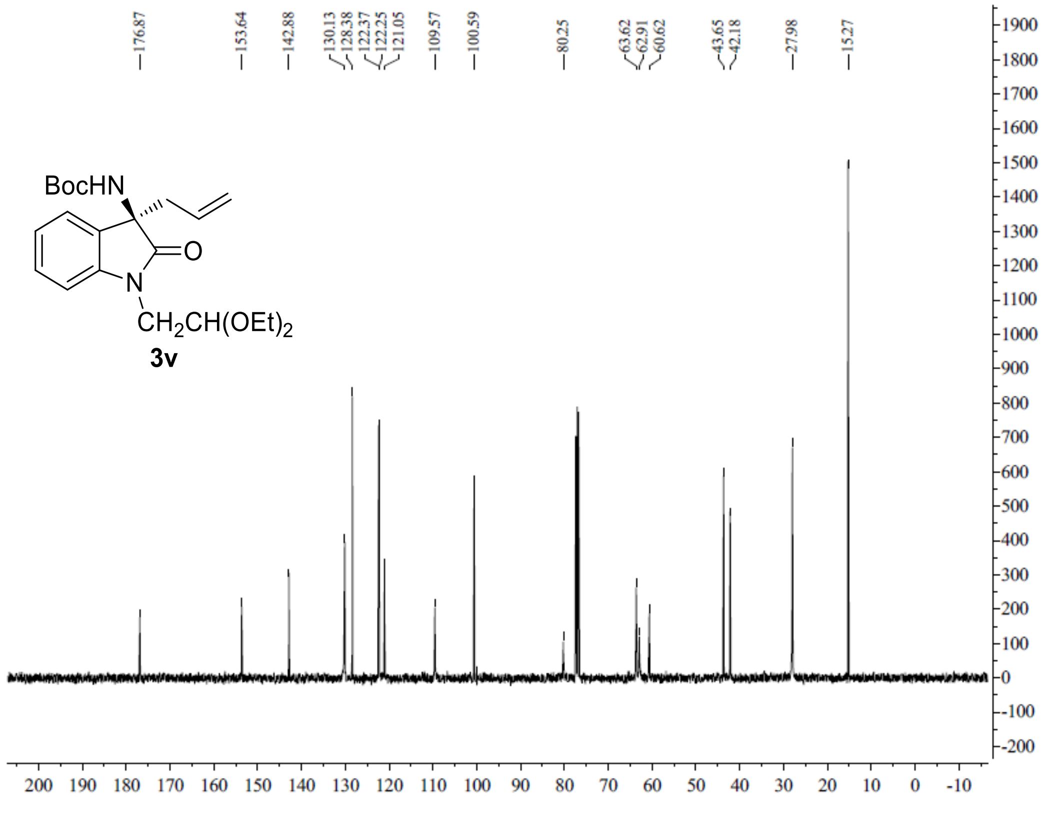

Figure S75. ^{13}C NMR spectrum of **3v**, related to **Figure 2.**

<Chromatogram>

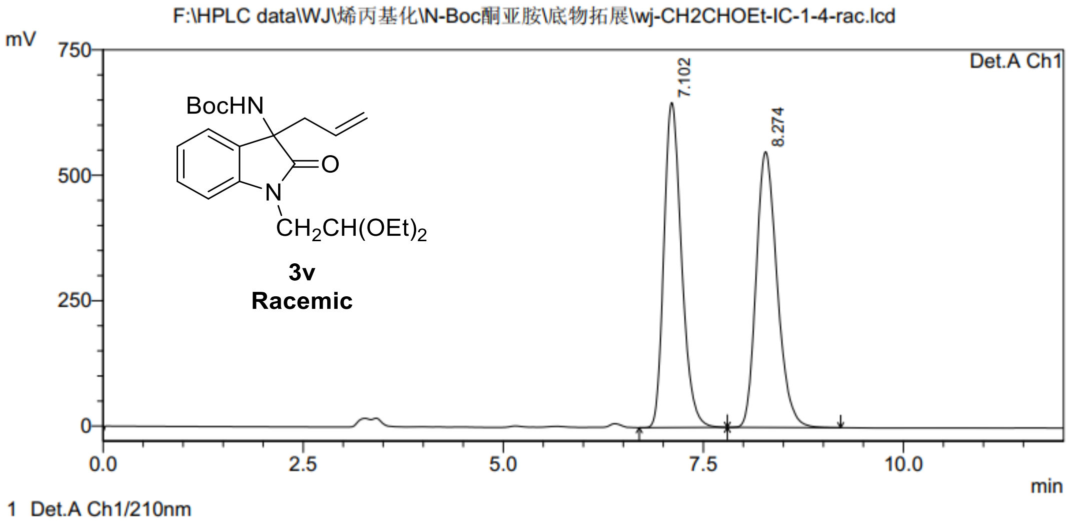

PeakTable

Detector A Ch1 210nm

Peak#	Ret. Time	Area	Height	Area %	Height %
1	7.102	9919474	647829	49.778	54.105
2	8.274	10008063	549530	50.222	45.895
Total		19927537	1197358	100.000	100.000

<Chromatogram>

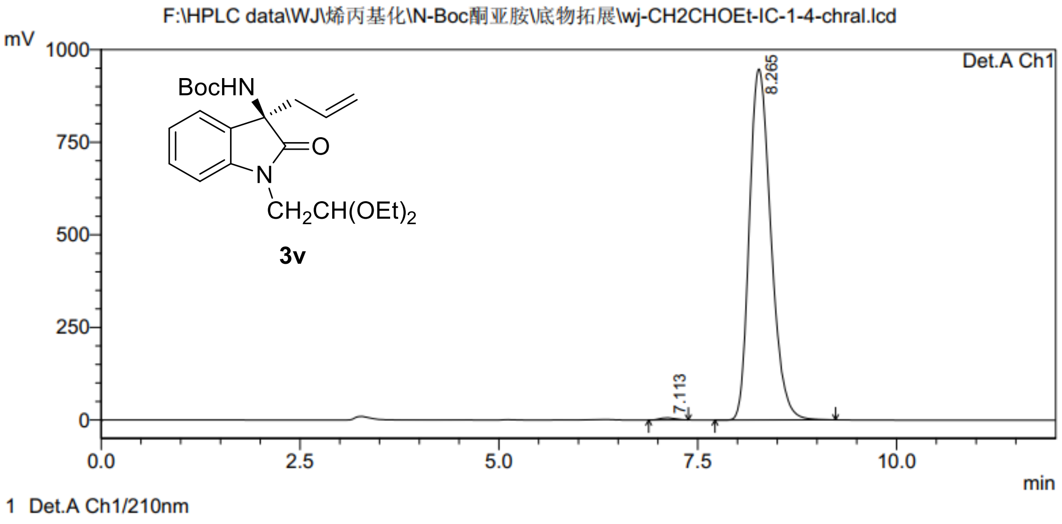

PeakTable

Detector A Ch1 210nm

Peak#	Ret. Time	Area	Height	Area %	Height %
1	7.113	81629	6009	0.456	0.630
2	8.265	17833920	947988	99.544	99.370
Total		17915549	953997	100.000	100.000

Figure S76. HPLC spectrum of **3v**, related to **Figure 2.**

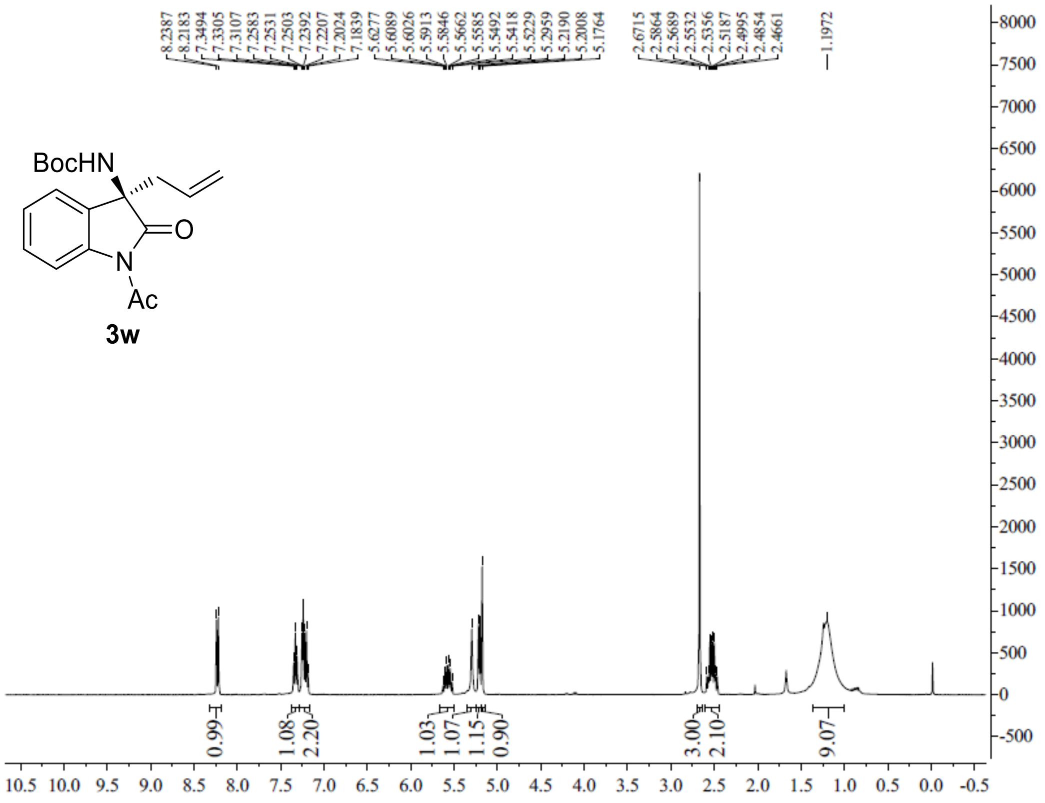

Figure S77. ^{1}H NMR spectrum of **3w**, related to **Figure 2**.

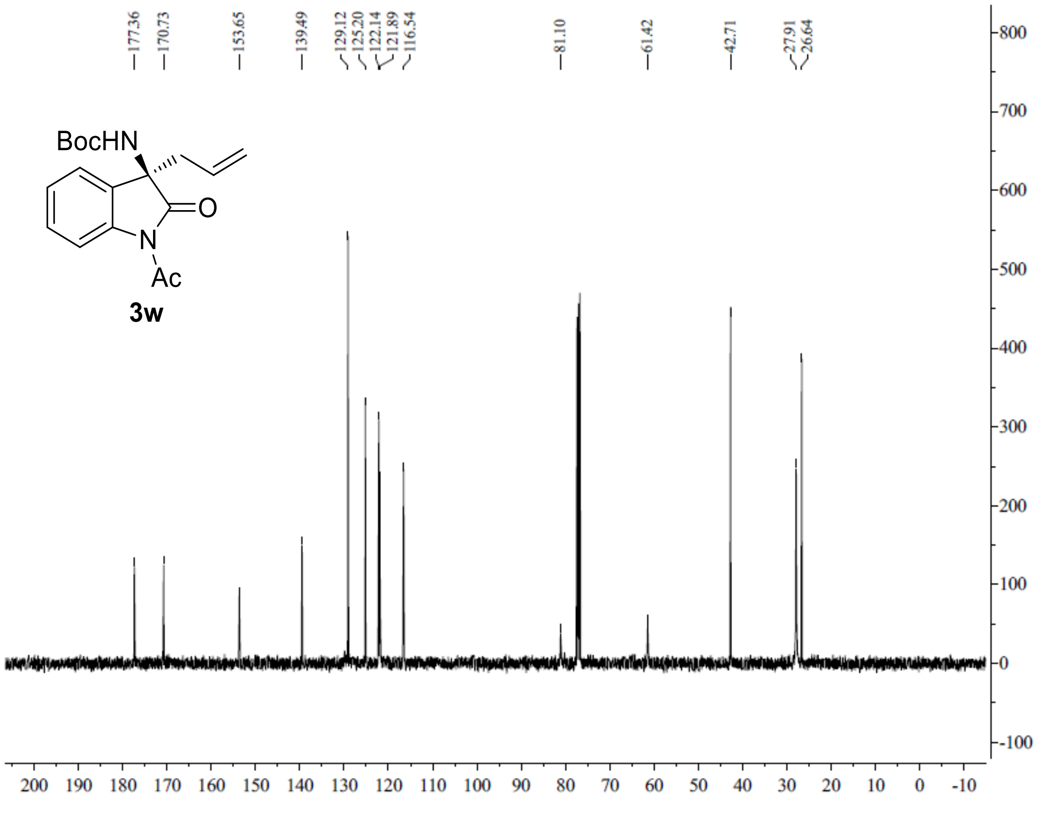

Figure S78. ^{13}C NMR spectrum of **3w**, related to **Figure 2.**

<Chromatogram>

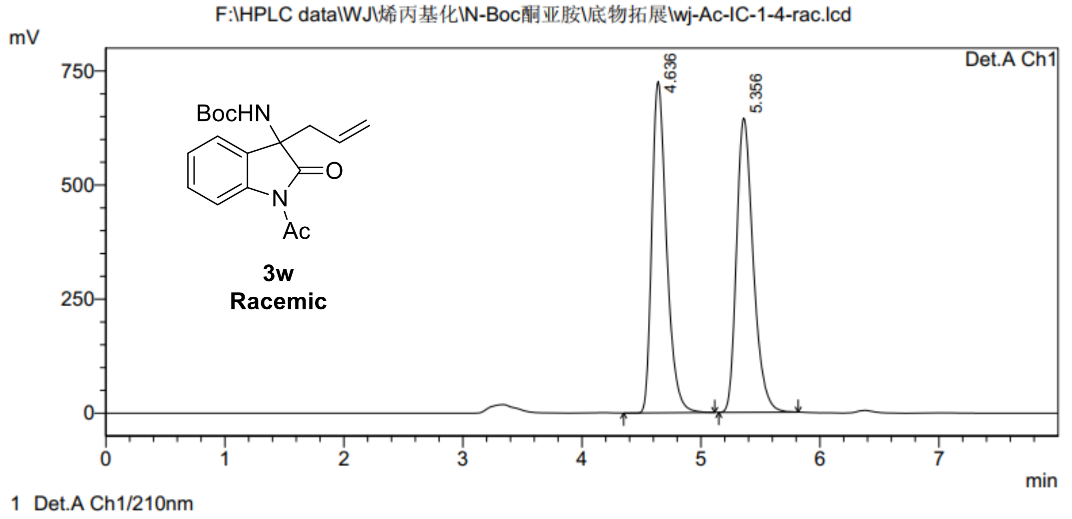

PeakTable

Detector A Ch1 210nm

Peak#	Ret. Time	Area	Height	Area %	Height %
1	4.636	6230155	726068	49.781	52.957
2	5.356	6284933	644990	50.219	47.043
Total		12515088	1371059	100.000	100.000

<Chromatogram>

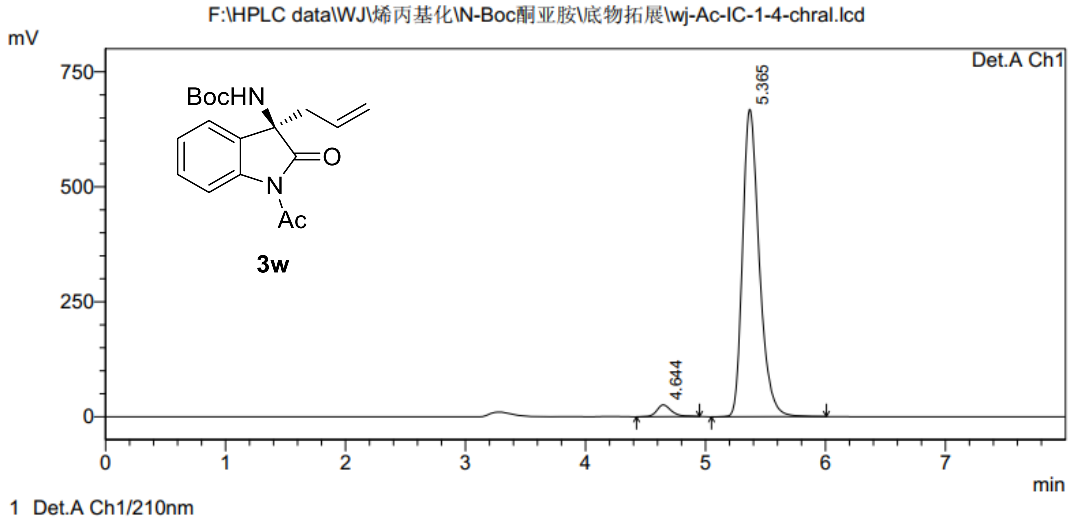

PeakTable

Detector A Ch1 210nm

Peak#	Ret. Time	Area	Height	Area %	Height %
1	4.644	217703	25691	3.269	3.701
2	5.365	6441109	668445	96.731	96.299
Total		6658812	694136	100.000	100.000

Figure S79. HPLC spectrum of **3w**, related to **Figure 2.**

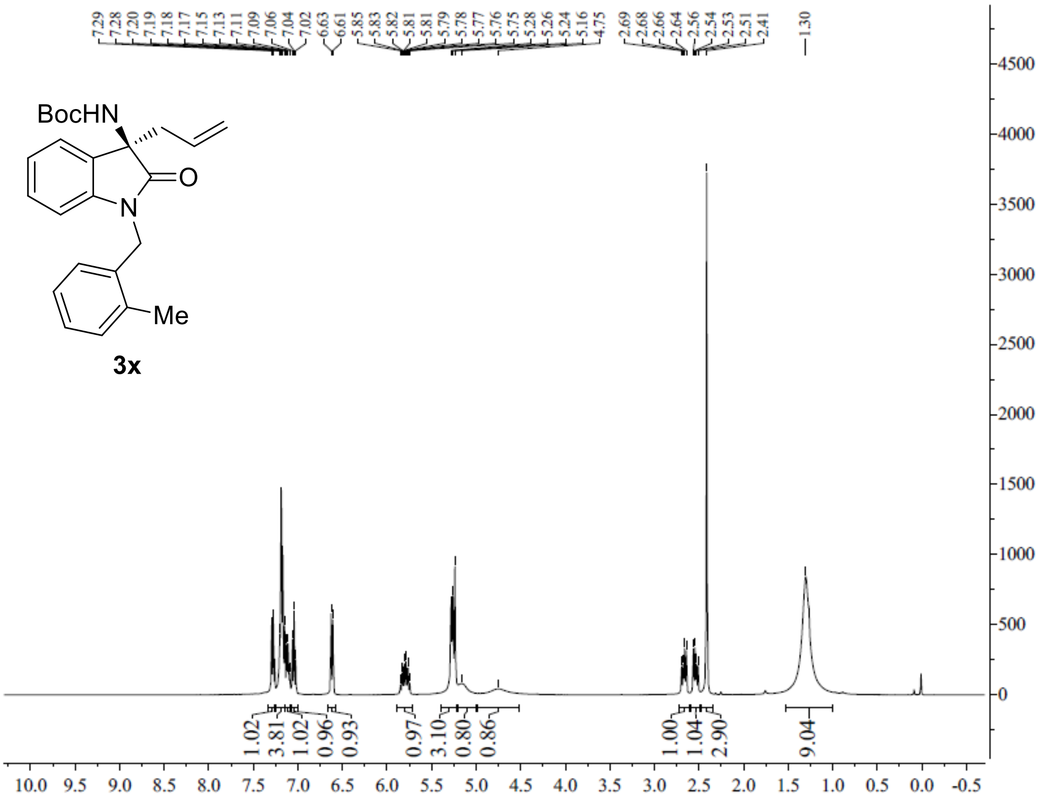

Figure S80. ^{1}H NMR spectrum of **3x**, related to **Figure 2**.

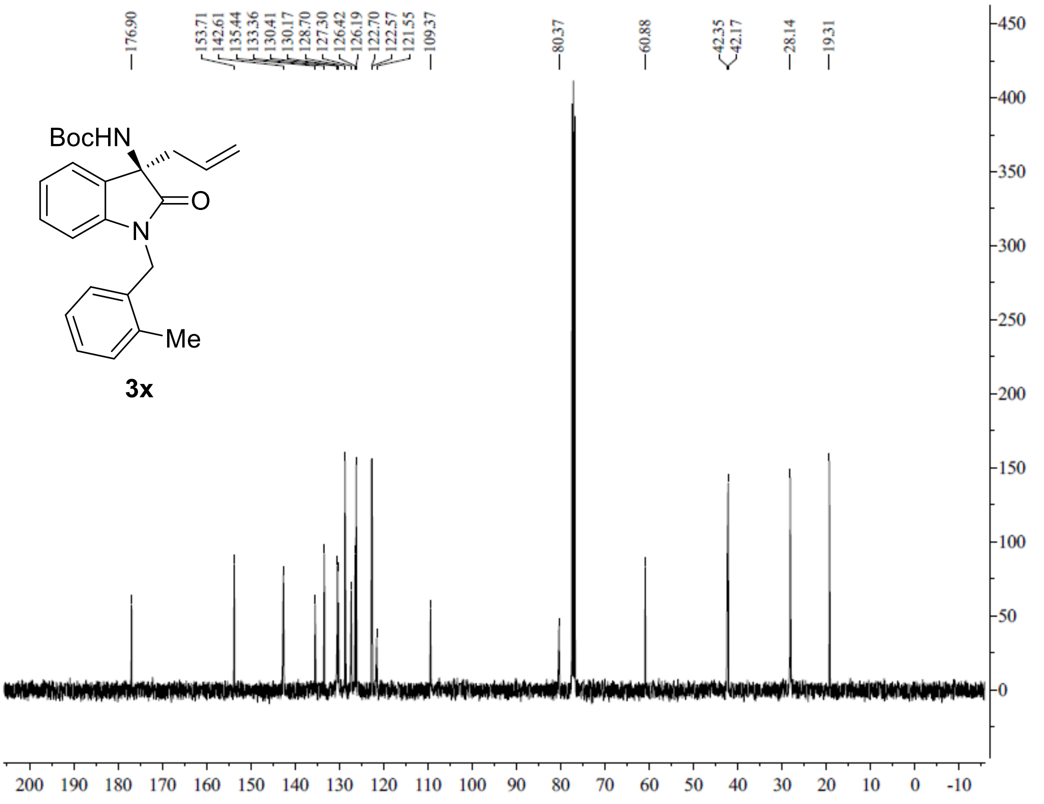

Figure S81. ^{13}C NMR spectrum of **3x**, related to **Figure 2.**

<Chromatogram>

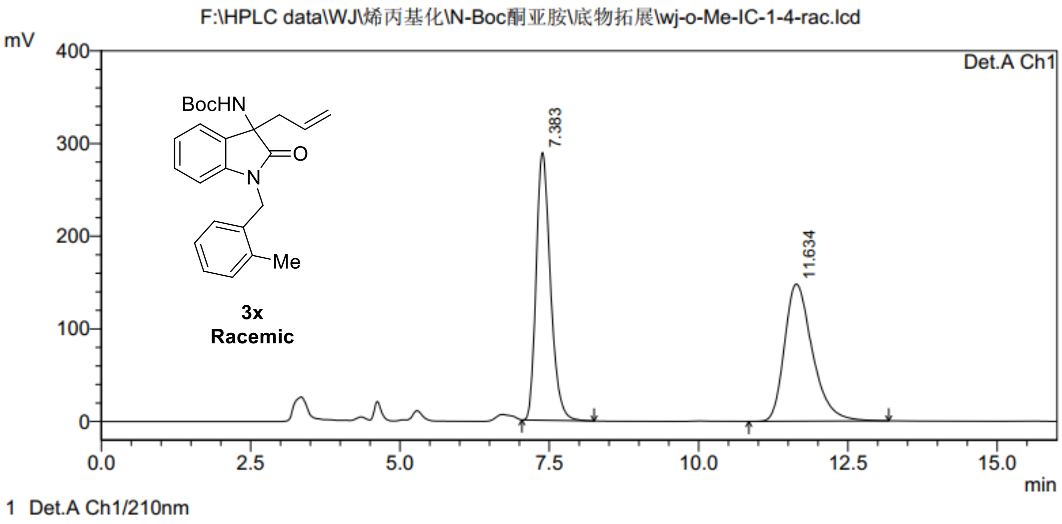

PeakTable

Detector A Ch1 210nm

Peak#	Ret. Time	Area	Height	Area %	Height %
1	7.383	4784449	288876	49.810	66.096
2	11.634	4820868	148178	50.190	33.904
Total		9605317	437054	100.000	100.000

<Chromatogram>

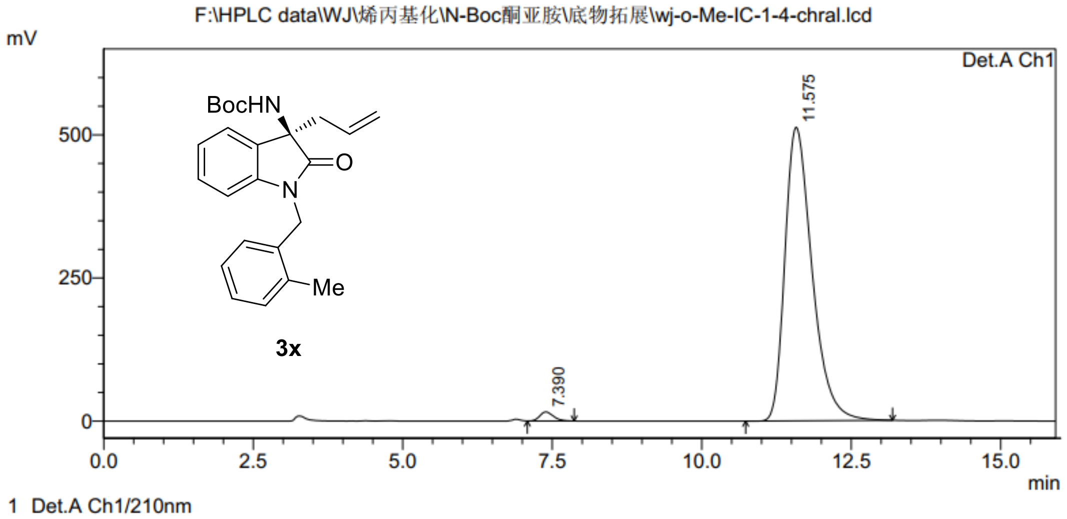

PeakTable

Detector A Ch1 210nm

Peak#	Ret. Time	Area	Height	Area %	Height %
1	7.390	249612	15853	1.518	2.999
2	11.575	16191604	512813	98.482	97.001
Total		16441216	528666	100.000	100.000

Figure S82. HPLC spectrum of **3x**, related to **Figure 2.**

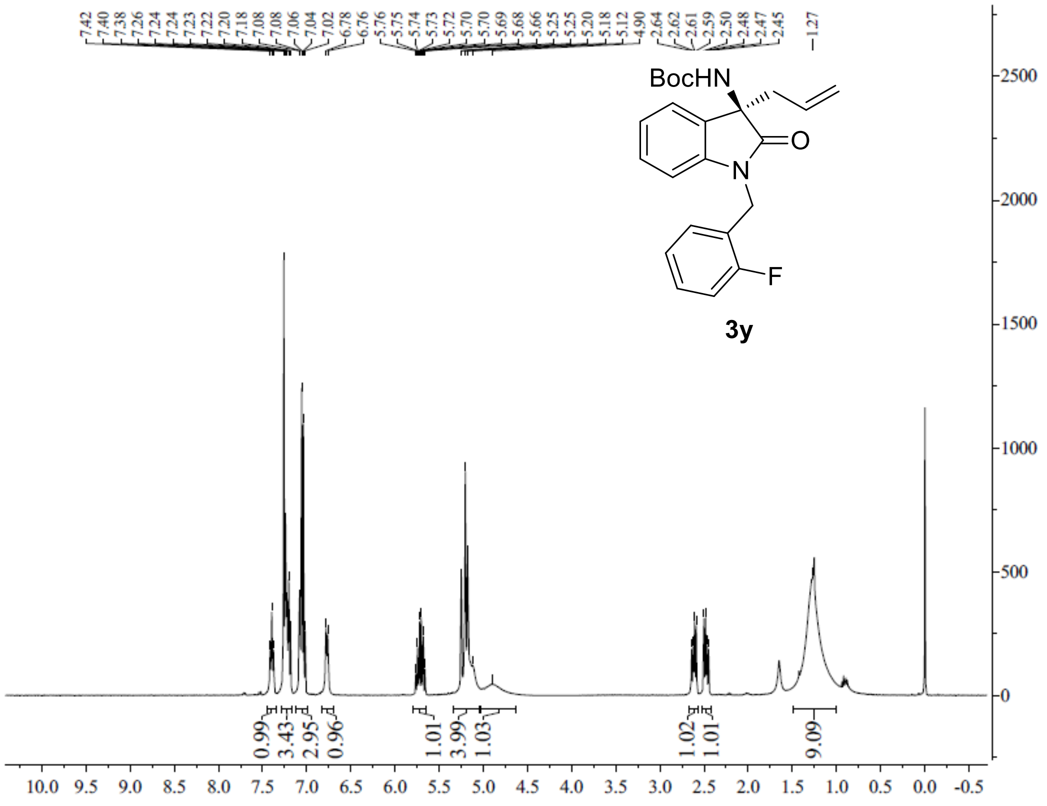

Figure S83. ^{1}H NMR spectrum of **3y**, related to **Figure 2**.

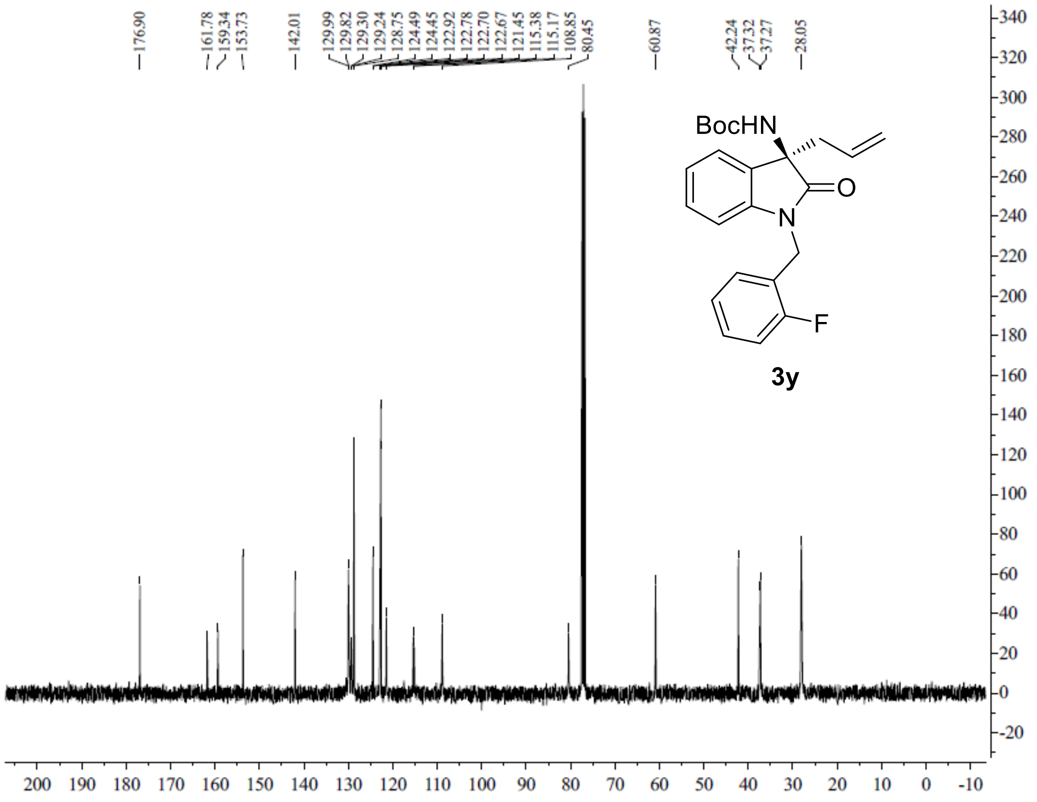

Figure S84. ^{13}C NMR spectrum of **3y**, related to **Figure 2.**

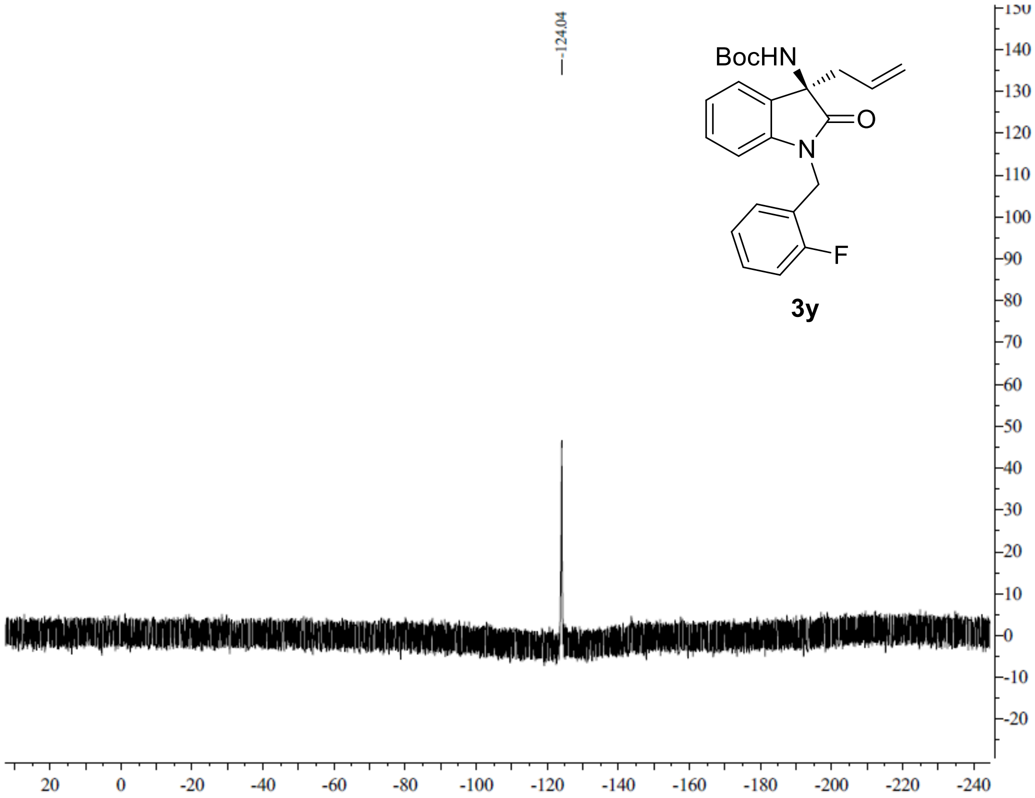

Figure S85. ^{19}F NMR spectrum of **3y**, related to **Figure 2.**

<Chromatogram>

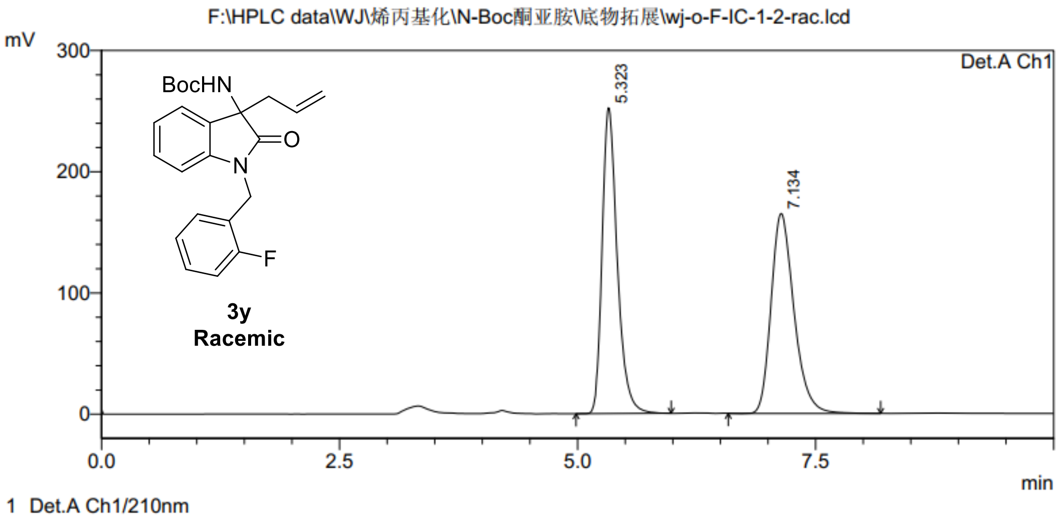

PeakTable

Detector A Ch1 210nm

Peak#	Ret. Time	Area	Height	Area %	Height %
1	5.323	2784700	252323	50.198	60.475
2	7.134	2762738	164910	49.802	39.525
Total		5547438	417233	100.000	100.000

<Chromatogram>

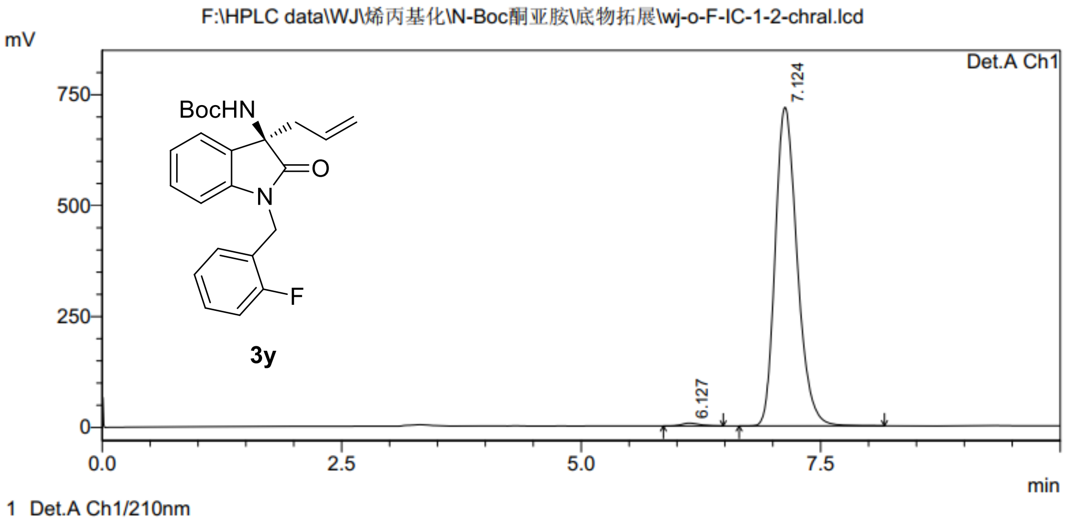

PeakTable

Detector A Ch1 210nm

Peak#	Ret. Time	Area	Height	Area %	Height %
1	6.127	79306	6231	0.689	0.860
2	7.124	11428048	718313	99.311	99.140
Total		11507355	724545	100.000	100.000

Figure S86. HPLC spectrum of **3y**, related to **Figure 2.**

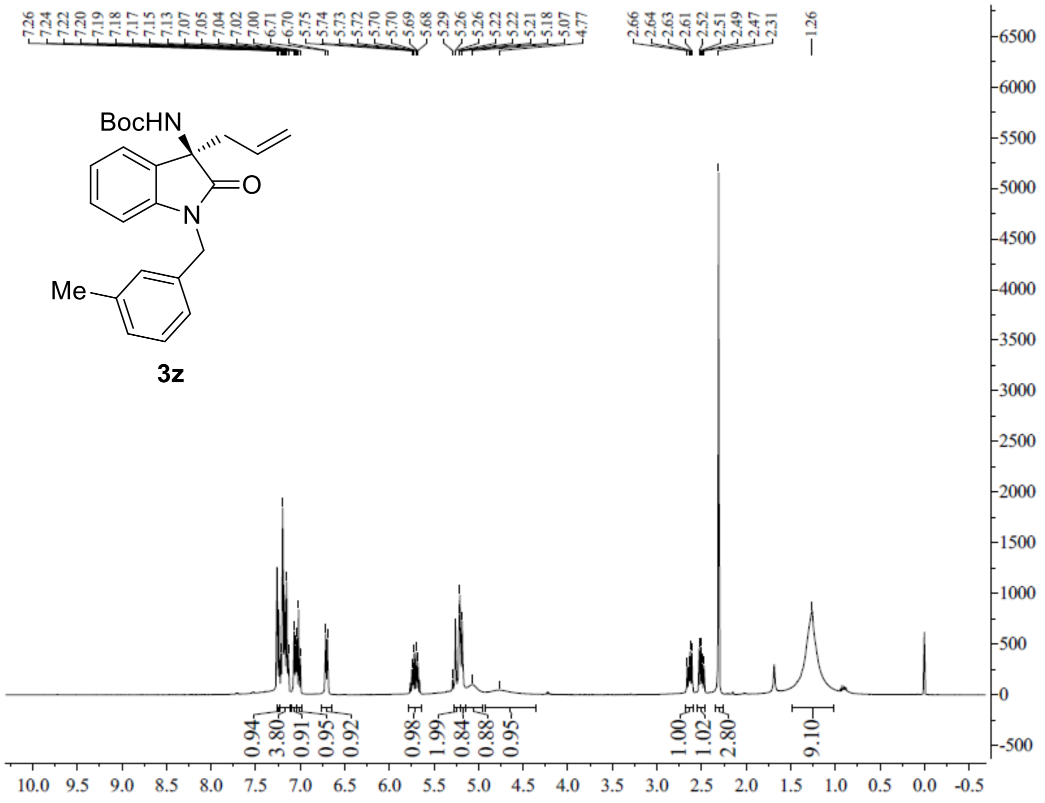

Figure S87. ^{1}H NMR spectrum of **3z**, related to **Figure 2**.

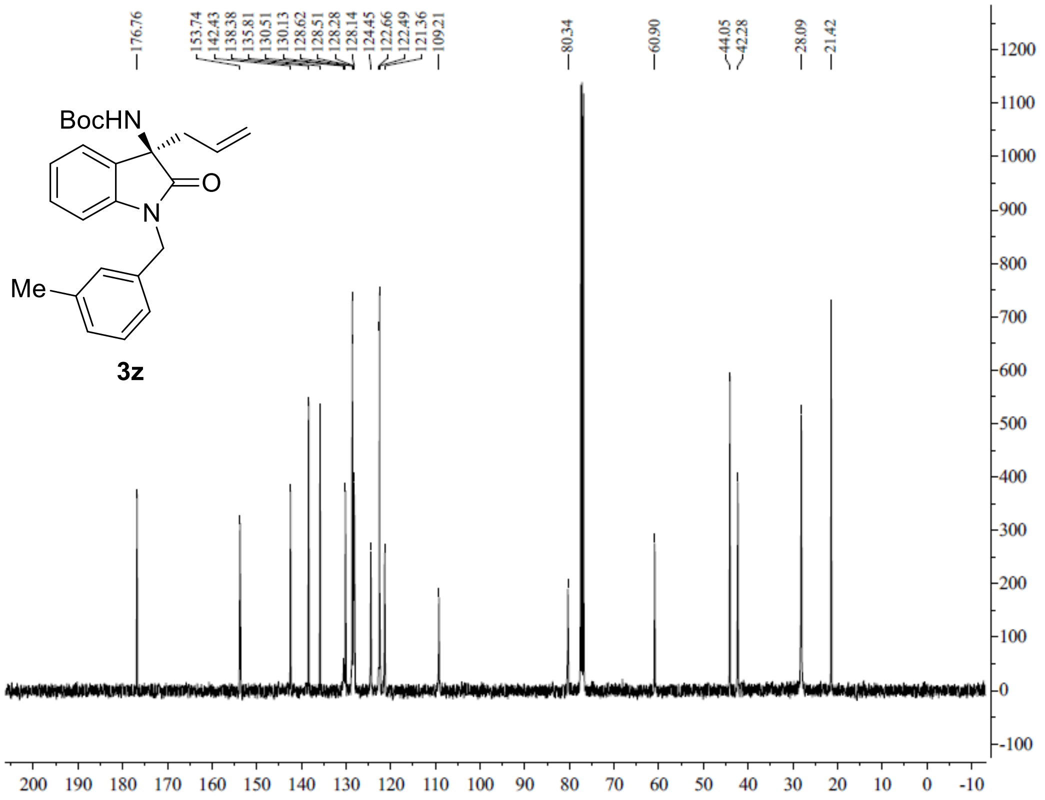

Figure S88. ^{13}C NMR spectrum of **3z**, related to **Figure 2.**

<Chromatogram>

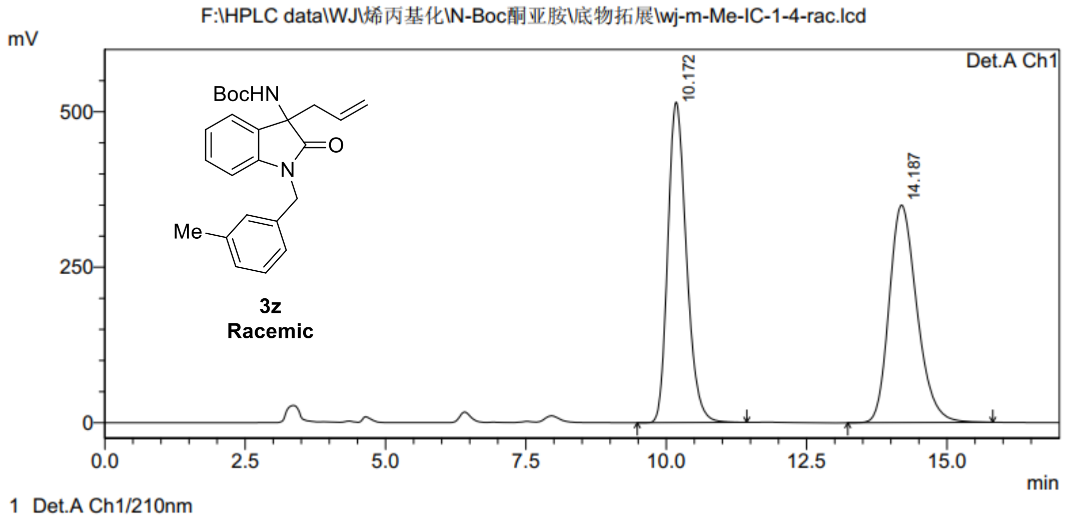

PeakTable

Detector A Ch1 210nm

Peak#	Ret. Time	Area	Height	Area %	Height %
1	10.172	11873752	515300	49.672	59.574
2	14.187	12030440	349670	50.328	40.426
Total		23904192	864970	100.000	100.000

<Chromatogram>

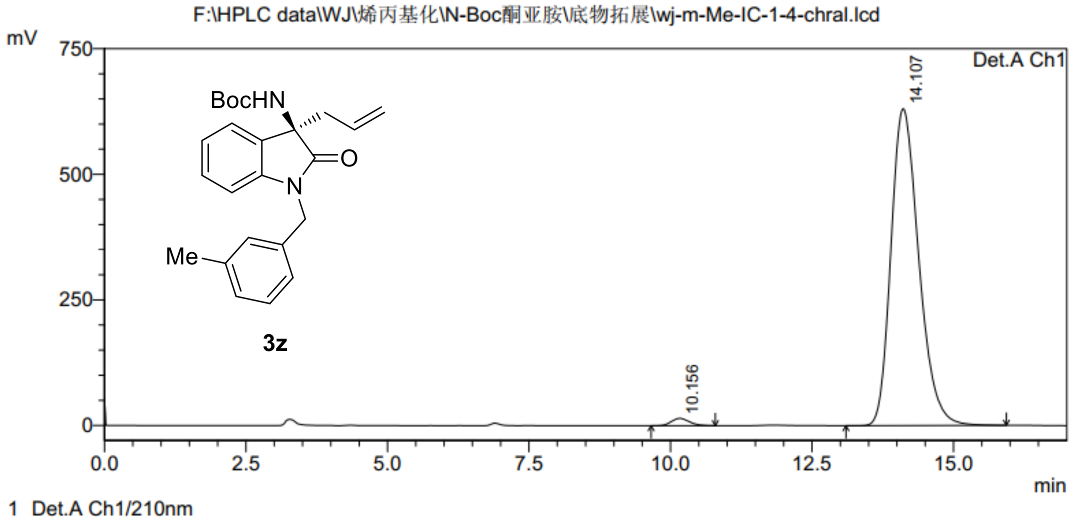

PeakTable

Detector A Ch1 210nm

Peak#	Ret. Time	Area	Height	Area %	Height %
1	10.156	324420	14664	1.481	2.272
2	14.107	21579926	630645	98.519	97.728
Total		21904346	645309	100.000	100.000

Figure S89. HPLC spectrum of **3z**, related to **Figure 2.**

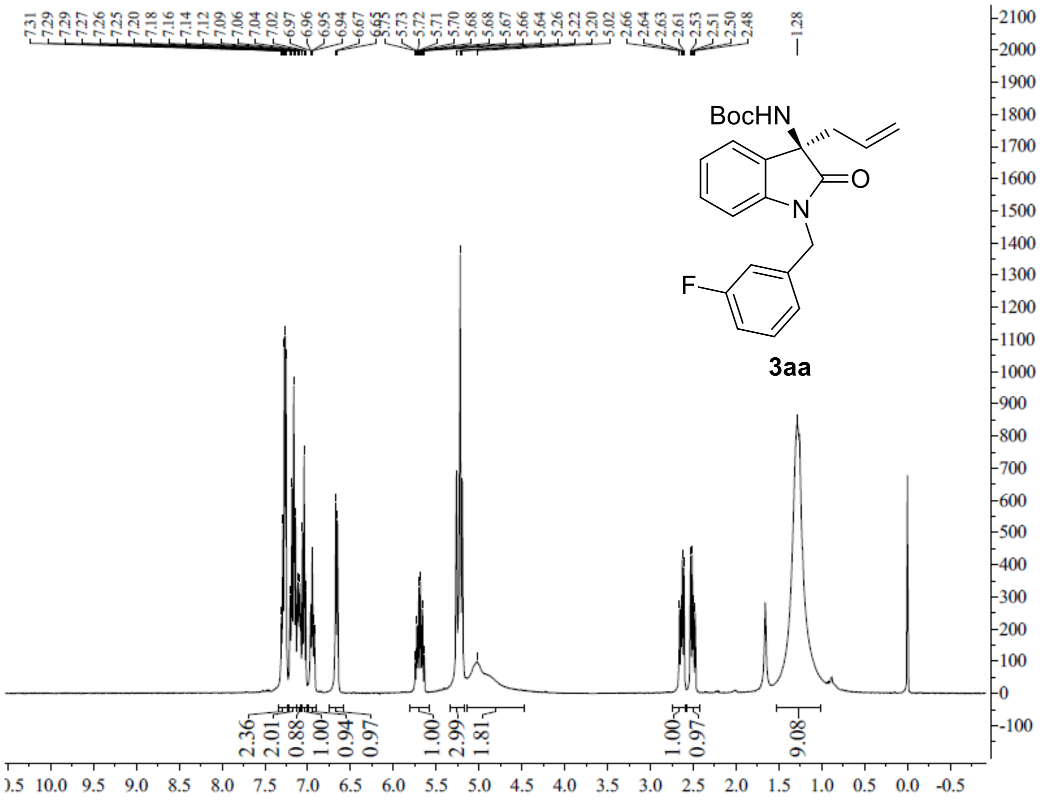

Figure S90. ^{1}H NMR spectrum of **3**aa, related to **Figure 2**.

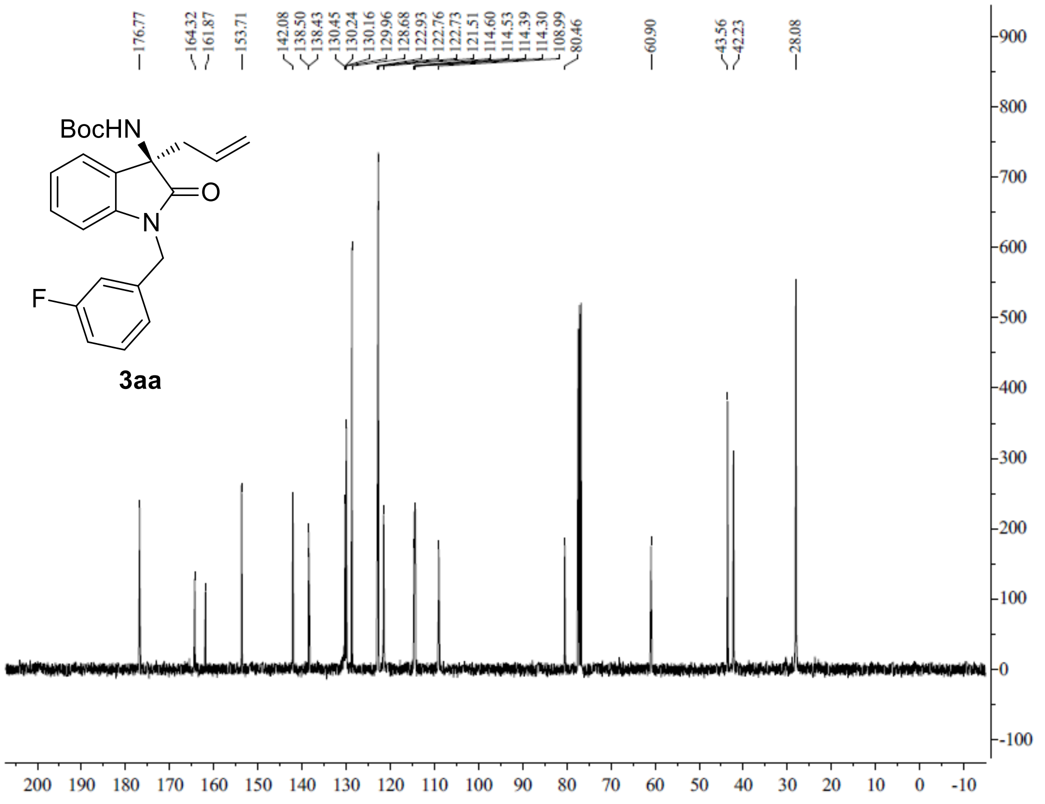

Figure S91. ^{13}C NMR spectrum of **3aa**, related to **Figure 2.**

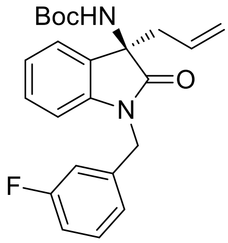

3aa

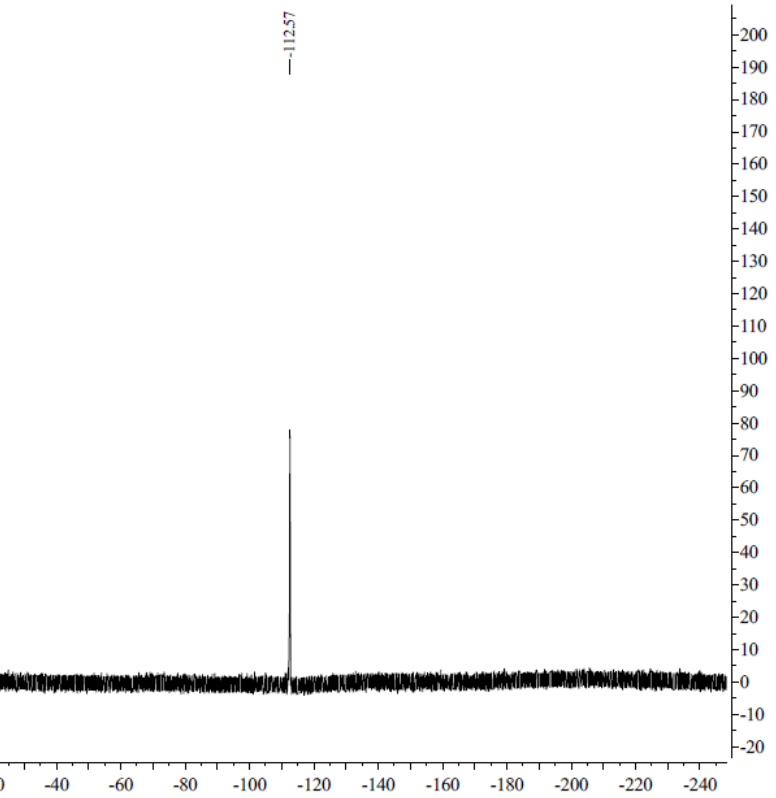

Figure S92. ^{19}F NMR spectrum of **3aa**, related to **Figure 2.**

<Chromatogram>

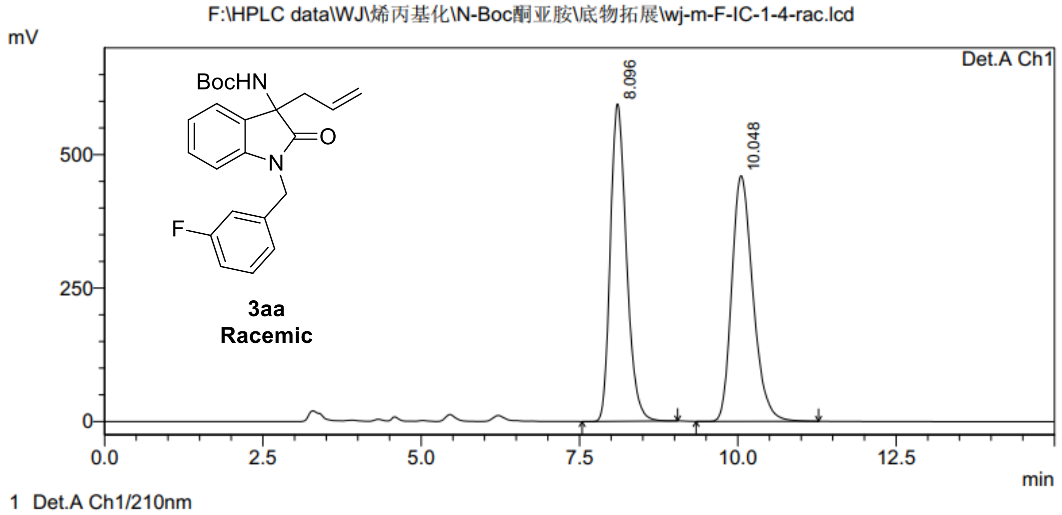

PeakTable

Detector A Ch1 210nm

Peak#	Ret. Time	Area	Height	Area %	Height %
1	8.096	10515271	594805	49.595	56.372
2	10.048	10687019	460340	50.405	43.628
Total		21202289	1055145	100.000	100.000

<Chromatogram>

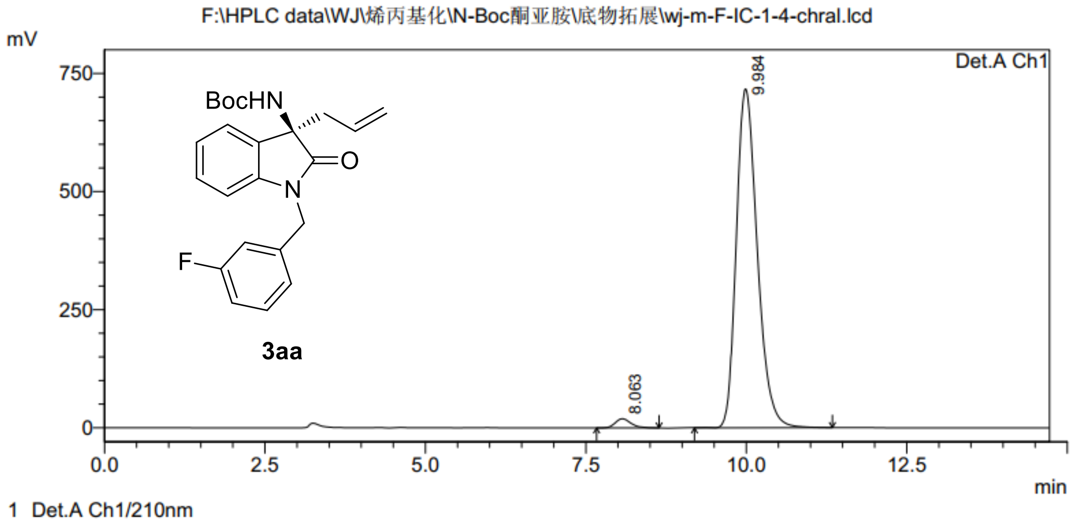

PeakTable

Detector A Ch1 210nm

Peak#	Ret. Time	Area	Height	Area %	Height %
1	8.063	323400	19443	1.944	2.639
2	9.984	16309376	717266	98.056	97.361
Total		16632776	736709	100.000	100.000

Figure S93. HPLC spectrum of **3aa**, related to **Figure 2.**

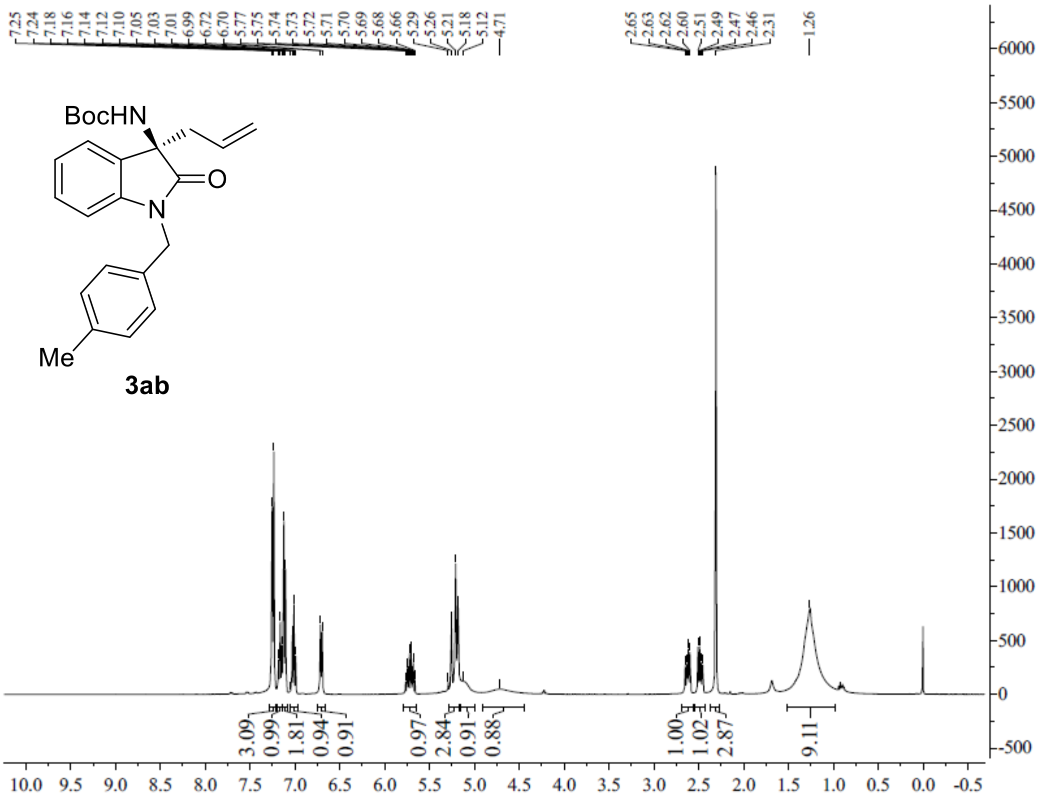

Figure S94. ^{1}H NMR spectrum of **3**ab, related to **Figure 2**.

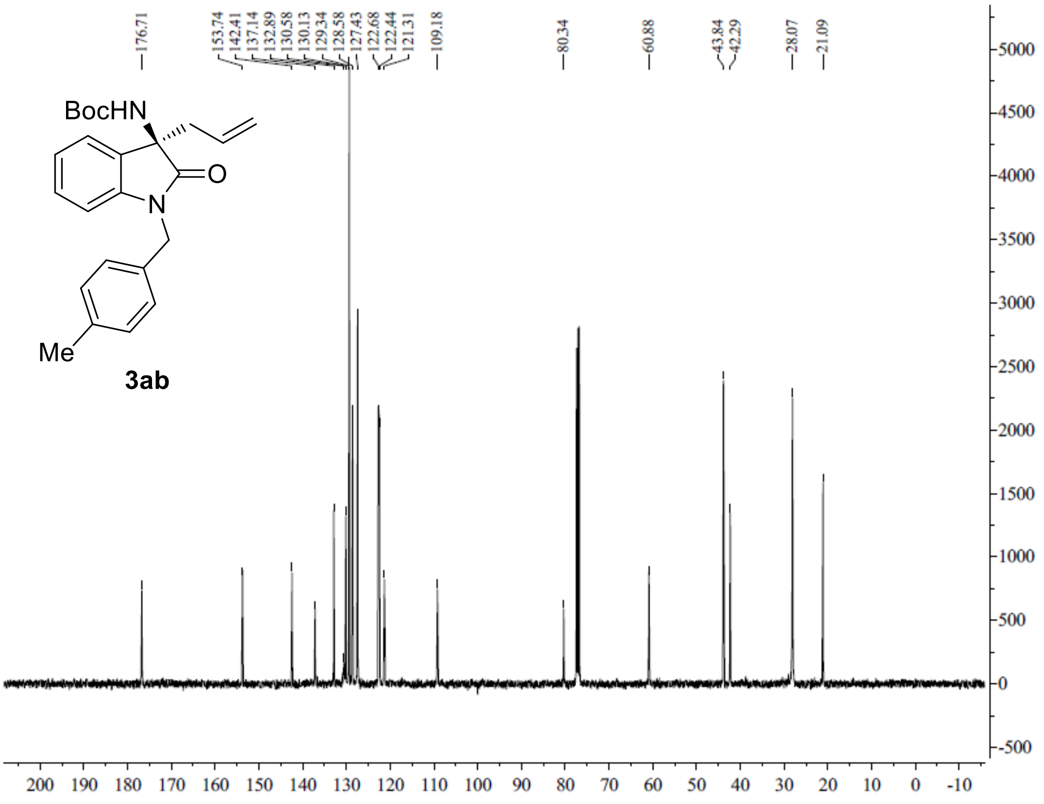

Figure S95. ^{13}C NMR spectrum of **3ab**, related to **Figure 2.**

<Chromatogram>

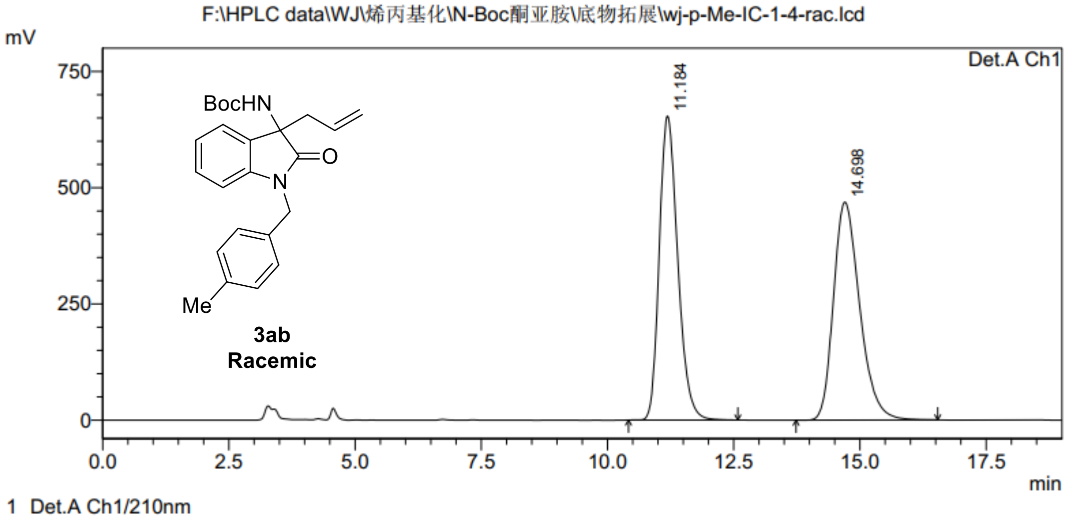

PeakTable

Detector A Ch1 210nm

Peak#	Ret. Time	Area	Height	Area %	Height %
1	11.184	16656677	653677	49.607	58.228
2	14.698	16920368	468932	50.393	41.772
Total		33577045	1122609	100.000	100.000

<Chromatogram>

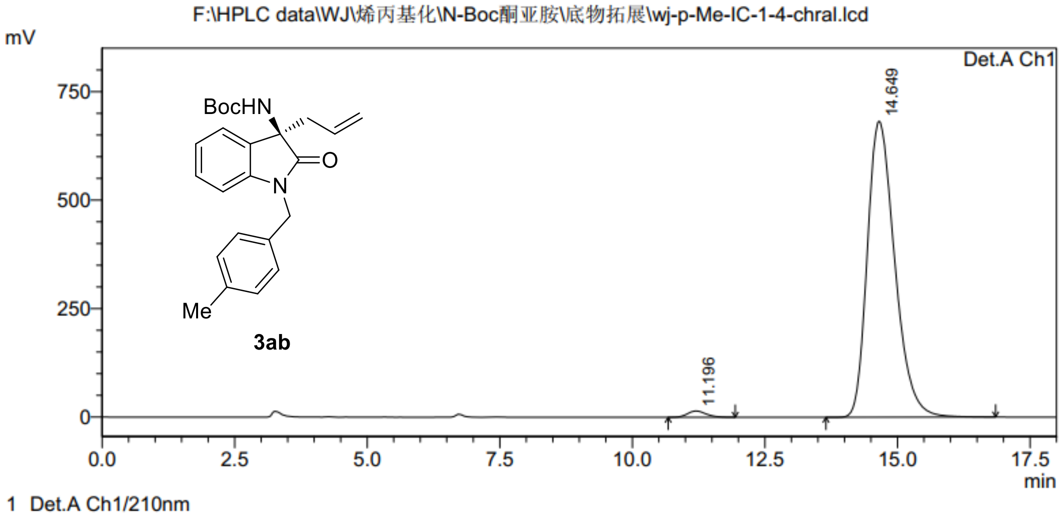

PeakTable

Detector A Ch1 210nm

Peak#	Ret. Time	Area	Height	Area %	Height %
1	11.196	352256	14434	1.413	2.073
2	14.649	24579474	682002	98.587	97.927
Total		24931730	696436	100.000	100.000

Figure S96. HPLC spectrum of **3ab**, related to **Figure 2.**

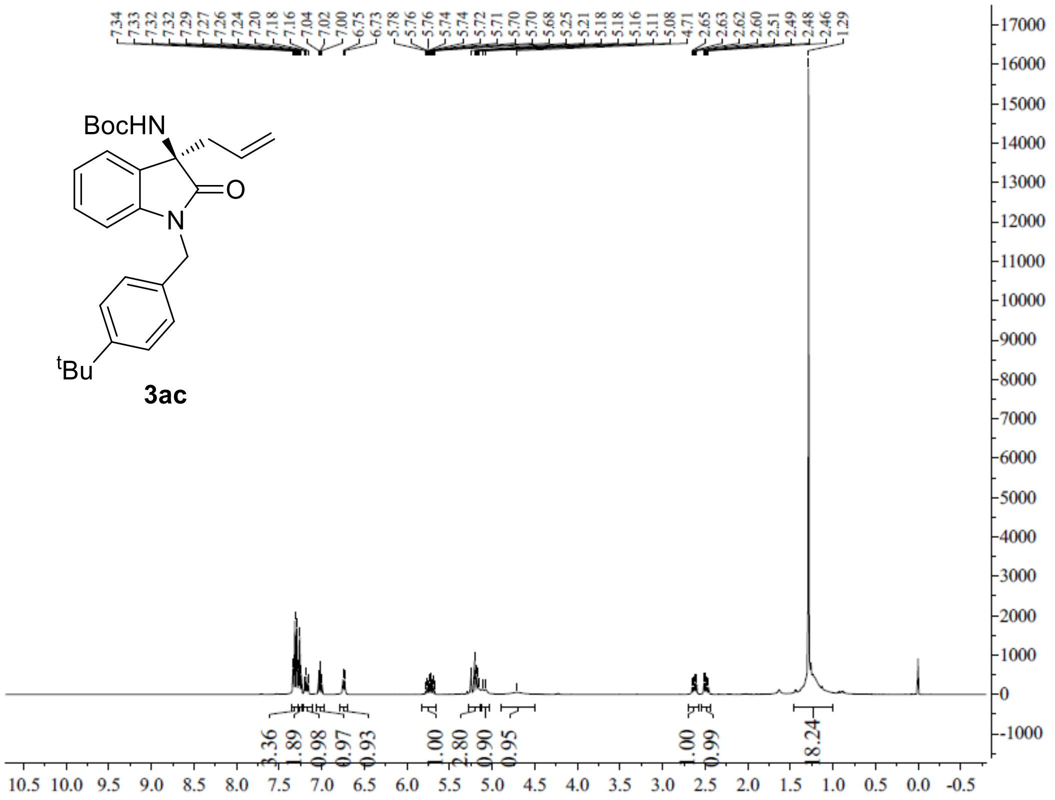

Figure S97. ^{1}H NMR spectrum of **3**ac, related to **Figure 2**.

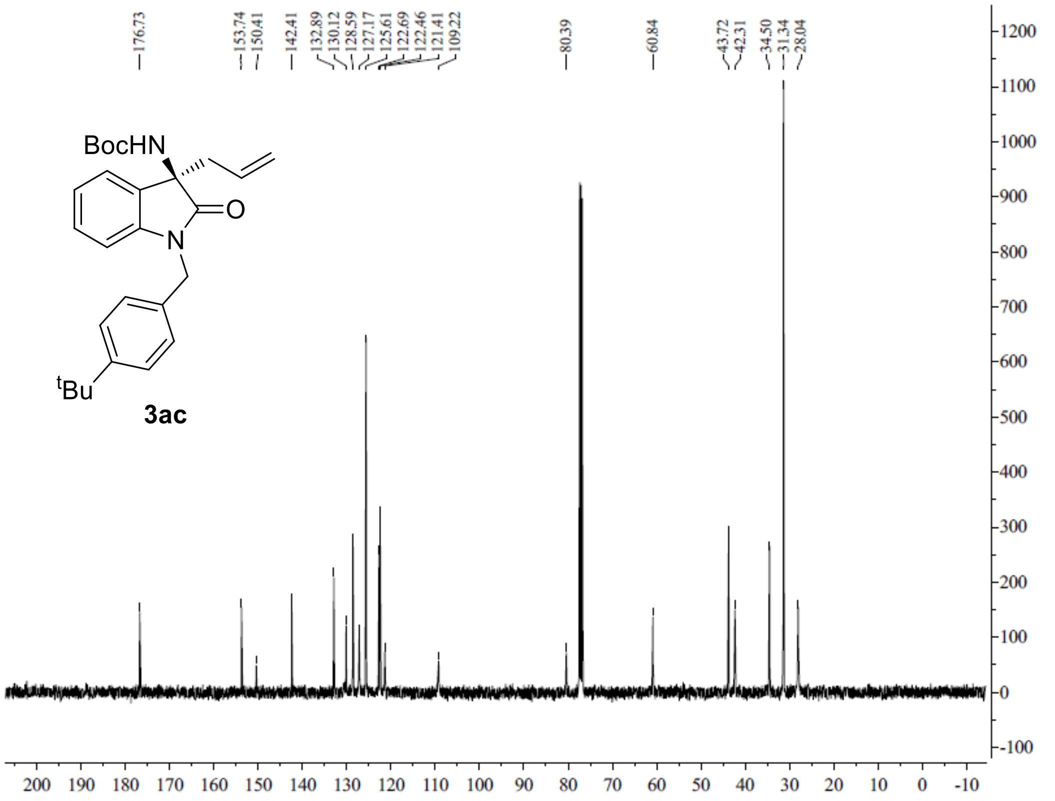

Figure S98. ^{13}C NMR spectrum of **3ac**, related to **Figure 2.**

<Chromatogram>

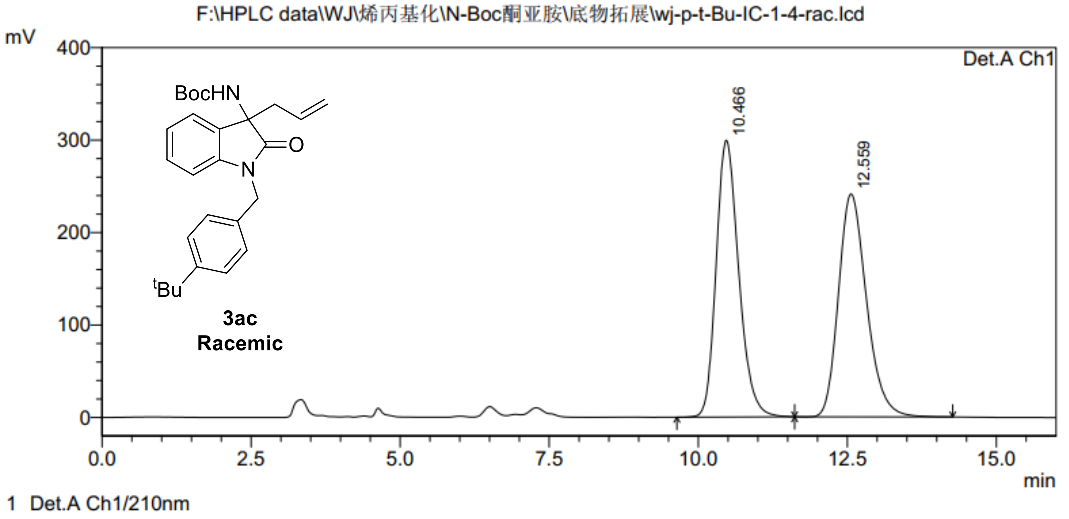

PeakTable

Detector A Ch1 210nm

Peak#	Ret. Time	Area	Height	Area %	Height %
1	10.466	7887562	299375	50.035	55.395
2	12.559	7876543	241062	49.965	44.605
Total		15764106	540437	100.000	100.000

<Chromatogram>

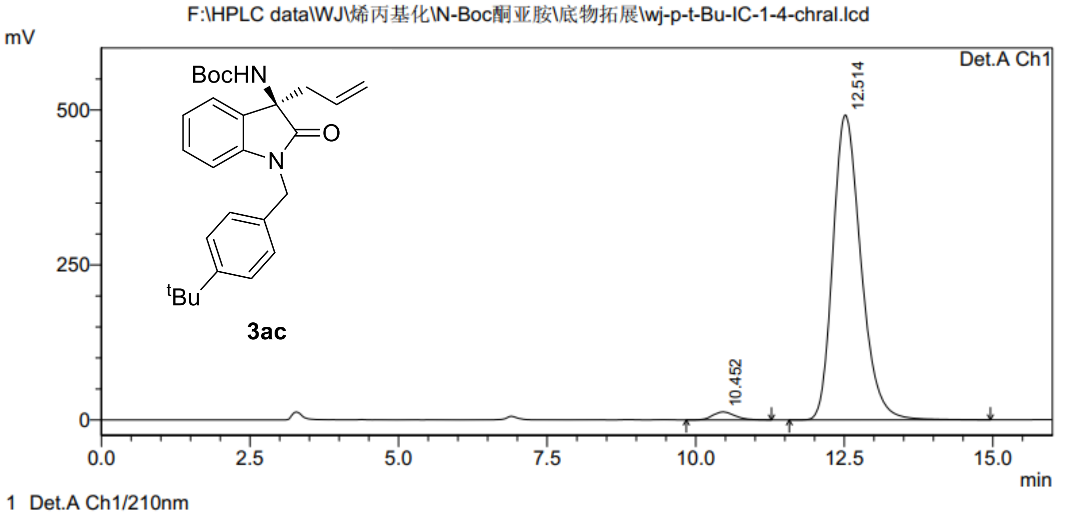

PeakTable

Detector A Ch1 210nm

Peak#	Ret. Time	Area	Height	Area %	Height %
1	10.452	335250	13078	2.036	2.590
2	12.514	16127312	491892	97.964	97.410
Total		16462561	504970	100.000	100.000

Figure S99. HPLC spectrum of **3ac**, related to **Figure 2.**

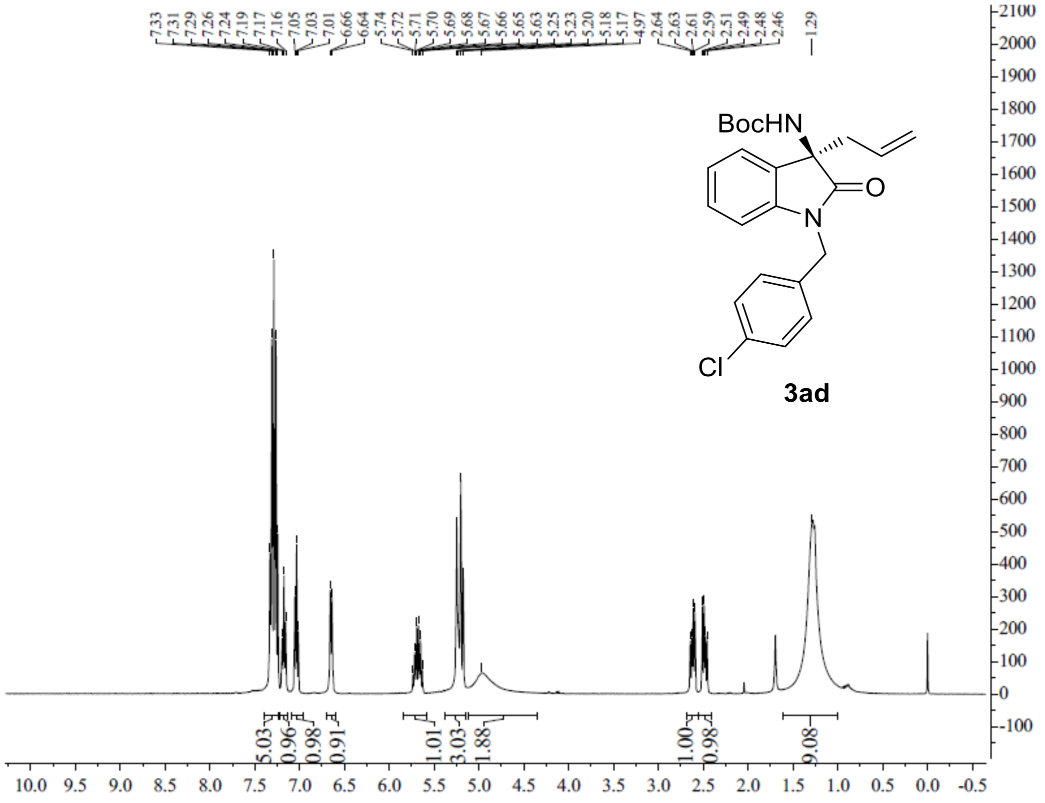

Figure S100. ^{1}H NMR spectrum of **3ad**, related to **Figure 2**.

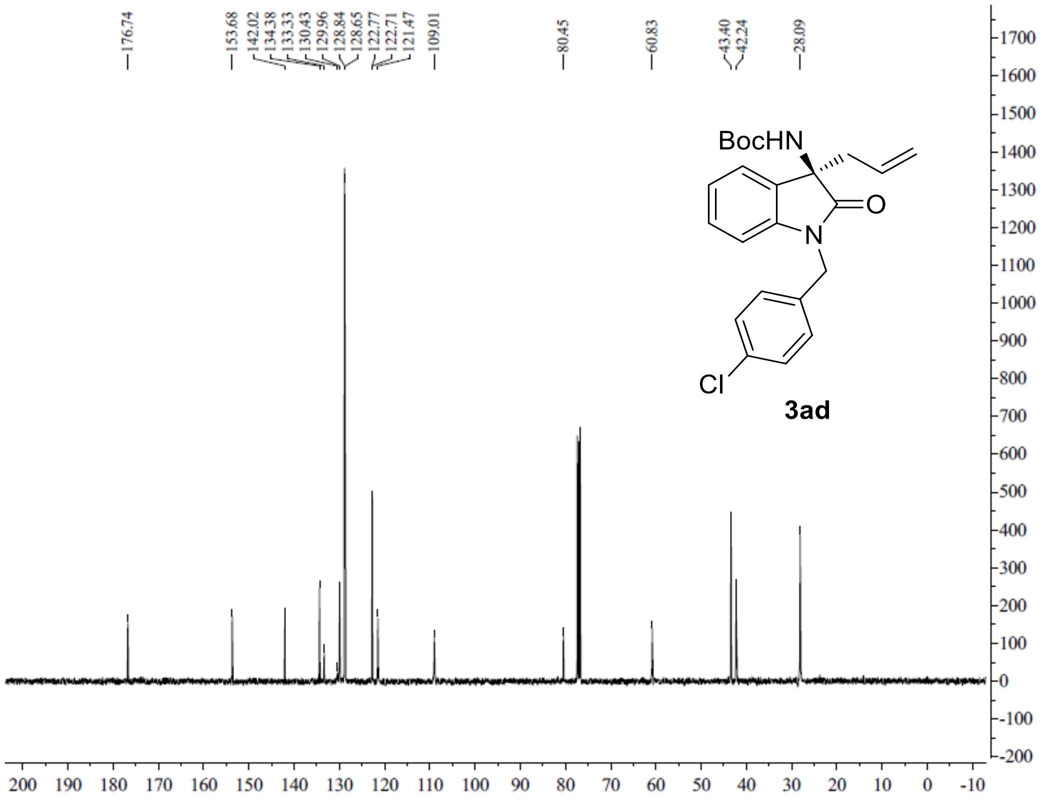

Figure S101. ^{13}C NMR spectrum of **3ad**, related to **Figure 2.**

<Chromatogram>

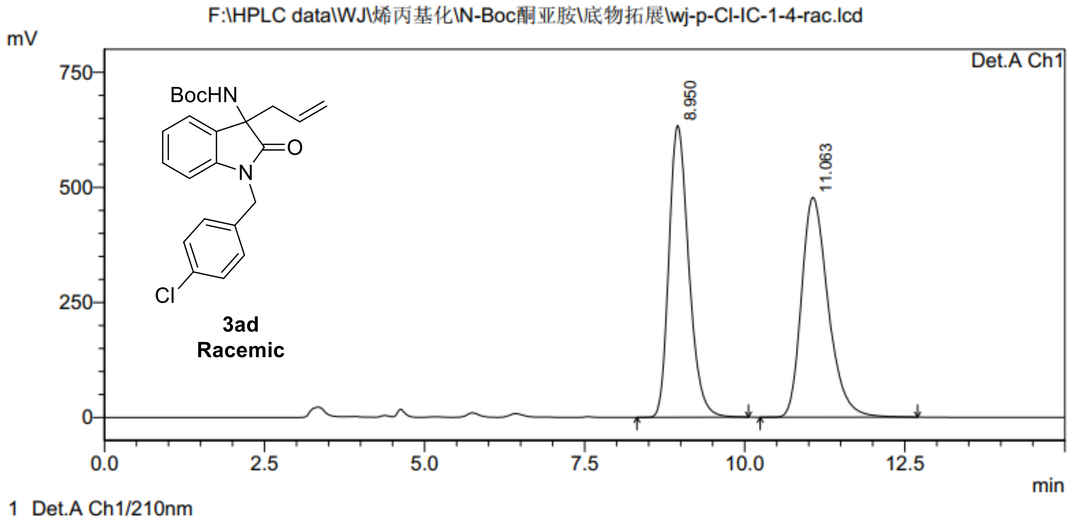

PeakTable

Detector A Ch1 210nm

Peak#	Ret. Time	Area	Height	Area %	Height %
1	8.950	13668238	633755	49.719	57.023
2	11.063	13822765	477649	50.281	42.977
Total		27491003	1111404	100.000	100.000

<Chromatogram>

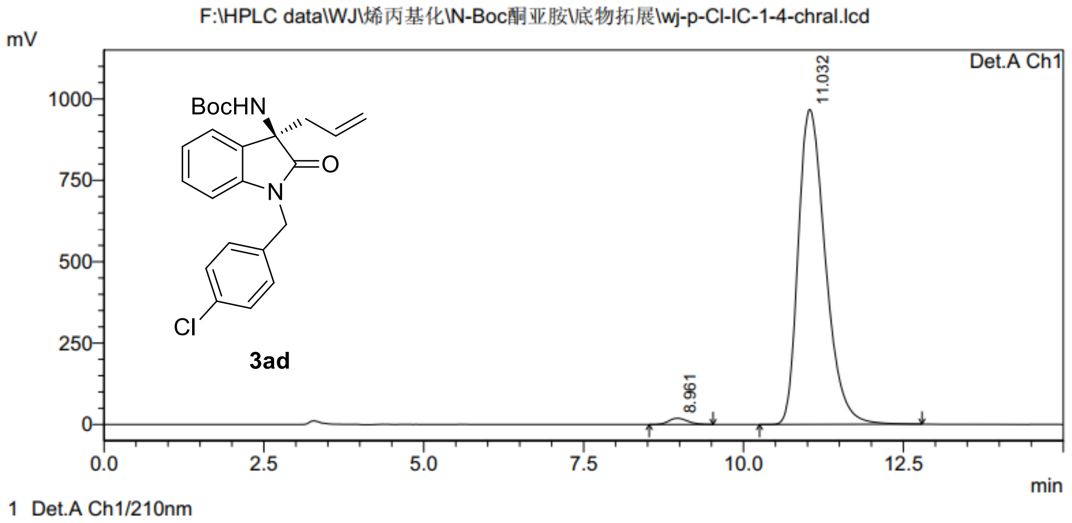

PeakTable

Detector A Ch1 210nm

Peak#	Ret. Time	Area	Height	Area %	Height %
1	8.961	394797	19204	1.360	1.947
2	11.032	28624871	967154	98.640	98.053
Total		29019669	986358	100.000	100.000

Figure S102. HPLC spectrum of **3ad**, related to **Figure 2.**

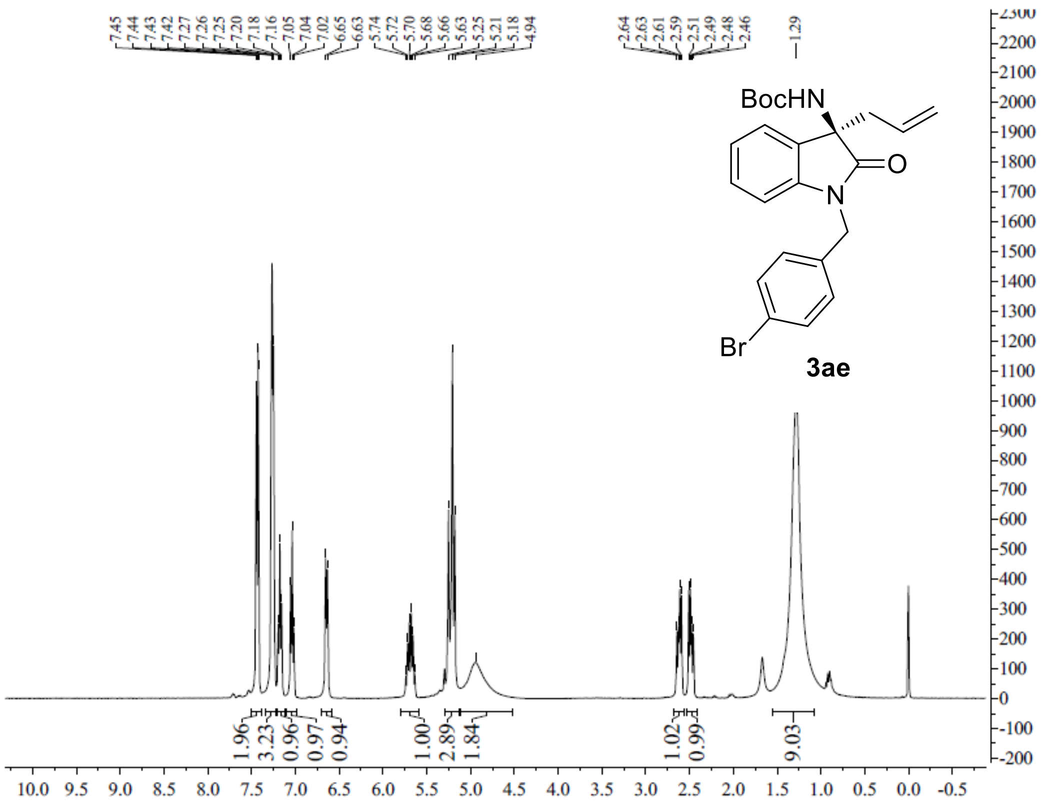

Figure S103. ^{1}H NMR spectrum of **3**ae, related to **Figure 2**.

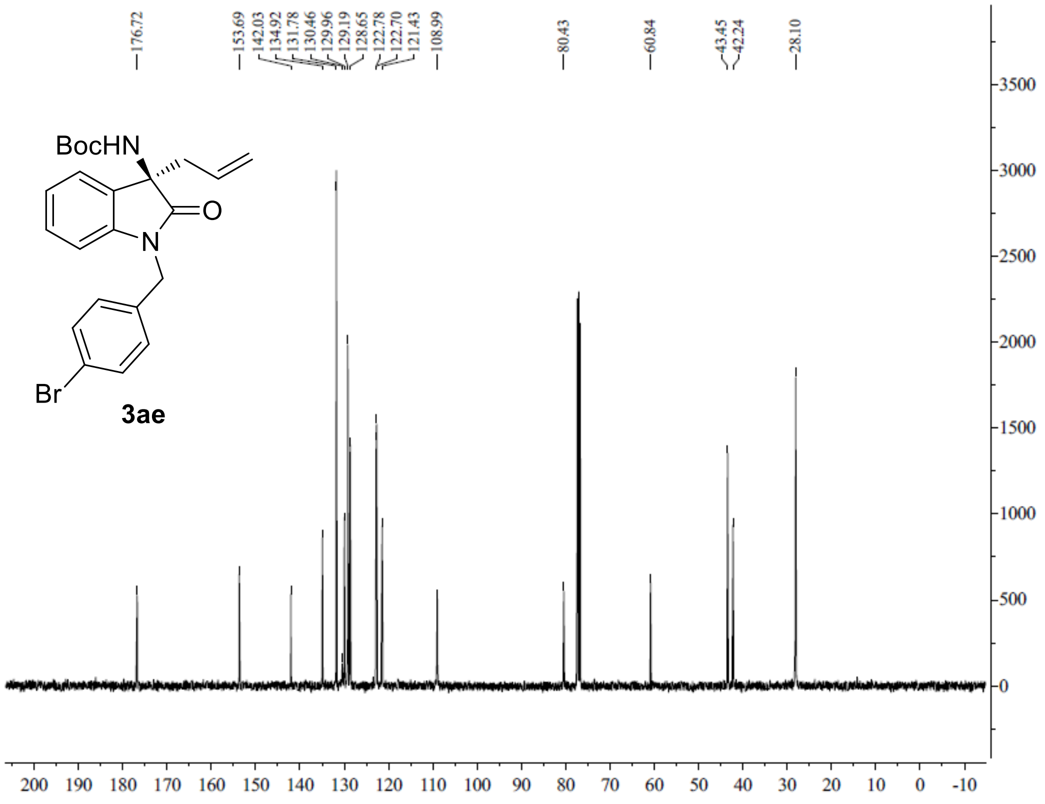

Figure S104. ^{13}C NMR spectrum of **3ae**, related to **Figure 2.**

<Chromatogram>

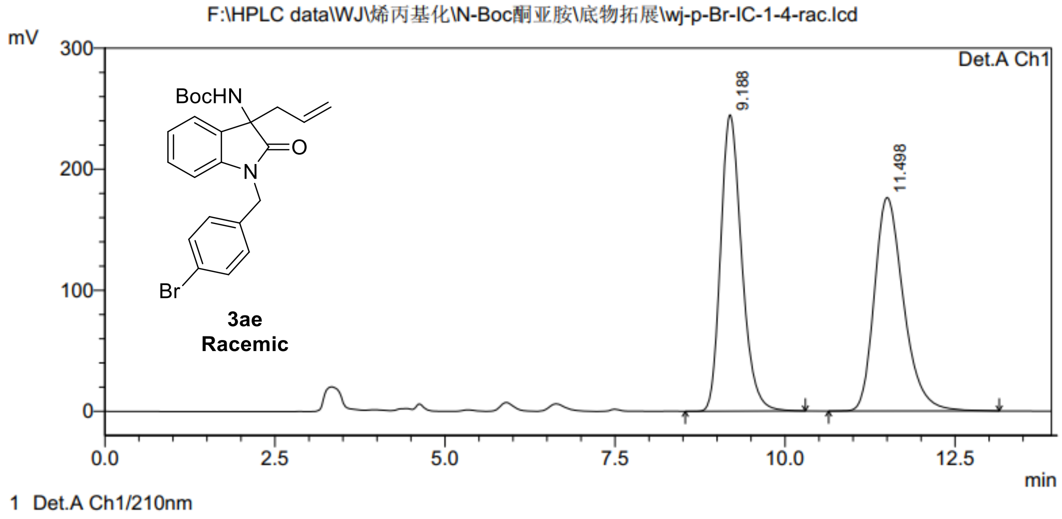

PeakTable

Detector A Ch1 210nm

Peak#	Ret. Time	Area	Height	Area %	Height %
1	9.188	5264945	244794	50.012	58.120
2	11.498	5262434	176395	49.988	41.880
Total		10527379	421189	100.000	100.000

<Chromatogram>

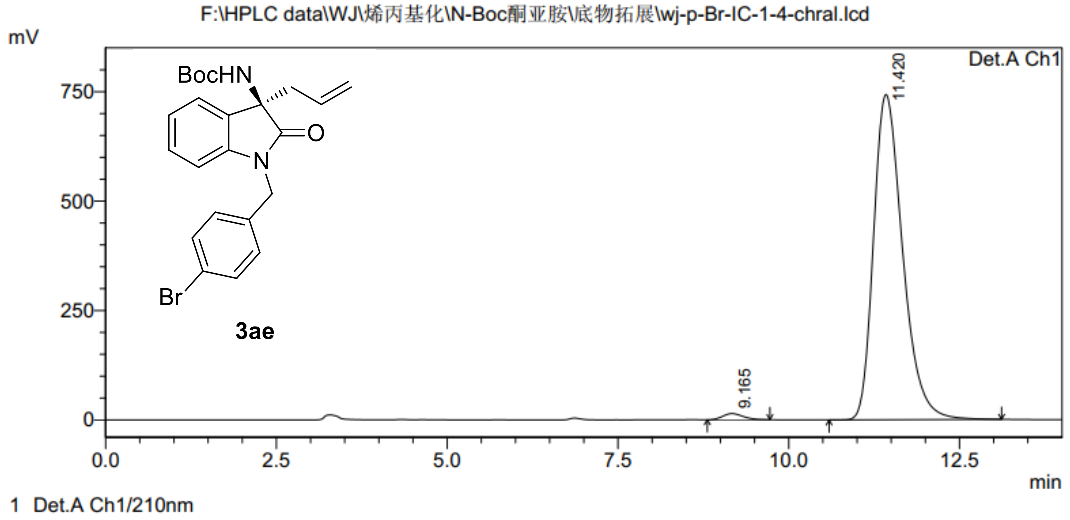

PeakTable

Detector A Ch1 210nm

Peak#	Ret. Time	Area	Height	Area %	Height %
1	9.165	291242	14031	1.306	1.853
2	11.420	22004911	743079	98.694	98.147
Total		22296153	757110	100.000	100.000

Figure S105. HPLC spectrum of **3ae**, related to **Figure 2.**

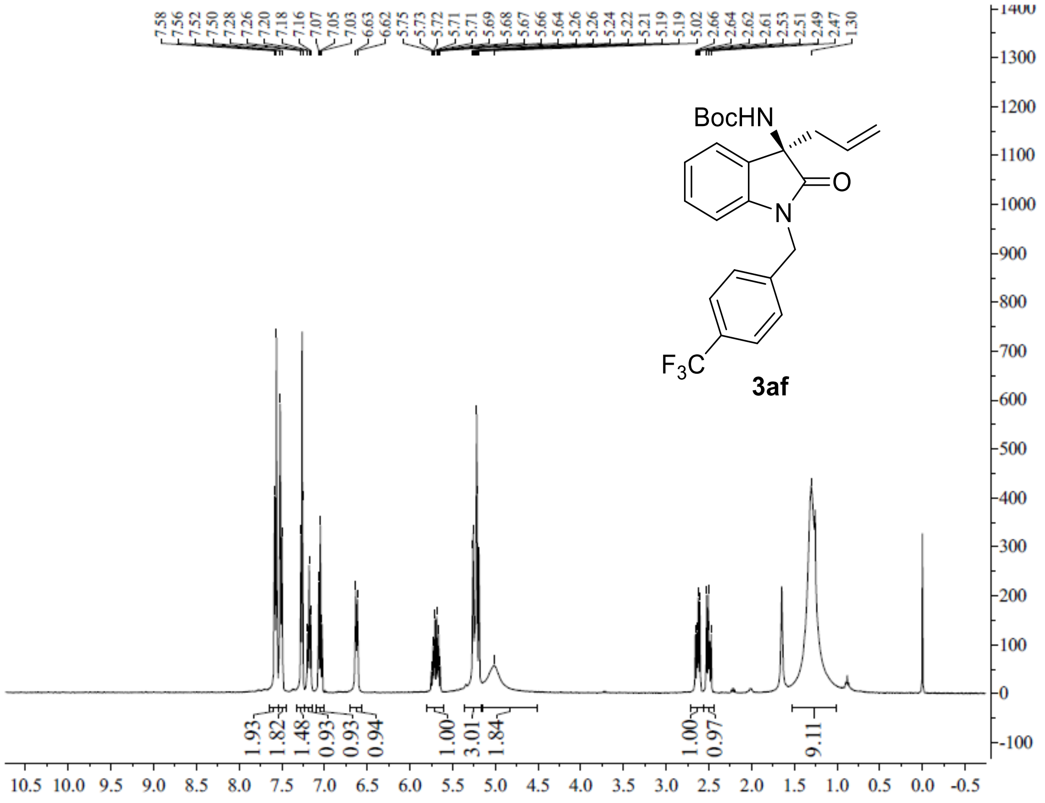

Figure S106. ^{1}H NMR spectrum of **3af**, related to **Figure 2**.

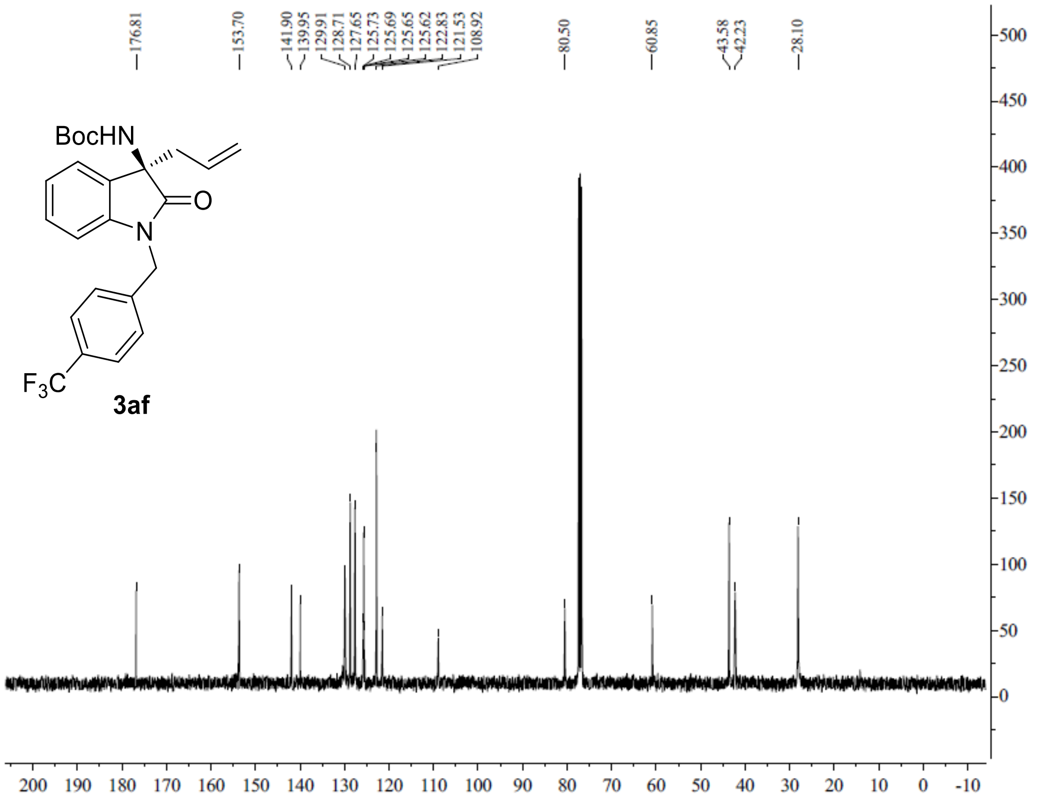

Figure S107. ^{13}C NMR spectrum of **3af**, related to **Figure 2.**

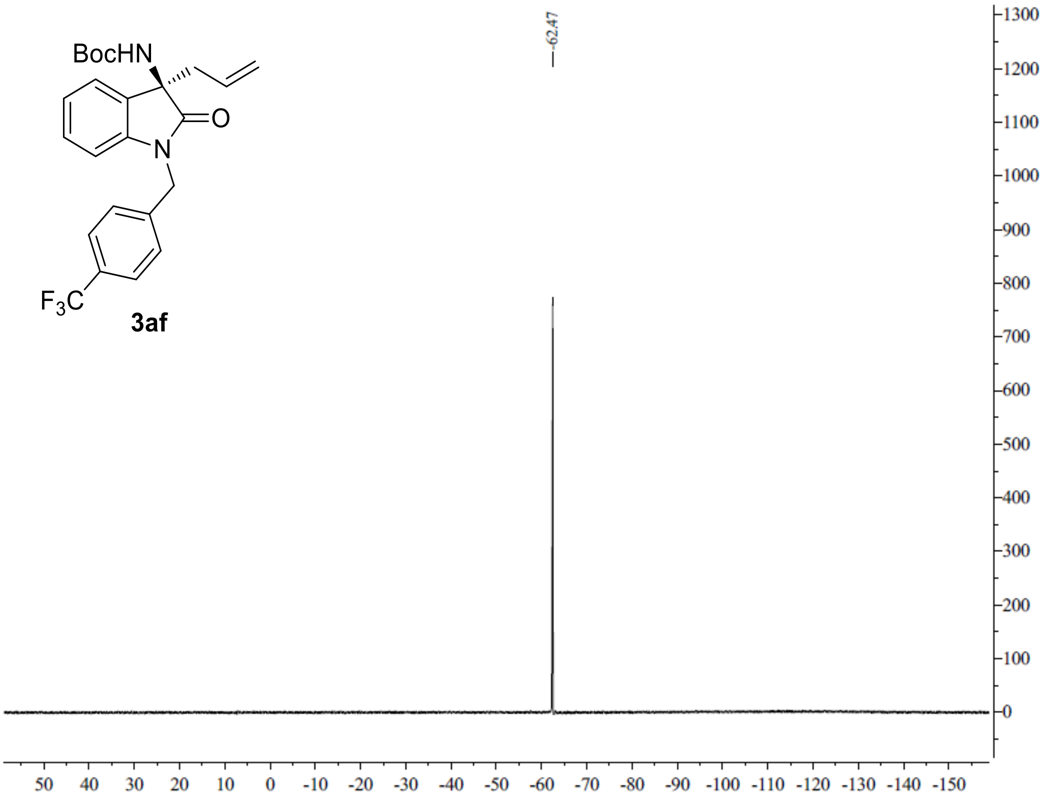

3af

Figure S108. ^{19}F NMR spectrum of **3af**, related to **Figure 2.**

<Chromatogram>

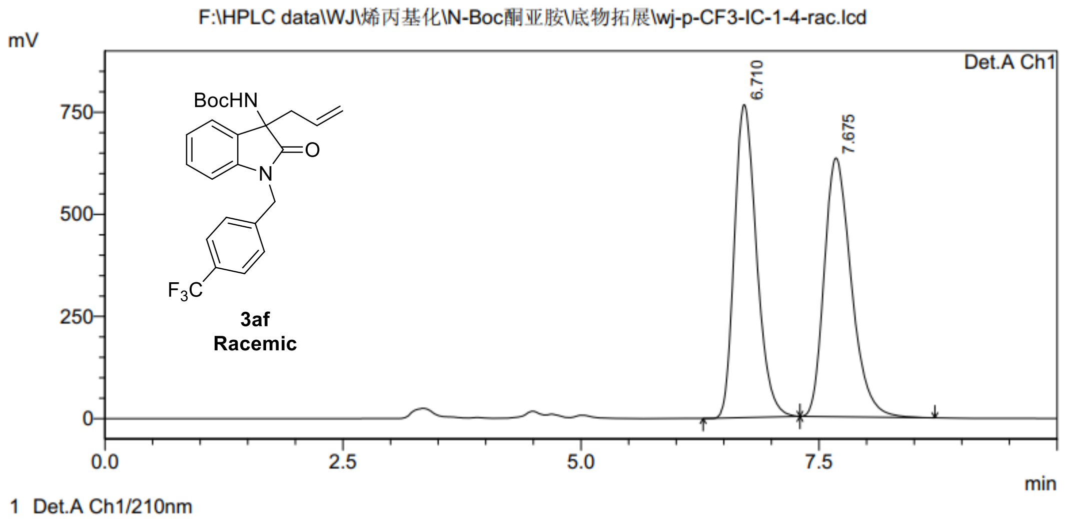

PeakTable

Detector A Ch1 210nm

Peak#	Ret. Time	Area	Height	Area %	Height %
1	6.710	12399574	766609	49.682	54.758
2	7.675	12558430	633377	50.318	45.242
Total		24958005	1399986	100.000	100.000

<Chromatogram>

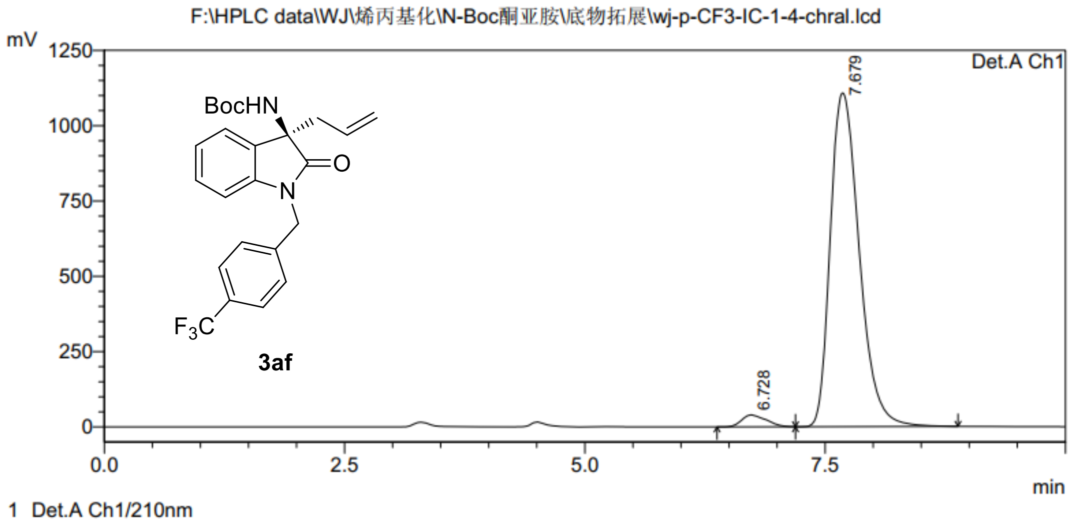

PeakTable

Detector A Ch1 210nm

Peak#	Ret. Time	Area	Height	Area %	Height %
1	6.728	700662	39808	2.929	3.469
2	7.679	23217488	1107694	97.071	96.531
Total		23918149	1147502	100.000	100.000

Figure S109. HPLC spectrum of **3af**, related to **Figure 2.**

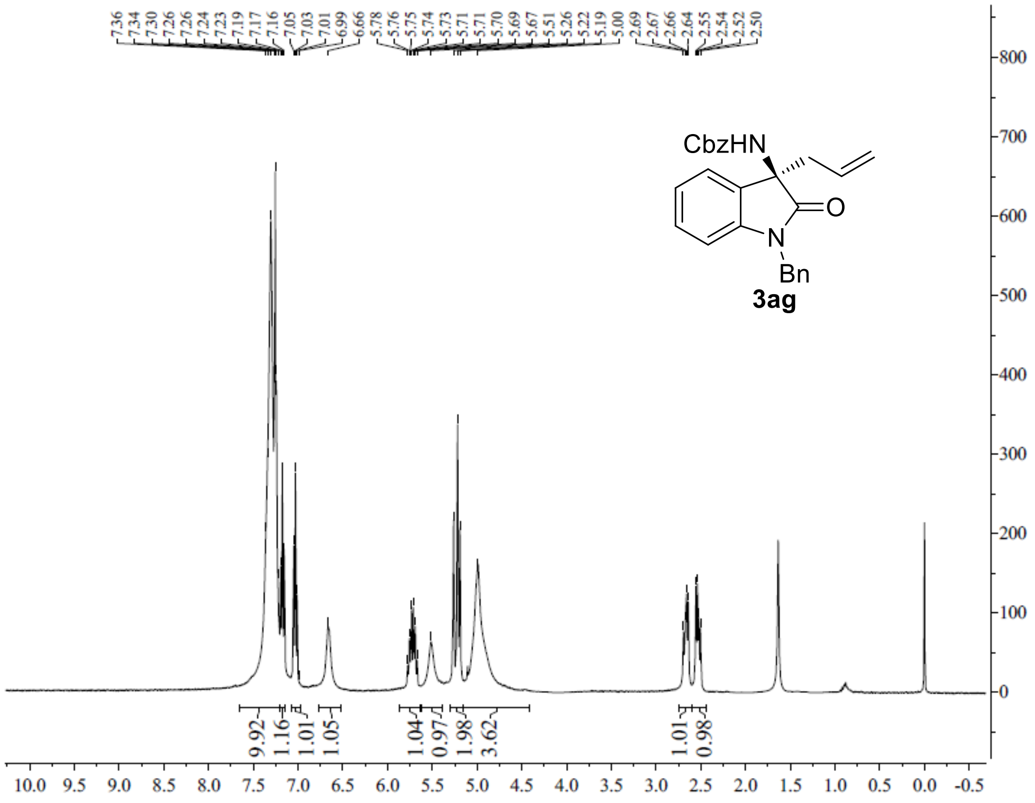

Figure S110. ^{1}H NMR spectrum of **3**ag, related to **Figure 2**.

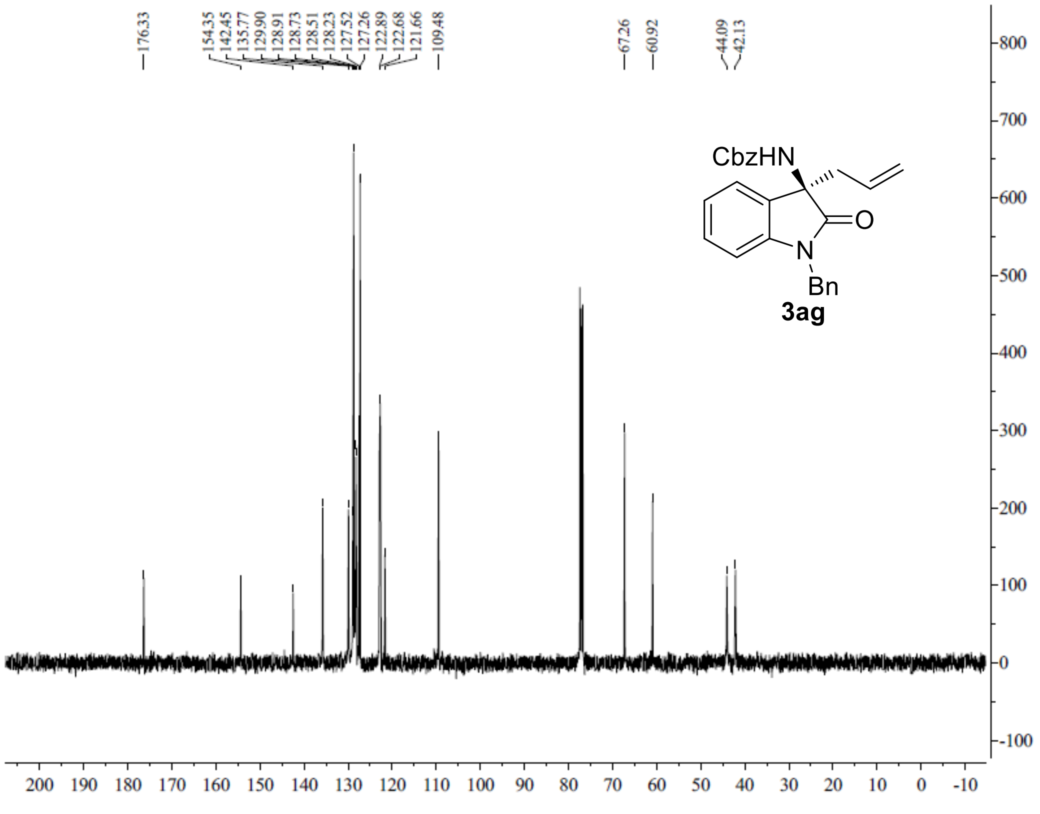

Figure S111. ^{13}C NMR spectrum of **3ag**, related to **Figure 2.**

<Chromatogram>

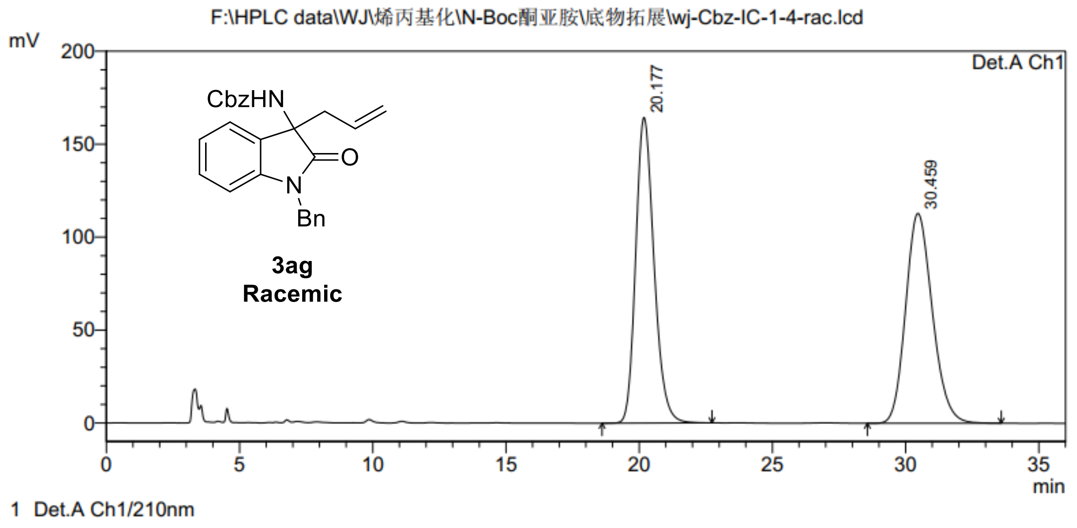

PeakTable

Detector A Ch1 210nm

Peak#	Ret. Time	Area	Height	Area %	Height %
1	20.177	7852147	164445	49.951	59.289
2	30.459	7867615	112916	50.049	40.711
Total		15719762	277361	100.000	100.000

<Chromatogram>

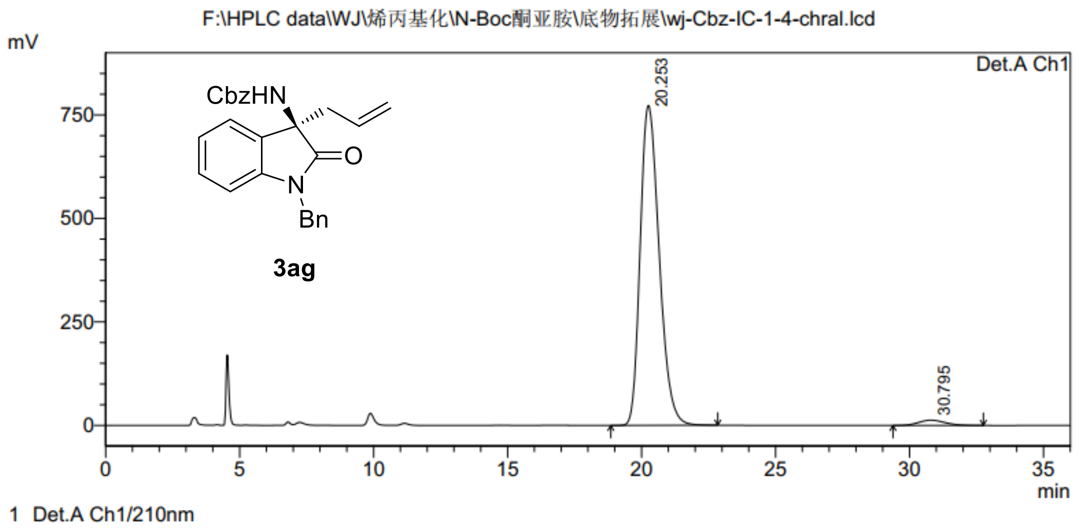

PeakTable

Detector A Ch1 210nm

Peak#	Ret. Time	Area	Height	Area %	Height %
1	20.253	38065591	772770	97.718	98.380
2	30.795	889033	12724	2.282	1.620
Total		38954624	785495	100.000	100.000

Figure S112. HPLC spectrum of **3ag**, related to **Figure 2.**

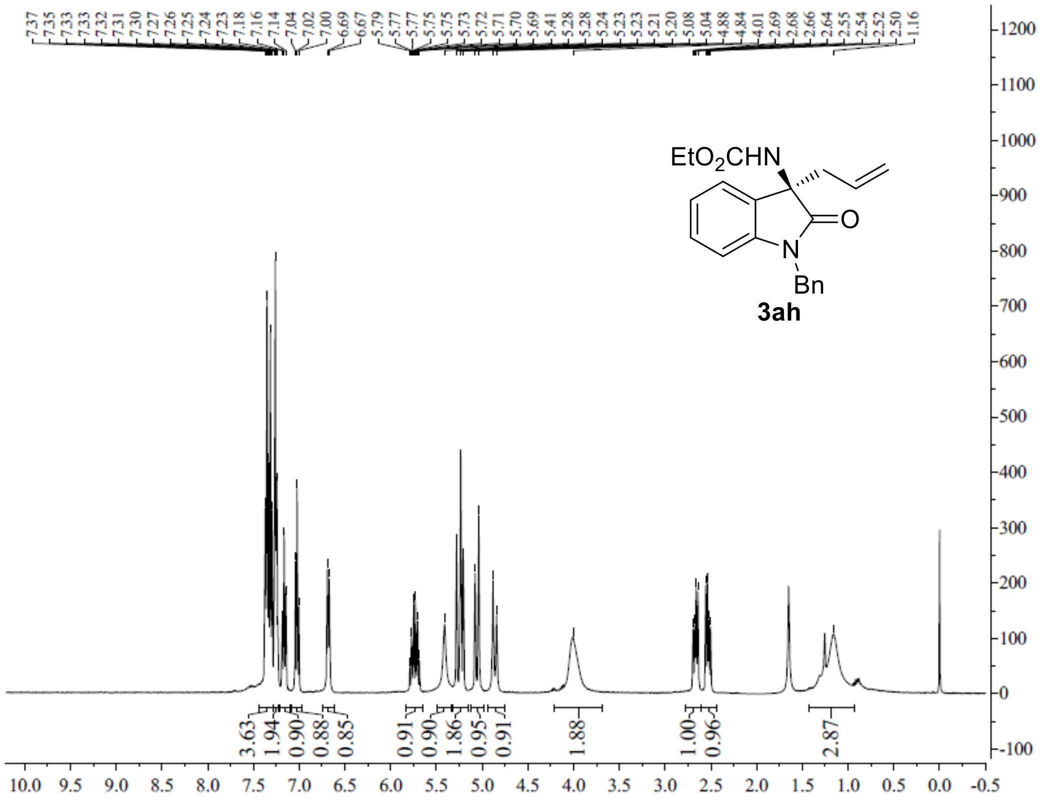

Figure S113. ^{1}H NMR spectrum of 3ah, related to **Figure 2**.

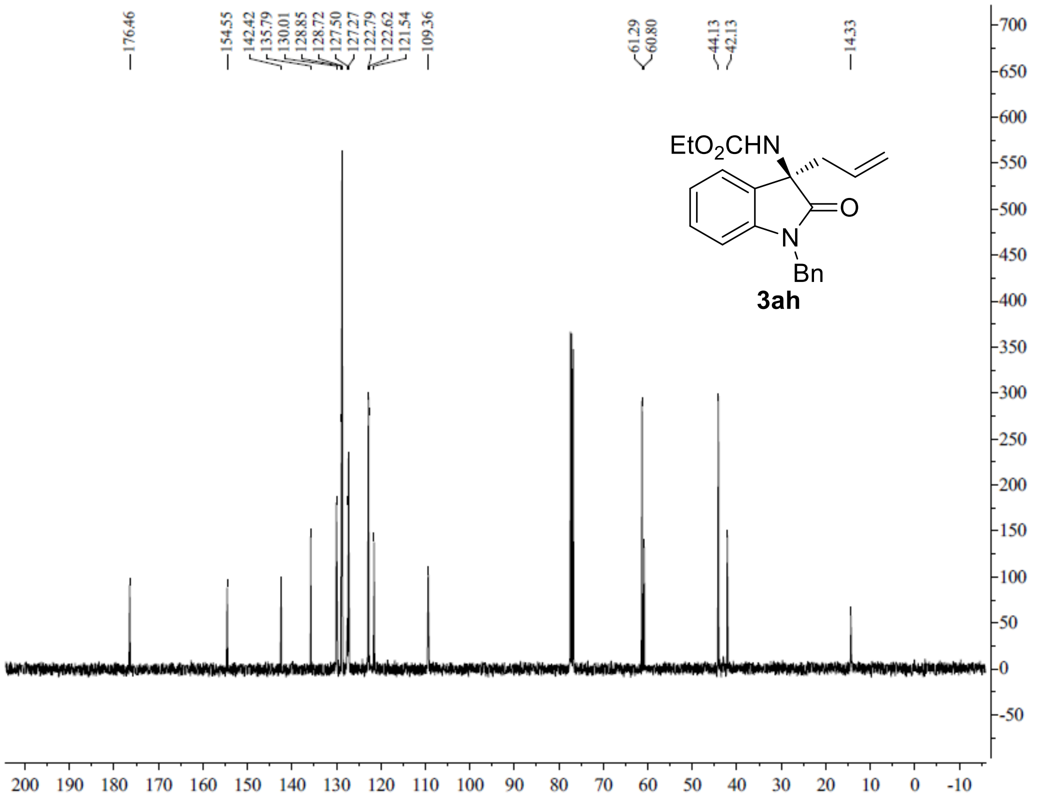

Figure S114. ^{13}C NMR spectrum of **3ah**, related to **Figure 2.**

<Chromatogram>

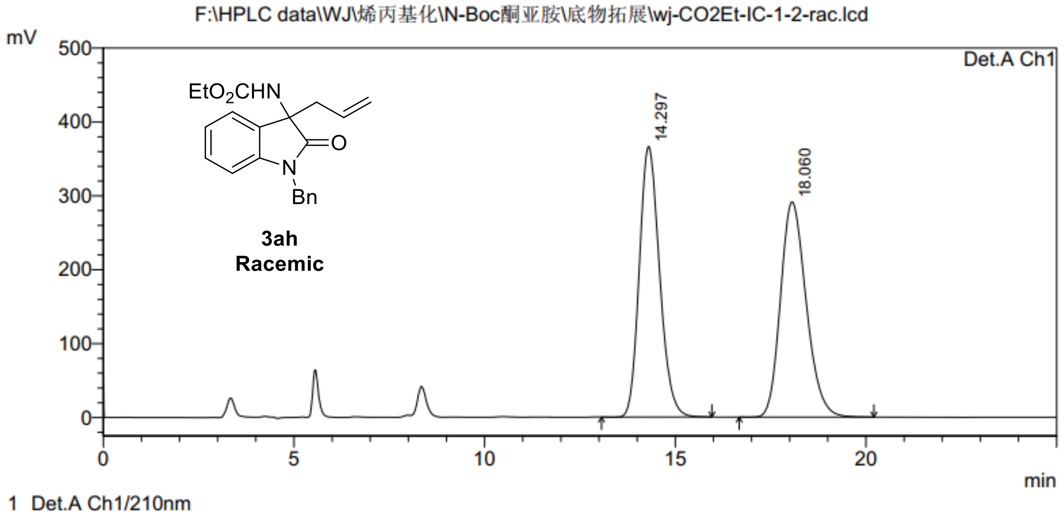

PeakTable

Detector A Ch1 210nm

Peak#	Ret. Time	Area	Height	Area %	Height %
1	14.297	13521364	366254	49.780	55.704
2	18.060	13640826	291241	50.220	44.296
Total		27162190	657496	100.000	100.000

<Chromatogram>

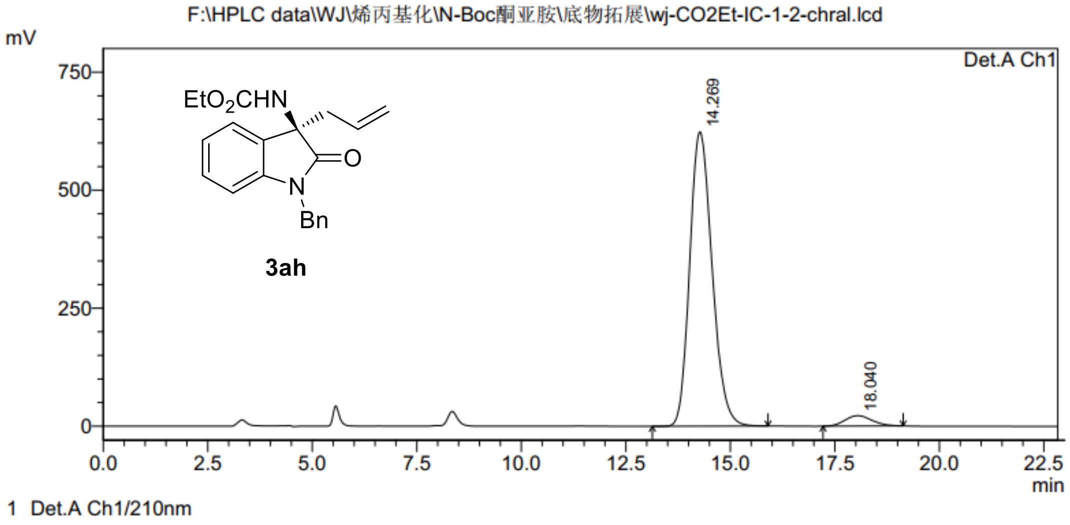

PeakTable

Detector A Ch1 210nm

Peak#	Ret. Time	Area	Height	Area %	Height %
1	14.269	23196016	623575	95.868	96.559
2	18.040	999817	22220	4.132	3.441
Total		24195833	645795	100.000	100.000

Figure S115. HPLC spectrum of **3ah**, related to **Figure 2.**

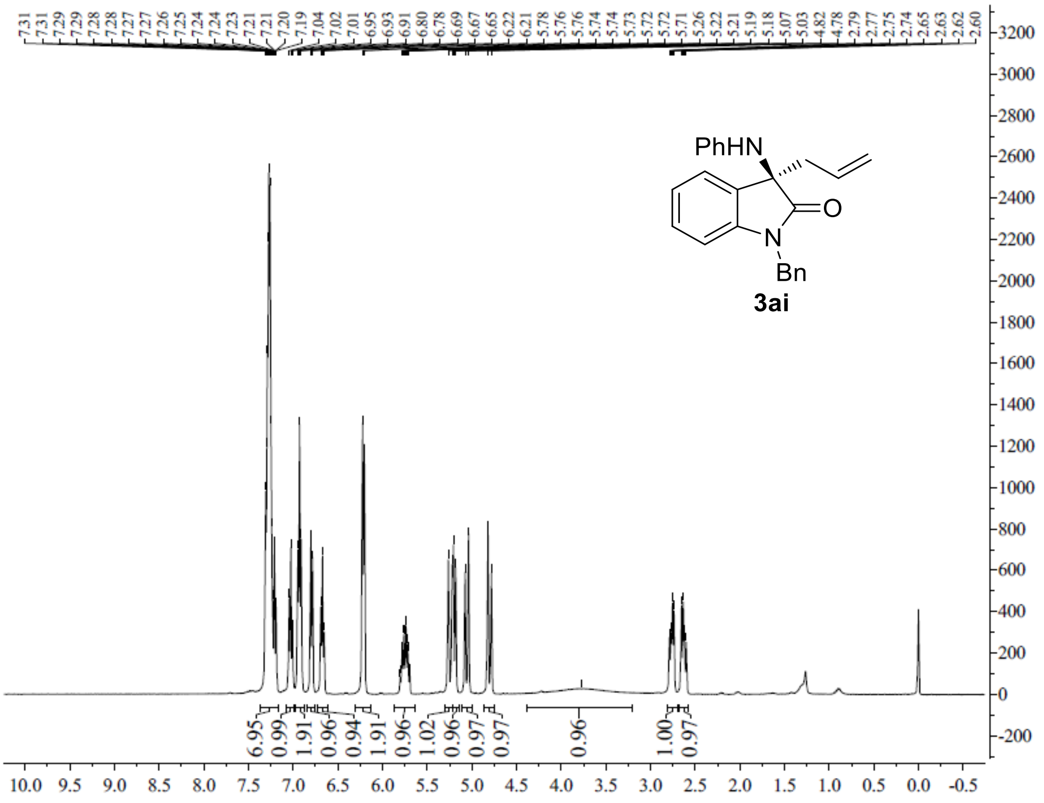

Figure S116. ^{1}H NMR spectrum of 3ai, related to **Figure 2**.

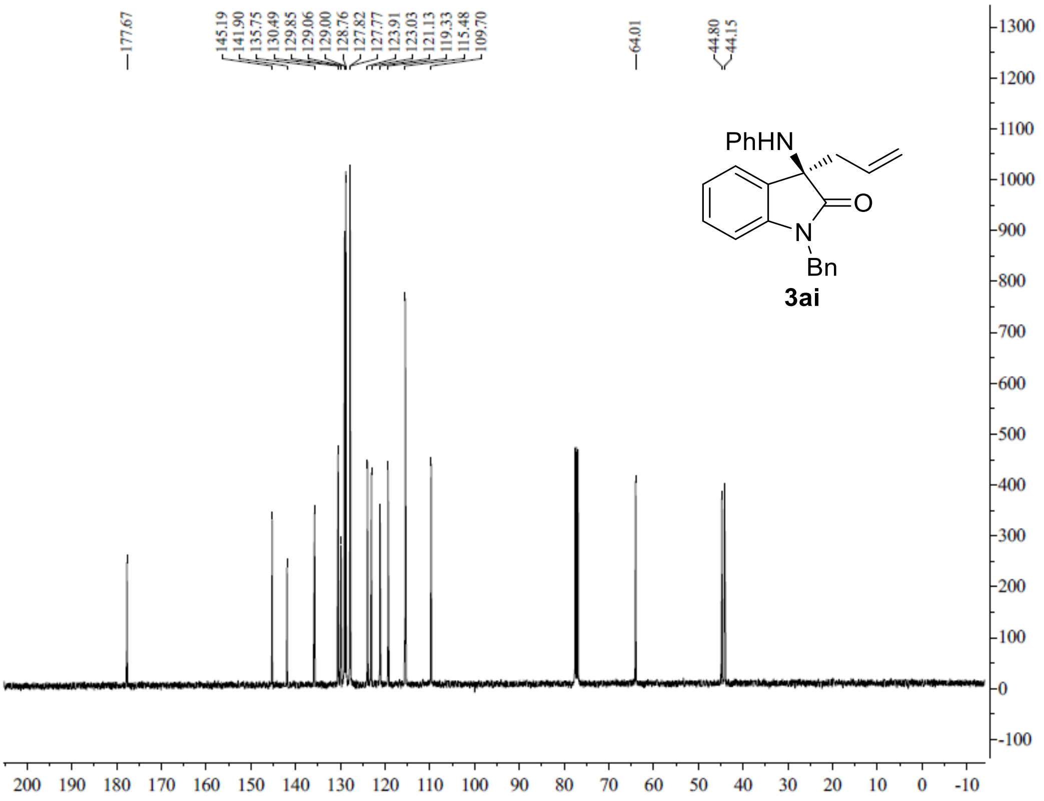

Figure S117. ^{13}C NMR spectrum of **3ai**, related to **Figure 2.**

<Chromatogram>

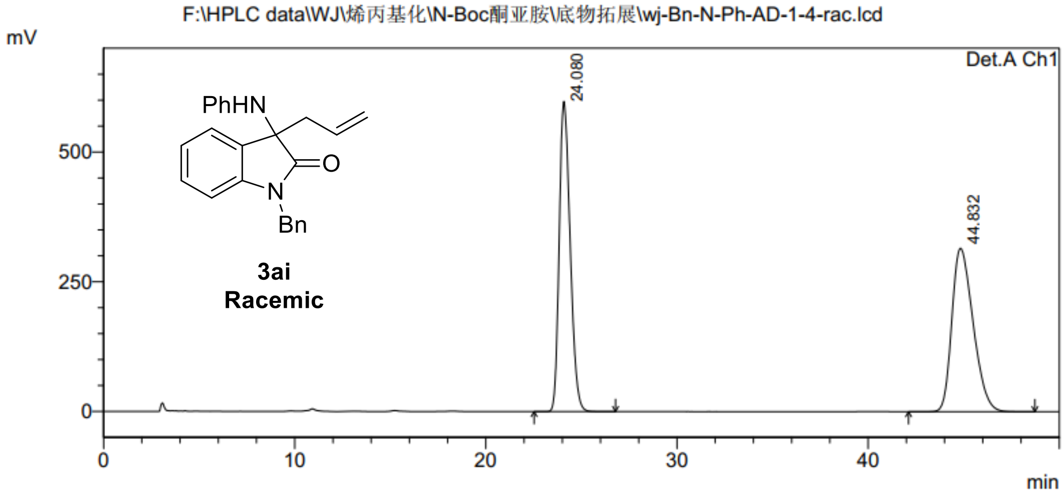

1 Det.A Ch1/210nm

PeakTable

Detector A Ch1 210nm

Peak#	Ret. Time	Area	Height	Area %	Height %
1	24.080	23818393	597827	49.514	65.513
2	44.832	24285624	314703	50.486	34.487
Total		48104017	912530	100.000	100.000

<Chromatogram>

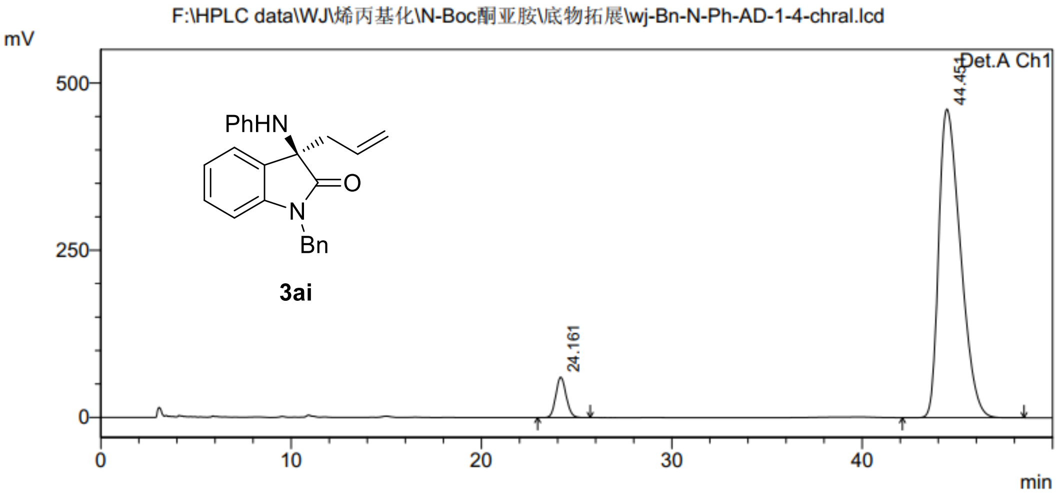

1 Det.A Ch1/210nm

PeakTable

Detector A Ch1 210nm

Peak#	Ret. Time	Area	Height	Area %	Height %
1	24.161	2283180	60379	5.889	11.574
2	44.451	36487158	461321	94.111	88.426
Total		38770337	521700	100.000	100.000

Figure S118. HPLC spectrum of **3ai**, related to **Figure 2.**

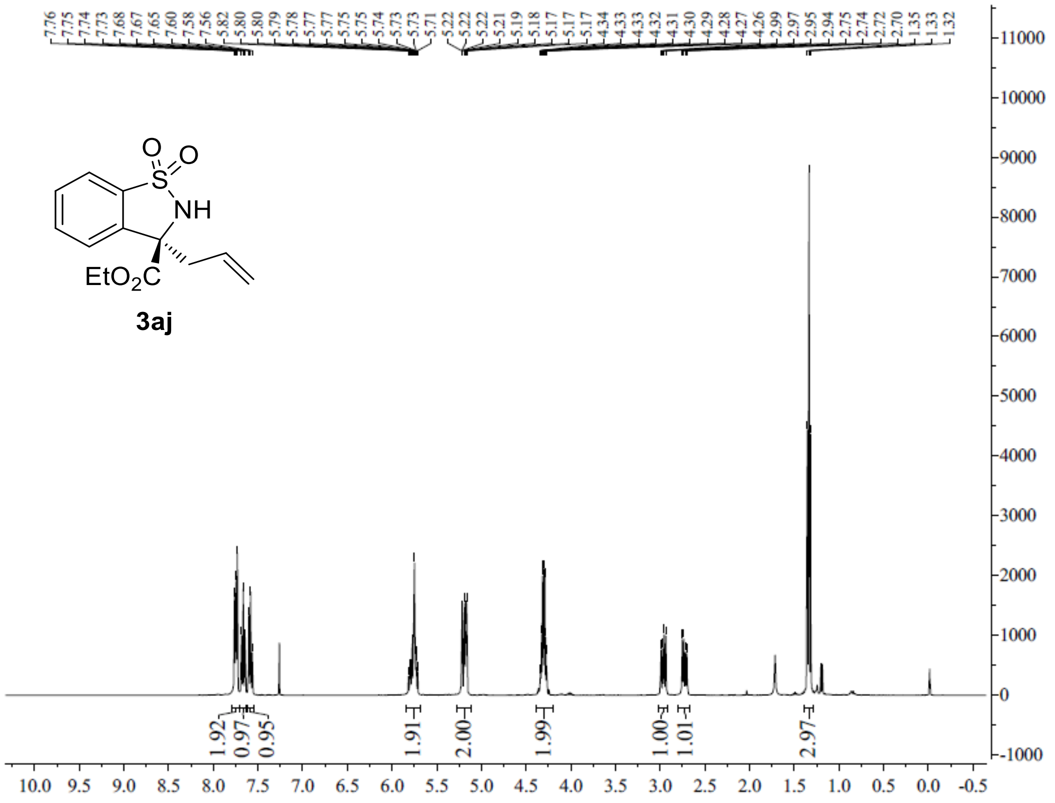

Figure S119. ^{1}H NMR spectrum of **3aj**, related to **Scheme 2**.

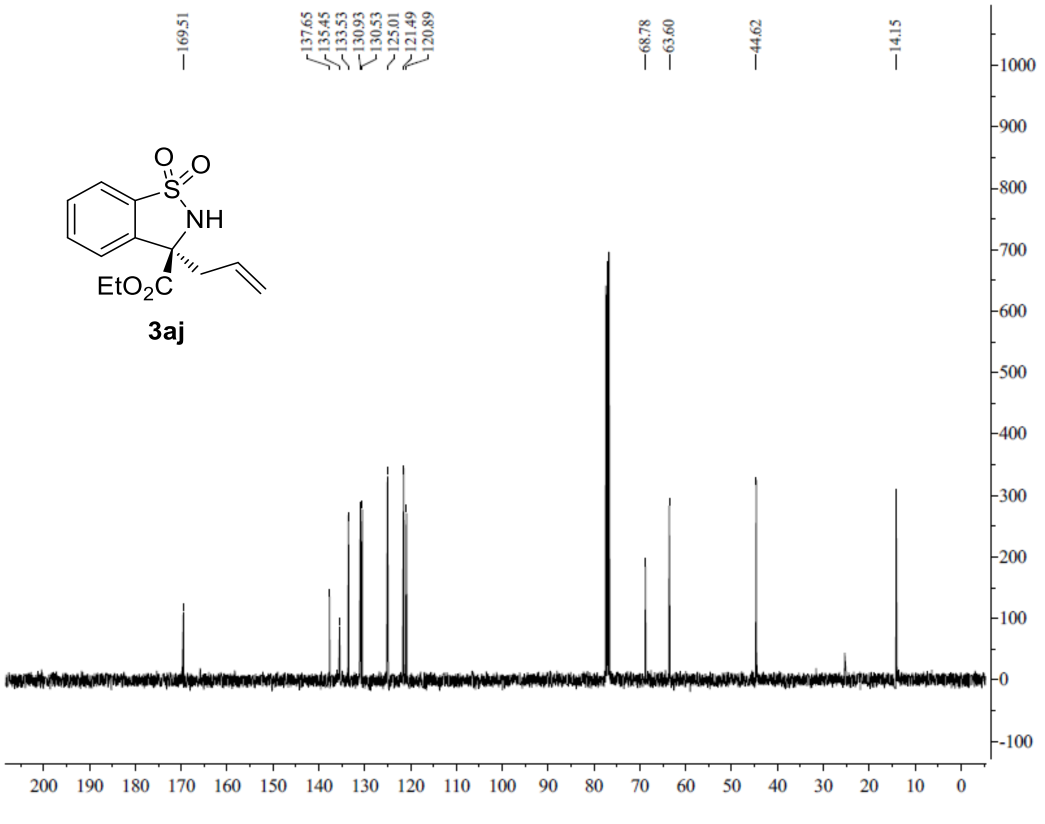

Figure S120. ^{13}C NMR spectrum of **3aj**, related to **Scheme 2.**

<Chromatogram>

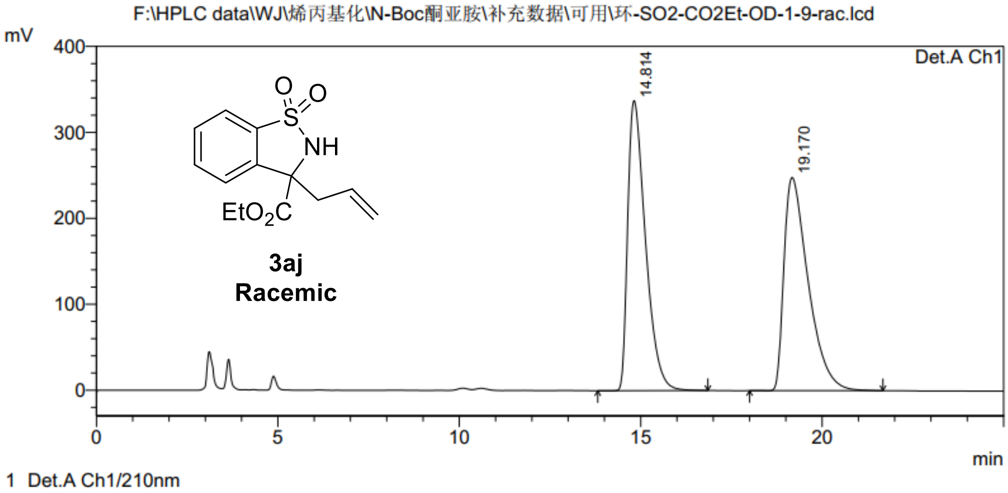

PeakTable

Detector A Ch1 210nm

Peak#	Ret. Time	Area	Height	Area %	Height %
1	14.814	11447339	337464	49.907	57.633
2	19.170	11490017	248079	50.093	42.367
Total		22937355	585542	100.000	100.000

<Chromatogram>

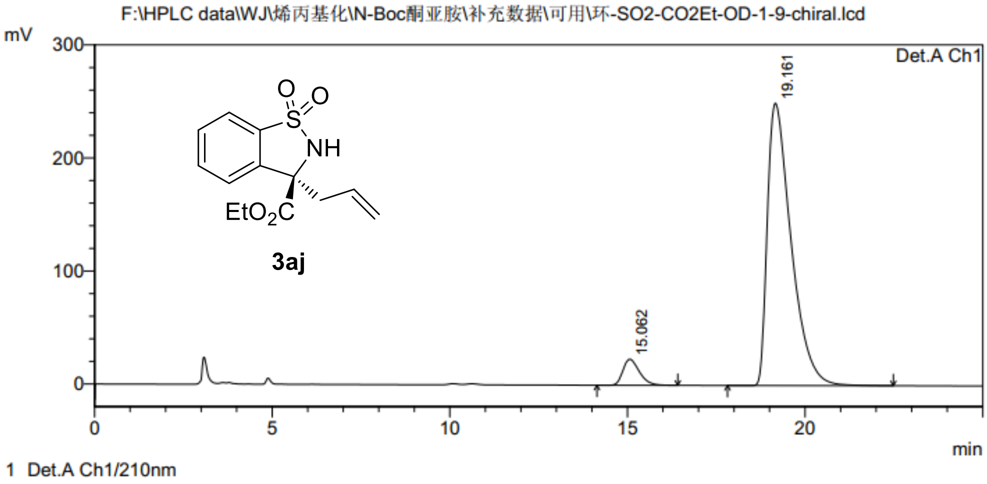

PeakTable

Detector A Ch1 210nm

Peak#	Ret. Time	Area	Height	Area %	Height %
1	15.062	737464	23034	5.978	8.443
2	19.161	11599704	249798	94.022	91.557
Total		12337169	272832	100.000	100.000

Figure S121. HPLC spectrum of **3aj**, related to **Scheme 2.**

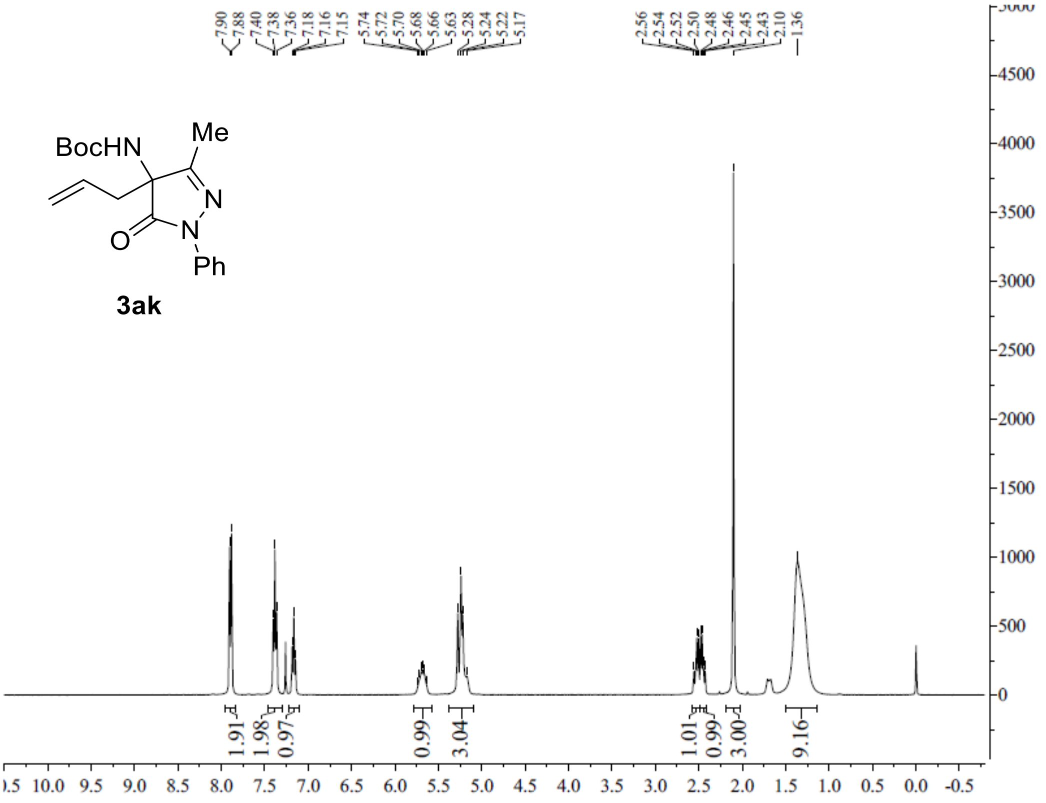

Figure S122. ^{1}H NMR spectrum of 3ak, related to Scheme 2.

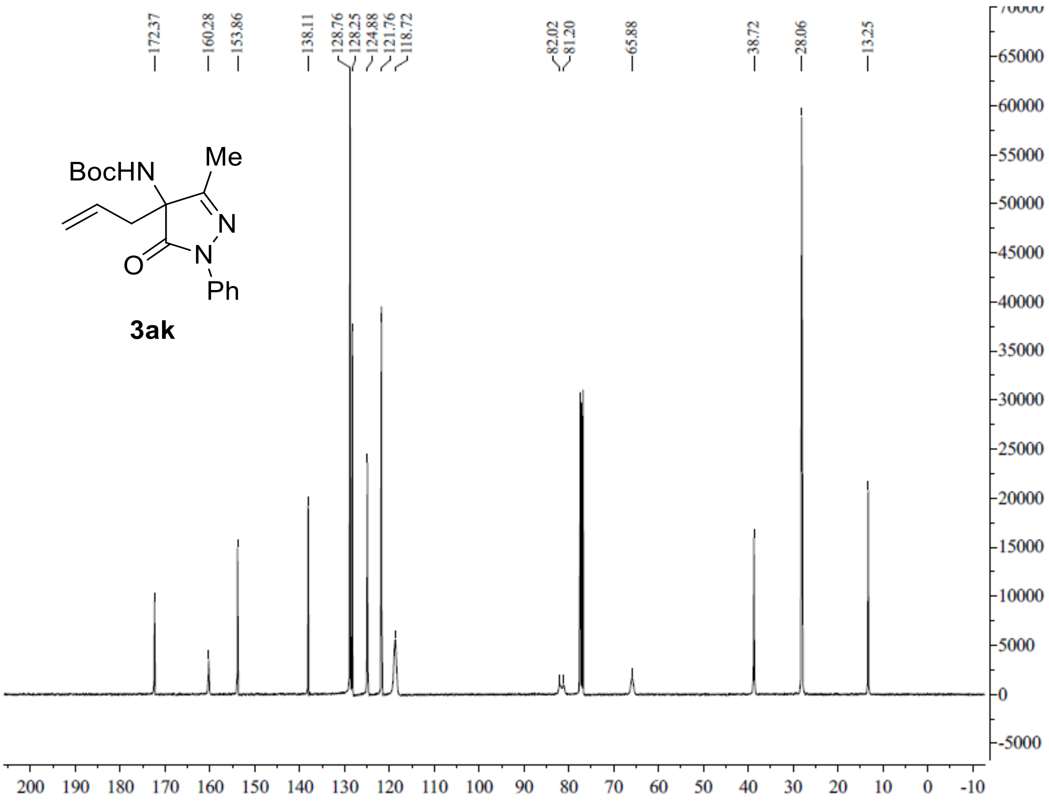

Figure S123. ^{13}C NMR spectrum of **3ak**, related to **Scheme 2.**

<Chromatogram>

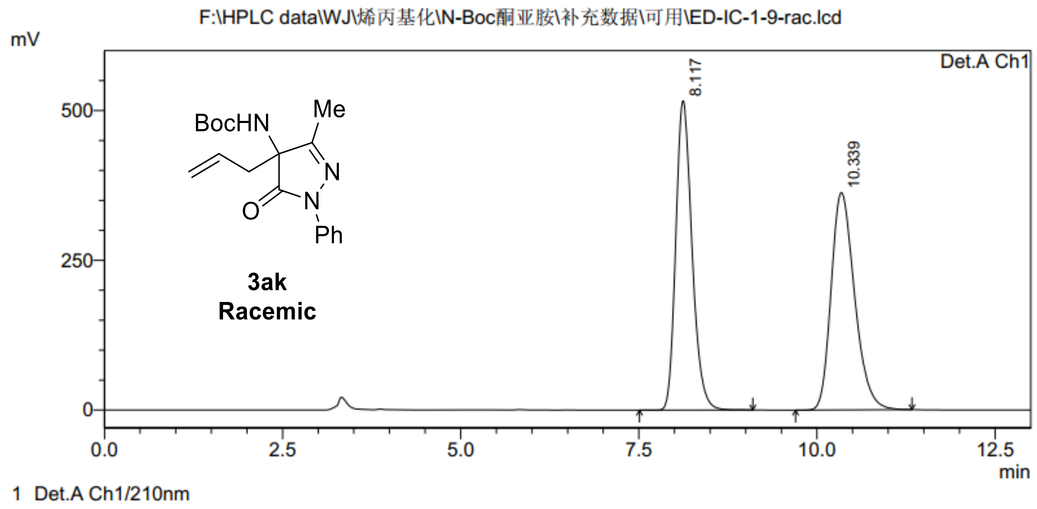

PeakTable

Detector A Ch1 210nm

Peak#	Ret. Time	Area	Height	Area %	Height %
1	8.117	8192732	516326	49.605	58.723
2	10.339	8323282	362924	50.395	41.277
Total		16516013	879250	100.000	100.000

<Chromatogram>

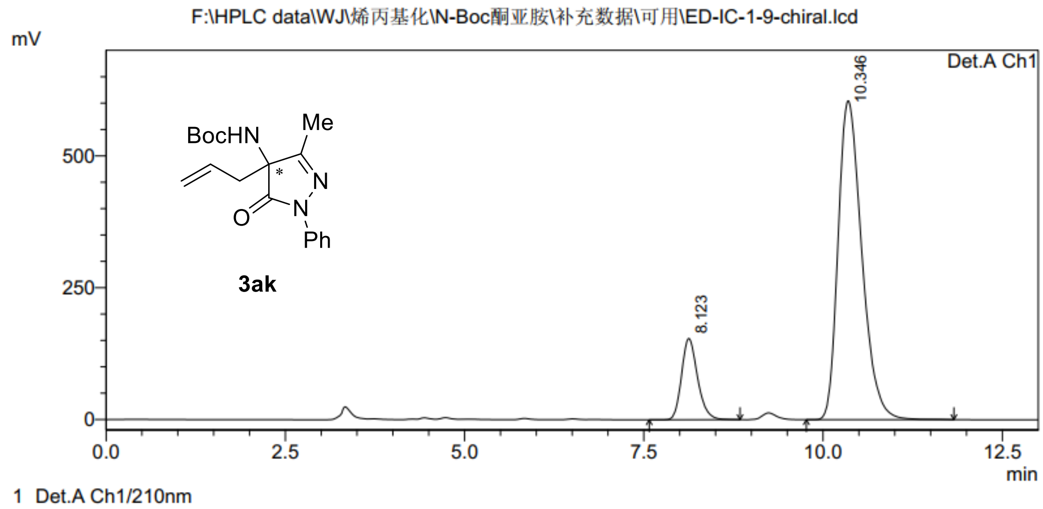

PeakTable

Detector A Ch1 210nm

Peak#	Ret. Time	Area	Height	Area %	Height %
1	8.123	2390747	153986	14.679	20.298
2	10.346	13896529	604649	85.321	79.702
Total		16287276	758634	100.000	100.000

Figure S124. HPLC spectrum of **3ak**, related to **Scheme 2.**

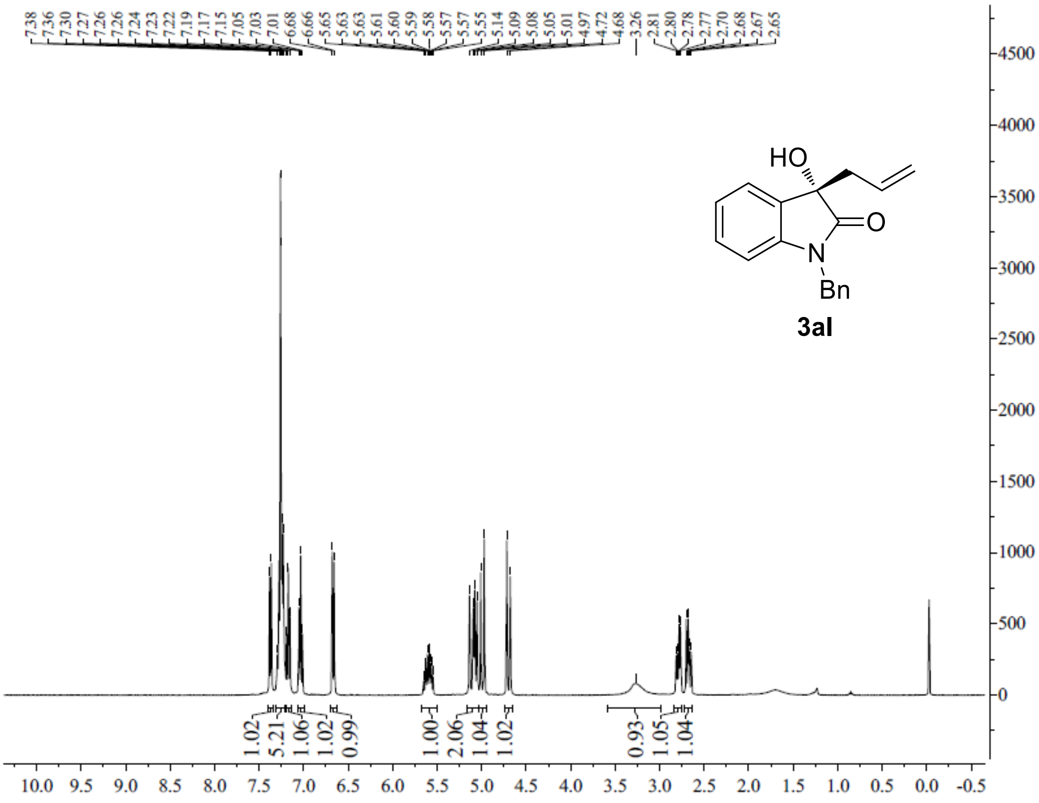

Figure S125. ^{1}H NMR spectrum of **3al**, related to **Scheme 2**.

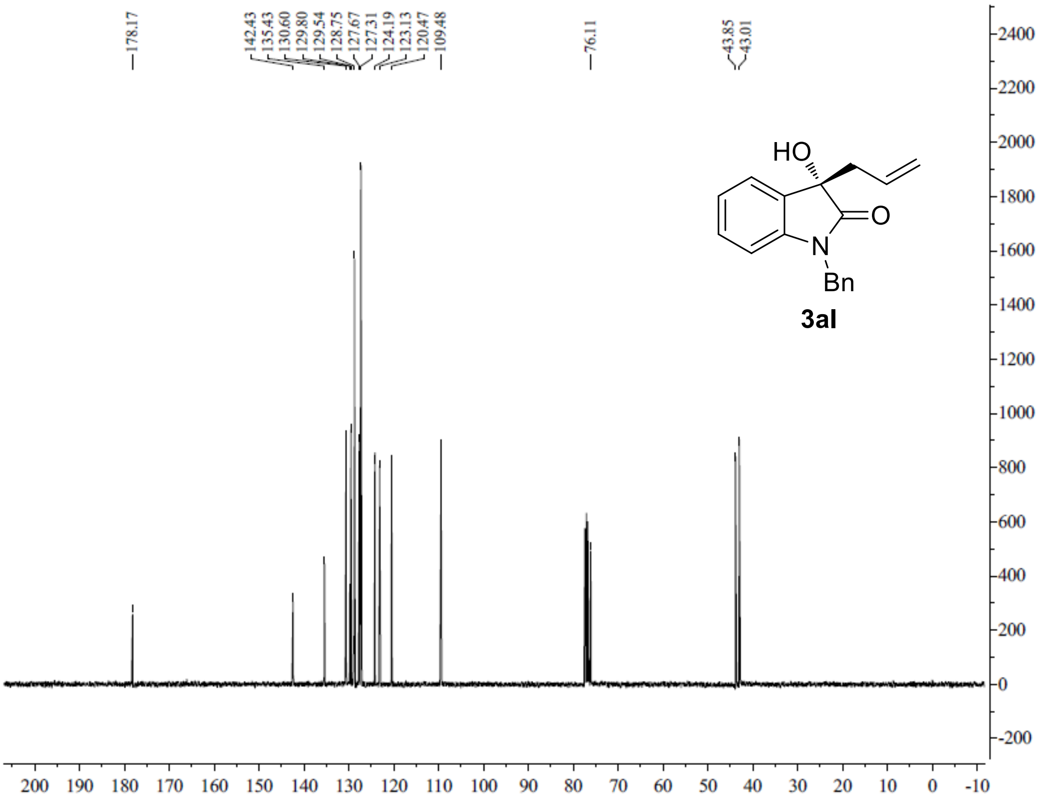

Figure S126. ^{13}C NMR spectrum of **3al**, related to **Scheme 2.**

<Chromatogram>

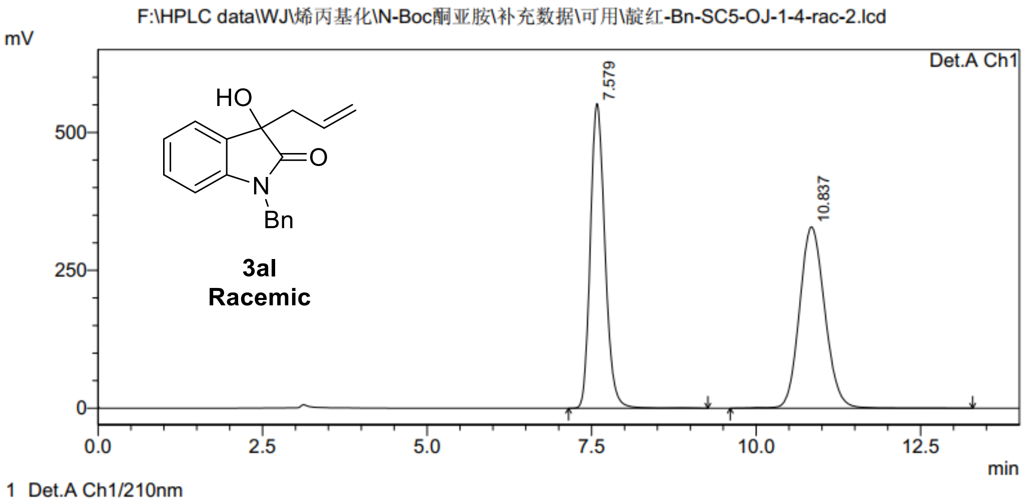

PeakTable

Detector A Ch1 210nm

Peak#	Ret. Time	Area	Height	Area %	Height %
1	7.579	8513497	552349	49.215	62.694
2	10.837	8785254	328670	50.785	37.306
Total		17298751	881019	100.000	100.000

<Chromatogram>

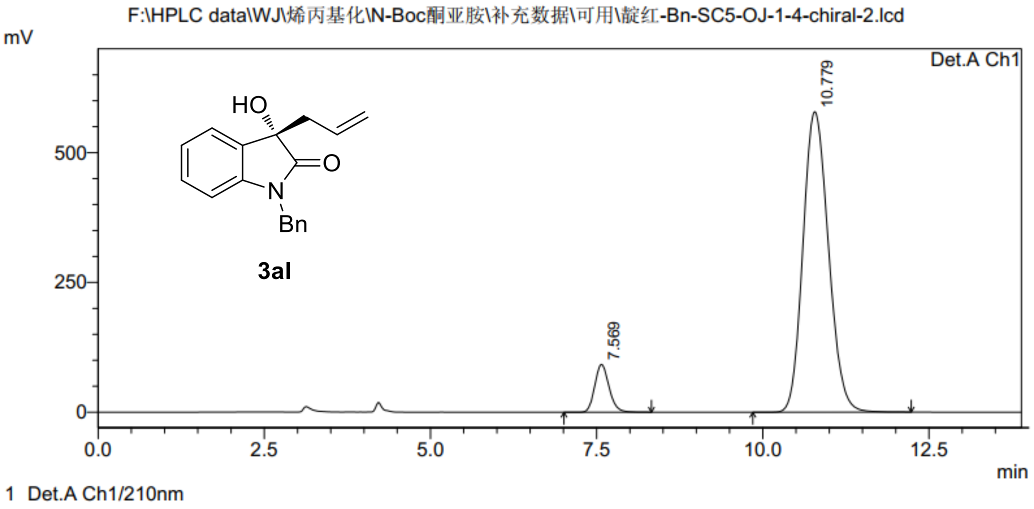

PeakTable

Detector A Ch1 210nm

Peak#	Ret. Time	Area	Height	Area %	Height %
1	7.569	1371310	92090	8.163	13.728
2	10.779	15428391	578743	91.837	86.272
Total		16799702	670833	100.000	100.000

Figure S127. HPLC spectrum of **3al**, related to **Scheme 2.**

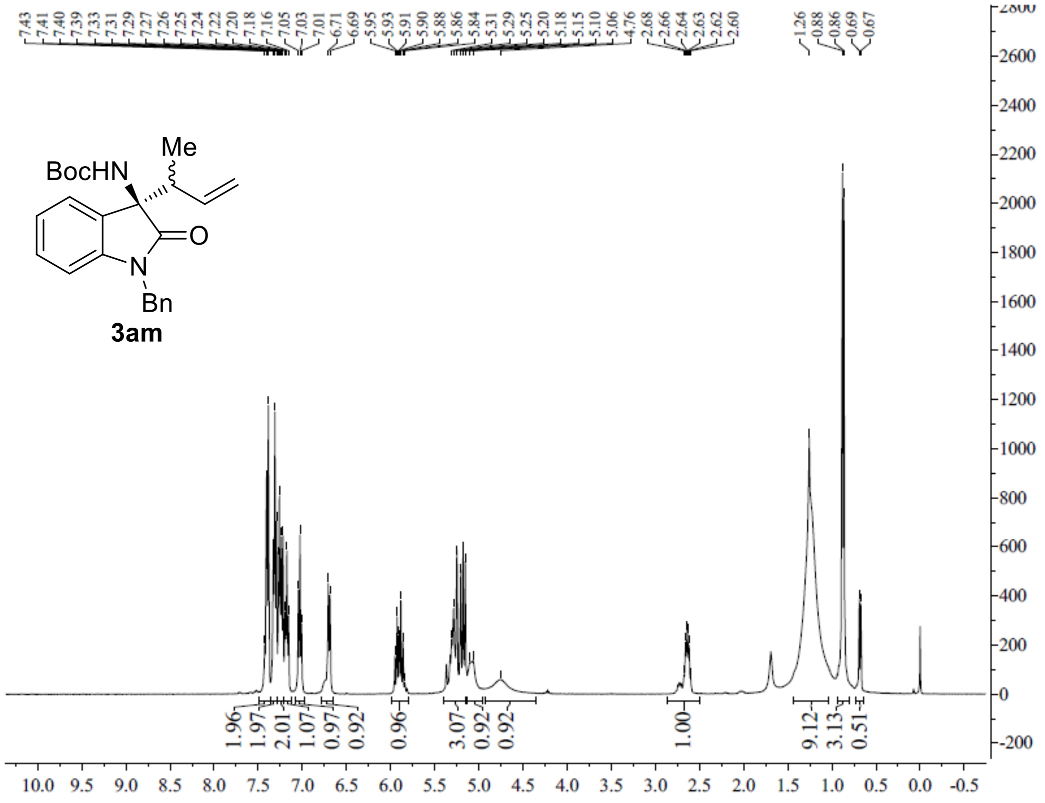

Figure S128. ^{1}H NMR spectrum of **3am**, related to **Scheme 2**.

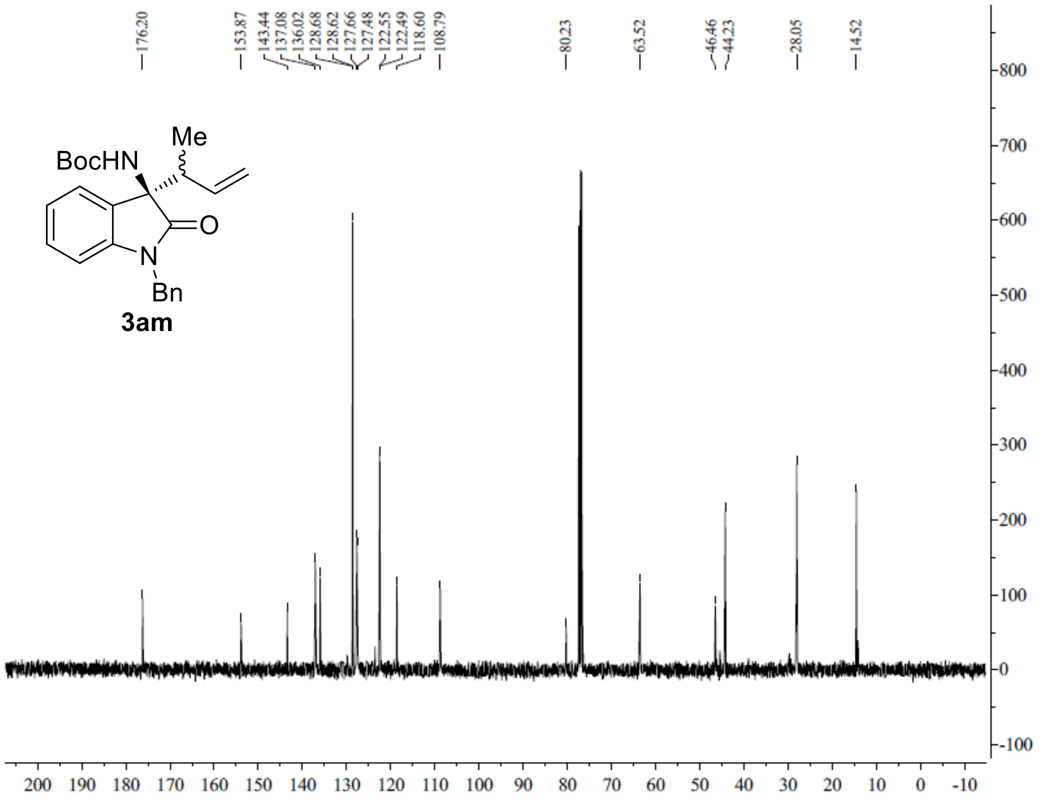

Figure S129. ^{13}C NMR spectrum of **3am**, related to **Scheme 2.**

<Chromatogram>

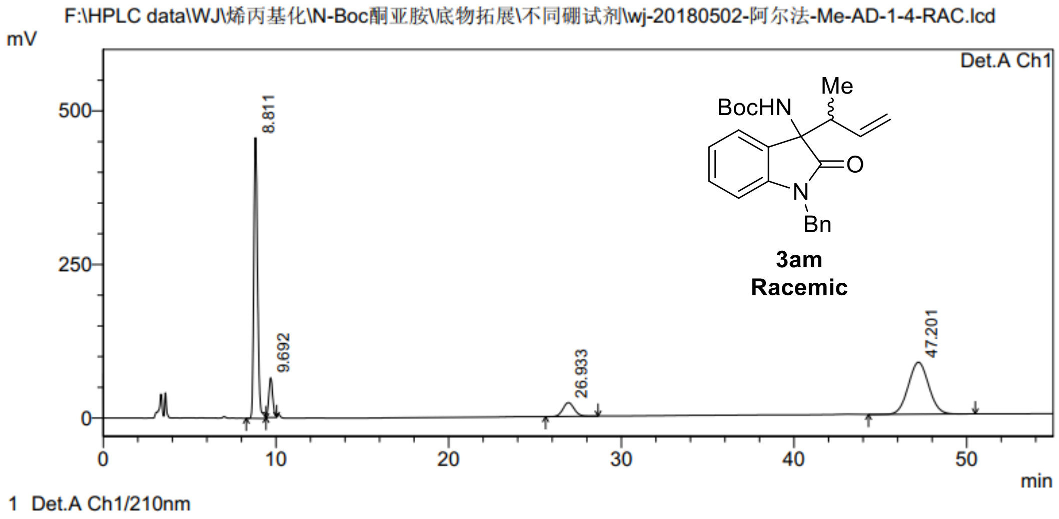

PeakTable

Detector A Ch1 210nm

Peak#	Ret. Time	Area	Height	Area %	Height %
1	8.811	6872392	457171	43.449	72.722
2	9.692	1013763	64527	6.409	10.264
3	26.933	1013573	22430	6.408	3.568
4	47.201	6917272	84528	43.733	13.446
Total		15817001	628655	100.000	100.000

<Chromatogram>

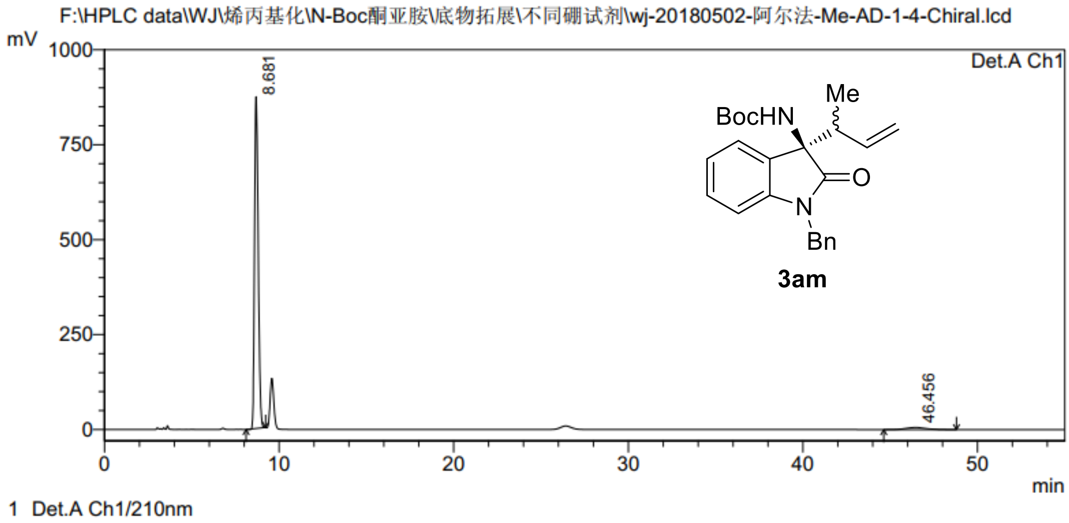

PeakTable

Detector A Ch1 210nm

Peak#	Ret. Time	Area	Height	Area %	Height %
1	8.681	12857205	873663	96.577	99.358
2	46.456	455654	5646	3.423	0.642
Total		13312860	879309	100.000	100.000

Figure S130. HPLC spectrum of **3am**, related to **Scheme 2.**

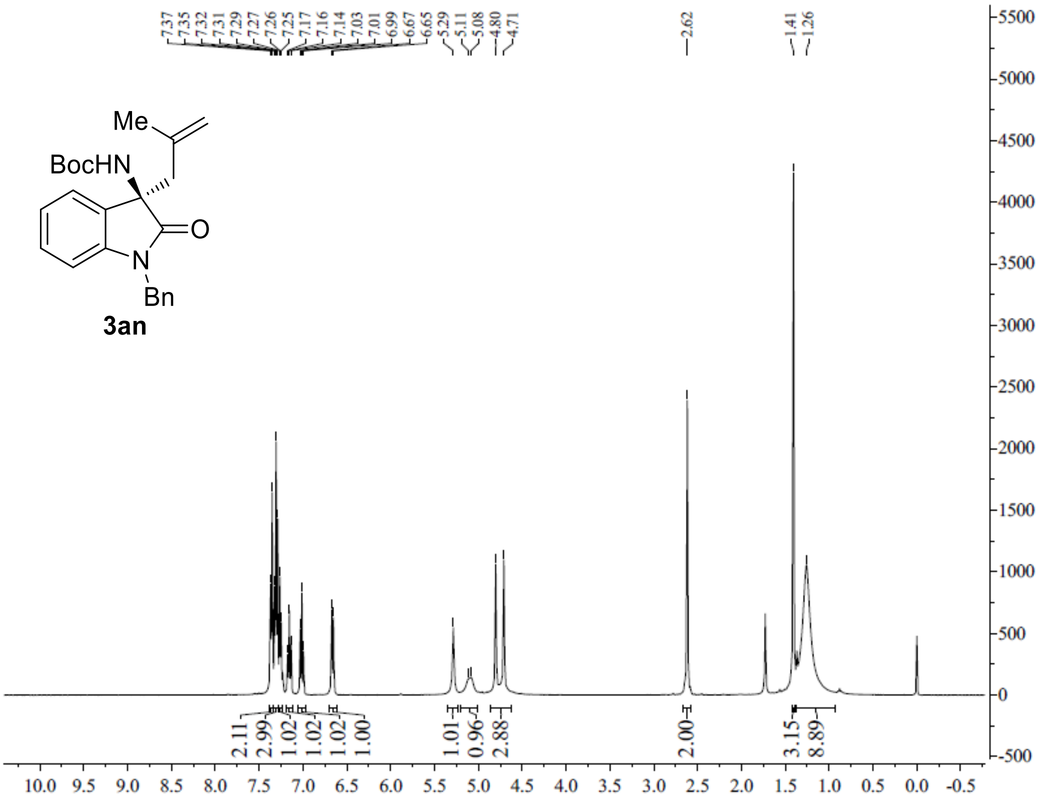

Figure S131. ^{1}H NMR spectrum of **3**an, related to **Scheme 2**.

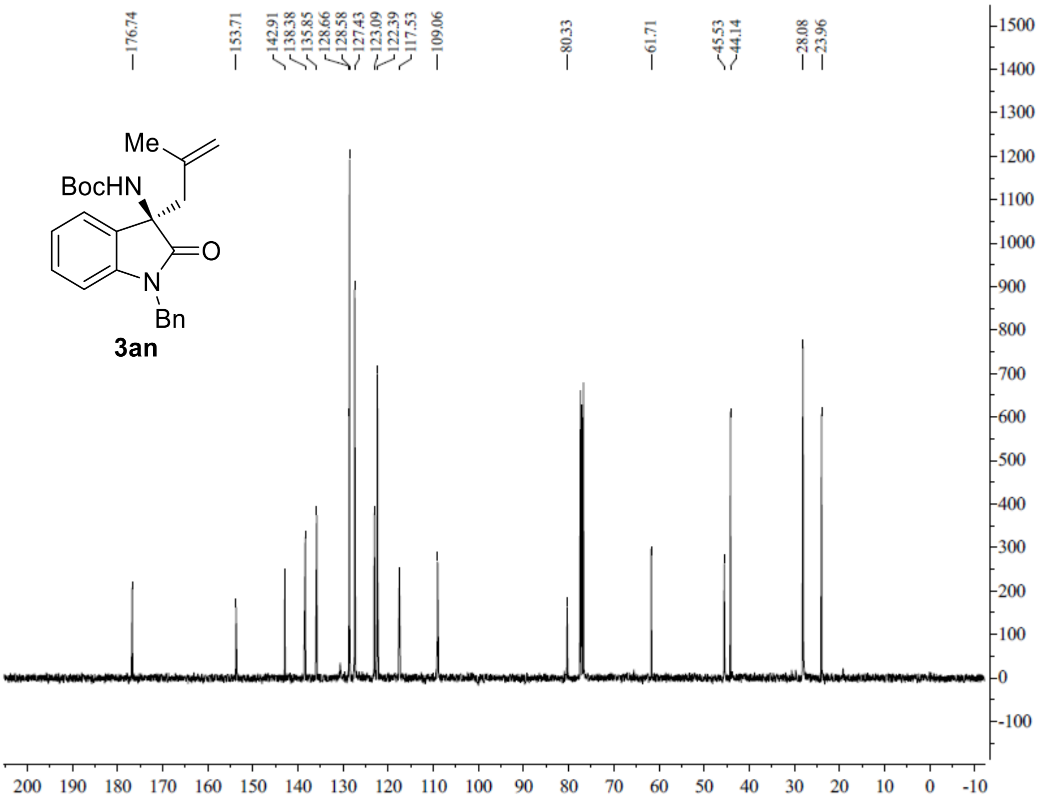

Figure S132. ^{13}C NMR spectrum of **3an**, related to **Scheme 2.**

<Chromatogram>

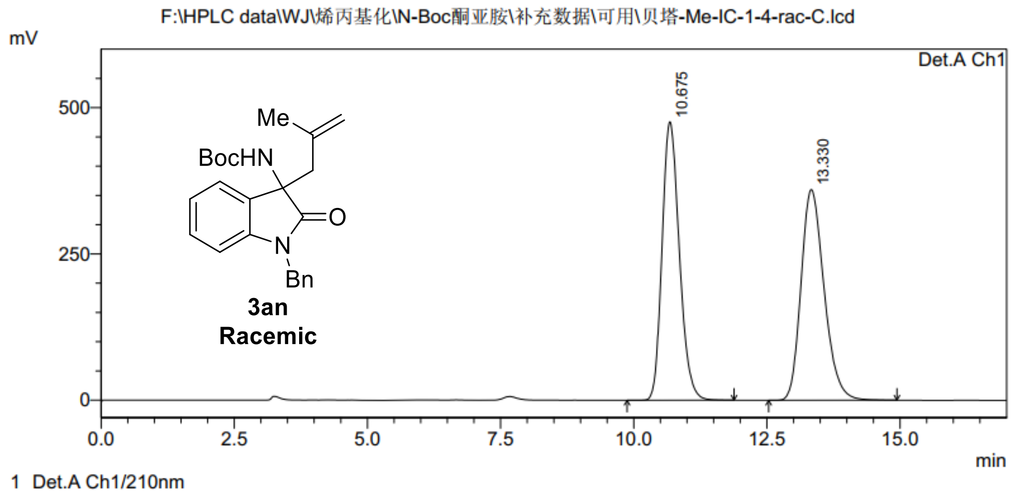

PeakTable

Detector A Ch1 210nm

Peak#	Ret. Time	Area	Height	Area %	Height %
1	10.675	10731003	475922	49.713	56.914
2	13.330	10854700	360287	50.287	43.086
Total		21585703	836209	100.000	100.000

<Chromatogram>

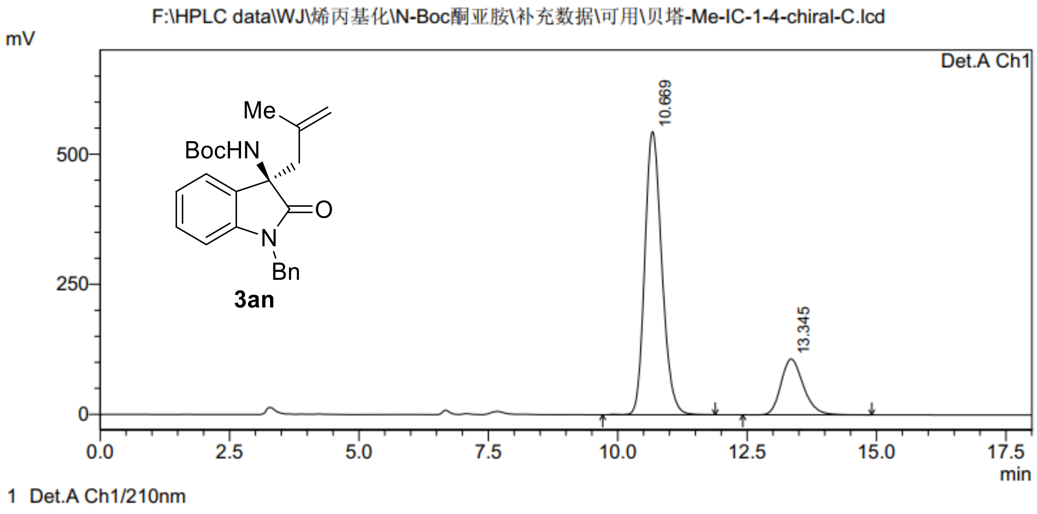

PeakTable

Detector A Ch1 210nm

Peak#	Ret. Time	Area	Height	Area %	Height %
1	10.669	12447697	544324	79.426	83.528
2	13.345	3224450	107339	20.574	16.472
Total		15672147	651663	100.000	100.000

Figure S133. HPLC spectrum of **3an**, related to **Scheme 2.**

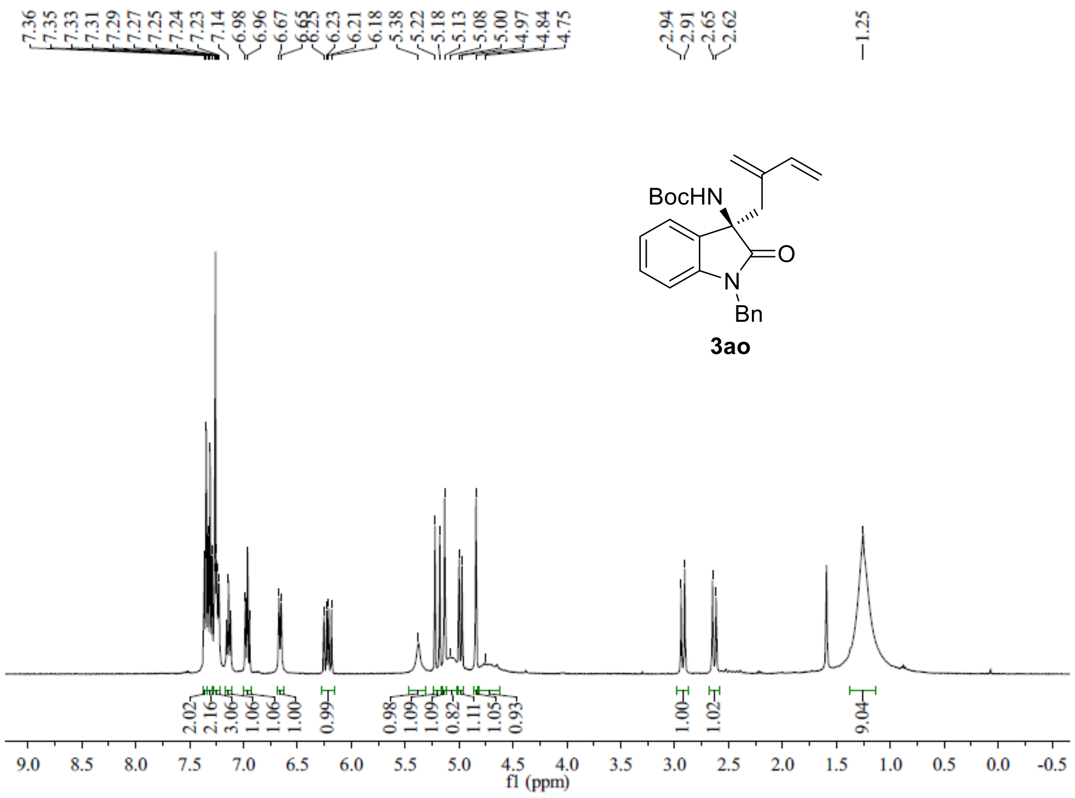

Figure S134. ^{1}H NMR spectrum of **3**ao, related to **Scheme 2**.

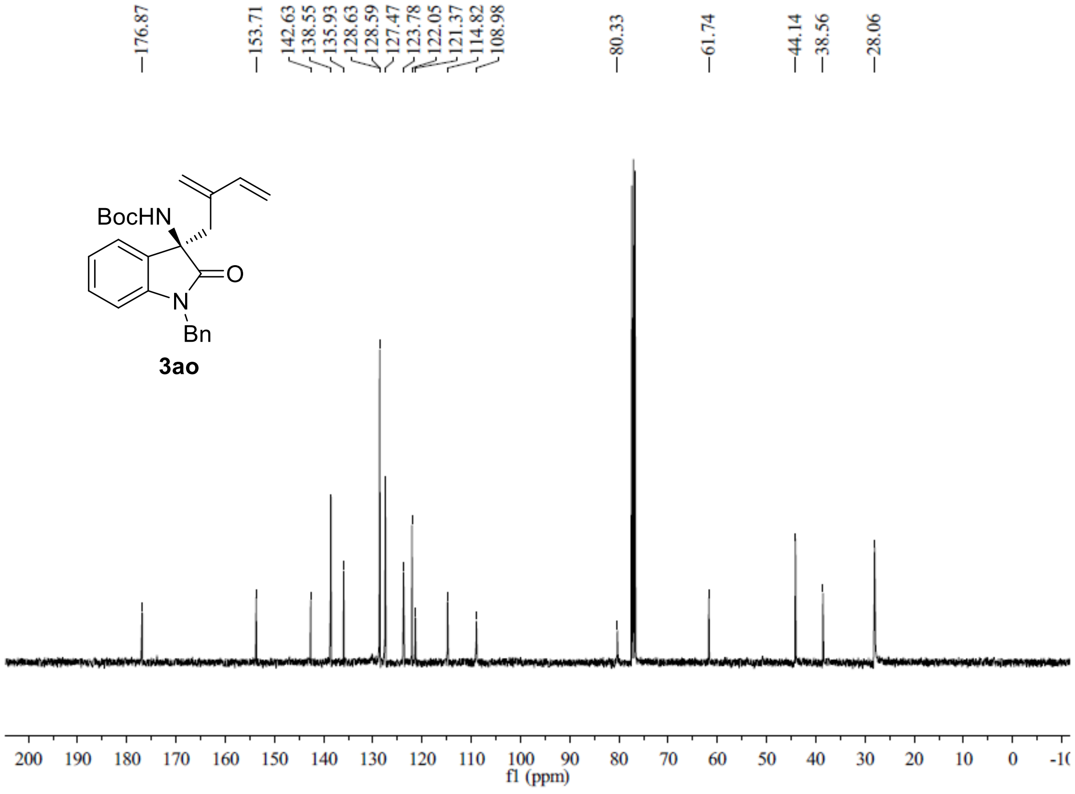

Figure S135. ^{13}C NMR spectrum of **3ao**, related to **Scheme 2.**

<Chromatogram>

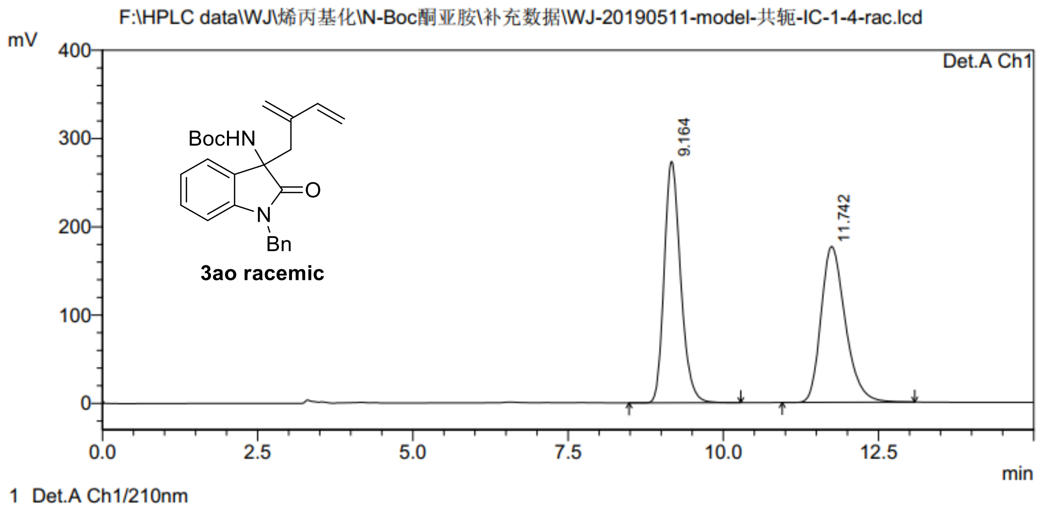

PeakTable

Detector A Ch1 210nm

Peak#	Ret. Time	Area	Height	Area %	Height %
1	9.164	5028532	273419	51.096	60.762
2	11.742	4812733	176566	48.904	39.238
Total		9841265	449985	100.000	100.000

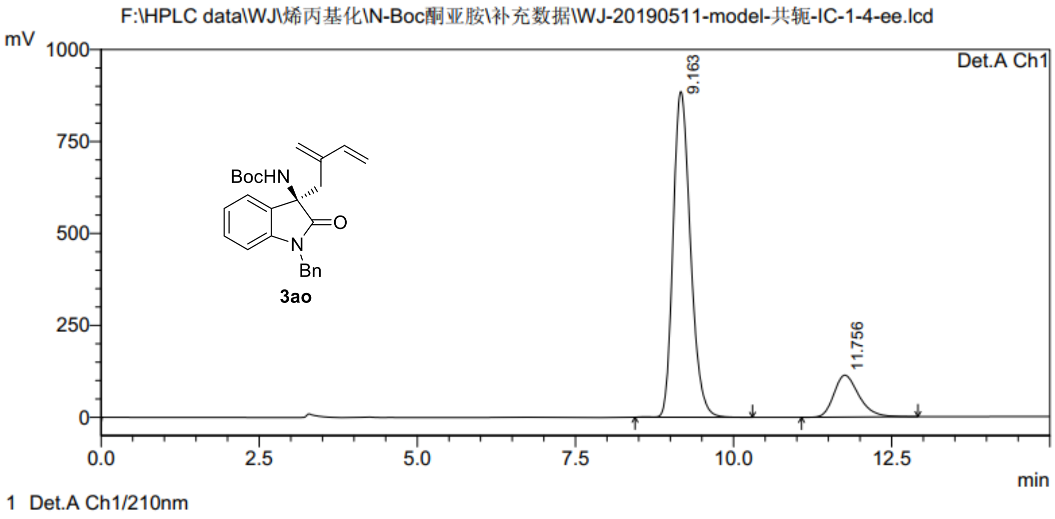

PeakTable

Detector A Ch1 210nm

Peak#	Ret. Time	Area	Height	Area %	Height %
1	9.163	16924549	885990	84.425	88.615
2	11.756	3122252	113831	15.575	11.385
Total		20046801	999821	100.000	100.000

Figure S136. HPLC spectrum of **3ao**, related to **Scheme 2.**

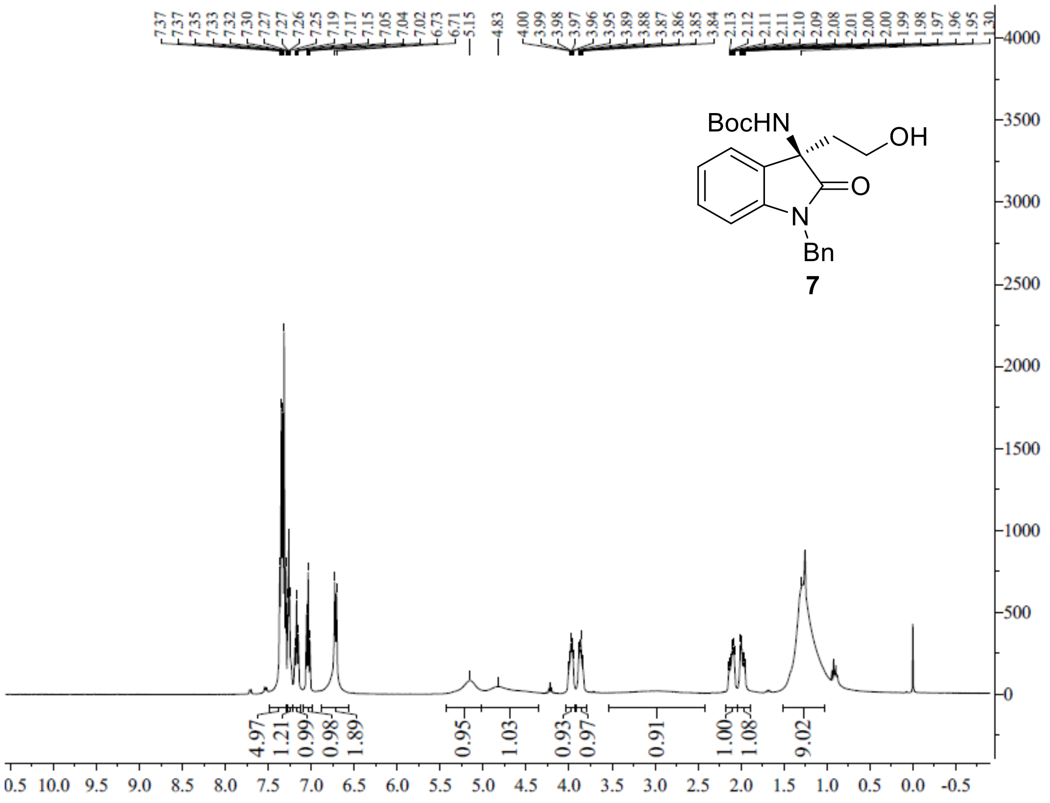

Figure S137. ^{1}H NMR spectrum of **7**, related to **Scheme 3**.

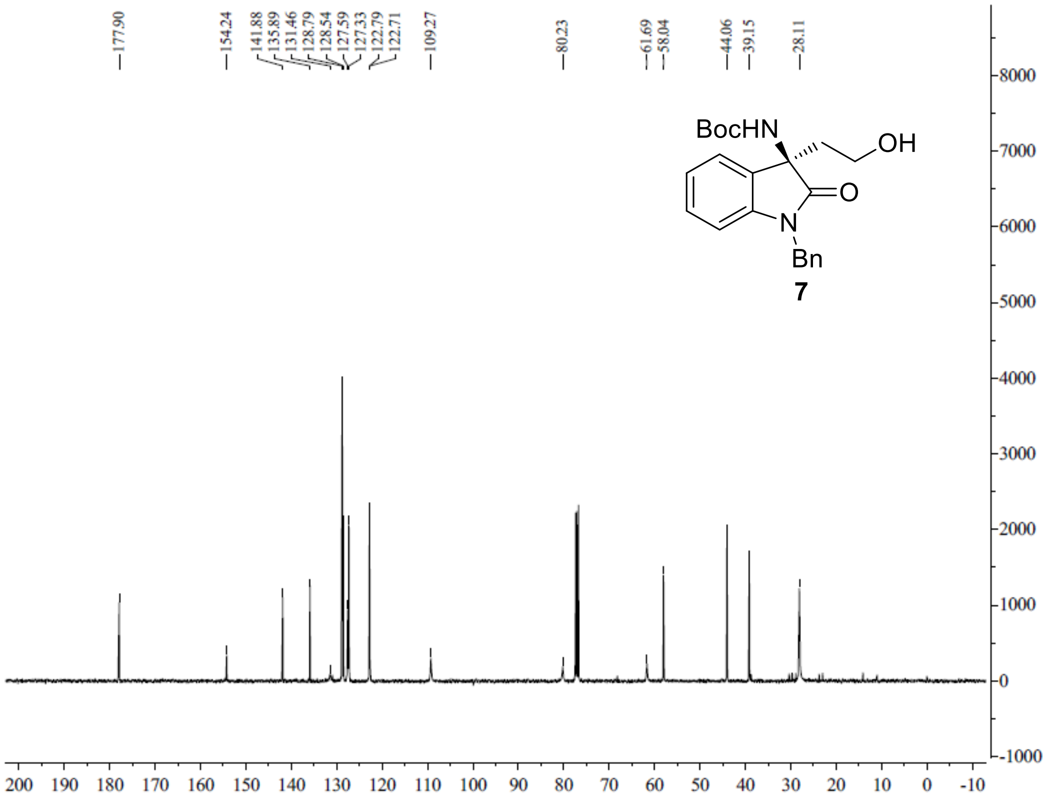

Figure S138. ^{13}C NMR spectrum of **7**, related to **Scheme 3.**

<Chromatogram>

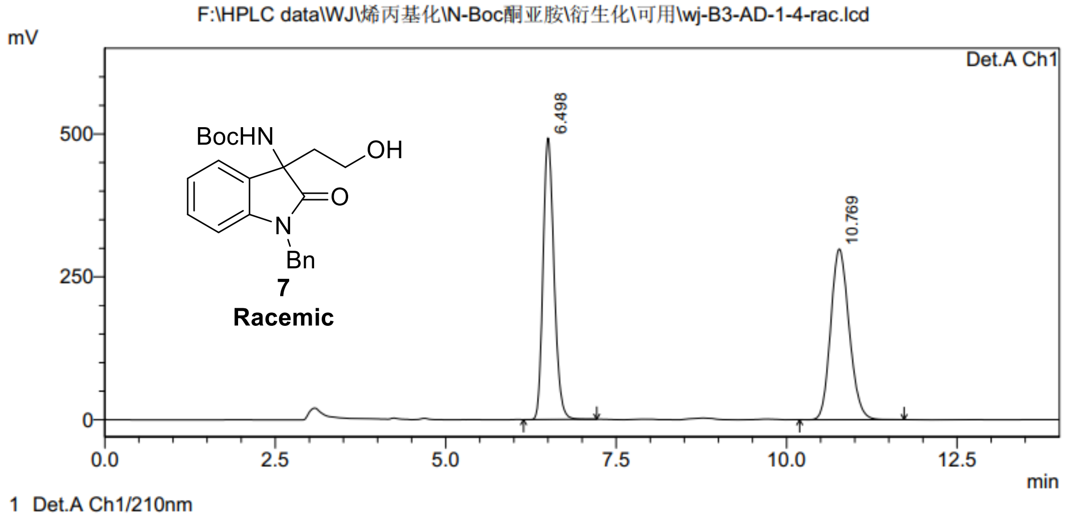

PeakTable

Detector A Ch1 210nm

Peak#	Ret. Time	Area	Height	Area %	Height %
1	6.498	5482902	492695	49.283	62.257
2	10.769	5642509	298699	50.717	37.743
Total		11125412	791394	100.000	100.000

<Chromatogram>

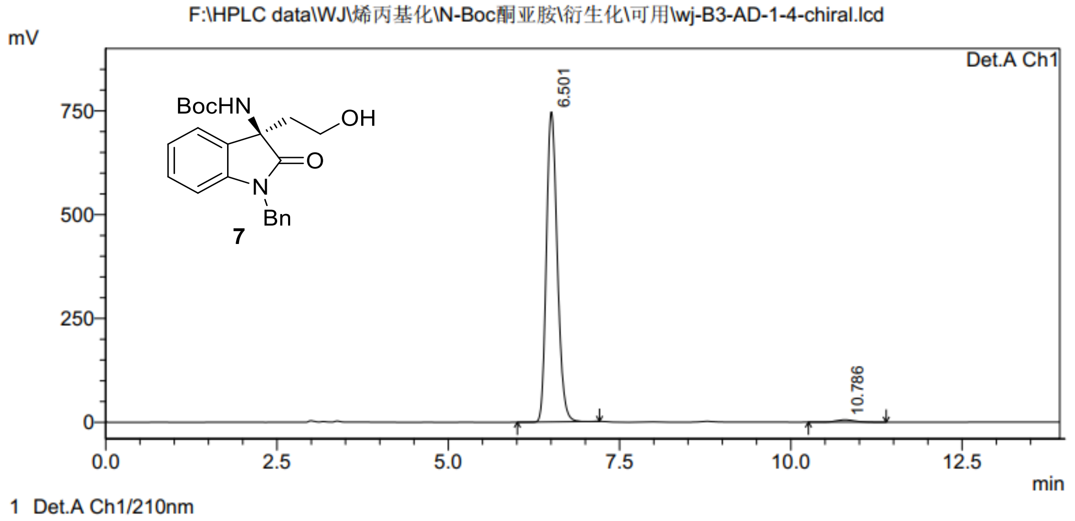

PeakTable

Detector A Ch1 210nm

Peak#	Ret. Time	Area	Height	Area %	Height %
1	6.501	8217489	746423	98.792	99.274
2	10.786	100485	5457	1.208	0.726
Total		8317974	751880	100.000	100.000

Figure S139. HPLC spectrum of **7**, related to **Scheme 3.**

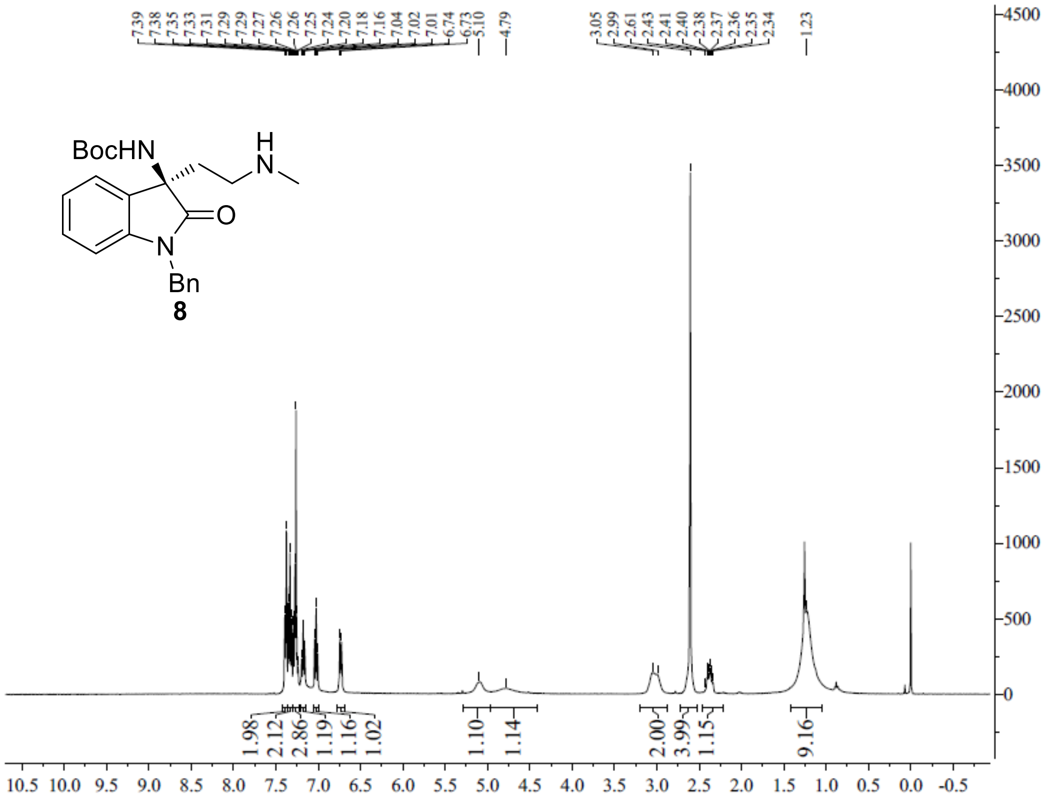

Figure S140. ^{1}H NMR spectrum of **8**, related to **Scheme 3**.

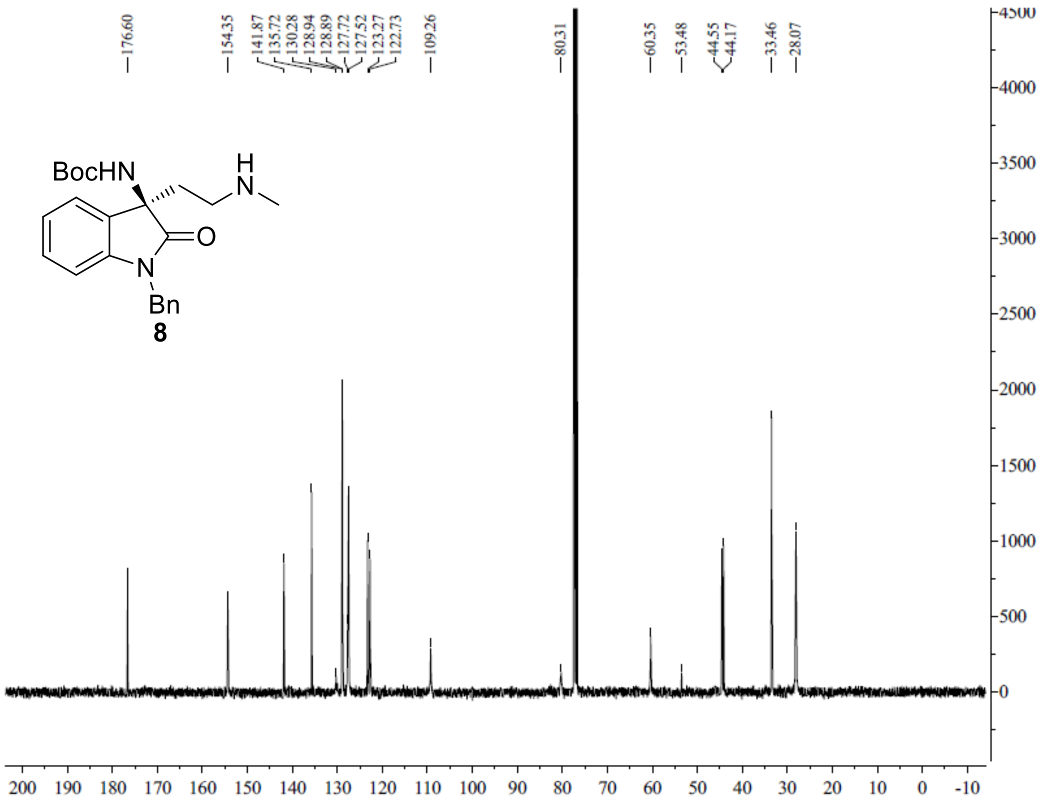

Figure S141. ^{13}C NMR spectrum of **8**, related to **Scheme 3.**

<Chromatogram>

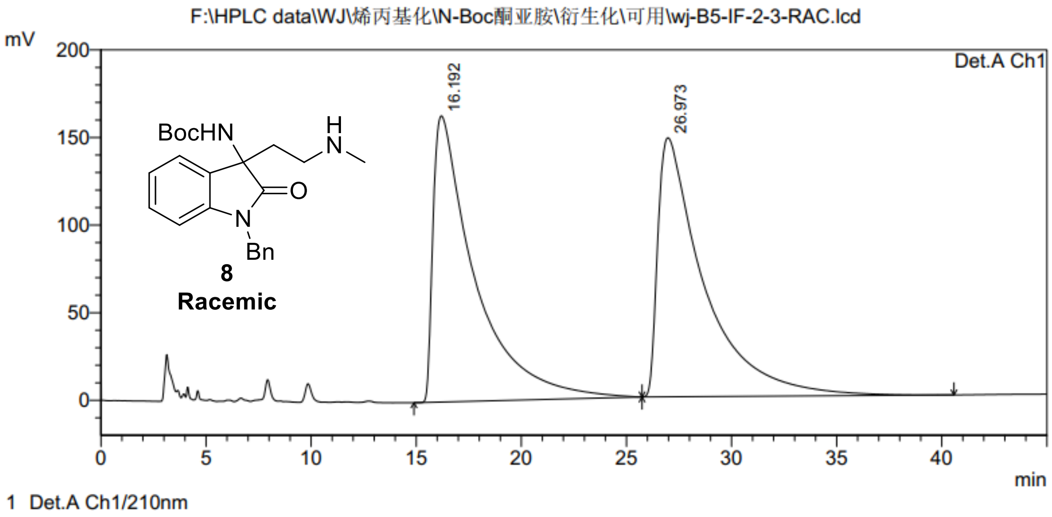

PeakTable

Detector A Ch1 210nm

Peak#	Ret. Time	Area	Height	Area %	Height %
1	16.192	22532452	163289	49.924	52.481
2	26.973	22601187	147850	50.076	47.519
Total		45133639	311139	100.000	100.000

<Chromatogram>

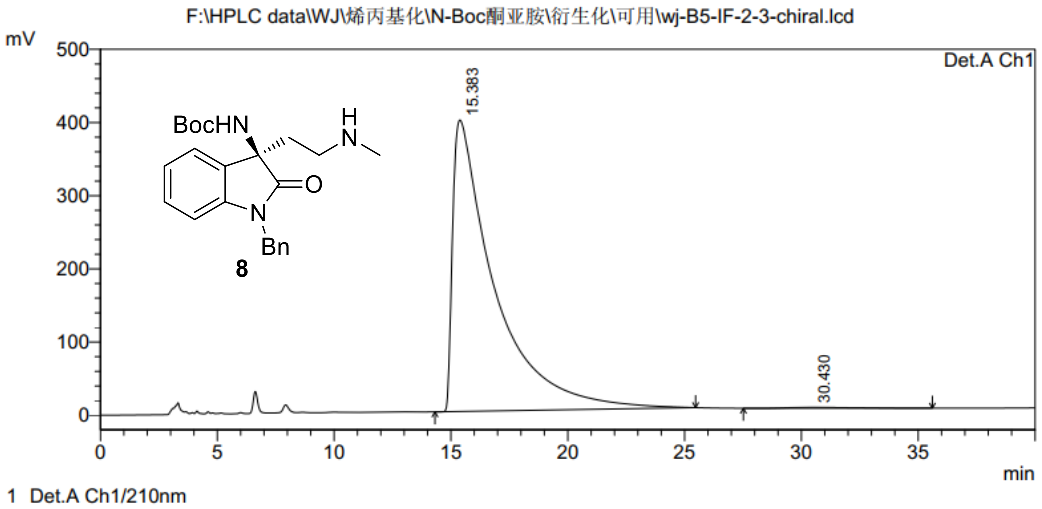

PeakTable

Detector A Ch1 210nm

Peak#	Ret. Time	Area	Height	Area %	Height %
1	15.383	49498257	398168	99.546	99.703
2	30.430	225843	1185	0.454	0.297
Total		49724100	399353	100.000	100.000

Figure S142. HPLC spectrum of **8**, related to **Scheme 3.**

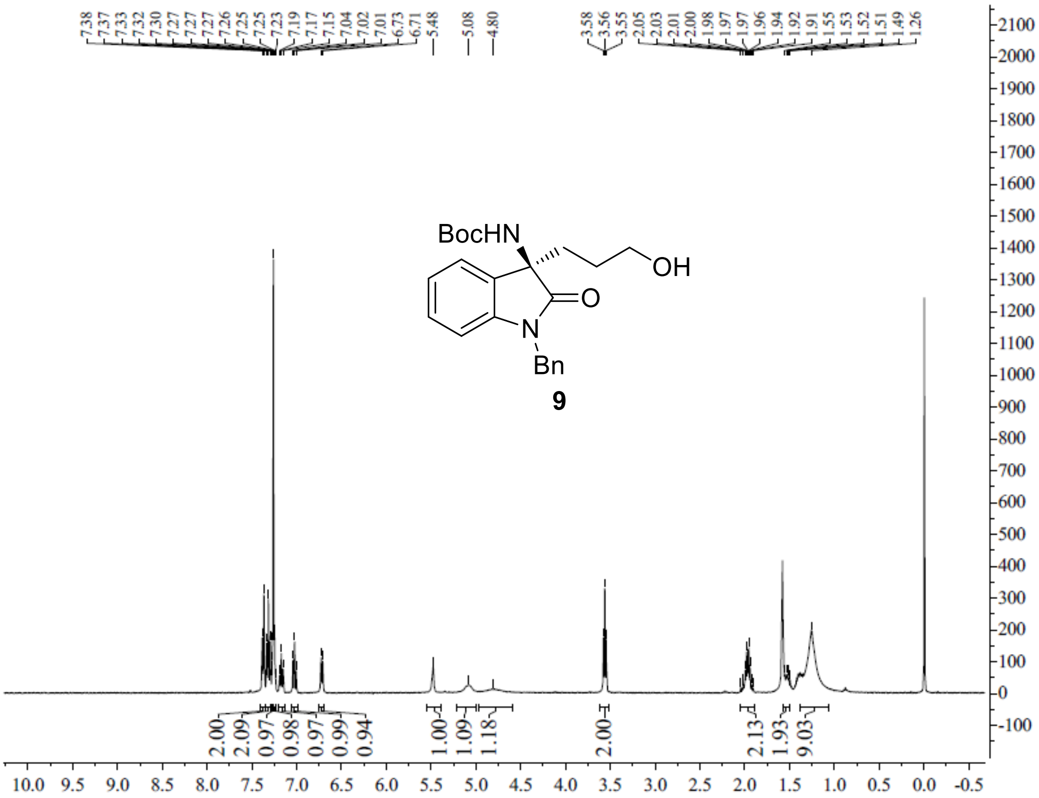

Figure S143. ^{1}H NMR spectrum of **9**, related to **Scheme 3**.

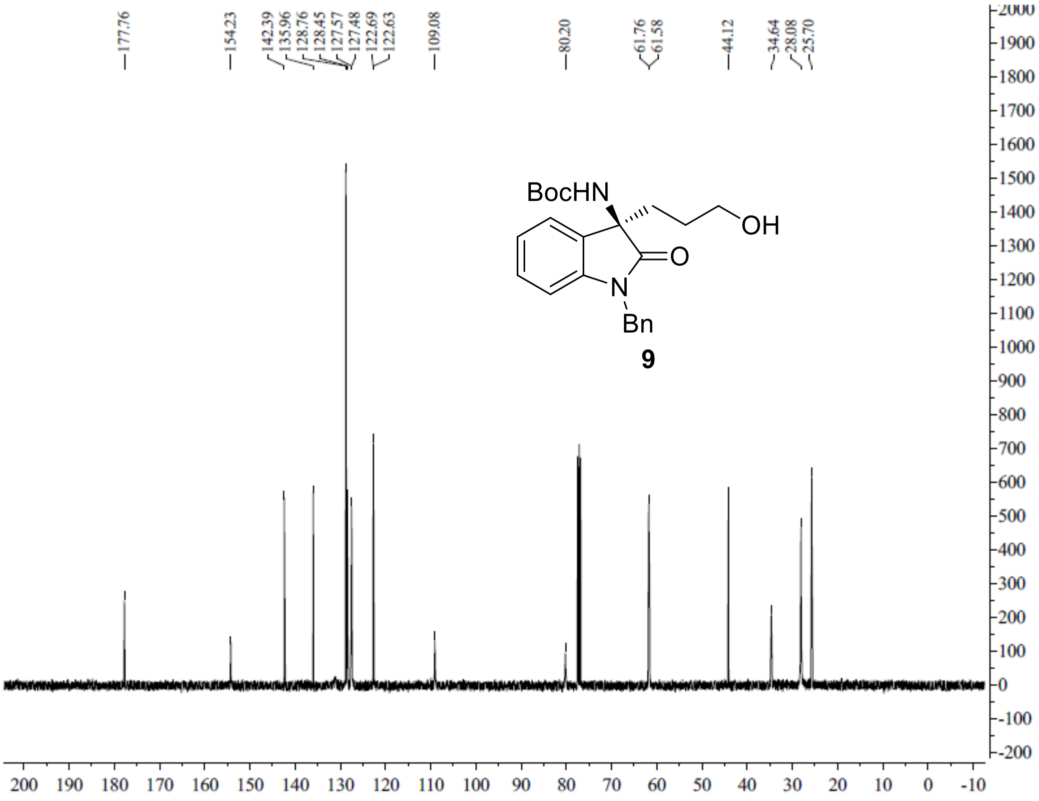

Figure S144. ^{13}C NMR spectrum of **9**, related to **Scheme 3.**

<Chromatogram>

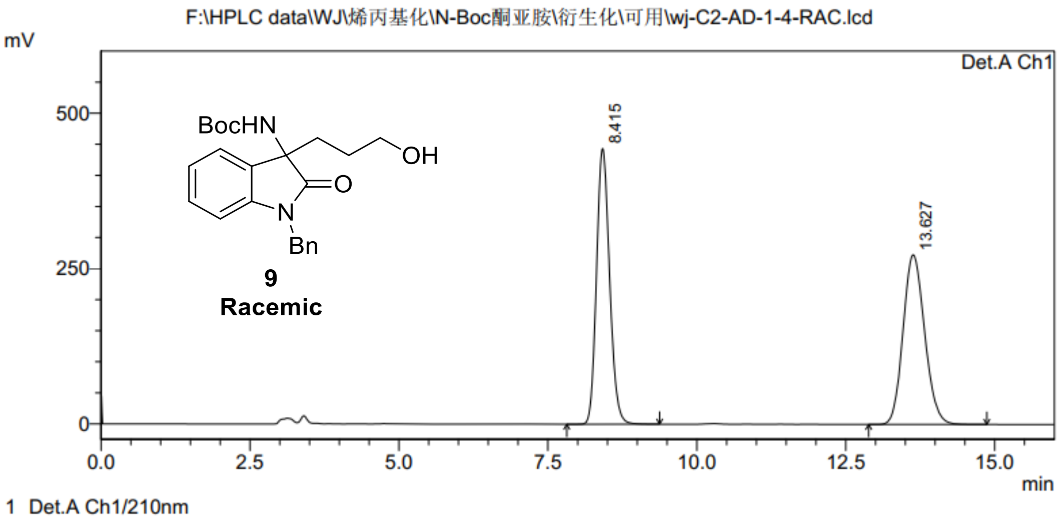

PeakTable

Detector A Ch1 210nm

Peak#	Ret. Time	Area	Height	Area %	Height %
1	8.415	6775027	443763	49.663	61.909
2	13.627	6867032	273032	50.337	38.091
Total		13642059	716795	100.000	100.000

<Chromatogram>

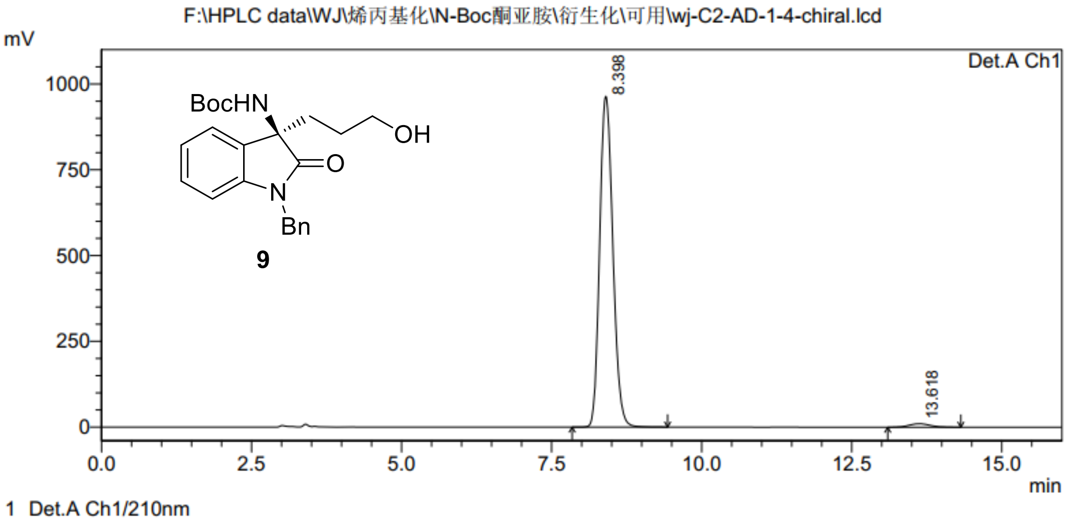

PeakTable

Detector A Ch1 210nm

Peak#	Ret. Time	Area	Height	Area %	Height %
1	8.398	14757628	964440	98.342	98.948
2	13.618	248755	10258	1.658	1.052
Total		15006383	974699	100.000	100.000

Figure S145. HPLC spectrum of **9**, related to **Scheme 3.**

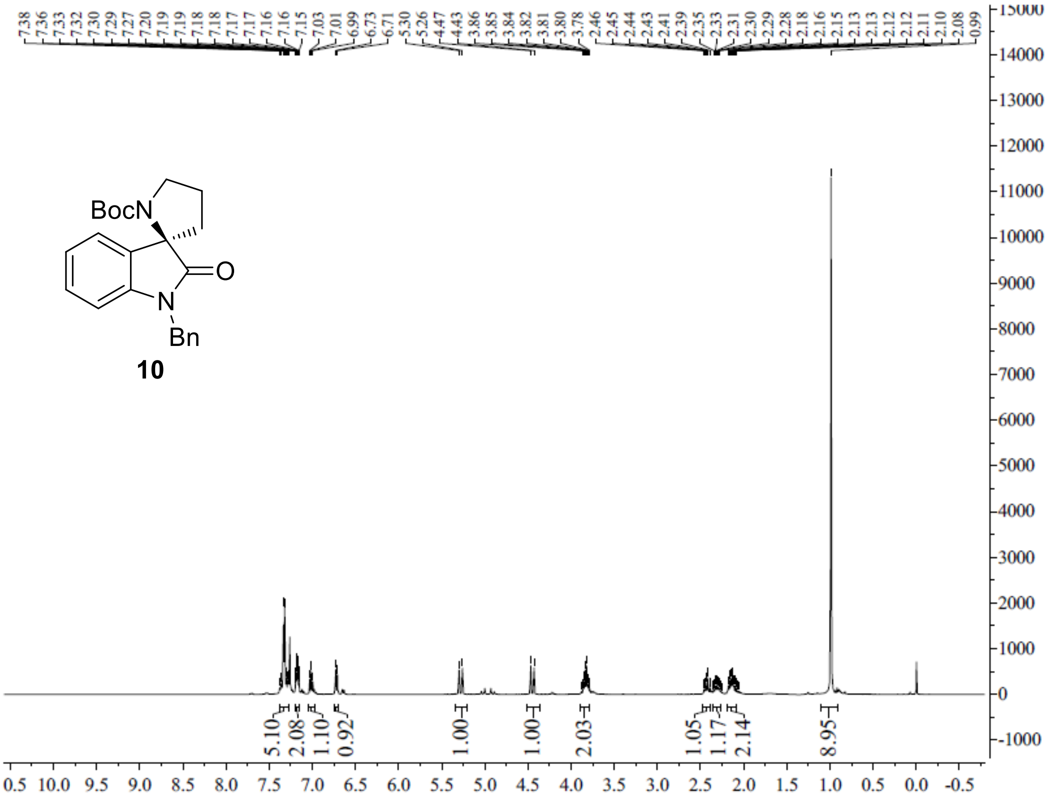

Figure S146. ^{1}H NMR spectrum of **10**, related to **Scheme 3**.

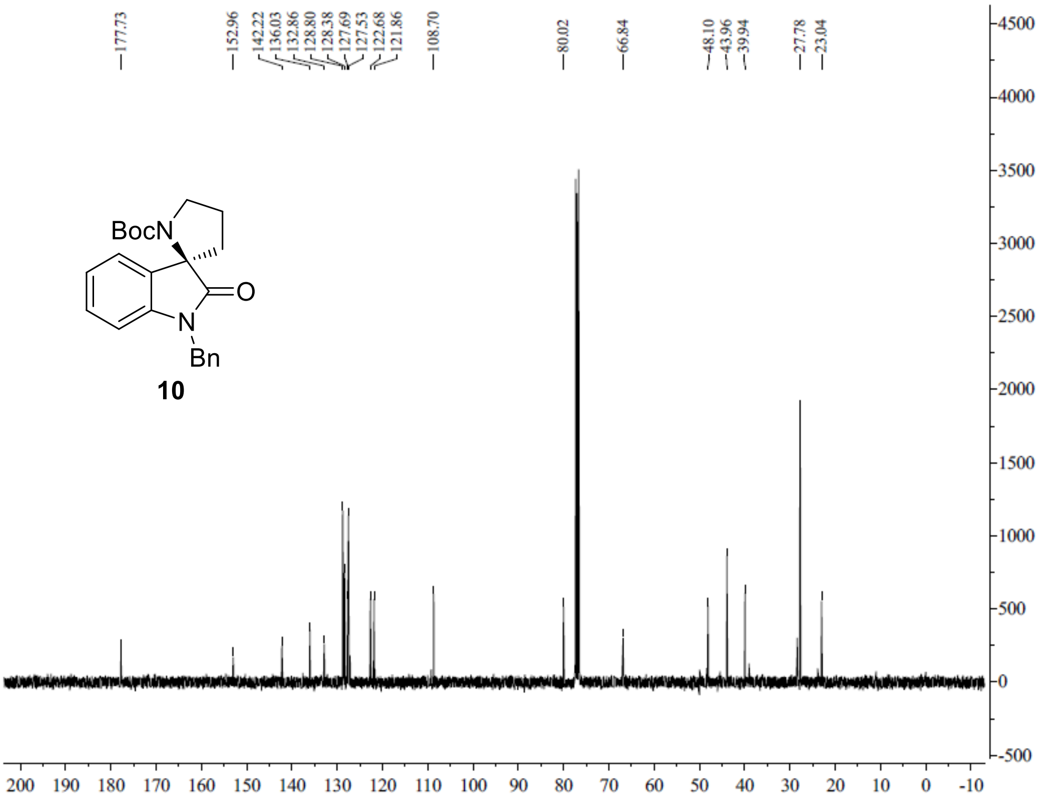

Figure S147. ^{13}C NMR spectrum of **10**, related to **Scheme 3.**

<Chromatogram>

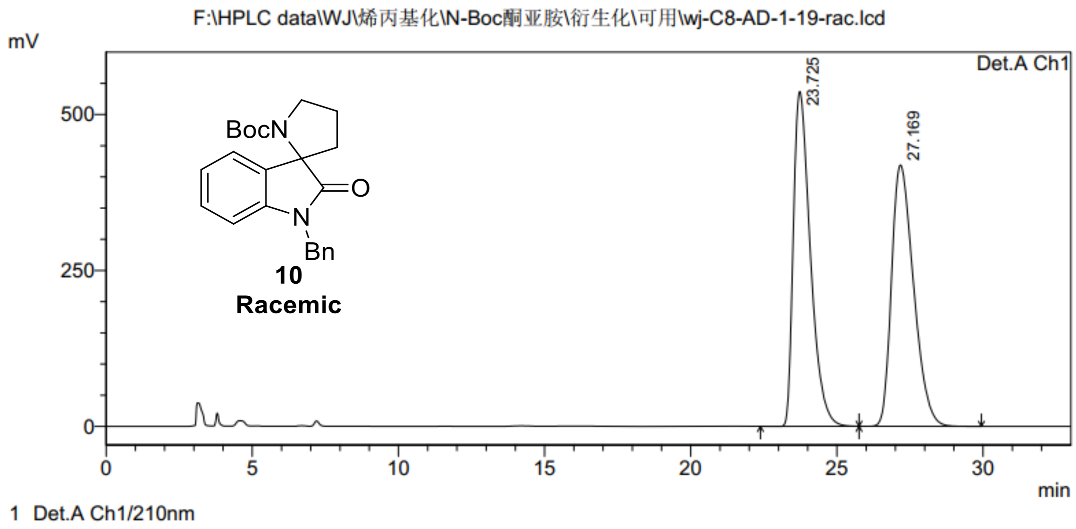

PeakTable

Detector A Ch1 210nm

Peak#	Ret. Time	Area	Height	Area %	Height %
1	23.725	21711394	536757	49.744	56.165
2	27.169	21934993	418930	50.256	43.835
Total		43646387	955686	100.000	100.000

<Chromatogram>

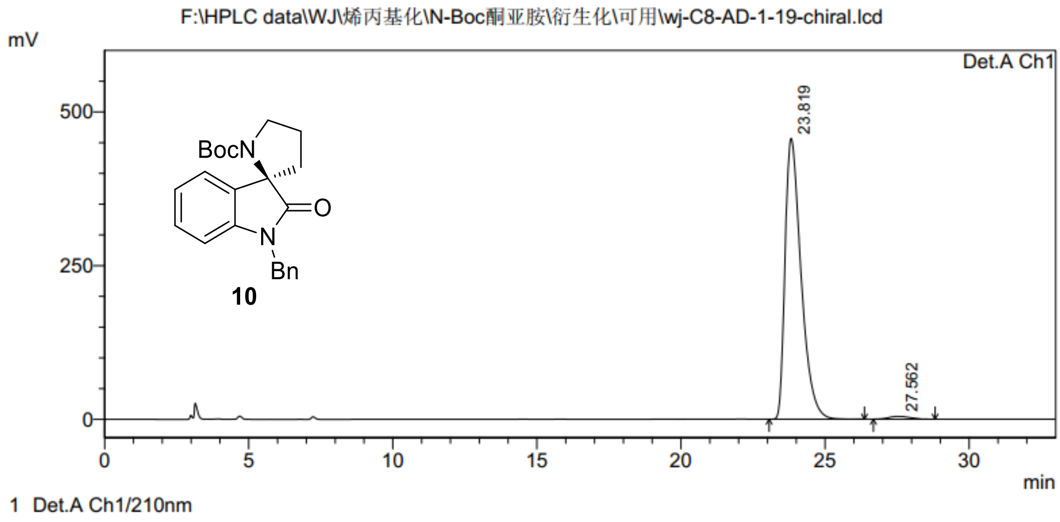

PeakTable

Detector A Ch1 210nm

Peak#	Ret. Time	Area	Height	Area %	Height %
1	23.819	17635779	456928	98.689	98.950
2	27.562	234259	4850	1.311	1.050
Total		17870038	461778	100.000	100.000

Figure S148. HPLC spectrum of **10**, related to **Scheme 3.**

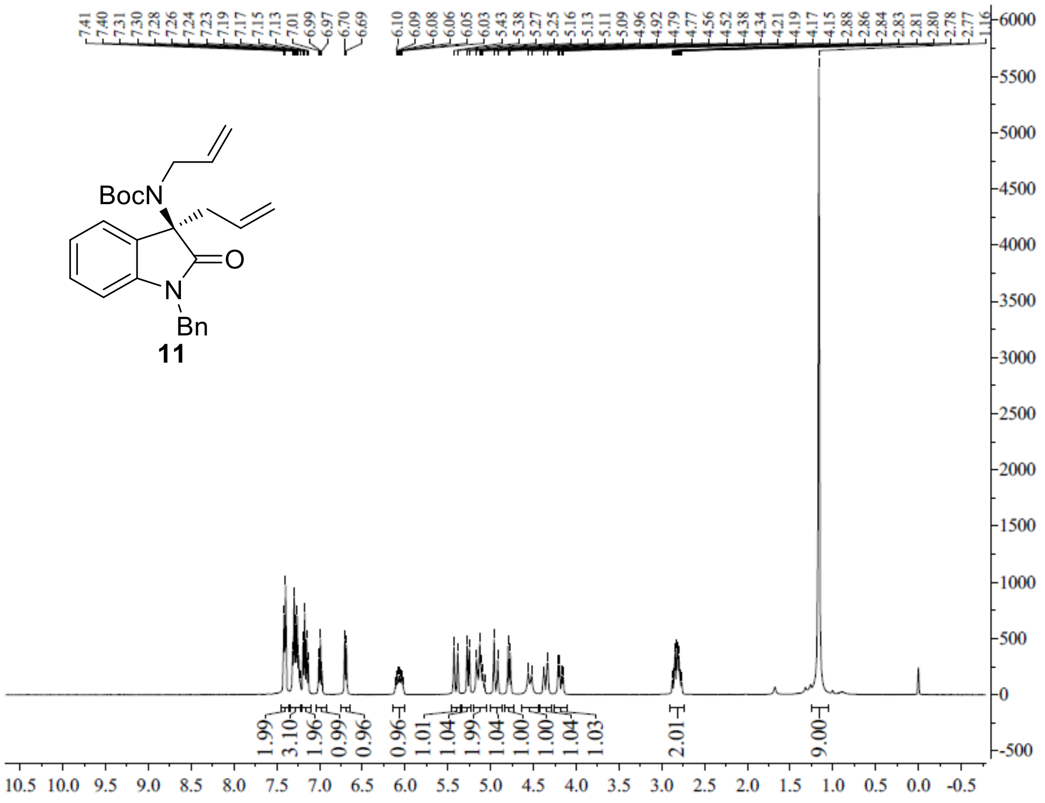

Figure S149. ^{1}H NMR spectrum of **11**, related to **Scheme 3**.

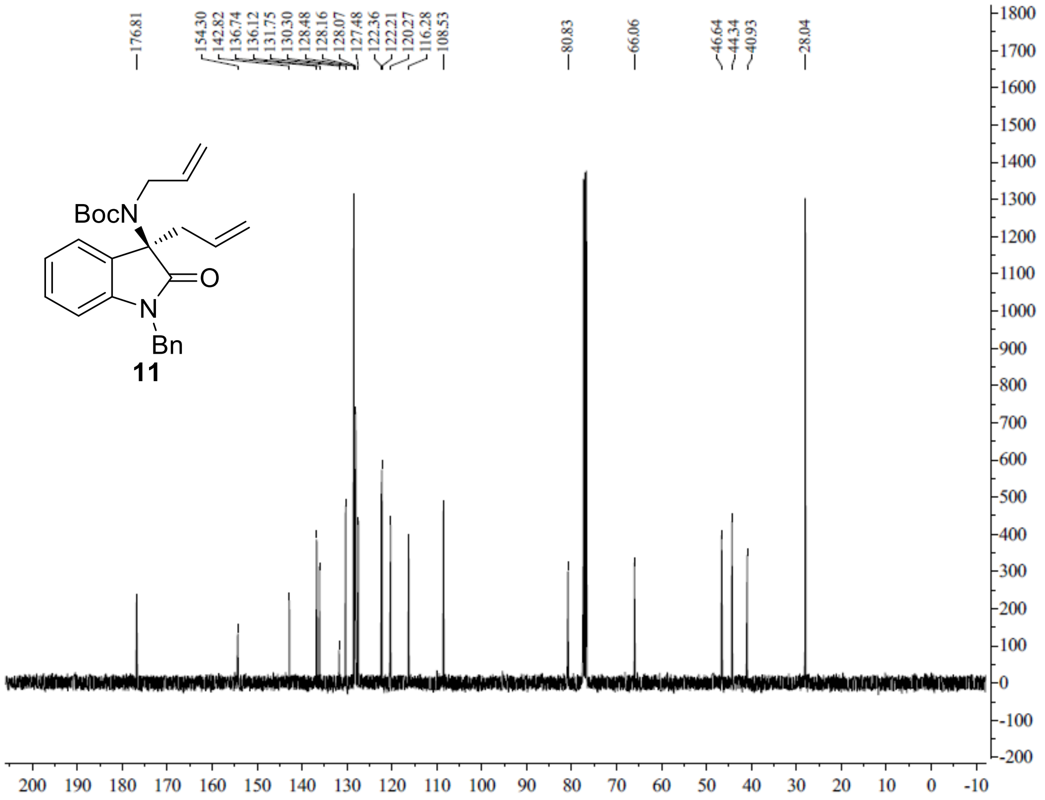

Figure S150. ^{13}C NMR spectrum of **11**, related to **Scheme 3.**

<Chromatogram>

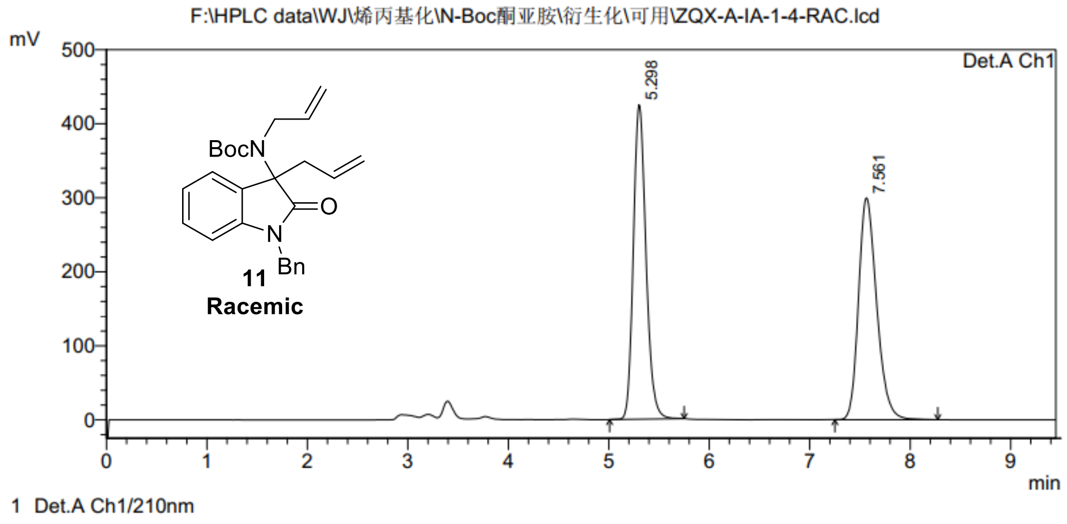

PeakTable

Detector A Ch1 210nm

Peak#	Ret. Time	Area	Height	Area %	Height %
1	5.298	3703224	424893	49.626	58.641
2	7.561	3759082	299677	50.374	41.359
Total		7462306	724570	100.000	100.000

<Chromatogram>

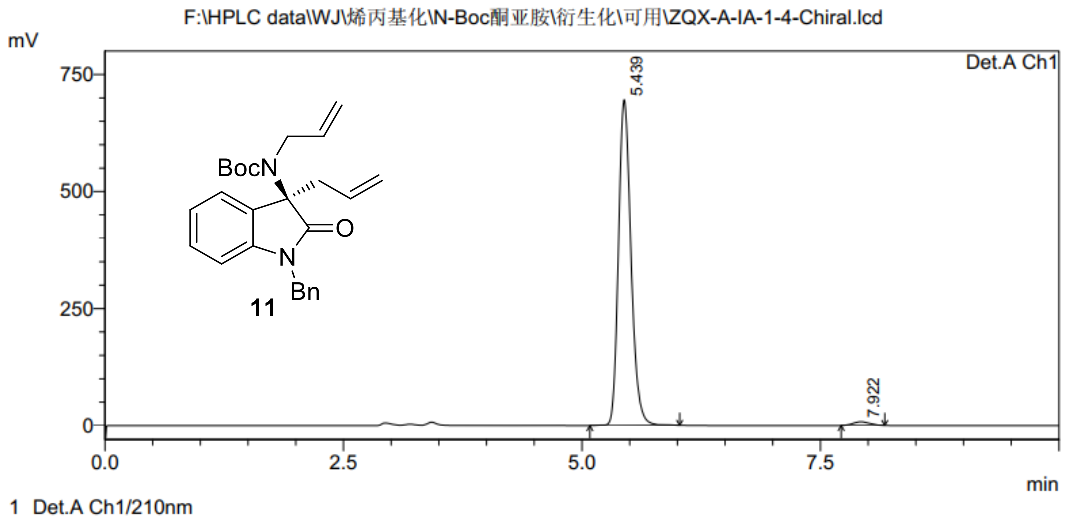

PeakTable

Detector A Ch1 210nm

Peak#	Ret. Time	Area	Height	Area %	Height %
1	5.439	6353429	695927	98.516	98.870
2	7.922	95734	7951	1.484	1.130
Total		6449164	703878	100.000	100.000

Figure S151. HPLC spectrum of **11**, related to **Scheme 3.**

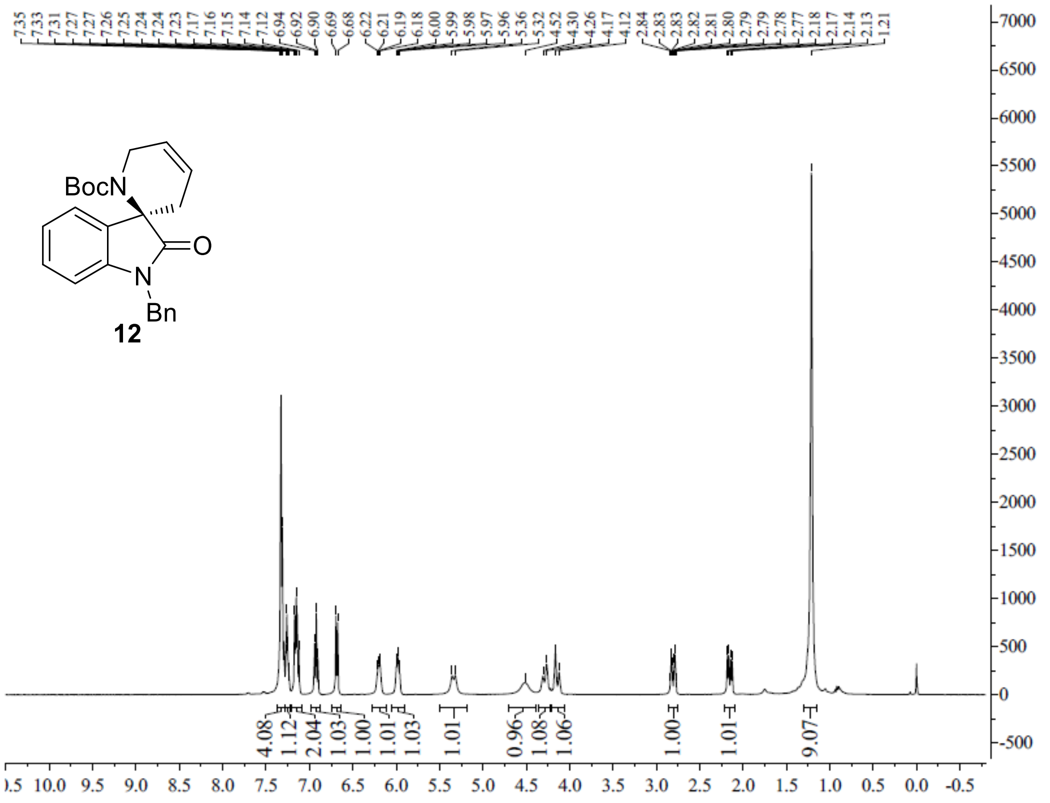

Figure S152. ^{1}H NMR spectrum of **12**, related to **Scheme 3**.

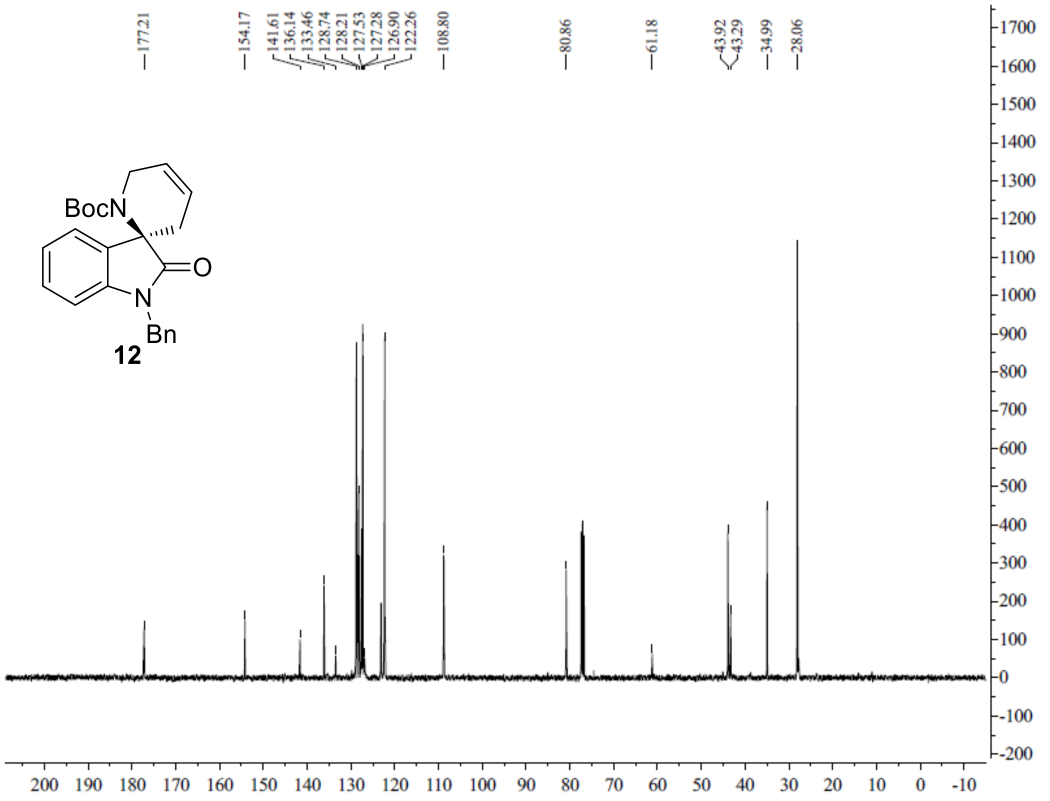

Figure S153. ^{13}C NMR spectrum of **12**, related to **Scheme 3.**

<Chromatogram>

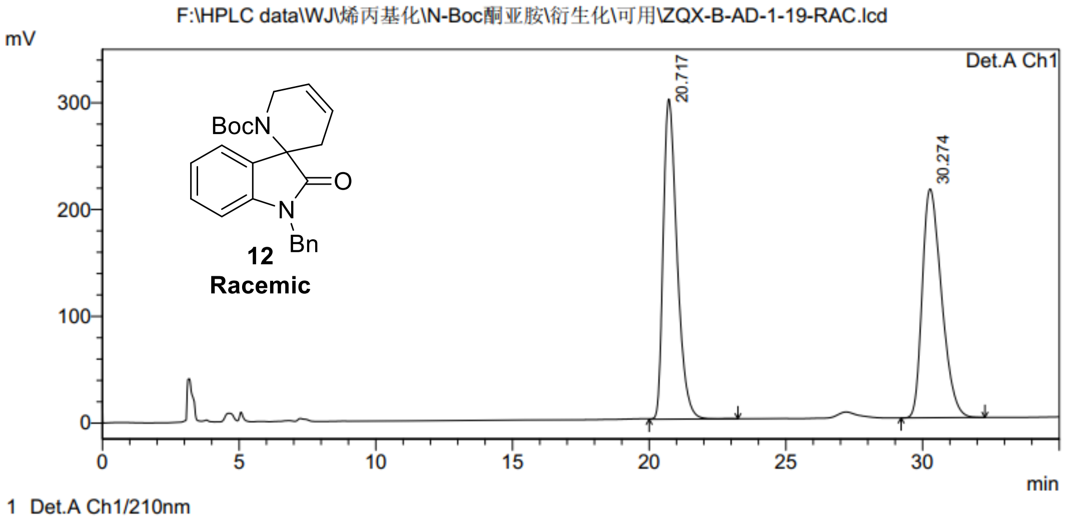

PeakTable

Detector A Ch1 210nm

Peak#	Ret. Time	Area	Height	Area %	Height %
1	20.717	10495228	299954	49.950	58.327
2	30.274	10516274	214309	50.050	41.673
Total		21011502	514263	100.000	100.000

<Chromatogram>

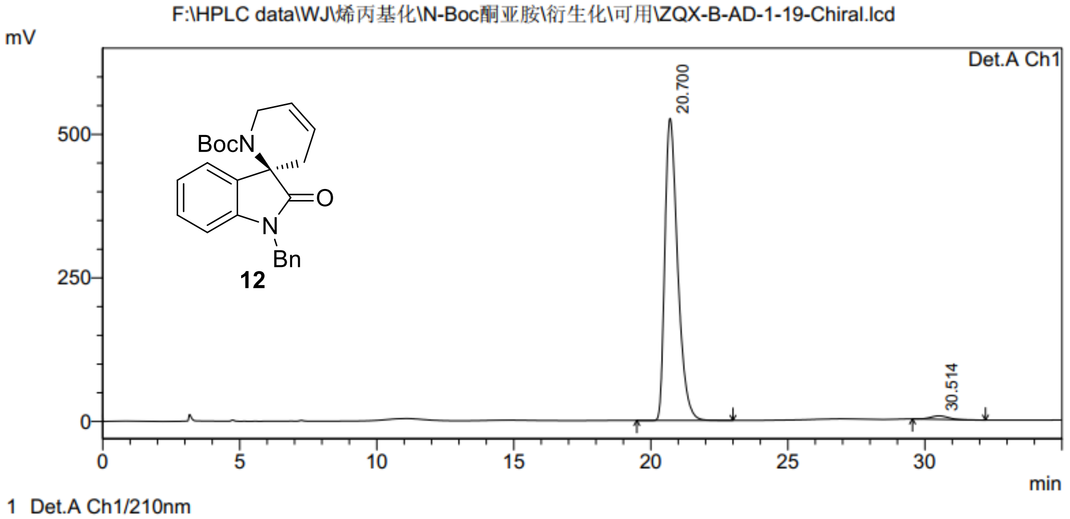

PeakTable

Detector A Ch1 210nm

Peak#	Ret. Time	Area	Height	Area %	Height %
1	20.700	17051550	526084	98.515	98.865
2	30.514	257011	6040	1.485	1.135
Total		17308561	532124	100.000	100.000

Figure S154. HPLC spectrum of **12**, related to **Scheme 3.**

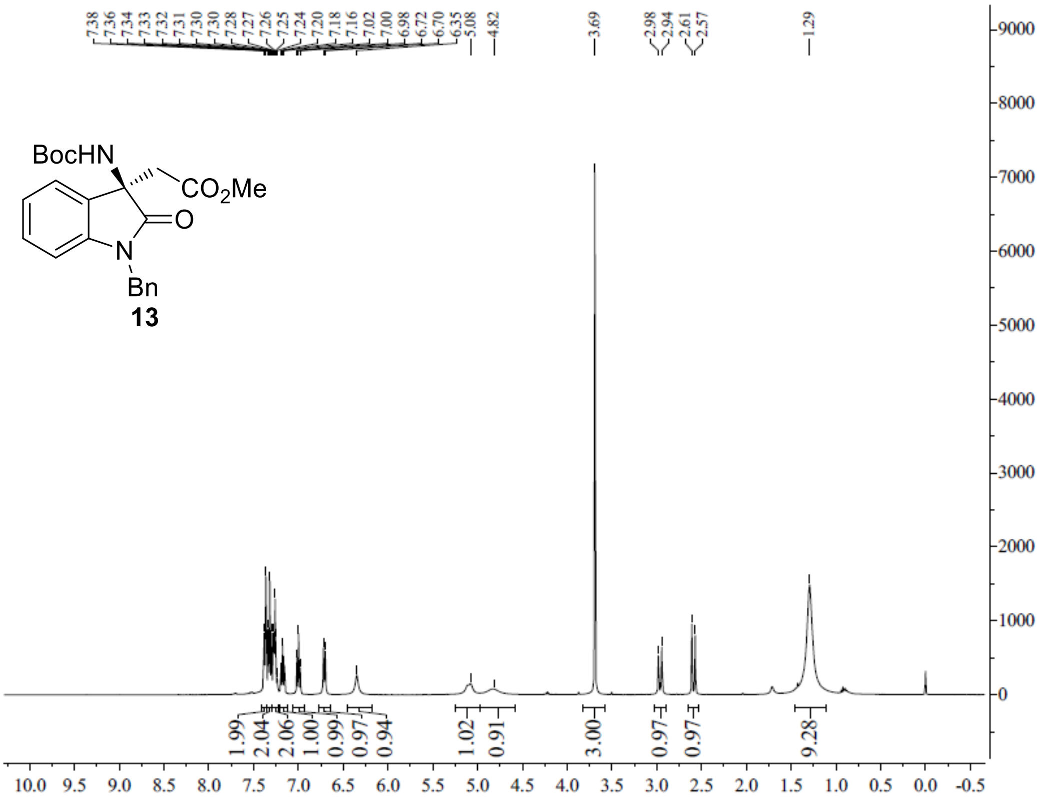

Figure S155. ^{1}H NMR spectrum of **13**, related to **Scheme 3**.

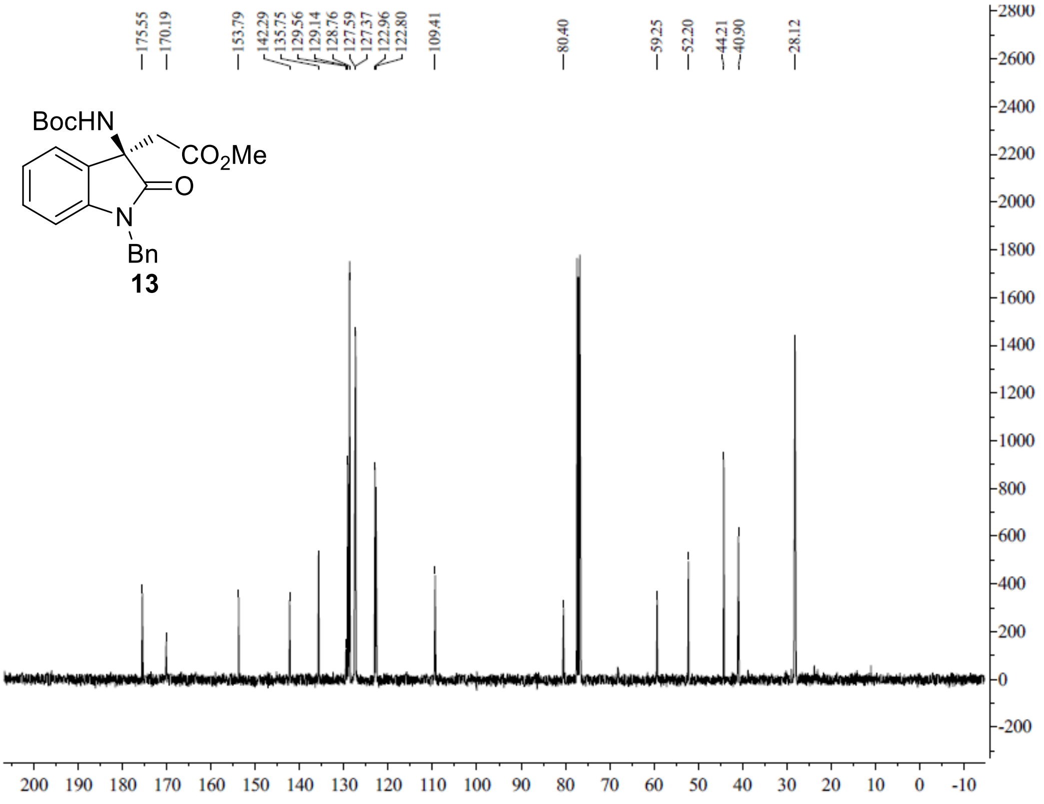

Figure S156. ^{13}C NMR spectrum of **13**, related to **Scheme 3.**

<Chromatogram>

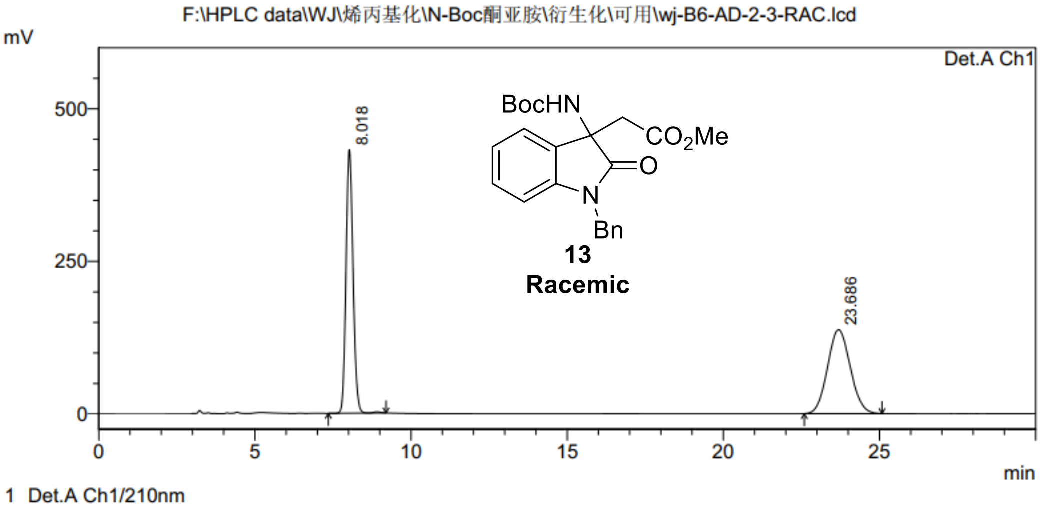

PeakTable

Detector A Ch1 210nm

Peak#	Ret. Time	Area	Height	Area %	Height %
1	8.018	6766262	431906	49.262	75.829
2	23.686	6968944	137671	50.738	24.171
Total		13735206	569577	100.000	100.000

<Chromatogram>

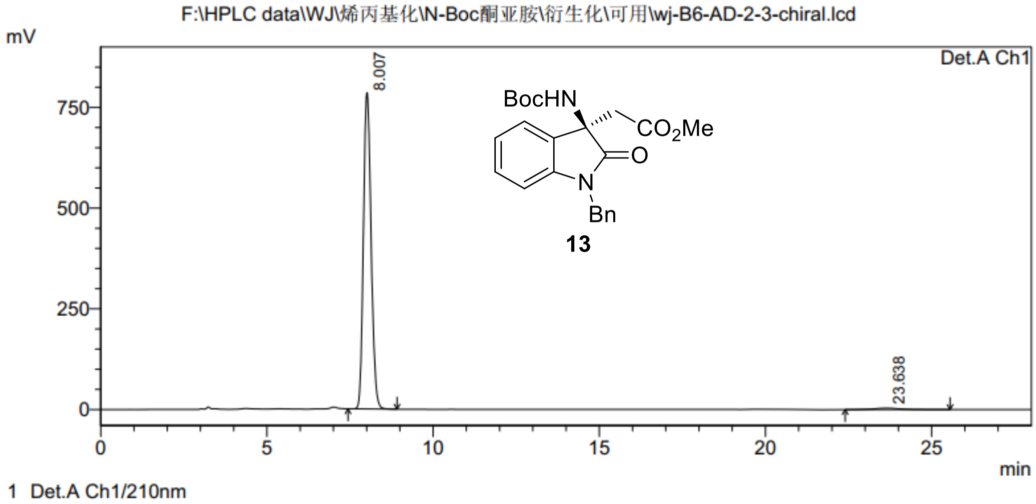

PeakTable

Detector A Ch1 210nm

Peak#	Ret. Time	Area	Height	Area %	Height %
1	8.007	12577355	785490	98.651	99.567
2	23.638	171978	3412	1.349	0.433
Total		12749333	788902	100.000	100.000

Figure S157. HPLC spectrum of **13**, related to **Scheme 3.**

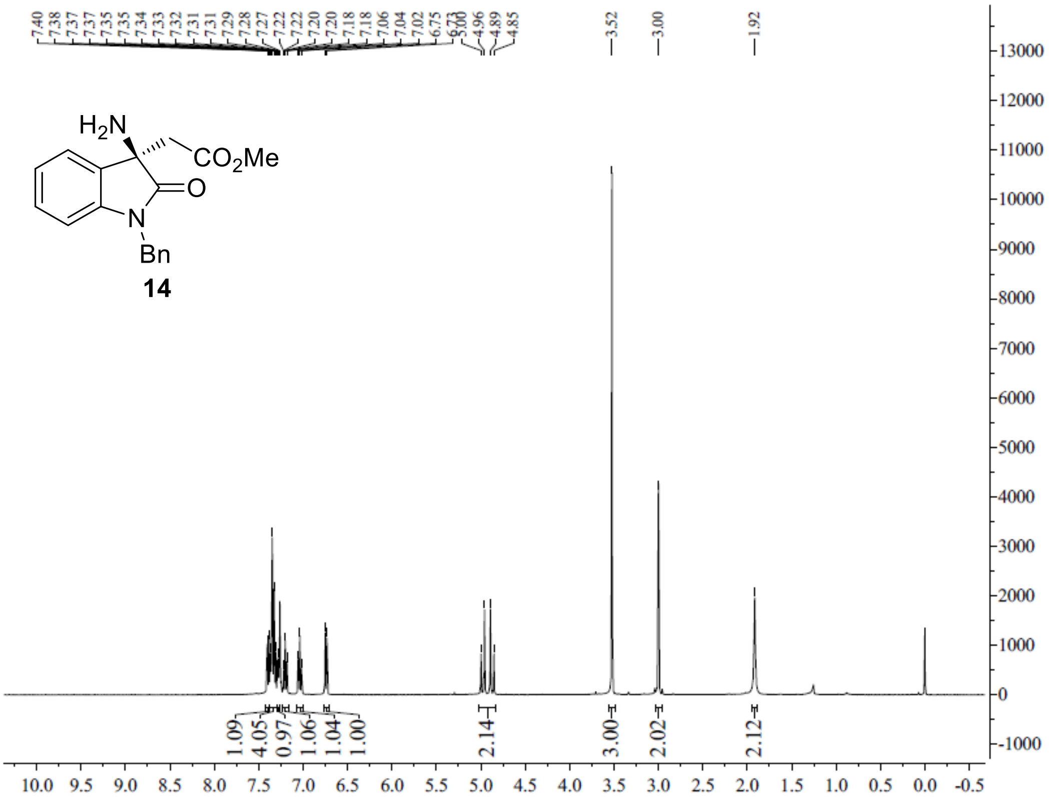

Figure S158. ^{1}H NMR spectrum of **14**, related to **Scheme 3**.

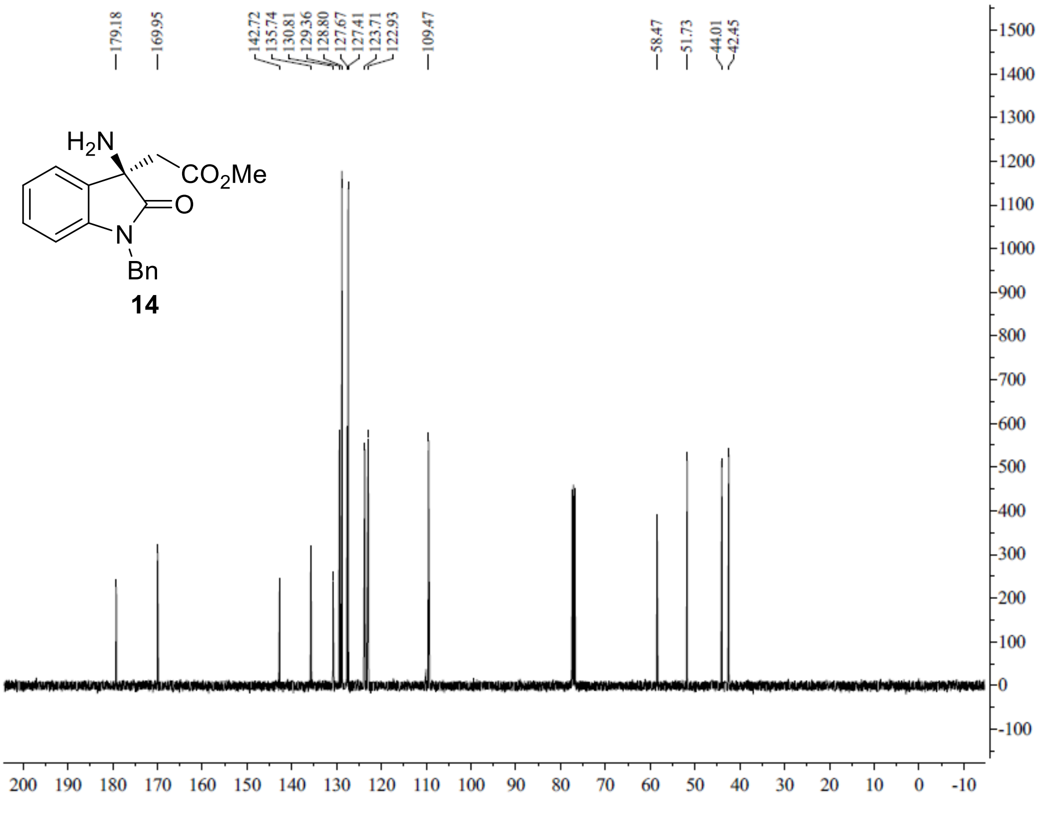

Figure S159. ^{13}C NMR spectrum of **14**, related to **Scheme 3.**

<Chromatogram>

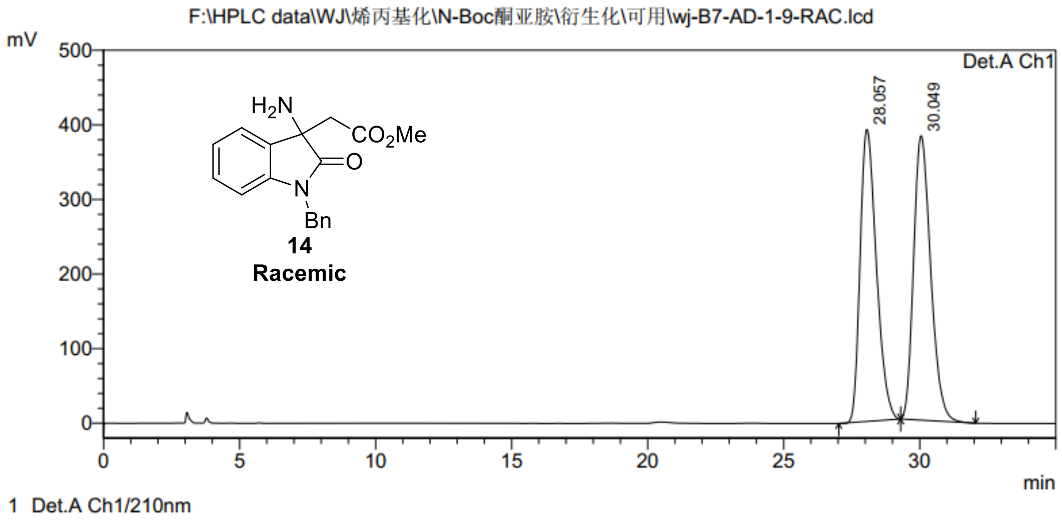

PeakTable

Detector A Ch1 210nm

Peak#	Ret. Time	Area	Height	Area %	Height %
1	28.057	16227027	391688	49.391	50.674
2	30.049	16627452	381271	50.609	49.326
Total		32854478	772959	100.000	100.000

<Chromatogram>

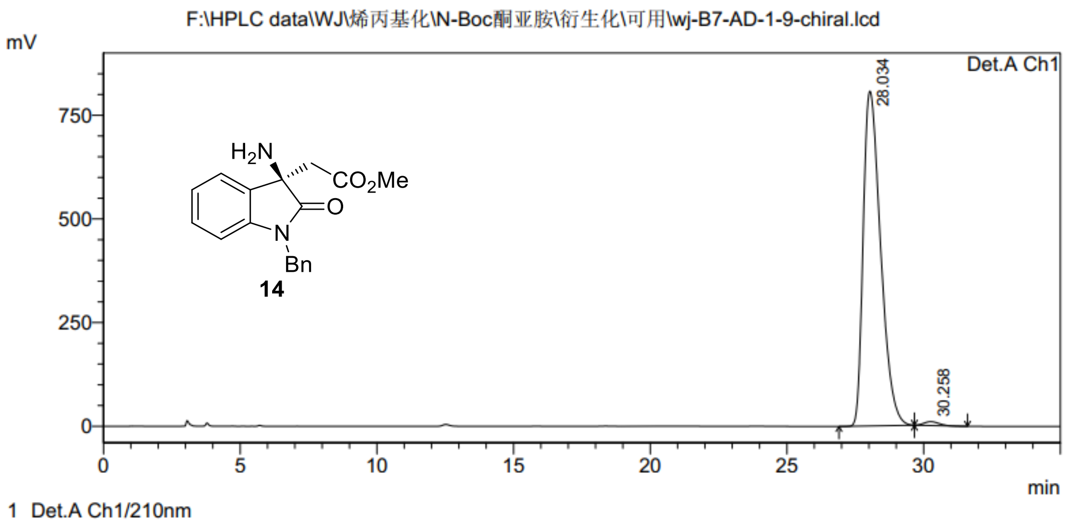

PeakTable

Detector A Ch1 210nm

Peak#	Ret. Time	Area	Height	Area %	Height %
1	28.034	35726218	807225	99.013	98.855
2	30.258	356276	9352	0.987	1.145
Total		36082494	816578	100.000	100.000

Figure S160. HPLC spectrum of **14**, related to **Scheme 3.**

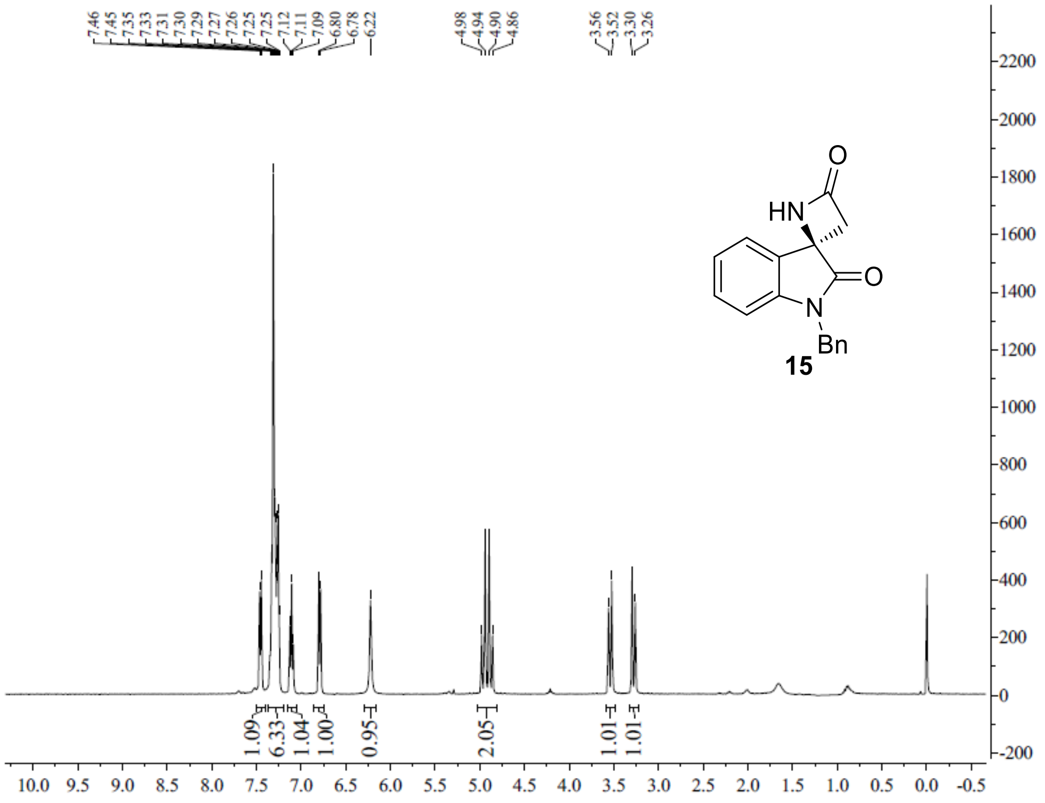

Figure S161. ^{1}H NMR spectrum of **15**, related to **Scheme 3**.

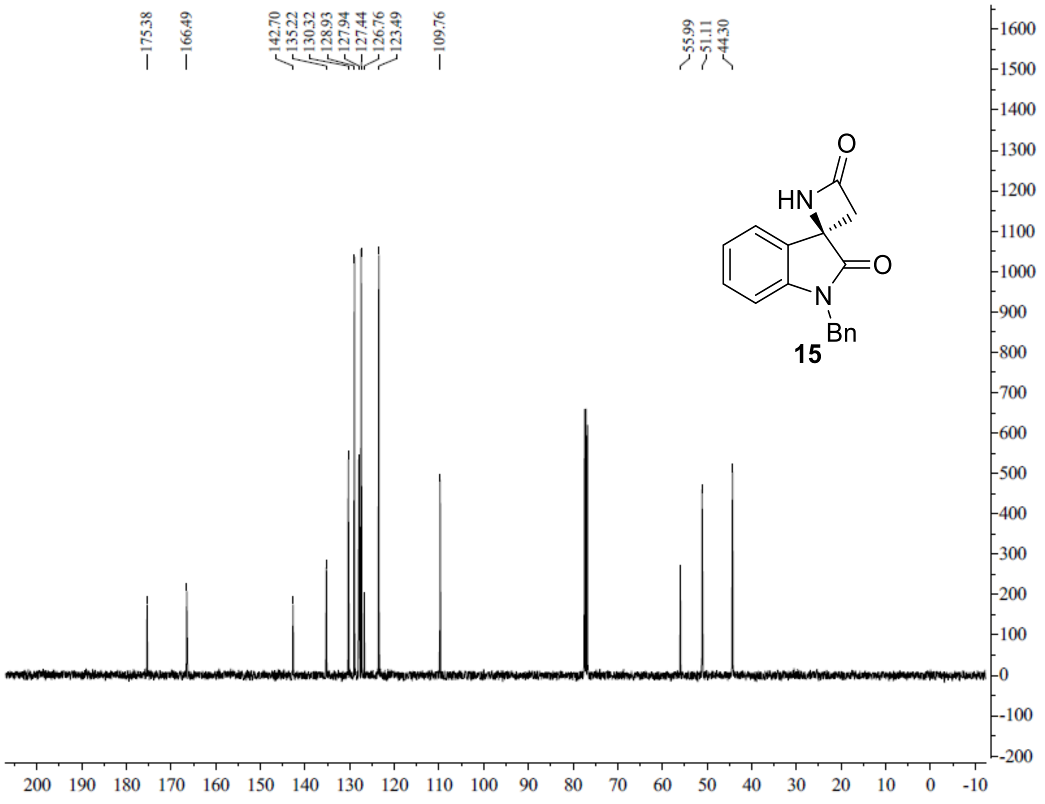

Figure S162. ^{13}C NMR spectrum of **15**, related to **Scheme 3.**

<Chromatogram>

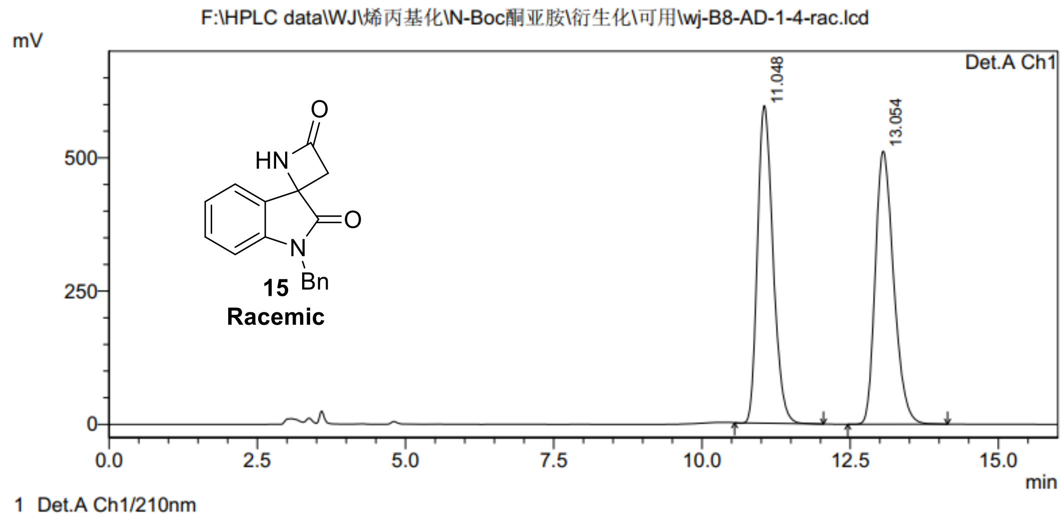

PeakTable

Detector A Ch1 210nm

Peak#	Ret. Time	Area	Height	Area %	Height %
1	11.048	10836714	595851	49.419	53.766
2	13.054	11091413	512384	50.581	46.234
Total		21928127	1108235	100.000	100.000

<Chromatogram>

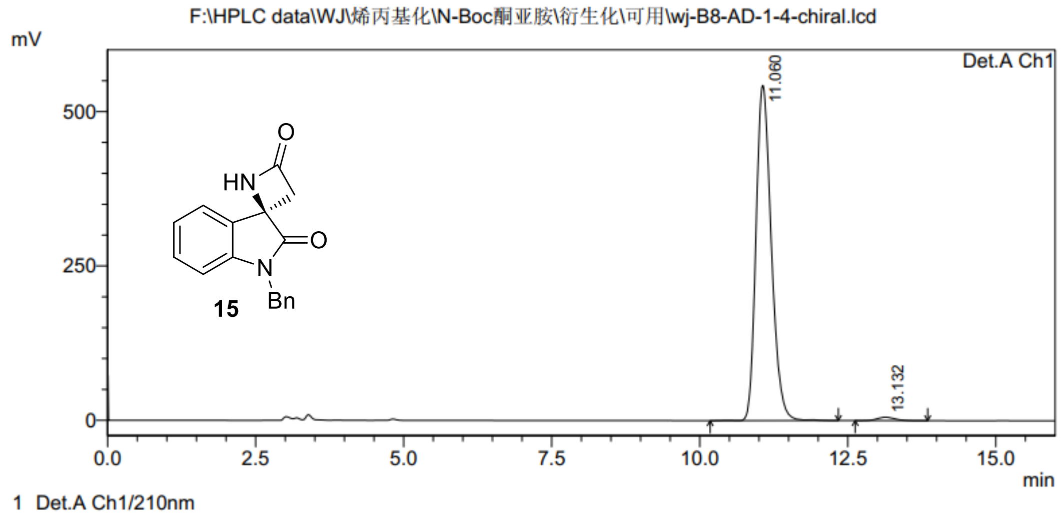

PeakTable

Detector A Ch1 210nm

Peak#	Ret. Time	Area	Height	Area %	Height %
1	11.060	9755785	543158	98.831	98.978
2	13.132	115407	5607	1.169	1.022
Total		9871193	548765	100.000	100.000

Figure S163. HPLC spectrum of **15**, related to **Scheme 3.**

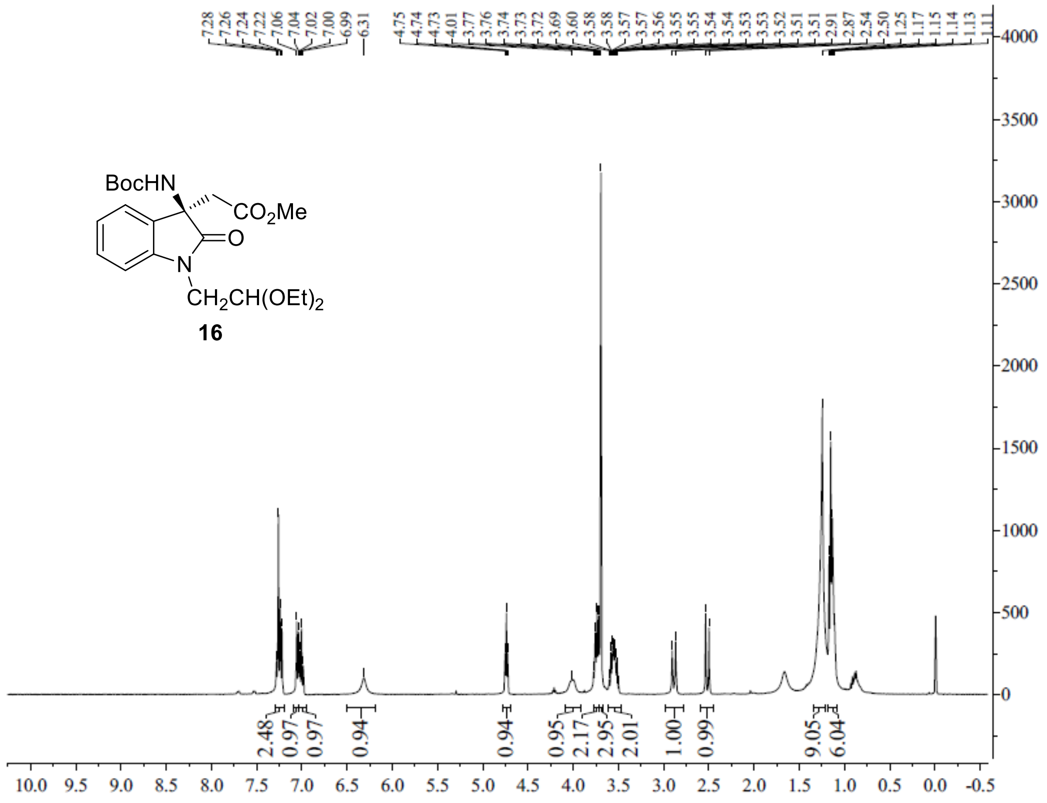

Figure S164. ^{1}H NMR spectrum of **16**, related to **Scheme 3**.

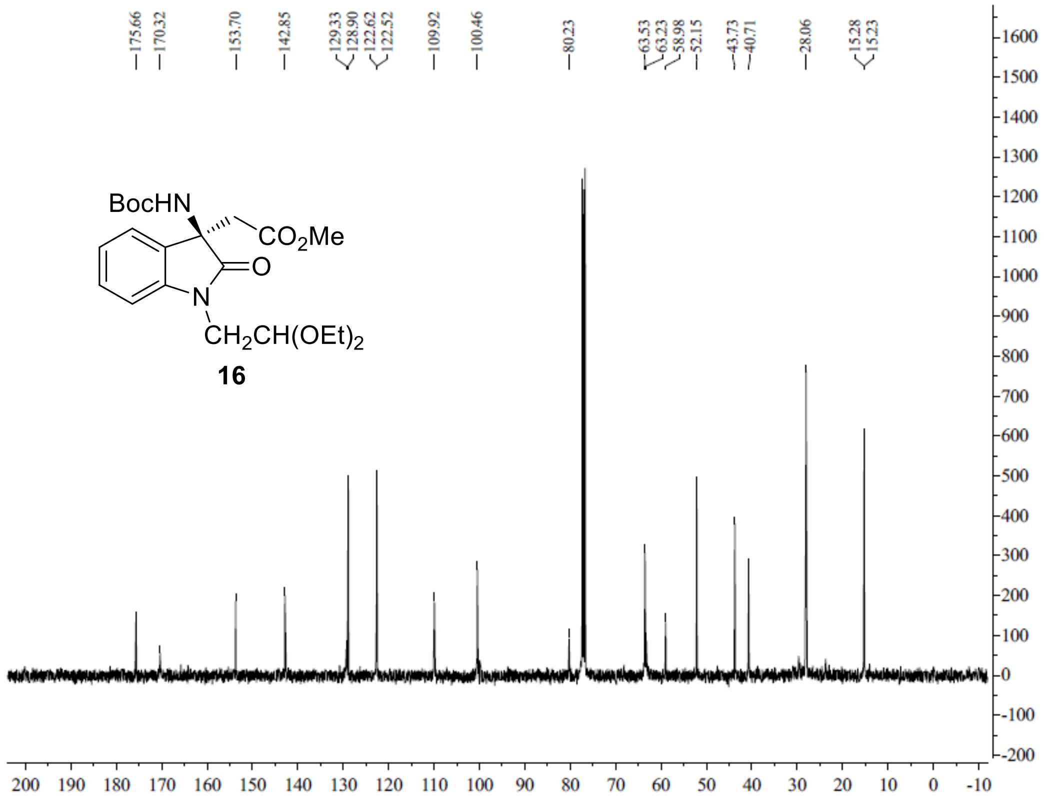

Figure S165. ^{13}C NMR spectrum of **16**, related to **Scheme 3.**

<Chromatogram>

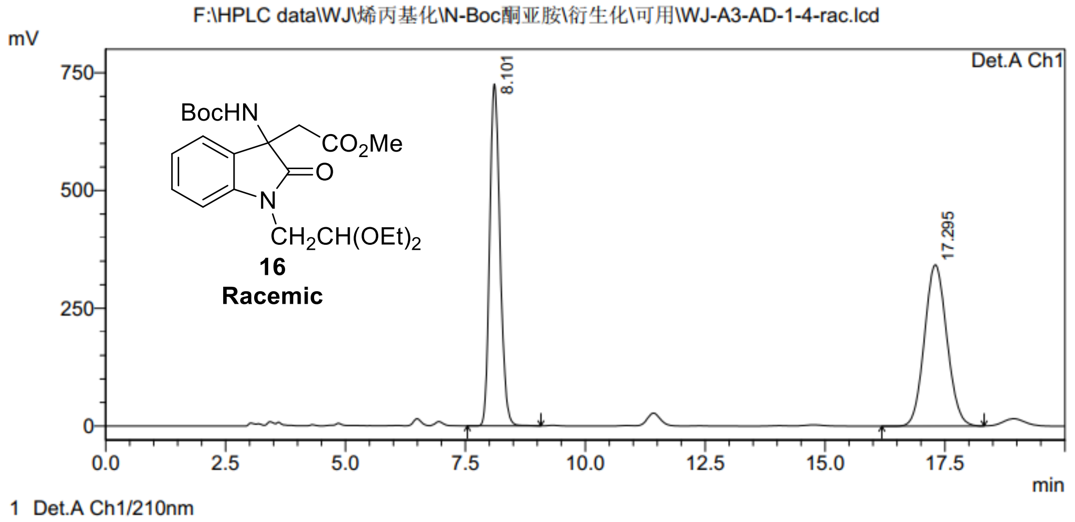

PeakTable

Detector A Ch1 210nm

Peak#	Ret. Time	Area	Height	Area %	Height %
1	8.101	10841943	726025	49.346	67.952
2	17.295	11129428	342416	50.654	32.048
Total		21971371	1068441	100.000	100.000

<Chromatogram>

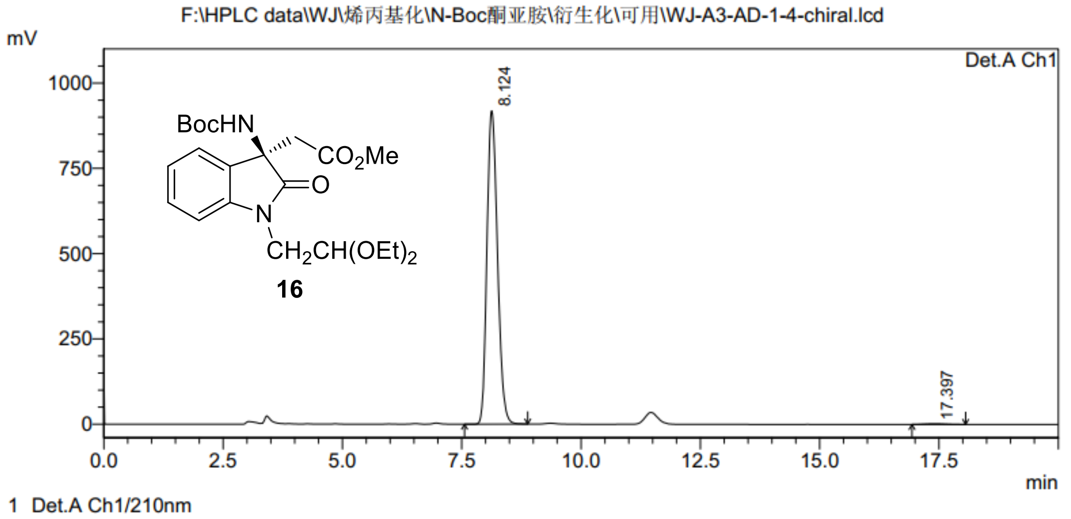

PeakTable

Detector A Ch1 210nm

Peak#	Ret. Time	Area	Height	Area %	Height %
1	8.124	14084779	918293	99.582	99.787
2	17.397	59081	1962	0.418	0.213
Total		14143861	920255	100.000	100.000

Figure S166. HPLC spectrum of **16**, related to **Scheme 3.**

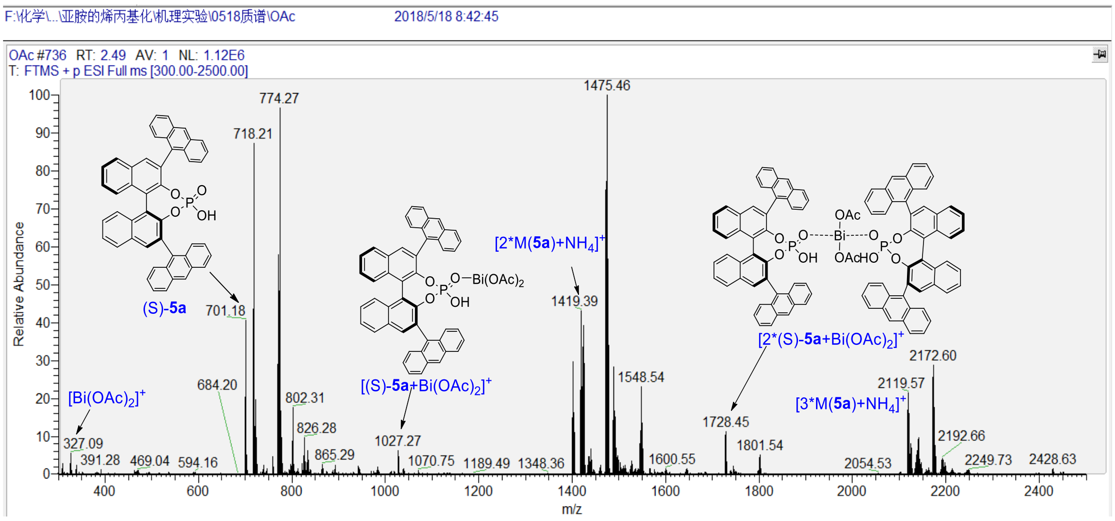

Data S1. ESI-MS experiment. To a sample bottle was added (*S*)-**5a** (0.01 mmol), $Bi(OAc)_3$ (0.01 mmol), and CH_3CN (0.5 mL). After 30 min stirring at rt, the supernate was diluted with CH_3CN and subjected to analysis by ESI-MS, related to **Figure 3 and Figure 4.**

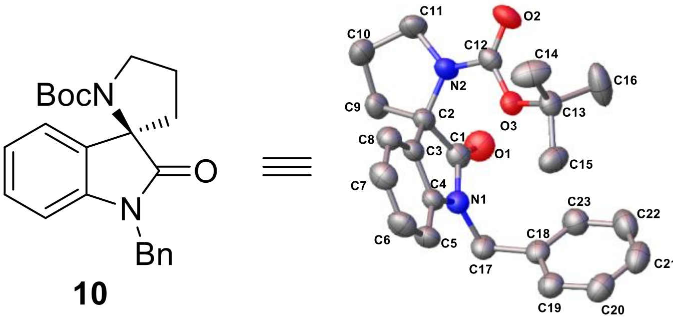

Data S2. Single X-ray structure of **10**, related to **Scheme 3.**

Table S1: Crystal data and structure refinement, related to **Scheme 3.**

Identification code	
Empirical formula	$C_{23}H_{26}N_2O_3$
Formula weight	378.46
Temperature / K	294
Crystal system	Orthorhombic
Space group	$P2_12_12_1$
a / Å, b / Å, c / Å	9.50943(4), 13.71174(5), 15.80478(8)
α/°, β/°, γ/°	90, 90, 90
Volume / $Å^3$	2060.799(15)
Z	4
ρ_{calc} / mg mm^{-3}	1.220
μ / mm^{-1}	0.649
F(000)	808
Crystal size / mm^3	0.34 × 0.3 × 0.22
Theta range for data collection	4.269 to 79.328°
Index ranges	$-12 \leq h \leq 12$, $-17 \leq k \leq 17$, $-15 \leq l \leq 18$
Reflections collected	24804
Independent reflections	4340[R(int) = 0.0242]
Data/restraints/parameters	4340/10/273
Goodness-of-fit on F^2	1.079
Final R indexes [I>2σ (I)]	$R_1 = 0.0268$, $wR_2 = 0.0734$
Final R indexes [all data]	$R_1 = 0.0270$, $wR_2 = 0.0736$
Largest diff. peak/hole / e $Å^{-3}$	0.128/-0.123

Table S2. Detailed reaction optimization,[a] related to **Table 1.**

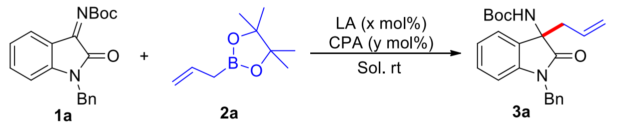

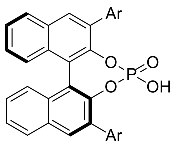

(*S*)-**5a**: Ar = 9-anthyl
(*S*)-**5b**: Ar = 3,5-$(CF_3)_2C_6H_3$
(*S*)-**5c**: Ar = 1-naphthyl
(*S*)-**5d**: Ar =9- phenanthrenyl
(*S*)-**5e**: Ar = 2,4,6-$^iPr_3C_6H_2$
(*S*)-**5f**: Ar = 2,4,6-$Me_3C_6H_2$
(*S*)-**5g**: Ar = 3,5-$Ph_2C_6H_3$
(*S*)-**5h**: Ar = triphenylsilyl
(*S*)-**5i**: Ar = 2-naphthyl
(*S*)-**5j**: Ar = 1-pyrenyl
(*S*)-**5k**: Ar = 3,5-tBu_2-4-$OMeC_6H_2$
(*S*)-**5l**: Ar = 4-(2-naphthyl)C_6H_4
(*S*)-**5m**: Ar = 4-Ph-C_6H_4

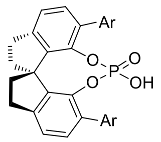

(*S*)-**6a**: Ar = 9-anthyl

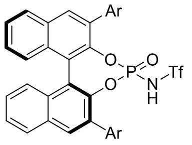

(*S*)-**6b**: Ar = 9-anthyl

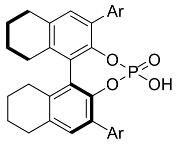

(*S*)-**6c**: Ar = 9-anthyl

Entry	LA	CPA	Solvent	x/%	y/%	Time/min	Yield[b]/%	*er*[c]/%
1	$Bi(OAc)_3$	**5a**	$CHCl_3$	2	3	20	99	87.9:12.1
2	$Bi(OAc)_3$	**5b**	$CHCl_3$	2	3	25	88	61.7:38.3
3	$Bi(OAc)_3$	**5c**	$CHCl_3$	2	3	40	98	75.3:24.7
4	$Bi(OAc)_3$	**5d**	$CHCl_3$	2	3	25	95	55.1:44.9
5	$Bi(OAc)_3$	**5e**	$CHCl_3$	2	3	25	92	50.4:49.6
6	$Bi(OAc)_3$	**5f**	$CHCl_3$	2	3	30	90	81.1:18.9
7	$Bi(OAc)_3$	**5g**	$CHCl_3$	2	3	20	99	79.1:20.9
8	$Bi(OAc)_3$	**5h**	$CHCl_3$	2	3	300	97	69.9:30.1
9	$Bi(OAc)_3$	**5i**	$CHCl_3$	2	3	25	99	61.6:38.4
10	$Bi(OAc)_3$	**5j**	$CHCl_3$	2	3	25	99	53.3:46.7
11	$Bi(OAc)_3$	**5k**	$CHCl_3$	2	3	30	85	36.2:63.8
12	$Bi(OAc)_3$	**5l**	$CHCl_3$	2	3	90	96	65.4:34.6
13	$Bi(OAc)_3$	**5m**	$CHCl_3$	2	3	150	92	70.5:29.5
14	$Bi(OAc)_3$	**6a**	$CHCl_3$	2	3	80	99	15.5:84.5
15	$Bi(OAc)_3$	**6b**	$CHCl_3$	2	3	30	97	89.0:11.0
16	$Bi(OAc)_3$	**6c**	$CHCl_3$	2	3	30	99	85.5:14.5
17	$Bi(OAc)_3$	**5a**	$CHCl_3$	2	3	20	99	87.9:12.1
18	$Bi(OAc)_3$	**5a**	DCE	2	3	25	93	84.2:15.8
19	$Bi(OAc)_3$	**5a**	TBME	2	3	35	94	98.6:1.4
20	$Bi(OAc)_3$	**5a**	PhOMe	2	3	15	99	94.0:6.0
21	$Bi(OAc)_3$	**5a**	Et_2O	2	3	20	99	99.1:0.9
22[d]	$Bi(OAc)_3$	**5a**	Et_2O	2	3	6h	84	99.4:0.6
23	$Bi(OAc)_3$	**5a**	Et_2O	2	3	20	99	98.4:1.6
24	$Bi(OAc)_3$	**5a**	Et_2O	2	2	25	95	98.6:1.4
25	$Bi(OAc)_3$	5a	Et_2O	1	2	35	96	98.9:1.1
26	$Bi(OAc)_3$	**5a**	Et_2O	1	1	45	87	98.4:1.6
27	$Bi(OAc)_3$	**5a**	Et_2O	0.5	1	7h	84	98.4:1.6
28	$Y(OTf)_3$	**5a**	Et_2O	1	2	56h	47	68.0:32.0
29	$Sc(OTf)_3$	**5a**	Et_2O	1	2	56h	42	60.8:39.2
30	$Yb(OTf)_3$	**5a**	Et_2O	1	2	54h	26	64.2:35.8
31	AgOTf	**5a**	Et_2O	1	2	58h	37	62.0:38.0
32	$La(OTf)_3$	**5a**	Et_2O	1	2	54h	80	70.2:29.8
33	$InBr_3$	**5a**	Et_2O	1	2	52h	51	50.4:49.6
34	$Y(OTf)_3$	**5a**	Et_2O	1	2	56h	47	68.0:32.0
35	$Yb(OAc)_3$	**5a**	Et_2O	1	2	25h	<5	--
36	$Fe(OAc)_2$	**5a**	Et_2O	1	2	25h	<5	--

[a] The reactions (entries 1-28) were carried out with **1a** (0.1 mmol), **2a** (0.12 mmol) in 0.5 mL solvent, while other entries were carried out with **1a** (0.2 mmol), **2a** (0.24 mmol) in 1.0 mL solvent. [b] Yield of isolated product. [c] Determined by HPLC analysis. [d] The reaction was performed at 0 °C.

Table S3. Detailed nonlinear effect experiment,[a] related to **Figure 3.**.

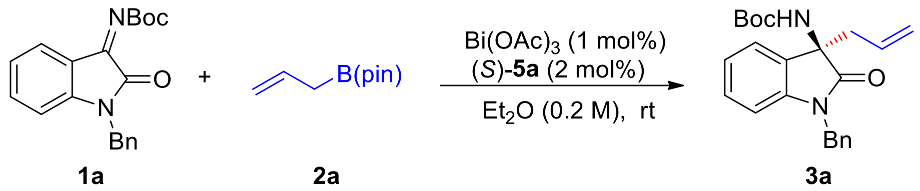

Entry	er-**5a**	er-1	er-2
A	50:50	49.0:51.0	51.9:48.1
B	60:40	66.7:33.3	68.9:31.1
C	70:30	82.2:17.8	82.2:17.8
D	80:20	89.0:11.0	91.9:8.1
E	90:10	96.0:4.0	96.8:3.2
F	99.5:0.5	98.7:1.3	98.8:1.2

[a]The reactions s were carried out with **1a** (0.2 mmol), **2a** (0.24 mmol) in 1.0 mL Et_2O at room temperature., and the er determined by HPLC analysis.

Computational Details

All density functional theory (DFT) calculations were performed with Gaussian 09 (Frisch et al., 2009). The system size, particularly for the catalyst, and conformational degrees of freedom make full QM geometry optimization unreasonable. So geometry optimization of all the minima and transition states involved was carried out at the hybrid ONIOM(QM:MM) (Morokuma et al., 1996; Morokuma and Vreven, 2000; Morokuma et al., 2015) methods which have provided reasonable agreement with experimental results for the study of similar systems (Simón and Goodman, 2008; Simón and Goodman, 2010; Simón and Goodman, 2011; Simón and Goodman, 2012; Simón and Paton, 2015; Simón and Paton, 2016; Simón and Paton, 2017; Simón and Paton, 2018). Atoms that participate in bond forming/breaking events or in establishing H-bond interactions were included in the highlevel layer and were treated by a QM method and the rest of the atoms of the catalysts were included in the low-level layer and were studied by a MM method. Atoms in different ONIOM layers are illustrated in 3D structures with the high level (HL) layer as "ball & stick" type and the low level (LL) layer as "wireframe" type. Additionally, atoms in the low level layer are hided in schemes. Figures were prepared with Pymol software. During optimization and TSs searches, the M06-2X (Zhao and Truhlar, et al., 2008) hybrid meta-GGA functional with the 6-31G(d) basis set for C, H, O, N and P atoms and LANL2DZ (Hay and Wadt, 1985) effective core potential (ECP) and split-valence basis set for Bi atoms was used for the QM layer. The low level layer was treated with a UFF (Rappe et al., 1992) force field. Single point energies used the M06-2X (Zhao and Truhlar, et al., 2008) hybrid meta-GGA functional with 6-311+G(d,p) basis set for C, H, O, N and P and the SDD (Andrae et al., 1990) ECP for Bi atoms in conjunction with the SMD implicit solvation model to account for the solvation effects of diethylether.

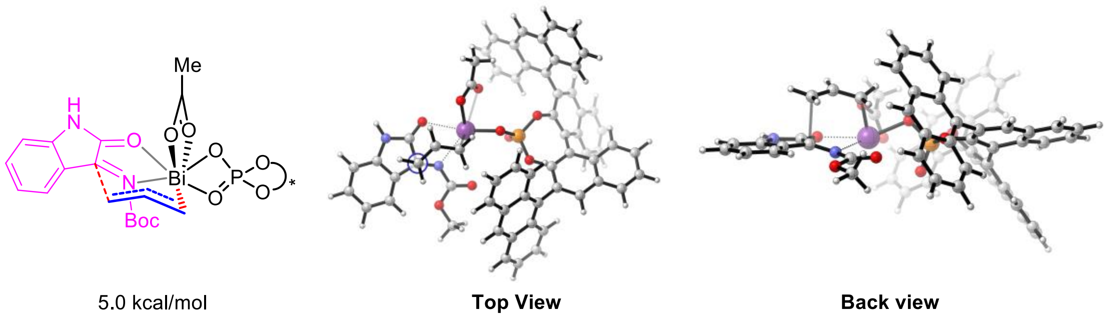

Figure S167. Optimized eclipsed conformation of **TS-1P-(*R*)**, related to **Figure 4.**

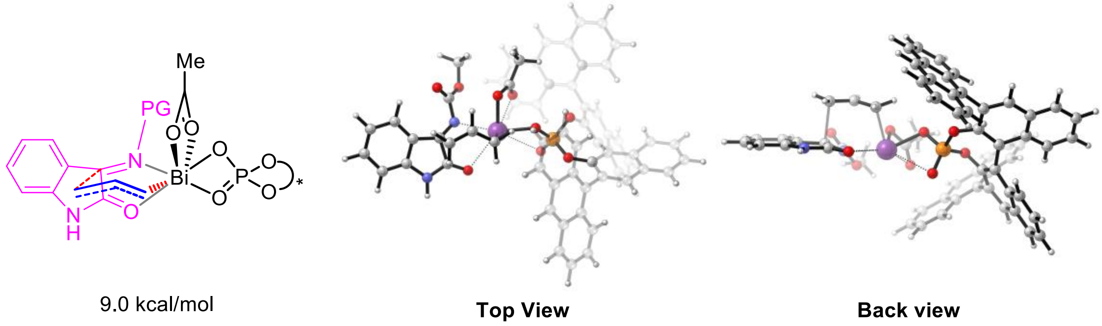

Figure S168. Optimized eclipsed conformation of **TS-1P-(*S*)**, related to **Figure 4.**

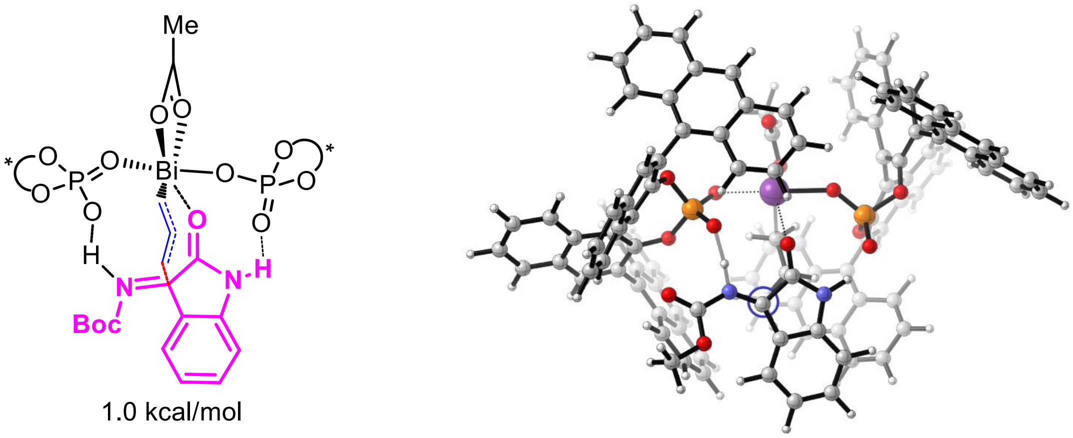

Figure S169. Optimized eclipsed conformation of **TS-2P-(*R*)**, related to **Figure 4.**

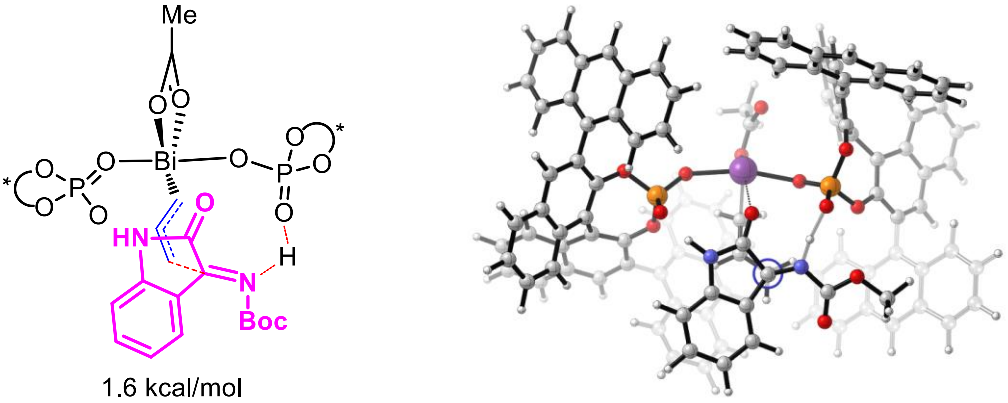

Figure S170. Optimized eclipsed conformation of **TS-2P-(*S*)**, related to **Figure 4.**

Transparent Methods

General information

Commercial reagents were used as received, unless otherwise indicated. ^{1}H and ^{13}C NMR were recorded on a Bruker - DPX 400 spectrometer. ^{19}F NMR were recorded on a Varian NMR 400 spectrometer. Tetramethylsilane (TMS) served as the internal standard for ^{1}H NMR, and $CDCl_3$ served as the internal standard for ^{13}C NMR. The following abbreviations were used to designate the multiplicities: s = singlet; d = doublet; t = triplet; q = quartet; m = multiplet; br = broad. All first-order splitting patterns were assigned on the basis of the appearance of the multiplet. Splitting patterns that could not be easily interpreted are designated as multiplet (m) or broad (br). HPLC analysis was performed using Chiralcel columns purchased. Mass spectra were obtained using electrospray ionization (ESI) mass spectrometer. ESI-MS studies on catalytic complex were conducted on Thermo LTQ XL. Isatin-derived ketimines **1**(Bittner et al., 1985; Wang et al., 2012; Shi et al., 2013; Mao et al., 2014; Zhou and Yua, 2015; Nakamura and Takahashi, 2015; Babu et al., 2015) and allyboronic acid pinacol ester **2d, 2e** (Maulide et al., 2013) were prepared according to the reported literature procedure.

General procedure for Asymmetric Allylation of Isatin-Derived Ketimines with Allylboronates.

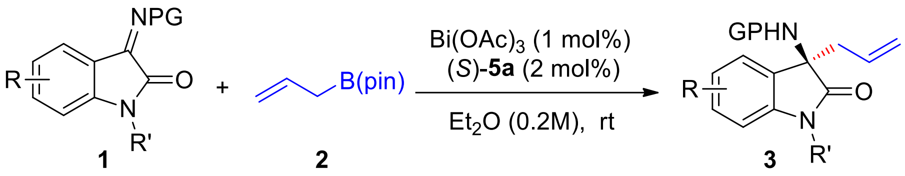

To a stirred solution of isatin-derived ketimines **1** (0.2 mmol), $Bi(OAc)_3$ (1 mol%) and chiral phosphoric acid catalyst **5a** (2 mol%) in Et_2O (1.0 mL) was added allyl pinacol boronic ester **2** (0.24 mmol). The reaction was stirred at room temperature until completed. Then, the crude mixture was direct purified by flash chromatography (petroleum ether/EtOAc = 5/1) to afford the product **3**.

Characterization data of ketimines 1h, 1i, and products 3.

***tert*-butyl (1-benzyl-6-methoxy-2-oxoindolin-3-ylidene)carbamate (1h)**

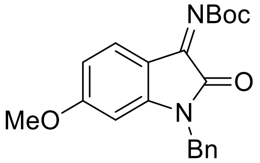

Yellow solid; ^{1}H NMR (400 MHz, $CDCl_3$) δ 7.59 (d, J = 8.5 Hz, 1H), 7.38 - 7.27 (m, 5H), 6.52 (dd, J = 8.6, 2.0 Hz, 1H), 6.24 (dd, J = 7.2, 2.1 Hz, 1H), 4.89 (s, 1H), 4.86 (s, 1H), 3.82 (s, 1H), 3.78 (s, 2H), 1.63 (s, 6H), 1.45 (s, 6H); ^{13}C NMR (101 MHz,

$CDCl_3$) δ 180.56, 168.13, 165.83, 159.68, 153.19, 149.43, 134.76, 129.03, 128.96, 128.12, 128.00, 127.37, 107.92, 107.55, 98.28, 98.06, 56.03, 55.78, 43.97, 28.23, 28.06; HRMS (ESI): *m/z* calcd for $C_{21}H_{23}N_2O_4$ $[M+H]^+$: 367.1658; found: 367.1655.

***tert*-butyl (1-benzyl-6-fluoro-2-oxoindolin-3-ylidene)carbamate (1i)**

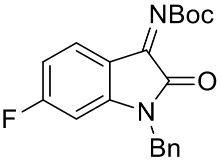

Yellow solid; ^1H NMR (400 MHz, $CDCl_3$) δ 7.70 - 7.60 (m, 1H), 7.40 - 7.26 (m, 5H), 6.74 (t, *J* = 8.9 Hz, 1H), 6.44 (d, *J* = 8.0 Hz, 1H), 4.87 (s, 2H), 1.64 (s, 9H); ^{13}C NMR (101 MHz, $CDCl_3$) δ 167.20 (d, *J* = 256.7 Hz), 160.28 , 157.47, 151.61, 149.44 (d, *J* = 11.7 Hz), 134.16, 129.10, 128.26, 127.41, 126.51 (d, *J* = 11.1 Hz), 115.39, 110.42 (d, *J* = 23.4 Hz), 99.36 (d, *J* = 27.9 Hz), 83.71, 44.16, 28.05; HRMS (ESI): *m/z* calcd for $C_{20}H_{20}FN_2O_3$ $[M+H]^+$: 355.1458; found: 355.1451.

(*R*)-*tert*-butyl (3-allyl-1-benzyl-2-oxoindolin-3-yl)carbamate (3a)

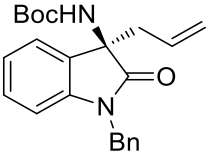

White solid, 72.6mg, 96% yield, 99.2:0.8 *er*; $[\alpha]_D^{27}$ = +12.0 (c = 0.3, $CHCl_3$); MP 63 - 64 °C; ^1H NMR (400 MHz, $CDCl_3$) δ 7.36 (d, *J* = 7.2 Hz, 2H), 7.31 (t, *J* = 7.4 Hz, 2H), 7.26 - 7.23 (m, 2H), 7.17 (t, *J* = 7.7 Hz, 1H), 7.02 (t, *J* = 7.5 Hz, 1H), 6.70 (d, *J* = 7.8 Hz, 1H), 5.71 (ddt, *J* = 17.4, 10.1, 7.4 Hz, 1H), 5.36 - 5.04 (m, 4H), 4.79 (br, 1H), 2.63 (dd, *J* = 13.4, 7.4 Hz, 1H), 2.50 (dd, *J* = 13.4, 7.4 Hz, 1H), 1.26 (s, 9H); ^{13}C NMR (101 MHz, $CDCl_3$) δ 176.75, 153.69, 142.31, 135.90, 130.07, 128.68, 128.61, 127.51, 127.40, 122.72, 122.52, 121.43, 109.16, 80.40, 60.83, 44.06, 42.31, 28.06; HRMS (ESI): *m/z* calcd for $C_{23}H_{27}N_2O_3$ $[M+H]^+$: 379.2016; found: 379.2017; HPLC: Daicel Chiralpak IC, *n*-hexane/*i*-PrOH = 4:1, Flow rate = 1.0 mL/min, λ = 210 nm, t_R = 9.8 min (minor) and t_R = 11.9 min (major).

(*R*)-*tert*-butyl (3-allyl-1-benzyl-5-methyl-2-oxoindolin-3-yl)carbamate (3b)

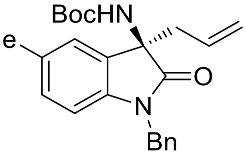

White solid, 76.9mg, 98% yield, 99.2:0.8 *er*; $[\alpha]_D^{27}$ = +32.6 (c = 1.0, $CHCl_3$); MP 95 - 96 °C; ^1H NMR (400 MHz, $CDCl_3$) δ 7.37 - 7.27 (m, 4H), 7.25 (t, *J* = 7.1 Hz, 1H), 7.07 (s, 1H), 6.96 (d, *J* = 7.8 Hz, 2H), 6.57 (d, *J* = 7.9 Hz, 2H), 5.82 - 5.64 (m, 1H), 5.30 - 5.15 (m, 4H), 4.78 (s, 1H), 2.61 (dd, *J* = 13.4, 7.4 Hz, 1H), 2.48 (dd, *J* = 13.5,

7.4 Hz, 1H), 2.30 (s, 3H), 1.27 (s, 9H); ^{13}C NMR (101 MHz, $CDCl_3$) δ 176.68, 153.76, 139.89, 136.00, 132.02, 130.24, 128.88, 128.65, 127.45, 127.37, 123.53, 121.29, 108.92, 80.33, 60.90, 44.06, 42.38, 28.10, 21.15; HRMS (ESI): *m/z* calcd for $C_{24}H_{29}N_2O_3$ $[M+H]^+$: 393.2173; found: 393.2170; HPLC: Daicel Chiralpak IC, *n*-hexane/*i*-PrOH = 4:1, Flow rate = 1.0 mL/min, λ = 210 nm, t_R = 11.6 min (minor) and t_R = 15.2 min (major).

(*R*)-*tert*-butyl (3-allyl-1-benzyl-5-methoxy-2-oxoindolin-3-yl)carbamate (3c)

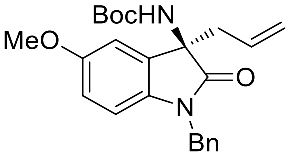

White solid, 77.5mg, 95% yield, 99.3:0.7 *er*; $[\alpha]_D^{27}$ = +34.2 (c = 1.0, $CHCl_3$); MP 89 - 90 °C; ^{1}H NMR (400 MHz, $CDCl_3$) δ 7.26 - 7.13 (m, 5H), 6.78 (d, *J* = 2.5 Hz, 1H), 6.58 (dd, *J* = 8.6, 2.5 Hz, 1H), 6.48 (d, *J* = 8.5 Hz, 1H), 5.62 (ddt, *J* = 17.3, 10.1, 7.4 Hz, 1H), 5.23 - 5.06 (m, 3H), 4.99 (s, 1H), 4.68 (s, 1H), 3.65 (s, 3H), 2.52 (dd, *J* = 13.5, 7.3 Hz, 1H), 2.39 (dd, *J* = 13.4, 7.5 Hz, 1H), 1.19 (s, 9H); ^{13}C NMR (101 MHz, $CDCl_3$) δ 176.44, 155.95, 153.74, 135.96, 135.75, 131.95, 130.05, 128.66, 127.47, 127.39, 121.38, 112.69, 110.37, 109.52, 80.40, 61.20, 55.76, 44.14, 42.34, 28.11; HRMS (ESI): *m/z* calcd for $C_{24}H_{29}N_2O_4$ $[M+H]^+$: 409.2122; found: 409.2124; HPLC: Daicel Chiralpak IC, *n*-hexane/*i*-PrOH = 4:1, Flow rate = 1.0 mL/min, λ = 210 nm, t_R = 13.3 min (minor) and t_R = 15.3 min (major).

(*R*)-*tert*-butyl (3-allyl-1-benzyl-5-fluoro-2-oxoindolin-3-yl)carbamate (3d)

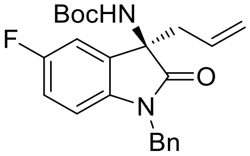

White solid, 76.9mg, 97% yield, 96.4:3.6 *er*; $[\alpha]_D^{27}$ = +6.7 (c = 0.3, $CHCl_3$); MP 68 - 69 °C; ^{1}H NMR (400 MHz, $CDCl_3$) δ 7.35 - 7.24 (m, 5H), 7.01 (dd, *J* = 7.8, 2.6 Hz, 1H), 6.86 (td, *J* = 8.9, 2.6 Hz, 1H), 6.59 (dd, *J* = 8.6, 4.1 Hz, 1H), 5.69 (ddt, *J* = 17.3, 10.1, 7.4 Hz, 1H), 5.35 - 5.10 (m, 3H), 5.05 (s, 1H), 4.84 (s, 1H), 2.63 (dd, *J* = 13.5, 7.4 Hz, 1H), 2.49 (dd, *J* = 13.4, 7.5 Hz, 1H), 1.32 (s, 9H); ^{13}C NMR (101 MHz, $CDCl_3$) δ 176.56, 159.24 (d, *J* = 241.5 Hz), 153.70, 138.21, 135.58, 132.25, 129.60, 128.74, 127.63, 127.36, 121.72, 114.78 (d, *J* = 23.3 Hz), 110.92 (d, *J* = 24.8 Hz), 109.74, 80.63, 61.15, 44.21, 42.11, 28.10; ^{19}F NMR (376 MHz, $CDCl_3$) δ -125.73; HRMS (ESI): *m/z* calcd for $C_{23}H_{26}FN_2O_3$ $[M+H]^+$: 397.1922; found: 397.1919; HPLC: Daicel Chiralpak IC, *n*-hexane/*i*-PrOH = 4:1, Flow rate = 1.0 mL/min, λ = 210 nm, t_R = 7.3 min (minor) and t_R = 8.3 min (major).

(*R*)-*tert*-butyl (3-allyl-1-benzyl-5-chloro-2-oxoindolin-3-yl)carbamate (3e)

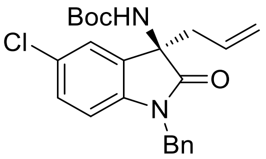

White solid, 81.6mg, 99% yield, 96.6:3.4 *er*; $[\alpha]_D^{27}$ = +36.8 (c = 1.0, $CHCl_3$); MP 105 - 106 °C; ^1H NMR (400 MHz, $CDCl_3$) δ 7.36 - 7.29 (m, 4H), 7.29 - 7.27 (m, 1H), 7.23 (d, *J* = 2.1 Hz, 1H), 7.13 (dd, *J* = 8.3, 2.1 Hz, 1H), 6.60 (d, *J* = 8.3 Hz, 1H), 5.69 (ddt, *J* = 17.3, 10.1, 7.4 Hz, 1H), 5.30 - 5.15 (m, 3H), 5.07 (s, 1H), 4.83 (s, 1H), 2.61 (dd, *J* = 13.4, 7.3 Hz, 1H), 2.48 (dd, *J* = 13.4, 7.5 Hz, 1H), 1.31 (s, 9H); ^{13}C NMR (101 MHz, CDCl3) δ 176.35, 153.67, 140.89, 135.43, 132.27, 129.54, 128.77, 128.54, 128.00, 127.68, 127.35, 123.18, 121.83, 110.20, 80.70, 60.94, 44.18, 42.08, 28.13; HRMS (ESI): *m/z* calcd for $C_{23}H_{25}ClN_2NaO_3$ $[M+Na]^+$: 435.1451, 437.1422; found: 435.1450, 437.1401; HPLC: Daicel Chiralpak IC, *n*-hexane/*i*-PrOH = 4:1, Flow rate = 1.0 mL/min, λ = 210 nm, t_R = 6.8 min (minor) and t_R = 7.6 min (major).

(*R*)-*tert*-butyl (3-allyl-1-benzyl-5-bromo-2-oxoindolin-3-yl)carbamate (3f)

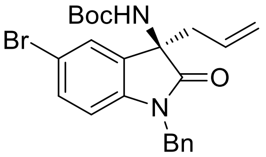

White solid, 90.3mg, 99% yield, 97.4:2.6 *er*; $[\alpha]_D^{27}$ = +45.0 (c = 1.0, $CHCl_3$); MP 115 - 116 °C; ^1H NMR (400 MHz, $CDCl_3$) δ 7.38 - 7.36 (t, *J* = 2.4 Hz, 1H), 7.30 - 7.35 (m, 4H), 7.30 - 7.23 (m, 2H), 6.56 (d, *J* = 8.3 Hz, 1H), 5.76 - 5.61 (m, 1H), 5.31 - 5.19 (m, 3H), 5.04 (s, 1H), 4.86 (s, 1H), 2.55 (ddd, *J* = 50.6, 13.5, 7.4 Hz, 2H), 1.32 (s, 9H); ^{13}C NMR (101 MHz, $CDCl_3$) δ 176.20, 153.62, 141.36, 135.37, 131.46, 129.52, 128.77, 127.68, 127.32, 125.90, 121.88, 115.33, 110.72, 80.74, 60.82, 44.15, 42.11, 28.12; HRMS (ESI): *m/z* calcd for $C_{23}H_{26}BrN_2O_3$ $[M+H]^+$: 457.1121, 459.1106; found: 457.1116, 459.1097; HPLC: Daicel Chiralpak IC, *n*-hexane/*i*-PrOH = 4:1, Flow rate = 1.0 mL/min, λ = 210 nm, t_R = 6.9 min (minor) and t_R = 7.7 min (major).

(*R*)-*tert*-butyl (3-allyl-1-benzyl-5-nitro-2-oxoindolin-3-yl)carbamate (3g)

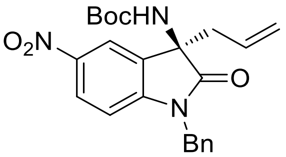

Yellow solid, 61.8mg, 73% yield, 93.8:6.2 *er*; $[\alpha]_D^{28}$ = +67.4 (c = 1.0, $CHCl_3$); MP 151 - 152 °C; ^1H NMR (400 MHz, $CDCl_3$) δ 8.14 (dd, J = 8.1, 2.0 Hz, 1H), 8.12 (s, 1H), 7.39 - 7.26 (m, 5H), 6.76 (d, J = 7.6 Hz, 1H), 5.66 (ddt, J = 17.3, 10.1, 7.4 Hz, 1H), 5.34 (s, 1H), 5.26 (d, J = 5.9 Hz, 1H), 5.22 (s, 1H), 5.03 (br, 1H), 4.99 (br, 1H), 2.65 (dd, J = 13.5, 7.3 Hz, 1H), 2.53 (dd, J = 13.5, 7.6 Hz, 1H), 1.35 (s, 9H); ^{13}C NMR (101 MHz, $CDCl_3$) δ 177.00, 153.68, 148.06, 143.44, 134.69, 131.45, 128.93, 128.82, 127.99, 127.34, 125.90, 122.46, 118.42, 108.89, 99.99, 81.13, 60.63, 44.46,

41.76, 28.13; HRMS (ESI): *m/z* calcd for $C_{23}H_{24}N_3O_5$ $[M-H]^-$: 422.1716; found: 422.1717; HPLC: Daicel Chiralpak IC, *n*-hexane/*i*-PrOH = 9:1, Flow rate = 1.0 mL/min, λ = 210 nm, t_R = 16.1 min (major) and t_R = 19.1 min (minor).

(*R*)-*tert*-butyl (3-allyl-1-benzyl-6-methoxy-2-oxoindolin-3-yl)carbamate (3h)

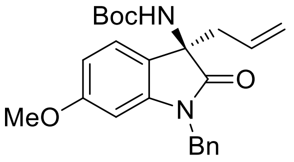

White solid, 76.7mg, 94% yield, 99.0:1.0 *er*; $[\alpha]_D^{27}$ = +29.6 (c = 1.0, $CHCl_3$); MP 105 - 106 °C; ^1H NMR (400 MHz, $CDCl_3$) δ 7.40 - 7.21 (m, 5H), 7.15 (d, *J* = 8.2 Hz, 1H), 6.51 (dd, *J* = 8.2, 2.3 Hz, 1H), 6.29 (d, *J* = 2.3 Hz, 1H), 5.70 (ddt, *J* = 17.3, 10.1, 7.4 Hz, 1H), 5.31 - 5.14 (m, 4H), 5.09 (s, 1H), 4.73 (s, 1H), 3.71 (s, 3H), 2.62 (dd, *J* = 13.4, 7.4 Hz, 1H), 2.47 (dd, *J* = 13.4, 7.4 Hz, 1H), 1.28 (s, 9H); ^{13}C NMR (101 MHz, $CDCl_3$) δ 177.19, 160.33, 153.76, 143.63, 135.85, 130.26, 128.69, 127.52, 127.41, 123.46, 121.21, 106.08, 97.37, 80.26, 60.53, 55.33, 44.08, 42.43, 28.13; HRMS (ESI): *m/z* calcd for $C_{24}H_{29}N_2O_4$ $[M+H]^+$: 409.2122; found: 409.2126; HPLC: Daicel Chiralpak IC, *n*-hexane/*i*-PrOH = 4:1, Flow rate = 1.0 mL/min, λ = 210 nm, t_R = 12.2 min (minor) and t_R = 14.2 min (major).

(*R*)-*tert*-butyl (3-allyl-1-benzyl-6-fluoro-2-oxoindolin-3-yl)carbamate (3i)

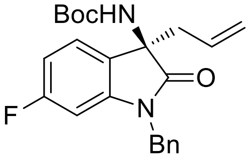

White solid, 77.6mg, 98% yield, 98.7:1.3 *er*; $[\alpha]_D^{27}$ = +10.8 (c = 1.0, $CHCl_3$); MP 97 - 98 °C; ^1H NMR (400 MHz, $CDCl_3$) δ 7.39 - 7.24 (m, 5H), 7.19 (ddd, *J* = 7.9, 5.2, 2.2 Hz, 1H), 6.70 (t, *J* = 8.8 Hz, 1H), 6.43 (d, *J* = 8.9 Hz, 1H), 5.78 - 5.54 (m, 1H), 5.31 - 5.16 (m, 3H), 5.10 (s, 1H), 4.76 (s, 1H), 2.62 (dd, *J* = 13.6, 7.4 Hz, 1H), 2.48 (dd, *J* = 13.5, 7.5 Hz, 1H), 1.29 (s, 9H); ^{13}C NMR (101 MHz, $CDCl_3$) δ 177.00, 163.13 (d, *J* = 245.3 Hz), 153.69, 143.86 (d, *J* = 11.6 Hz), 135.35, 129.76, 128.81, 127.74, 127.40, 123.73 (d, *J* = 9.7 Hz), 121.63, 108.61 (d, *J* = 22.6 Hz), 98.09 (d, *J* = 26.2 Hz), 80.55, 60.53, 44.23, 42.23, 28.11; ^{19}F NMR (376 MHz, $CDCl_3$) δ -116.68; HRMS (ESI): *m/z* calcd for $C_{23}H_{26}FN_2O_3$ $[M+H]^+$: 397.1922; found: 397.1918; HPLC: Daicel Chiralpak IC, *n*-hexane/*i*-PrOH = 4:1, Flow rate = 1.0 mL/min, λ = 210 nm, t_R = 7.1 min (minor) and t_R = 8.4 min (major).

(*R*)-*tert*-butyl (3-allyl-1-benzyl-6-chloro-2-oxoindolin-3-yl)carbamate (3j)

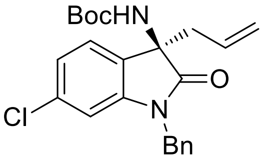

White solid, 79.1mg, 96% yield, 97.5:2.5 *er*; $[\alpha]_D^{27}$ = +19.8 (c = 1.0, $CHCl_3$); MP 127 - 128 °C; ^{1}H NMR (400 MHz, $CDCl_3$) δ 7.38 - 7.26 (m, 5H), 7.16 (d, *J* = 7.9 Hz, 1H), 7.00 (dd, *J* = 7.9, 1.9 Hz, 1H), 6.69 (s, 1H), 5.66 (ddt, *J* = 17.4, 10.0, 7.4 Hz, 1H), 5.28 - 5.16 (m, 3H), 5.08 (s, 1H), 4.76 (s, 1H), 2.61 (dd, *J* = 13.4, 7.3 Hz, 1H), 2.47 (dd, *J* = 13.4, 7.5 Hz, 1H), 1.29 (s, 9H); ^{13}C NMR (101 MHz, $CDCl_3$) δ 176.71, 153.70, 143.58, 135.32, 134.31, 129.63, 128.82, 127.74, 127.35, 123.63, 122.45, 121.72, 109.82, 80.65, 60.61, 44.19, 42.09, 28.12; HRMS (ESI): *m/z* calcd for $C_{23}H_{25}ClN_2NaO_3$ $[M+Na]^+$: 435.1451, 437.1422; found: 435.1450, 437.1370; HPLC: Daicel Chiralpak IC, *n*-hexane/*i*-PrOH = 4:1, Flow rate = 1.0 mL/min, λ = 210 nm, t_R = 6.6 min (minor) and t_R = 7.7 min (major).

(*R*)-*tert*-butyl (3-allyl-1-benzyl-6-bromo-2-oxoindolin-3-yl)carbamate (3k)

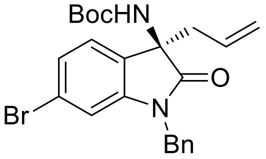

White solid, 84.8mg, 93% yield, 98.6:1.4 *er*; $[\alpha]_D^{27}$ = +26.4 (c = 1.0, $CHCl_3$); MP 139 - 140 °C; ^{1}H NMR (400 MHz, $CDCl_3$) δ 7.39 - 7.27 (m, 5H), 7.16 (dd, *J* = 7.9, 1.6 Hz, 1H), 7.11 (d, *J* = 7.9 Hz, 1H), 6.83 (s, 1H), 5.66 (ddt, *J* = 17.3, 10.0, 7.4 Hz, 1H), 5.27 - 5.15 (m, 3H), 5.06 (s, 1H), 4.76 (s, 1H), 2.61 (dd, *J* = 13.5, 7.3 Hz, 1H), 2.47 (dd, *J* = 13.4, 7.5 Hz, 1H), 1.30 (s, 9H); ^{13}C NMR (101 MHz, $CDCl_3$) δ 176.61, 153.69, 143.71, 135.31, 129.59, 128.83, 127.74, 127.34, 125.40, 124.00, 122.18, 121.77, 112.52, 80.67, 60.66, 44.17, 42.02, 28.13; HRMS (ESI): *m/z* calcd for $C_{23}H_{26}BrN_2O_3$ $[M+H]^+$: 457.1121, 459.1106; found: 457.1121, 459.1104; HPLC: Daicel Chiralpak IC, *n*-hexane/*i*-PrOH = 4:1, Flow rate = 1.0 mL/min, λ = 210 nm, t_R = 6.6 min (minor) and t_R = 7.7 min (major).

(*R*)-*tert*-butyl (3-allyl-1-benzyl-2-oxo-6-(trifluoromethyl)indolin-3-yl)carbamate (3l)

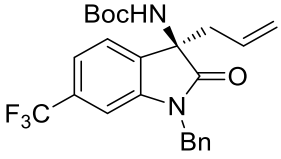

White solid, 88.3mg, 99% yield, 98.8:1.2 *er*; $[\alpha]_D^{28}$ = +17.4 (c = 1.0, $CHCl_3$); MP 170 - 171 °C; ^{1}H NMR (400 MHz, $CDCl_3$) δ 7.47 - 7.27 (m, 7H), 6.90 (s, 1H), 5.67 (ddt, J = 17.3, 10.0, 7.4 Hz, 1H), 5.26 - 5.20 (m, 3H), 5.11 (br, 1H), 4.86 (br, 1H), 2.63 (dd, J = 13.5, 7.3 Hz, 1H), 2.49 (dd, J = 13.5, 7.5 Hz,1H), 1.33 (s, 9H); ^{13}C NMR (101 MHz, $CDCl_3$) δ 176.57, 153.74, 142.99, 135.18, 130.98 (q, J = 32.4 Hz), 129.35, 128.85,

127.82, 127.41, 125.20, 122.86, 122.49, 121.90, 119.65 (q, J = 4.3 Hz), 105.77, 80.80, 60.81, 44.26, 41.94, 28.06; ^{19}F NMR (376 MHz, $CDCl_3$) δ -62.44; HRMS (ESI): *m/z* calcd for $C_{24}H_{25}F_3N_2NaO_3$ $[M+Na]^+$: 469.1715; found: 469.1714; HPLC: Daicel Chiralpak IC, *n*-hexane/*i*-PrOH = 4:1, Flow rate = 1.0 mL/min, λ = 210 nm, t_R = 5.3 min (minor) and t_R = 5.7 min (major).

(*R*)-*tert*-butyl (3-allyl-1-benzyl-7-methyl-2-oxoindolin-3-yl)carbamate (3m)

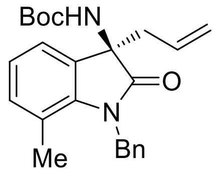

White solid, 77.6mg, 99% yield, 97.8:2.2 *er*; $[\alpha]_D^{27}$ = -0.8 (c = 1.0, $CHCl_3$); MP 116 - 117 °C; ^{1}H NMR (400 MHz, $CDCl_3$) δ 7.36 - 7.19 (m, 5H), 7.12 (t, *J* = 4.4 Hz, 1H), 6.95 (d, *J* = 4.5 Hz, 2H), 5.78 (ddt, *J* = 17.5, 10.4, 7.5 Hz, 1H), 5.29 - 5.20 (m, 5H), 2.62 (dd, *J* = 13.5, 7.4 Hz, 1H), 2.50 (dd, *J* = 13.5, 7.5 Hz, 1H), 2.25 (s, 3H), 1.30 (s, 9H); ^{13}C NMR (101 MHz, $CDCl_3$) δ 177.77, 153.71, 140.40, 137.93, 132.67, 131.42, 130.27, 128.74, 127.04, 125.98, 122.59, 121.35, 120.64, 119.71, 80.33, 60.20, 45.36, 42.82, 28.13, 18.85; HRMS (ESI): *m/z* calcd for $C_{24}H_{29}N_2O_3$ $[M+H]^+$: 393.2173; found: 393.2171; HPLC: Daicel Chiralpak IC, *n*-hexane/*i*-PrOH = 4:1, Flow rate = 1.0 mL/min, λ = 210 nm, t_R = 10.6 min (minor) and t_R = 15.5 min (major).

(*R*)-*tert*-butyl (3-allyl-1-benzyl-7-chloro-2-oxoindolin-3-yl)carbamate (3n)

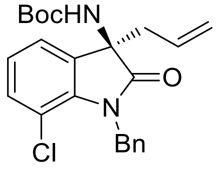

White solid, 79.1mg, 96% yield, 97.1:2.9 *er*; $[\alpha]_D^{27}$ = -7.0 (c = 1.0, $CHCl_3$); MP 117 - 118 °C; ^{1}H NMR (400 MHz, $CDCl_3$) δ 7.38 - 7.18 (m, 5H), 7.16 (d, *J* = 7.6 Hz, 2H), 6.97 (t, *J* = 7.7 Hz, 1H), 5.78 - 5.63 (m, 1H), 5.36 (s, 2H), 5.27 - 5.14 (m, 3H), 2.59 (dd, *J* = 13.5, 7.3 Hz, 1H), 2.48 (dd, *J* = 13.4, 7.5 Hz, 1H), 1.30 (s, 9H); ^{13}C NMR (101 MHz, $CDCl_3$) δ 177.34, 153.63, 138.46, 137.73, 131.24, 129.56, 128.42, 127.01, 126.74, 123.42, 121.83, 121.19, 115.51, 80.74, 60.43, 45.11, 42.52, 28.09; HRMS (ESI): *m/z* calcd for $C_{23}H_{25}ClN_2NaO_3$ $[M+Na]^+$: 435.1451, 437.1422; found: 435.1451, 437.1365; HPLC: Daicel Chiralpak IC, *n*-hexane/*i*-PrOH = 4:1, Flow rate = 1.0 mL/min, λ = 210 nm, t_R = 6.8 min (minor) and t_R = 9.3 min (major).

(*R*)-*tert*-butyl (3-allyl-1-benzyl-7-bromo-2-oxoindolin-3-yl)carbamate (3o)

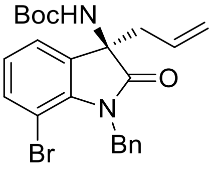

White solid, 89.4mg, 98% yield, 96.2:3.8 *er*; $[\alpha]_D^{27}$ = -8.8 (c = 1.0, $CHCl_3$); MP 117 - 118 °C; ^1H NMR (400 MHz, $CDCl_3$) δ 7.38 - 7.27 (m, 5H), 7.26 - 7.22 (m, 1H), 7.20 (d, *J* = 7.3 Hz, 1H), 6.91 (t, *J* = 7.7 Hz, 1H), 5.77 - 5.61 (m, 1H), 5.40 (q, *J* = 16.5 Hz, 2H), 5.27 - 5.10 (m, 3H), 2.59 (dd, *J* = 13.5, 7.3 Hz, 1H), 2.48 (dd, *J* = 13.5, 7.5 Hz, 1H), 1.31 (s, 9H); ^{13}C NMR (101 MHz, $CDCl_3$) δ 177.53, 153.66, 139.95, 137.67, 134.60, 134.05, 129.56, 128.41, 126.94, 126.64, 123.81, 121.80, 121.75, 102.56, 80.74, 60.40, 44.77, 42.55, 28.11; HRMS (ESI): *m/z* calcd for $C_{23}H_{26}BrN_2O_3$ $[M+H]^+$: 457.1121, 459.1106; found: 457.1123, 459.1105; HPLC: Daicel Chiralpak IC, *n*-hexane/*i*-PrOH = 4:1, Flow rate = 1.0 mL/min, λ = 210 nm, t_R = 6.9 min (minor) and t_R = 10.2 min (major).

(*R*)-*tert*-butyl (3-allyl-1-benzyl-2-oxo-7-(trifluoromethyl)indolin-3-yl)carbamate (3p)

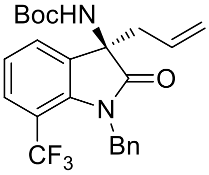

White solid, 87.4mg, 98% yield, 91.7:8.3 *er*; $[\alpha]_D^{28}$ = +27.2 (c = 1.0, $CHCl_3$); MP 129 - 130 °C; ^1H NMR (400 MHz, $CDCl_3$) δ 7.56 (d, J = 8.1 Hz, 1H), 7.46 (d, J = 7.3 Hz, 1H), 7.35 - 7.18 (m, 5H), 7.15 (t, J = 7.8 Hz, 1H), 5.69 (ddt, J = 17.4, 10.2, 7.4 Hz, 1H), 5.34 (s, 1H), 5.30 - 4.95 (m, 4H), 2.60 (dd, J = 13.5, 7.3 Hz, 1H), 2.49 (dd, J = 13.5, 7.6 Hz, 1H), 1.29 (s, 9H); ^{13}C NMR (101 MHz, $CDCl_3$) δ 178.13, 153.63, 140.64, 136.47, 129.31, 128.20, 126.81 (q, J = 6.0 Hz), 126.23, 126.05, 124.78, 122.01, 112.75 (q, J = 32.7 Hz), 80.9, 59.26, 46.04 (q, J = 4.0 Hz), 42.55, 28.01; ^{19}F NMR (376 MHz, $CDCl_3$) δ -54.44; HRMS (ESI): *m/z* calcd for $C_{24}H_{24}F_3N_2O_3$ $[M-H]^-$: 445.1739; found: 445.1740; HPLC: Daicel Chiralpak IC, *n*-hexane/*i*-PrOH = 4:1, Flow rate = 1.0 mL/min, λ = 210 nm, t_R = 4.9 min (minor) and t_R = 6.9 min (major).

(*R*)-*tert*-butyl (3-allyl-2-oxoindolin-3-yl)carbamate (3q)

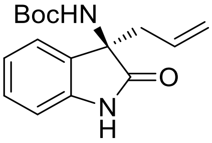

White solid, 40.3mg, 70% yield, 98.8:1.2 *er*; $[\alpha]_D^{27}$ = +12.8 (c = 0.5, $CHCl_3$); MP 124 - 125 °C; ^1H NMR (400 MHz, $CDCl_3$) δ 8.14 (s, 1H), 7.24 - 7.21 (m, 2H), 7.03 (t, *J* = 7.5 Hz, 1H), 6.86 (d, *J* = 7.8 Hz, 1H), 5.76 (dq, *J* = 17.0, 9.0, 8.4 Hz, 1H), 5.29 -

5.15 (m, 3H), 2.58 (dd, *J* = 13.6, 7.6 Hz, 1H), 2.48 (dd, *J* = 13.6, 7.3 Hz, 1H), 1.26 (s, 9H); ^{13}C NMR (101 MHz, $CDCl_3$) δ 179.21, 153.98, 140.55, 130.92, 129.95, 128.69, 122.90, 122.41, 121.40, 110.33, 80.71, 61.36, 41.90, 28.01; HRMS (ESI): *m/z* calcd for $C_{16}H_{21}N_2O_3$ $[M+H]^+$: 289.1547; found: 289.1547; HPLC: Daicel Chiralpak IC, *n*-hexane/*i*-PrOH = 4:1, Flow rate = 1.0 mL/min, λ = 210 nm, t_R = 5.6 min (minor) and t_R = 10.7 min (major).

(*R*)-*tert*-butyl (3-allyl-2-oxo-1-phenylindolin-3-yl)carbamate (3r)

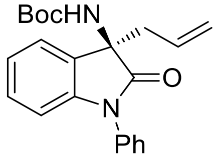

White solid, 69.9mg, 96% yield, 92.4:7.6 *er*; $[\alpha]_D^{27}$ = +22.6 (c = 1.0, $CHCl_3$); MP 79 - 80 °C; ^{1}H NMR (400 MHz, $CDCl_3$) δ 7.56 - 7.42 (m, 4H), 7.39 (t, *J* = 7.3 Hz, 1H), 7.32 (d, *J* = 7.3 Hz, 1H), 7.22 (t, *J* = 7.7 Hz, 1H), 7.09 (t, *J* = 7.5 Hz, 1H), 6.83 (d, *J* = 7.9 Hz, 1H), 5.73 (ddt, *J* = 17.4, 10.2, 7.4 Hz, 1H), 5.33 - 5.09 (m, 3H), 2.68 (dd, *J* = 13.3, 7.3 Hz, 1H), 2.58 (dd, *J* = 13.3, 7.5 Hz, 1H), 1.27 (s, 9H); ^{13}C NMR (101 MHz, $CDCl_3$) δ 176.05, 153.81, 143.17, 134.62, 130.28, 129.88, 129.53, 128.56, 127.93, 126.52, 122.99, 122.93, 121.44, 109.37, 80.52, 61.09, 42.40, 28.14; HRMS (ESI): *m/z* calcd for $C_{25}H_{25}N_2O_3$ $[M+H]^+$: 365.1860; found: 365.1859; HPLC: Daicel Chiralpak IC, *n*-hexane/*i*-PrOH = 4:1, Flow rate = 1.0 mL/min, λ = 210 nm, t_R = 6.7 min (minor) and t_R = 24.9 min (major).

(*R*)-*tert*-butyl (3-allyl-1-methyl-2-oxoindolin-3-yl)carbamate (3s)

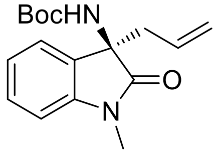

White solid, 51.3mg, 85% yield, 98.9:1.1 *er*; $[\alpha]_D^{27}$ = +47.2 (c = 0.5, $CHCl_3$); MP 129 - 130 °C; ^{1}H NMR (400 MHz, $CDCl_3$) δ 7.30 (t, *J* = 7.7 Hz, 1H), 7.24 (d, *J* = 7.3 Hz, 1H), 7.06 (t, *J* = 7.5 Hz, 1H), 6.84 (d, *J* = 7.7 Hz, 1H), 5.72 (ddt, *J* = 17.4, 10.2, 7.4 Hz, 1H), 5.23 (d, *J* = 9.1 Hz, 1H), 5.19 (s, 1H), 5.15 (s, 1H), 3.23 (s, 3H), 2.56 (dd, *J* = 13.5, 7.7 Hz, 1H), 2.42 (dd, *J* = 13.5, 7.2 Hz, 1H), 1.22 (s, 9H); ^{13}C NMR (101 MHz, $CDCl_3$) δ 176.62, 153.68, 143.14, 130.03, 128.69, 122.62, 122.47, 121.24, 108.06, 80.29, 60.83, 42.04, 27.98, 26.36; HRMS (ESI): *m/z* calcd for $C_{16}H_{21}N_2O_3$ $[M+H]^+$: 303.1703; found: 303.1698; HPLC: Daicel Chiralpak IC, *n*-hexane/*i*-PrOH = 4:1, Flow rate = 1.0 mL/min, λ = 210 nm, t_R = 14.0 min (minor) and t_R = 18.9 min (major).

(*R*)-*tert*-butyl (1,3-diallyl-2-oxoindolin-3-yl)carbamate (3t)

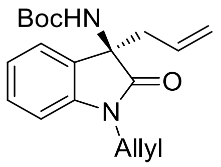

White solid, 64.9mg, 99% yield, 98.4:1.6 *er*; $[\alpha]_D^{27}$ = +40.2 (c = 1.0, $CHCl_3$); MP 130 - 131 °C; ^{1}H NMR (400 MHz, $CDCl_3$) δ 7.30 - 7.22 (m, 2H), 7.05 (t, *J* = 7.5 Hz, 1H), 6.82 (d, *J* = 7.5 Hz, 1H), 5.84 (ddt, *J* = 17.2, 10.3, 5.2 Hz, 1H), 5.70 (ddt, *J* = 17.4, 10.1, 7.4 Hz, 1H), 5.36 - 5.26 (m, 1H), 5.26 - 5.15 (m, 3H), 5.15 (br, 1H), 4.55 (br, 1H), 4.18 (br, 1H), 2.59 (dd, *J* = 13.4, 7.5 Hz, 1H), 2.46 (dd, *J* = 13.4, 7.4 Hz, 1H), 1.24 (s, 9H); ^{13}C NMR (101 MHz, $CDCl_3$) δ 176.36, 153.66, 142.36, 131.51, 130.51, 130.02, 128.58, 122.67, 122.44, 121.33, 117.53, 109.02, 80.31, 60.82, 42.57, 42.22, 28.05; HRMS (ESI): *m/z* calcd for $C_{19}H_{25}N_2O_3$ $[M+H]^+$: 329.1860; found: 329.1864; HPLC: Daicel Chiralpak IC, *n*-hexane/*i*-PrOH = 4:1, Flow rate = 1.0 mL/min, λ = 210 nm, t_R = 10.8 min (minor) and t_R = 12.7 min (major).

(*R*)-*tert*-butyl (3-allyl-1-(methoxymethyl)-2-oxoindolin-3-yl)carbamate (3u)

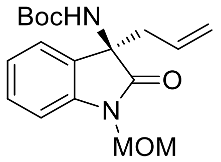

White solid, 65.1mg, 98% yield, 99.3:0.7 *er*; $[\alpha]_D^{27}$ = +23.4 (c = 1.0, $CHCl_3$); MP 105 - 106 °C; ^{1}H NMR (400 MHz, $CDCl_3$) δ 7.34 - 7.22 (m, 2H), 7.09 (t, *J* = 8.0 Hz, 1H), 7.04 (d, *J* = 7.8 Hz, 1H), 5.70 (ddt, *J* = 17.4, 10.1, 7.4 Hz, 1H), 5.28 - 5.16 (m, 4H), 5.09 (br, 1H), 3.38 (s, 3H), 2.59 (dd, *J* = 13.4, 7.3 Hz, 1H), 2.47 (dd, *J* = 13.4, 7.6 Hz, 1H), 1.25 (s, 9H); ^{13}C NMR (101 MHz, $CDCl_3$) δ 177.20, 153.63, 141.49, 129.89, 128.84, 123.00, 122.68, 121.40, 109.54, 80.43, 71.69, 61.16, 56.49, 42.30, 28.03; HRMS (ESI): *m/z* calcd for $C_{18}H_{24}N_2NaO_4$ $[M+Na]^+$: 355.1634; found: 355.1633; HPLC: Daicel Chiralpak AD - H, *n*-hexane/*i*-PrOH = 4:1, Flow rate = 1.0 mL/min, λ = 210 nm, t_R = 7.2 min (major) and t_R = 22.7 min (minor).

(*R*)-*tert*-butyl (3-allyl-1-(2,2-diethoxyethyl)-2-oxoindolin-3-yl)carbamate (3v)

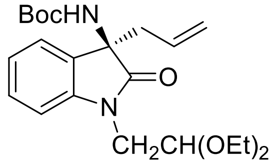

White solid, 80.0mg, 99% yield, 99.5:0.5 *er*; $[\alpha]_D^{27}$ = +52.0 (c = 0.5, $CHCl_3$); MP 47 - 48 °C; ^{1}H NMR (400 MHz, $CDCl_3$) δ 7.29 - 7.18 (m, 2H), 7.04 - 7.00 (m, 2H), 5.70 (ddt, *J* = 17.3, 10.1, 7.4 Hz, 1H), 5.25 - 5.13 (m, 2H), 5.11 (br, 1H), 4.70 (t, *J* = 5.4 Hz, 1H), 4.07 (d, *J* = 14.3 Hz, 1H), 3.73 (dqd, *J* = 9.3, 7.0, 4.7 Hz, 2H), 3.61 - 3.45 (m, 3H), 2.55 (dd, *J* = 13.4, 7.6 Hz, 1H), 2.43 (dd, *J* = 13.4, 7.2 Hz, 1H), 1.32 - 1.06 (m, 15H); ^{13}C NMR (101 MHz, $CDCl_3$) δ 176.87, 153.64, 142.88, 130.13, 128.38, 122.37, 122.25, 121.05, 109.57, 100.59, 80.25, 63.62, 62.91, 60.62, 43.65, 42.18, 27.98, 15.27;

HRMS (ESI): *m/z* calcd for $C_{22}H_{32}N_2NaO_5$ $[M+Na]^+$: 427.2209; found: 427.2207; HPLC: Daicel Chiralpak IC, *n*-hexane/*i*-PrOH = 4:1, Flow rate = 1.0 mL/min, λ = 210 nm, t_R = 7.1 min (minor) and t_R = 8.3 min (major).

(*R*)-*tert*-butyl (1-acetyl-3-allyl-2-oxoindolin-3-yl)carbamate (3w)

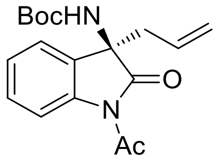

White solid, 61.4mg, 93% yield, 96.7:3.3 *er*; $[\alpha]_D^{27}$ = +8.4 (c = 1.0, $CHCl_3$); MP 129 - 130 °C; ^{1}H NMR (400 MHz, $CDCl_3$) δ 8.23 (d, *J* = 8.2 Hz, 1H), 7.33 (t, *J* = 7.6 Hz, 1H), 7.29 - 7.16 (m, 2H), 5.58 (ddt, *J* = 17.3, 10.5, 7.5 Hz, 1H), 5.30 (s, 1H), 5.21 (d, *J* = 7.3 Hz, 1H), 5.18 (s, 1H), 2.67 (s, 3H), 2.56 (dd, *J* = 13.3, 7.0 Hz, 1H), 2.49 (dd, *J* = 13.4, 7.7 Hz, 1H), 1.20 (s, 9H); ^{13}C NMR (101 MHz, $CDCl_3$) δ 177.36, 170.73, 153.65, 139.49, 129.12, 125.20, 122.14, 121.89, 116.54, 81.10, 61.42, 42.71, 27.91, 26.64; HRMS (ESI): *m/z* calcd for $C_{18}H_{22}N_2NaO_4$ $[M+Na]^+$: 353.1477; found: 353.1477; HPLC: Daicel Chiralpak IC, *n*-hexane/*i*-PrOH = 4:1, Flow rate = 1.0 mL/min, λ = 210 nm, t_R = 4.6 min (minor) and t_R = 5.4 min (major).

(*R*)-*tert*-butyl (3-allyl-1-(2-methylbenzyl)-2-oxoindolin-3-yl)carbamate (3x)

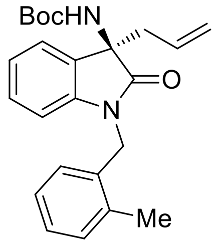

White solid, 77.6mg, 99% yield, 98.5:1.5 *er*; $[\alpha]_D^{27}$ = -1.8 (c = 1.0, $CHCl_3$); MP 84 - 85 °C; ^{1}H NMR (400 MHz, $CDCl_3$) δ 7.28 (d, *J* = 7.4 Hz, 1H), 7.24 - 7.14 (m, 4H), 7.15 - 7.07 (m, 1H), 7.04 (t, *J* = 7.5 Hz, 1H), 6.62 (d, *J* = 7.7 Hz, 1H), 5.80 (ddt, *J* = 17.5, 10.2, 7.4 Hz, 1H), 5.35 - 5.21 (m, 3H), 5.16 (br, 1H), 4.75 (br, 1H), 2.67 (dd, *J* = 13.5, 7.5 Hz, 1H), 2.54 (dd, *J* = 13.5, 7.4 Hz, 1H), 2.41 (s, 3H), 1.30 (s, 9H); ^{13}C NMR (101 MHz, $CDCl_3$) δ 176.90, 153.71, 142.61, 135.44, 133.36, 130.41, 130.17, 128.70, 127.30, 126.42, 126.19, 122.70, 122.57, 121.55, 109.37, 80.37, 60.88, 42.35, 42.17, 28.14, 19.31; HRMS (ESI): *m/z* calcd for $C_{24}H_{29}N_2O_3$ $[M+H]^+$: 393.2178; found: 393.2178; HPLC: Daicel Chiralpak IC, *n*-hexane/*i*-PrOH = 4:1, Flow rate = 1.0 mL/min, λ = 210 nm, t_R = 7.4 min (minor) and t_R = 11.6 min (major).

(*R*)-*tert*-butyl (3-allyl-1-(2-fluorobenzyl)-2-oxoindolin-3-yl)carbamate (3y)

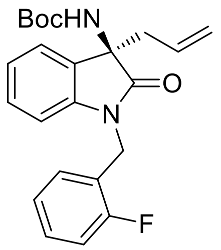

White solid, 77.6mg, 98% yield, 99.3:0.7 *er*; $[\alpha]_D^{27}$ = +18.0 (c = 1.0, $CHCl_3$); MP 86 - 87 °C; ^1H NMR (400 MHz, $CDCl_3$) δ 7.40 (t, *J* = 7.7 Hz, 1H), 7.29 - 7.16 (m, 3H), 7.11 - 6.99 (m, 3H), 6.77 (d, *J* = 7.8 Hz, 1H), 5.71 (ddt, *J* = 17.4, 10.1, 7.4 Hz, 1H), 5.31 - 5.05 (m, 4H), 4.90 (br, 1H), 2.61 (dd, *J* = 13.5, 7.5 Hz, 1H), 2.48 (dd, *J* = 13.4, 7.4 Hz, 1H), 1.27 (s, 9H); ^{13}C NMR (101 MHz, $CDCl_3$) δ 176.90, 160.56 (d, *J* = 246.1 Hz), 153.73, 142.01, 129.99, 129.82, 129.27 (d, *J* = 6.9 Hz), 128.75, 124.47 (d, *J* = 3.7 Hz), 122.85 (d, *J* = 14.0 Hz), 122.69 (d, *J* = 3.4 Hz), 121.45 , 115.27 (d, *J* = 21.3 Hz), 108.85, 80.45, 60.87, 42.24 , 37.29 (d, *J* = 5.3 Hz), 28.05; ^{19}F NMR (376 MHz, $CDCl_3$) δ -124.04; HRMS (ESI): *m/z* calcd for $C_{23}H_{26}FN_2O_3$ $[M+H]^+$: 397.1927; found: 397.1922; HPLC: Daicel Chiralpak IC, *n*-hexane/*i*-PrOH = 2:1, Flow rate = 1.0 mL/min, λ = 210 nm, t_R = 6.1 min (minor) and t_R = 7.1 min (major).

(*R*)-*tert*-butyl (3-allyl-1-(3-methylbenzyl)-2-oxoindolin-3-yl)carbamate (3z)

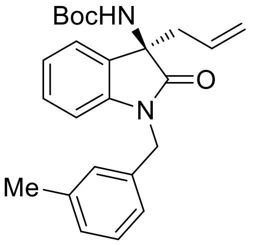

White solid, 77.6mg, 99% yield, 98.9:1.1 *er*; $[\alpha]_D^{27}$ = +7.2 (c = 1.0, $CHCl_3$); MP 85 - 86 °C; ^1H NMR (400 MHz, $CDCl_3$) δ 7.25 (d, *J* = 6.1 Hz, 1H), 7.22 - 7.13 (m, 4H), 7.06 (d, *J* = 7.5 Hz, 1H), 7.02 (t, *J* = 7.5 Hz, 1H), 6.70 (d, *J* = 7.8 Hz, 1H), 5.82 - 5.61 (m, 1H), 5.30 - 5.15 (m, 3H), 5.07 (br, 1H), 4.77 (br, 1H), 2.63 (dd, *J* = 13.4, 7.4 Hz, 1H), 2.50 (dd, *J* = 13.4, 7.4 Hz, 1H), 2.31 (s, 3H), 1.26 (s, 9H); ^{13}C NMR (101 MHz, $CDCl_3$) δ 176.76, 153.74, 142.43, 138.38, 135.81, 130.51, 130.13, 128.62, 128.51, 128.28, 128.14, 124.45, 122.66, 122.49, 121.36, 109.21, 80.34, 60.90, 44.05, 42.28, 28.09, 21.42. HRMS (ESI): *m/z* calcd for $C_{24}H_{29}N_2O_3$ $[M+H]^+$: 393.2178; found: 393.2174; HPLC: Daicel Chiralpak IC, *n*-hexane/*i*-PrOH = 4:1, Flow rate = 1.0 mL/min, λ = 210 nm, t_R = 10.2 min (minor) and t_R = 14.1 min (major).

(*R*)-*tert*-butyl (3-allyl-1-(3-fluorobenzyl)-2-oxoindolin-3-yl)carbamate (3aa)

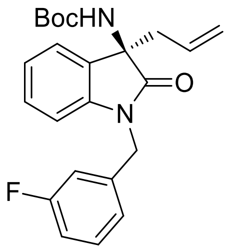

White solid, 78.4mg, 99% yield, 98.1:1.9 *er*; $[\alpha]_D^{27}$ = +15.4 (c = 1.0, $CHCl_3$); MP 62 - 63 °C; ^{1}H NMR (400 MHz, $CDCl_3$) δ 7.33 - 7.23 (m, 2H), 7.20 - 7.14 (m, 2H), 7.10 (d, *J* = 9.7 Hz, 1H), 7.04 (t, *J* = 7.5 Hz, 1H), 6.94 (td, *J* = 8.5, 2.5 Hz, 1H), 6.66 (d, *J* = 7.8 Hz, 1H), 5.69 (ddt, *J* = 17.3, 10.0, 7.4 Hz, 1H), 5.33 - 5.14 (m, 3H), 5.02 (s, 2H), 2.63 (dd, *J* = 13.4, 7.3 Hz, 1H), 2.50 (dd, *J* = 13.4, 7.5 Hz, 1H), 1.28 (s, 9H); ^{13}C NMR (101 MHz, $CDCl_3$) δ 176.77, 163.10 (d, *J* = 246.3 Hz), 153.71, 142.08, 138.47 (d, *J* = 6.6 Hz), 130.45, 130.20 (d, *J* = 8.0 Hz), 129.96, 128.68, 122.93, 122.74 (d, *J* = 3.5 Hz), 121.51, 114.60, 114.39 (d, *J* = 21.3 Hz), 114.53, 114.30 (d, *J* = 22.6 Hz), 108.99, 80.46, 60.90, 43.56, 42.23, 28.08; ^{19}F NMR (376 MHz, $CDCl_3$) δ -112.57; HRMS (ESI): *m/z* calcd for $C_{23}H_{26}FN_2O_3$ $[M+H]^+$: 397.1927; found: 397.1926; HPLC: Daicel Chiralpak IC, *n*-hexane/*i*-PrOH = 4:1, Flow rate = 1.0 mL/min, λ = 210 nm, t_R = 8.1 min (minor) and t_R = 10.0 min (major).

(*R*)-*tert*-butyl (3-allyl-1-(4-methylbenzyl)-2-oxoindolin-3-yl)carbamate (3ab)

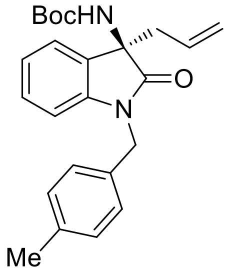

White solid, 77.6mg, 99% yield, 98.6:1.4 *er*; $[\alpha]_D^{27}$ = +7.8 (c = 1.0, $CHCl_3$); MP 121 - 122 °C; ^{1}H NMR (400 MHz, $CDCl_3$) δ 7.25 (d, *J* = 7.8 Hz, 3H), 7.16 (t, *J* = 7.8 Hz, 1H), 7.11 (d, *J* = 7.8 Hz, 2H), 7.01 (t, *J* = 7.5 Hz, 1H), 6.71 (d, *J* = 7.8 Hz,1H), 5.72 (ddt, *J* = 17.2, 9.8, 7.3 Hz, 1H), 5.33 - 5.00 (m, 4H), 4.71 (br, 1H), 2.62 (dd, *J* = 13.4, 7.5 Hz, 1H), 2.48 (dd, *J* = 13.4, 7.3 Hz, 1H), 2.31 (s, 3H), 1.26 (s, 9H); ^{13}C NMR (101 MHz, $CDCl_3$) δ 176.71, 153.74, 142.41, 137.14, 132.89, 130.58, 130.13, 129.34, 128.58, 127.43, 122.68, 122.44, 121.31, 109.18, 80.34, 60.88, 43.84, 42.29, 28.07, 21.09; HRMS (ESI): *m/z* calcd for $C_{24}H_{29}N_2O_3$ $[M+H]^+$: 393.2178; found: 393.2172; HPLC: Daicel Chiralpak IC, *n*-hexane/*i*-PrOH = 4:1, Flow rate = 1.0 mL/min, λ = 210 nm, t_R = 11.2 min (minor) and t_R = 14.6 min (major).

(*R*)-*tert*-butyl (3-allyl-1-(4-(tert-butyl)benzyl)-2-oxoindolin-3-yl)carbamate (3ac)

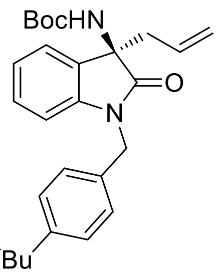

White solid, 75.5mg, 87% yield, 98.0:2.0 *er*; $[\alpha]_D^{27}$ = +10.0 (c = 1.0, $CHCl_3$); MP 100 - 101 °C; ^{1}H NMR (400 MHz, $CDCl_3$) δ 7.34 - 7.29 (m, 3H), 7.30 - 7.22 (m, 2H), 7.18 (t, *J* = 7.7 Hz, 1H), 7.02 (t, *J* = 7.5 Hz, 1H), 6.74 (d, *J* = 7.8 Hz, 1H), 5.73 (ddt, *J* = 17.3, 10.0, 7.4 Hz, 1H), 5.28 - 4.99 (m, 4H), 4.71 (br, 1H), 2.63 (dd, *J* = 13.5, 7.5 Hz, 1H), 2.49 (dd, *J* = 13.5, 7.4 Hz, 1H), 1.29 (s, 18H); ^{13}C NMR (101 MHz, $CDCl_3$) δ 176.73, 153.74, 150.41, 142.41, 132.89, 130.12, 128.59, 127.17, 125.61, 122.69, 122.46, 121.41, 109.22, 80.39, 60.84, 43.72, 42.31, 34.50, 31.34, 28.04; HRMS (ESI): *m/z* calcd for $C_{27}H_{35}N_2O_3$ $[M+H]^+$: 435.2648; found: 435.2649; HPLC: Daicel Chiralpak IC, *n*-hexane/*i*-PrOH = 4:1, Flow rate = 1.0 mL/min, λ = 210 nm, t_R = 10.5 min (minor) and t_R = 12.5 min (major).

(*R*)-*tert*-butyl (3-allyl-1-(4-chlorobenzyl)-2-oxoindolin-3-yl)carbamate (3ad)

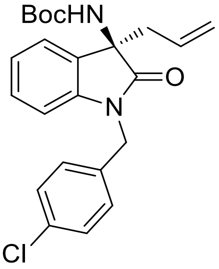

White solid, 81.6mg, 99% yield, 98.6:1.4 *er*; $[\alpha]_D^{27}$ = +21.6 (c = 1.0, $CHCl_3$); MP 131 - 132 °C; ^{1}H NMR (400 MHz, $CDCl_3$) δ 7.33 - 7.24 (m, 1H), 7.17 (t, *J* = 7.7 Hz, 1H), 7.03 (t, *J* = 7.5 Hz, 1H), 6.65 (d, *J* = 7.8 Hz, 1H), 5.68 (ddt, *J* = 17.3, 10.2, 7.4 Hz, 1H), 5.32 - 5.14 (m, 3H), 4.97 (br, 2H), 2.62 (dd, *J* = 13.4, 7.4 Hz, 1H), 2.48 (dd, *J* = 13.4, 7.5 Hz, 1H), 1.29 (s, 9H); ^{13}C NMR (101 MHz, $CDCl_3$) δ 176.74, 153.68, 142.02, 134.38, 133.33, 129.96, 128.84, 128.65, 122.74 (d, *J* = 6.6 Hz), 121.47, 109.01, 80.45, 60.83, 43.40, 42.24, 28.09; HRMS (ESI): *m/z* calcd for $C_{23}H_{25}ClN_2NaO_3$ $[M+Na]^+$: 435.1451, 437.1422; found: 435.1451, 437.1406; HPLC: Daicel Chiralpak IC, *n*-hexane/*i*-PrOH = 4:1, Flow rate = 1.0 mL/min, λ = 210 nm, t_R = 9.0 min (minor) and t_R = 11.0 min (major).

(*R*)-*tert*-butyl (3-allyl-1-(4-bromobenzyl)-2-oxoindolin-3-yl)carbamate (3ae)

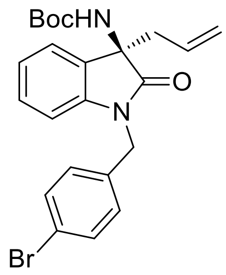

White solid, 90.3mg, 99% yield, 98.7:1.3 *er*; $[\alpha]_D^{27}$ = +15.3 (c = 0.3, $CHCl_3$); MP 138 - 139 °C; ^{1}H NMR (400 MHz, $CDCl_3$) δ 7.45 - 7.42 (m, 2H), 7.31 - 7.23 (m, 3H), 7.18 (t, *J* = 7.8 Hz, 1H), 7.04 (t, *J* = 7.6 Hz, 1H), 6.64 (d, *J* = 7.8 Hz, 1H), 5.74 - 5.63 (m, 1H), 5.34 - 5.16 (m, 3H), 4.94 (br, 2H), 2.62 (dd, *J* = 13.5, 7.4 Hz, 1H), 2.48 (dd, *J* = 13.5, 7.4 Hz, 1H), 1.29 (s, 9H); ^{13}C NMR (101 MHz, $CDCl_3$) δ 176.72, 153.69, 142.03, 134.92, 131.78, 130.46, 129.96, 129.19, 128.65, 122.78, 122.70, 121.43, 108.99, 80.43, 60.84, 43.45, 42.24, 28.10; HRMS (ESI): *m/z* calcd for $C_{23}H_{26}BrN_2O_3$ $[M+H]^+$: 457.1121, 459.1106; found: 457.1124, 459.1102; HPLC: Daicel Chiralpak IC, *n*-hexane/*i*-PrOH = 4:1, Flow rate = 1.0 mL/min, λ = 210 nm, t_R = 9.2 min (minor) and t_R = 11.4 min (major).

(*R*)-*tert*-butyl (3-allyl-2-oxo-1-(4-(trifluoromethyl)benzyl)indolin-3-yl)carbamate (3af)

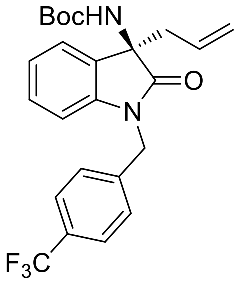

White solid, 86.5mg, 97% yield, 97.1:2.9 *er*; $[\alpha]_D^{27}$ = +21.8 (c = 1.0, $CHCl_3$); MP 127 - 128 °C; ^{1}H NMR (400 MHz, $CDCl_3$) δ 7.61 - 7.48 (m, 4H), 7.27 (d, *J* = 7.5 Hz, 1H), 7.18 (t, *J* = 7.7 Hz, 1H), 7.05 (t, *J* = 7.5 Hz, 1H), 6.62 (d, *J* = 7.8 Hz, 1H), 5.70 (ddt, *J* = 17.3, 10.1, 7.4 Hz, 1H), 5.32 - 5.16 (m, 3H), 5.02 (br, 2H), 2.63 (dd, *J* = 13.4, 7.3 Hz, 1H), 2.50 (dd, *J* = 13.4, 7.5 Hz, 1H), 1.30 (s, 9H); ^{13}C NMR (101 MHz, $CDCl_3$) δ 176.81, 153.70, 141.90, 139.95, 129.91, 128.71, 127.65, 125.67 (q, *J* = 3.8 Hz), 122.83, 121.53, 108.92, 80.50, 60.85, 43.58, 42.23, 28.10; ^{19}F NMR (376 MHz, $CDCl_3$) δ -62.47; HRMS (ESI): *m/z* calcd for $C_{24}H_{25}FN_2NaO_3$ $[M+Na]^+$: 469.1715; found: 469.1710; HPLC: Daicel Chiralpak IC, *n*-hexane/*i*-PrOH = 4:1, Flow rate = 1.0 mL/min, λ = 210 nm, t_R = 6.7 min (minor) and t_R = 7.7 min (major).

(*R*)-benzyl (3-allyl-1-benzyl-2-oxoindolin-3-yl)carbamate (3ag)

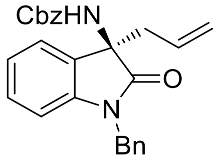

White solid, 65.9mg, 80% yield, 97.7:2.3 *er*; $[\alpha]_D^{27}$ = -2.4 (c = 1.0, $CHCl_3$); MP 92 - 93°C; ^{1}H NMR (400 MHz, $CDCl_3$) δ 7.50 - 7.20 (m, 10H), 7.17 (t, *J* = 7.7 Hz, 1H), 7.03 (t, *J* = 7.5 Hz, 1H), 6.66 (s, 1H), 5.72 (ddt, *J* = 17.3, 10.1, 7.4 Hz, 1H), 5.51 (s, 1H), 5.31 - 5.16 (m, 2H), 5.00 (s, 4H), 2.67 (dd, *J* = 13.4, 7.6 Hz, 1H), 2.53 (dd, *J* = 13.4, 7.3 Hz, 1H); ^{13}C NMR (101 MHz, $CDCl_3$) δ 176.33, 154.35, 142.45, 135.77, 129.90, 128.91, 128.73, 128.51, 128.23, 127.52, 127.26, 122.89, 122.68, 121.66, 109.48, 67.26, 60.92, 44.09, 42.13; HRMS (ESI): *m/z* calcd for $C_{26}H_{24}N_2NaO_3$ $[M+Na]^+$: 435.1685; found: 435.1678; HPLC: Daicel Chiralpak IC, *n*-hexane/*i*-PrOH = 4:1, Flow rate = 1.0 mL/min, λ = 210 nm, t_R = 20.3 min (major) and t_R = 30.8 min (minor).

(*R*)-ethyl (3-allyl-1-benzyl-2-oxoindolin-3-yl)carbamate (3ah)

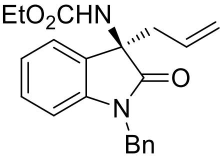

White solid, 60.2mg, 86% yield, 95.9:4.1 *er*; $[\alpha]_D^{27}$ = +19.6 (c = 0.5, $CHCl_3$); MP 110 - 111 °C; ^{1}H NMR (400 MHz, $CDCl_3$) δ 7.37 - 7.30 (m, 4H), 7.28 - 7.23 (m, 2H), 7.16 (t, *J* = 7.7 Hz, 1H), 7.02 (t, *J* = 7.5 Hz, 1H), 6.68 (d, *J* = 7.8 Hz, 1H), 5.74 (ddt, *J* = 17.4, 10.1, 7.4 Hz, 1H), 5.41 (s, 1H), 5.30 - 5.18 (m, 2H), 5.06 (d, *J* = 15.8 Hz, 1H), 4.86 (d, *J* = 15.9 Hz, 1H), 4.01 (s, 2H), 2.67 (dd, *J* = 13.4, 7.5 Hz, 1H), 2.53 (dd, *J* = 13.4, 7.3 Hz, 1H), 1.16 (s, 3H); ^{13}C NMR (101 MHz, $CDCl_3$) δ 176.46, 154.55, 142.42, 135.79, 130.01, 128.85, 128.72, 127.50, 127.27, 122.79, 122.62, 121.54, 109.36, 61.29, 60.80, 44.13, 42.13, 14.33; HRMS (ESI): *m/z* calcd for $C_{21}H_{23}N_2O_3$ $[M+H]^+$: 351.1709; found: 351.1709; HPLC: Daicel Chiralpak IC, *n*-hexane/*i*-PrOH = 4:1, Flow rate = 1.0 mL/min, λ = 210 nm, t_R = 14.3 min (major) and t_R = 18.0 min (minor).

(*R*)-3-allyl-1-benzyl-3-(phenylamino)indolin-2-one (3ai)

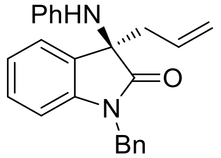

White solid, 69.4mg, 98% yield, 94.1:5.9 *er*; $[\alpha]_D^{27}$ = -80.4 (c = 1.0, $CHCl_3$); MP 83 - 84 °C; ^{1}H NMR (400 MHz, $CDCl_3$) δ 7.34 - 7.16 (m, 7H), 7.02 (t, *J* = 7.6 Hz, 1H), 6.93 (t, *J* = 6.9 Hz, 2H), 6.79 (d, *J* = 7.9 Hz, 1H), 6.67 (t, *J* = 7.3 Hz, 1H), 6.21 (d, *J* = 7.1 Hz, 2H), 5.85 - 5.66 (m, 1H), 5.22 (dd, *J* = 19.4, 13.8 Hz, 2H), 5.05 (d, *J* = 15.5 Hz, 1H), 4.80 (d, *J* = 15.4 Hz, 1H), 3.77 (s, 1H) 2.76 (dd, *J* = 13.4, 6.9 Hz, 1H), 2.63 (dd, *J* = 13.3, 7.8 Hz, 1H); ^{13}C NMR (101 MHz, $CDCl_3$) δ 177.67, 145.19, 141.90,

135.75, 130.49, 129.85, 129.06, 129.00, 128.76, 127.82, 127.77, 123.91, 123.03, 121.13, 119.33, 115.48, 109.70, 64.01, 44.80, 44.15; HRMS (ESI): *m/z* calcd for $C_{24}H_{23}N_2O$ $[M+H]^+$: 355.1810; found: 355.1809; HPLC: Daicel Chiralpak AD - H, *n*-hexane/*i*-PrOH = 4:1, Flow rate = 1.0 mL/min, λ = 210 nm, t_R = 24.2 min (minor) and t_R = 44.5 min (major).

(*R*)-ethyl 3-allyl-2,3-dihydrobenzo[d]isothiazole-3-carboxylate 1,1-dioxide (3aj)

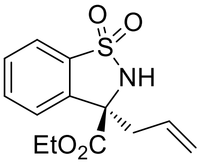

Colorless Oil, 51.1mg, 91% yield, 94.0:6.0 *er*; $[\alpha]_D^{28}$ = +61.6 (c = 1.0, $CHCl_3$); ^1H NMR (400 MHz, $CDCl_3$) δ 7.76 (d, J = 4.7 Hz, 2H), 7.74 (d, J = 5.4 Hz, 1H), 7.67 (t, J = 7.6 Hz, 1H), 7.58 (t, J = 7.5 Hz, 1H), 5.84 - 5.69 (m, 2H), 5.24 - 5.13 (m, 2H), 4.31 (ddq, J = 10.5, 7.1, 3.5 Hz, 2H), 2.96 (dd, J = 13.9, 7.8 Hz, 1H), 2.73 (dd, J = 13.9, 6.5 Hz, 1H), 1.33 (t, J = 7.1 Hz, 3H); ^{13}C NMR (101 MHz, $CDCl_3$) δ 169.51, 137.65, 135.45, 133.53, 130.93, 130.53, 125.01, 121.49, 120.89, 68.78, 63.60, 44.62, 14.15; HRMS (ESI): *m/z* calcd for $C_{13}H_{16}NO_4S$ $[M+H]^+$: 282.0800; found: 282.0798; HPLC: Daicel Chiralpak OD-H, *n*-hexane/*i*-PrOH = 9:1, Flow rate = 1.0 mL/min, λ = 210 nm, t_R = 15.1 min (minor) and t_R = 19.2 min (major).

***tert*-butyl (4-allyl-3-methyl-5-oxo-1-phenyl-4,5-dihydro-1H-pyrazol-4-yl) carbamate (3ak)**

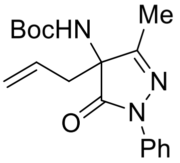

White solid, 64.5mg, 98% yield, 85.3:14.7 *er*; $[\alpha]_D^{28}$ = -7.2 (c = 1.0, $CHCl_3$); MP 143 - 144 °C; ^1H NMR (400 MHz, $CDCl_3$) δ 7.89 (d, J = 8.1 Hz, 2H), 7.38 (t, J = 7.8 Hz, 2H), 7.16 (t, J = 7.4 Hz, 1H), 5.69 (td, J = 17.0, 15.9, 7.9 Hz, 1H), 5.33 - 5.09 (m, 3H), 2.53 (dd, J = 13.5, 7.8 Hz, 1H), 2.45 (dd, J = 13.4, 7.0 Hz, 1H), 2.10 (s, 3H), 1.36 (s, 9H); ^{13}C NMR (101 MHz, $CDCl_3$) δ 172.37, 160.28, 153.86, 138.11, 128.76, 128.25, 124.88, 121.76, 118.72, 81.61 (d, J = 82.5 Hz), 65.88, 38.72, 28.06, 13.25; HRMS (ESI): *m/z* calcd for $C_{18}H_{23}N_3NaO_3$ $[M+Na]^+$: 352.1637; found: 352.1635; HPLC: Daicel Chiralpak IC, *n*-hexane/*i*-PrOH = 9:1, Flow rate = 1.0 mL/min, λ = 210 nm, t_R = 8.1 min (minor) and t_R = 10.3 min (major).

(*S*)-3-allyl-1-benzyl-3-hydroxyindolin-2-one (3al)

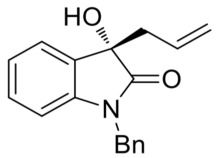

White solid, 54.1mg, 97% yield, 91.8:8.2 *er*; $[\alpha]_D^{28}$ = -39.2 (c = 1.0, $CHCl_3$); ^1H NMR (400 MHz, $CDCl_3$) δ 7.37 (d, J = 7.3 Hz, 1H), 7.32 - 7.21 (m, 5H), 7.17 (t, J = 7.8 Hz, 1H), 7.03 (t, J = 7.5 Hz, 1H), 6.67 (d, J = 7.8 Hz, 1H), 5.68 - 5.50 (m, 1H), 5.16 - 5.03 (m, 2H), 4.99 (d, J = 15.7 Hz, 1H), 4.70 (d, J = 15.7 Hz, 1H), 3.26 (br, 1H), 2.79 (dd, J = 13.4, 6.3 Hz, 1H), 2.67 (dd, J = 13.3, 8.4 Hz, 1H); ^{13}C NMR (101 MHz, $CDCl_3$) δ 178.17, 142.43, 135.43, 130.60, 129.80, 129.54, 128.75, 127.67, 127.31, 124.19, 123.13, 120.47, 109.48, 76.11, 43.85, 43.01; HRMS (ESI): *m/z* calcd for $C_{18}H_{17}NNaO_2$ $[M+Na]^+$: 302.1157; found: 302.1155; HPLC: Daicel Chiralpak OJ-H, *n*-hexane/*i*-PrOH = 4:1, Flow rate = 1.0 mL/min, λ = 210 nm, t_R = 7.6 min (minor) and t_R = 10.8 min (major).

(*R*)-*tert*-butyl (1-benzyl-3-(but-3-en-2-yl)-2-oxoindolin-3-yl)carbamate (3am)

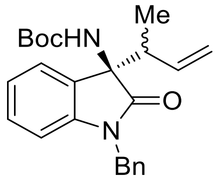

White solid, 71.3mg, 91% yield, 5.8:1 dr, 96.6:3.4 *er*; $[\alpha]_D^{27}$ = +53.2 (c = 0.5, $CHCl_3$); MP 109 -110 °C; ^1H NMR (400 MHz, $CDCl_3$) δ 7.46 - 7.36 (m, 2H), 7.31 (t, *J* = 7.3 Hz, 2H), 7.28 - 7.21 (m, 2H), 7.18 (t, *J* = 7.6 Hz, 1H), 7.03 (t, *J* = 7.4 Hz, 1H), 6.70 (d, *J* = 7.8 Hz, 1H), 5.90 (dt, *J* = 17.0, 9.8 Hz, 1H), 5.40 - 5.13 (m, 3H), 5.08 (d, *J* = 15.4 Hz, 1H), 4.76 (s, 1H), 2.63 (dt, *J* = 13.8, 7.1 Hz, 1H), 1.26 (s, 9H), 0.87 (d, *J* = 6.9 Hz, 3H); ^{13}C NMR (101 MHz, $CDCl_3$) δ 176.20, 153.87, 143.44, 137.08, 136.02, 128.62, 127.66, 127.48, 122.55, 122.49, 118.60, 108.79, 80.23, 63.52, 46.46, 44.23, 28.05, 14.52; HRMS (ESI): *m/z* calcd for $C_{24}H_{29}N_2O_3$ $[M+H]^+$: 393.2178; found: 393.2176; HPLC: Daicel Chiralpak AD - H, *n*-hexane/*i*-PrOH = 4:1, Flow rate = 1.0 mL/min, λ = 210 nm, t_R = 8.7 min (major) and t_R = 46.5 min (minor).

(*R*)-*tert*-butyl (1-benzyl-3-(2-methylallyl)-2-oxoindolin-3-yl)carbamate (3an)

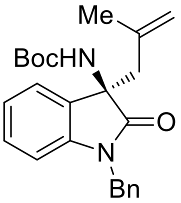

White solid, 65.9mg, 84% yield, 79.4:20.6 *er*; $[\alpha]_D^{28}$ = -6.2 (c = 1.0, $CHCl_3$); MP 116 - 118 °C; ^1H NMR (400 MHz, $CDCl_3$) δ 7.36 (d, J = 7.1 Hz, 2H), 7.31 (t, J = 7.2 Hz, 3H), 7.27 - 7.24 (m, 1H), 7.16 (t, J = 7.4 Hz, 1H), 7.01 (t, J = 7.5 Hz, 1H), 6.66 (d, J = 7.8 Hz, 1H), 5.29 (s, 1H), 5.10 (d, J = 12.0 Hz, 1H), 4.75 (d, J = 37.3 Hz, 3H), 2.62 (s,

2H), 1.41 (s, 3H), 1.26 (s, 9H); ^{13}C NMR (101 MHz, $CDCl_3$) δ 176.74, 153.71, 142.91, 138.38, 135.85, 128.66, 128.58, 127.43, 123.09, 122.39, 117.53, 109.06, 80.33, 61.71, 45.53, 44.14, 28.08, 23.96; HRMS (ESI): *m/z* calcd for $C_{24}H_{28}N_2NaO_3$ $[M+Na]^+$: 415.1998; found: 415.1995; HPLC: Daicel Chiralpak IC, *n*-hexane/*i*-PrOH = 4:1, Flow rate = 1.0 mL/min, λ = 210 nm, t_R = 10.7 min (major) and t_R = 13.3 min (minor).

(R)-tert-butyl (1-benzyl-3-(2-methylenebut-3-en-1-yl)-2-oxoindolin-3-yl) carbamate (3ao)

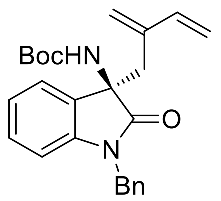

Colorless oil, 55.0mg, 69% yield, 84.4:15.6 *er*; $[\alpha]_D^{23}$ = 14.6 (c = 1.0, $CHCl_3$); ^1H NMR (400 MHz, $CDCl_3$) δ 7.35 (d, J = 6.7 Hz, 2H), 7.31 (t, J = 7.2 Hz, 2H), 7.25 (dd, J = 9.6, 6.9 Hz, 2H), 7.14 (t, J = 7.7 Hz, 1H), 6.96 (t, J = 7.5 Hz, 1H), 6.66 (d, J = 7.8 Hz, 1H), 6.22 (dd, J = 17.6, 10.9 Hz, 1H), 5.38 (br, 1H), 5.20 (d, J = 17.6 Hz, 1H), 5.13 (s, 1H), 5.08 (br, 1H), 4.99 (d, J = 10.9 Hz, 1H), 4.84 (s, 1H), 4.75 (br, 1H), 2.92 (d, J = 13.2 Hz, 1H), 2.63 (d, J = 13.2 Hz, 1H), 1.25 (s, 9H); ^{13}C NMR (101 MHz, $CDCl_3$) δ 176.87, 153.71, 142.63, 138.55, 135.93, 128.63, 128.59, 127.47, 123.78, 122.05, 121.37, 114.82, 108.98, 80.33, 61.74, 44.14, 38.56, 28.06; HRMS (ESI): *m/z* calcd for $C_{25}H_{28}N_2NaO_3$ $[M+Na]^+$: 427.1998; found: 427.1997; HPLC: Daicel Chiralpak IC, *n*-hexane/*i*-PrOH = 4:1, Flow rate = 1.0 mL/min, λ = 210 nm, t_R = 9.2 min (major) and t_R = 11.7 min (minor).

Product Derivatizations.

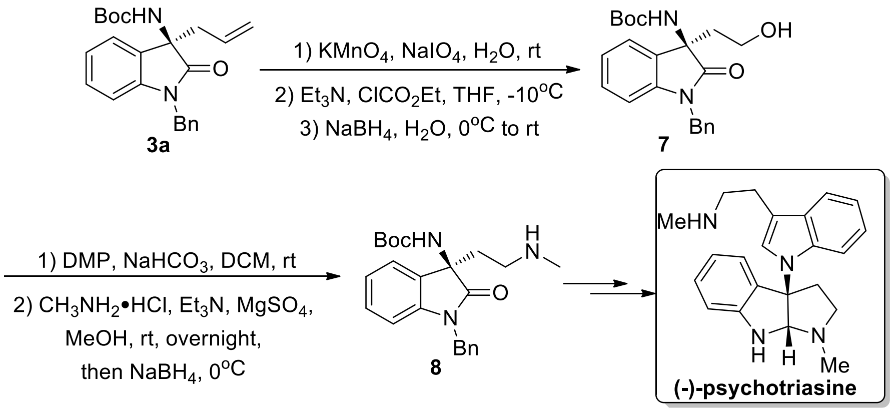

(*R*)-*tert*-butyl (1-benzyl-3-(2-hydroxyethyl)-2-oxoindolin-3-yl)carbamate (7)

(Laschat and Kunz, 1991; Kung et al., 2011)

To a stirred solution of $KMnO_4$ (28.44 mg, 0.18 mmol) and $NaIO_4$ (1.80 g, 8.40 mmol) in water (30 mL) was added compound **3a** (1.20 mmol) at room temperature, and the suspension was stirred until **3a** was completely consumed. The reaction

mixture was extracted five times with ether (300 mL). The combined organic layers were dried with $MgSO_4$, filted and evaporated in vacuo. The residue was directly used for the next step without further purification.

The above residue acid was dissolved in THF (5 mL) and the solution was cooled to -10 ºC. Et_3N (183 µL, 1.32 mmol) and ethyl chloroformate (126 µL, 1.32 mmol) were added dropwise to this solution. After stirring for 60 min, the reaction mixture was filtered off. $NaBH_4$ (95.76 mg, 2.52 mmol) was dissolved in 5 mL H_2O and cooled with an ice bath, then the above filtrate was added slowly to this solution. Returned to room temperature and stirred for 4 h, acidified with 1 M HCl until the pH = 2 - 3. The organic phase was separated and water phase was extracted with EtOAc (20 mL× 3). The organic phases were washed with Sat. $NaHCO_3$ and brine, then dried with $MgSO_4$. Filtered and concentrated in vacuo, and the residue was purified by silica gel column chromatography (ethyl acetate/petroleum ether = 1/5) to afford colorless liquid **7** (279.6 mg, 61% yield, 98.8:1.2 *er*), $[\alpha]_D^{27}$ = +11.6 (c = 0.5, $CHCl_3$); ^{1}H NMR (400 MHz, $CDCl_3$) δ 7.37 - 7.30 (m, 5H), 7.26 (t, *J* = 3.5 Hz, 1H), 7.17 (t, *J* = 7.7 Hz, 1H), 7.04 (t, *J* = 7.5 Hz, 1H), 6.72 (d, *J* = 7.8 Hz, 2H), 5.15 (s, 2H), 4.83 (s, 2H), 3.92 (dddd, *J* = 46.8, 11.4, 7.1, 3.7 Hz, 2H), 2.97 (s, 1H), 2.05 (dddd, *J* = 50.5, 14.8, 7.3, 3.7 Hz, 2H), 1.30 (s, 9H); ^{13}C NMR (101 MHz, $CDCl_3$) δ 177.90, 154.24, 141.88, 135.89, 128.79, 128.54, 127.59, 127.33, 122.79, 122.71, 109.27, 80.23, 61.69, 58.04, 44.06, 39.15, 28.11; HRMS (ESI): *m/z* calcd for $C_{22}H_{27}N_2O_4$ $[M+H]^+$: 383.1971; found: 383.1969; HPLC: Daicel Chiralpak AD - H, *n*-hexane/*i*-PrOH = 4:1, Flow rate = 1.0 mL/min, λ = 210 nm, t_R = 6.5 min (major) and t_R = 10.8 min (minor).

(*R*)-*tert*-butyl (1-benzyl-3-(2-(methylamino)ethyl)-2-oxoindolin-3-yl)carbamate (8) (Kung et al., 2011; Shao et al., 2017)

A mixture of amino alcohol **7** (199.00 mg, 0.52 mmol), $NaHCO_3$ (436.8 mg, 5.20mmol) and Dess-Martin periodinane reagent (331.00 mg, 0.78 mmol) in DCM (5 mL) was stirred at room temperature for 1 h. 2.5 mL $Na_2S_2O_3$ (1.0 M) was added and the resulting mixture was vigorously stirred for 15 min. Saturated $NaHCO_3$ (5 mL) was then added and extracted with DCM (10 mL × 3). The combined organic layers was dried with $MgSO_4$ and concentrated in vacuo to afford crude product.

In a 50 mL round bottom flask under argon atmosphere, the above crude product, methylamine hydrochloride (351.00 mg, 5.20 mmol) and $MgSO_4$ (249.60 mg, 2.08 mmol) were placed. Methanol (10 mL) and Et_3N (721 µL, 5.20 mmol) were added in order at room temperature. After overnight stirring, $NaBH_4$ (59.28 mg, 1.56 mmol) was added at 0°C. After stirring at room temperature for 0.5 h, the reaction mixture was quenched with water, and extracted with EtOAc (10 mL × 3). The combined organic layers were dried over Na_2SO_4 and filtered. Concentrated in vacuo, and the residue was purified by silica gel column chromatography (ethyl acetate to DCM/MeOH = 15/1) to afford compound **8** as yellow liquid (174.6 mg, 85% yield, 99.5:0.5 *er*); $[\alpha]_D^{27}$ = +18.4 (c = 0.5, $CHCl_3$); ^{1}H NMR (400 MHz, $CDCl_3$) δ 7.39 - 7.37 (m, 2H), 7.33 (t, *J* = 7.5 Hz, 2H), 7.30 - 7.23 (m, 3H), 7.18 (t, *J* = 14..8 Hz, 1H), 7.02 (t, *J* = 7.5 Hz, 1H), 6.73 (d, *J* = 7.8 Hz, 1H), 5.10 (br, 1H), 4.79 (br, 1H), 3.02 (s,

2H), 2.61 (s, 4H), 2.38 (ddt, J = 14.2, 10.0, 6.2 Hz, 1H), 1.23 (s, 9H); ^{13}C NMR (101 MHz, $CDCl_3$) δ 176.60, 154.35, 141.87, 135.72, 130.28, 128.94, 128.89, 127.72, 127.52, 123.27, 122.73, 109.26, 80.31, 60.35, 53.48, 44.55, 44.17, 33.46, 33.37, 28.07; HRMS (ESI): *m/z* calcd for $C_{23}H_{30}N_3O_3$ $[M+H]^+$: 396.2287; found: 396.2286; HPLC: Daicel Chiralpak IF, *n*-hexane/*i*-PrOH = 3:2, Flow rate = 1.0 mL/min, λ = 210 nm, t_R = 15.4 min (major) and t_R = 30.4 min (minor).

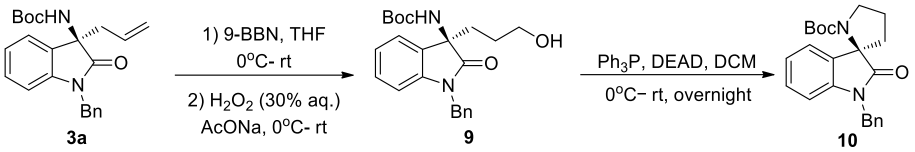

(*R*)-*tert*-butyl (1-benzyl-3-(3-hydroxypropyl)-2-oxoindolin-3-yl)carbamate (9)

(Shibasaki et al., 2003)

To a stirred solution of compound **3a** (869.40 mg, 2.3 mmol) in dry THF (5.0 mL) was added 9-BBN (0.5 M in THF, 11.50 mL,5.7 mmol) at 0 ˚C. The mixture was warmed to room temperature and stirred for 24 h. H_2O_2 (30%, 12.70 mL) and NaOAc (20%, 16.10 mL) were added in order at 0 ˚C, and the resulting mixture was stirred for 5 h at room temperature. The aqueous layer was extracted with EtOAc (20 mL× 3), and the combined organic layers were dried over anhydrous Na_2SO_4. After filtered and evaporation, the crude mixture was purified by silica gel column chromatography (ethyl acetate/petroleum ether = 1/1) to give compound **9** as white solid (867.4 mg, 95% yield, 98.3:1.7 *er*); $[\alpha]_D^{27}$ = +22.0 (c = 1.0, $CHCl_3$); MP 72 - 73 °C; ^{1}H NMR (400 MHz, $CDCl_3$) δ 7.37 (d, J = 7.2 Hz, 2H), 7.31 (t, J = 7.3 Hz, 2H), 7.28 - 7.23 (m, 2H), 7.18 (t, J = 7.8 Hz, 1H), 7.02 (t, J = 7.5 Hz, 1H), 6.72 (d, J = 7.8 Hz, 1H), 5.48 (s, 1H), 5.08 (br, 1H), 4.80 (br, 1H), 3.56 (t, J = 6.2 Hz, 2H), 2.09- 1.90 (m, 2H), 1.56- 1.48 (m, 2H), 1.26 (s, 9H); ^{13}C NMR (101 MHz, $CDCl_3$) δ 177.76, 154.23, 142.39, 135.96, 128.76, 128.45, 127.57, 127.48, 122.69, 122.63, 109.08, 80.20, 61.76, 61.58, 44.12, 34.64, 28.08, 25.70; HRMS (ESI): *m/z* calcd for $C_{23}H_{29}N_2O_4$ $[M+H]^+$: 397.2127; found: 397.2122; HPLC: Daicel Chiralpak AD - H, *n*-hexane/*i*-PrOH = 4:1, Flow rate = 1.0 mL/min, λ = 210 nm, t_R = 8.4 min (major) and t_R = 13.6 min (minor).

(*R*)-*tert*-butyl 1-benzyl-2-oxospiro[indoline-3,2'-pyrrolidine]-1'-carboxylate (10)

(Lam et al., 2013)

To a stirred solution of compound **9** (79.20 mg, 0.20 mmol) and Ph_3P (68.10 mg, 0.26 mmol) in DCM (2 mL) at 0 °C was added a solution of DEAD (38.00 μL, 0.24 mmol) in DCM (2 mL). The resulting mixture was warmed to room temperature slowly, and then stirred overnight. The reaction was quenched with EtOH (1 mL) and concentrated under reduced pressure. The residue was purified by silica gel flash column chromatography (ethyl acetate/petroleum ether = 1/2) to afford compound **10** as white solid (54.1 mg, 72% yield, 98.7:1.3 *er*); $[\alpha]_D^{27}$ = -11.1 (c = 0.2, $CHCl_3$); MP 106 - 107 °C; ^{1}H NMR (400 MHz, $CDCl_3$) δ 7.39 - 7.27 (m, 5H), 7.20 - 7.15 (m, 2H), 7.01 (t, J = 7.0 Hz, 1H), 6.72 (d, J = 8.5 Hz, 1H), 5.28 (d, J = 15.5 Hz, 1H), 4.45 (d, J

= 15.5 Hz, 1H), 3.92 - 3.76 (m, 2H), 2.49 - 2.38 (m, 1H), 2.36 - 2.25 (m, 1H), 2.20 - 2.08 (m, 2H), 0.99 (s, 9H); ^{13}C NMR (101 MHz, $CDCl_3$) δ 177.73, 152.96, 142.22, 136.03, 132.86, 128.80, 128.38, 127.69, 127.53, 122.68, 121.86, 108.70, 80.02, 66.84, 48.10, 43.96, 39.94, 27.78, 23.04; HRMS (ESI): *m/z* calcd for $C_{23}H_{27}N_2O_3$ $[M+H]^+$: 379.2022; found: 379.2018; HPLC: Daicel Chiralpak AD - H, *n*-hexane/*i*-PrOH = 19:1, Flow rate = 1.0 mL/min, λ = 210 nm, t_R = 23.8min (major) and t_R = 27.6 min (minor).

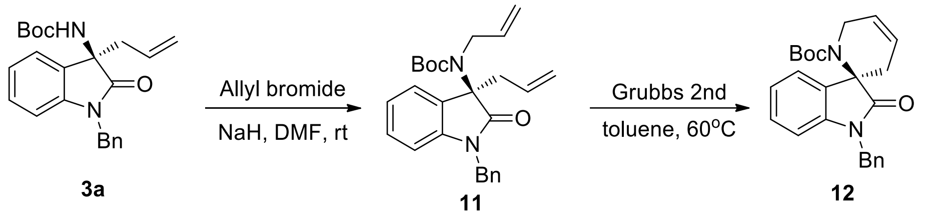

3a 11 12

(*R*)-*tert*-butyl allyl(3-allyl-1-benzyl-2-oxoindolin-3-yl)carbamate (11) (Nakamura et al., 2013)

To a stirred solution of compound **3a** (124.70 mg, 0.33 mmol) in DMF (2.0 mL) was added NaH (60% in oil, 15.80 mg, 0.39 mmol) at 0˚C. The resulting mixture was warmed to room temperature, after stirring for 30 min, allylbromide (31.50 µL, 0.36 mmol) was added. The resulting mixture continued to stir for 30min untill disappearance of **3a** monitored by TLC. The crude mixture was directly purified by silica gel column chromatography (ethyl acetate/petroleum ether = 1/5) to give compound **11** as white solid (130.7 mg, 98% yield, 98.5:1.5 *er*); $[\alpha]_D^{27}$ = -56.8 (c = 1.0, $CHCl_3$); MP 51 - 52 °C; ^{1}H NMR (400 MHz, $CDCl_3$) δ 7.40 (d, *J* = 7.4 Hz, 2H), 7.31 - 7.23 (m, 3H), 7.20 - 7.12 (m, 2H), 6.99 (t, *J* = 7.5 Hz, 1H), 6.69 (d, *J* = 7.7 Hz, 1H), 6.12 - 6.02 (m, 1H), 5.41 (d, *J* = 17.3 Hz, 1H), 5.26 (d, *J* = 10.3 Hz, 1H), 5.20 - 5.05 (m, 2H), 4.94 (d, *J* = 16.9 Hz, 1H), 4.78 (d, *J* = 10.0 Hz, 1H), 4.54 (d, *J* = 15.6 Hz, 1H), 4.36 (d, *J* = 18.8 Hz, 1H), 4.18 (dd, *J* = 17.1, 6.7 Hz, 1H), 2.88 - 2.77 (m, 2H), 1.16 (s, 9H); ^{13}C NMR (101 MHz, $CDCl_3$) δ 176.81, 154.30, 142.82, 136.74, 136.12, 131.75, 130.30, 128.48, 128.16, 128.07, 127.48, 122.36, 122.21, 120.27, 116.28, 108.53, 80.83, 66.06, 46.64, 44.34, 40.93, 28.04; HRMS (ESI): *m/z* calcd for $C_{26}H_{31}N_2O_3$ $[M+H]^+$: 419.2335; found: 419.2329; HPLC: Daicel Chiralpak IA, *n*-hexane/*i*-PrOH = 4:1, Flow rate = 1.0 mL/min, λ = 210 nm, t_R = 5.4 min (major) and t_R = 7.9 min (minor).

(*R*)-*tert*-butyl 1-benzyl-2-oxo-3',6'-dihydro-1'H-spiro[indoline-3,2'-pyridine]-1'-carboxylate (12) (Nakamura et al., 2013)

A mixture of compound **11** (121.20 mg, 0.30 mmol) and Grubbs 2nd (25.47 mg, 0.03 mmol) in toluene (2.0 mL) was stirred for 20 min at 60 ˚C. After cooling to room temperature, the crude mixture was directly purified by silica gel column chromatography (ethyl acetate/petroleum ether = 1/6) to give compound **12** as white solid (92.5 mg, 79% yield, 98.5:1.5 *er*); $[\alpha]_D^{27}$ = +70.2 (c = 1.0, $CHCl_3$); MP 89 - 90

°C; ^{1}H NMR (400 MHz, $CDCl_3$) δ 7.35 - 7.31 (m, 4H), 7.27 - 7.23 (m, 1H), 7.18 - 7.11 (m, 2H), 6.92 (t, *J* = 7.5 Hz, 1H), 6.68 (d, *J* = 7.7 Hz, 1H), 6.22 - 6.18 (m, 1H), 6.02 - 5.95 (m, 1H), 5.34 (d, *J* = 15.6 Hz, 1H), 4.52 (s, 1H), 4.28 (d, *J* = 15.7 Hz, 1H), 4.15 (d, *J* = 17.6 Hz, 1H), 2.81 (dp, *J* = 15.6, 2.8 Hz, 1H), 2.15 (dd, *J* = 15.8, 6.6 Hz, 1H), 1.21 (s, 9H); ^{13}C NMR (101 MHz, $CDCl_3$) δ 177.21, 154.17, 141.61, 136.14, 133.46, 128.74, 128.21, 127.53, 127.28, 123.12, 122.26, 108.80, 80.86, 61.18, 43.92, 43.29, 34.99, 28.06; HRMS (ESI): *m/z* calcd for $C_{24}H_{27}N_2O_3$ $[M+H]^+$: 391.2022; found: 391.2016; HPLC: Daicel Chiralpak AD - H, *n*-hexane/*i*-PrOH = 19:1, Flow rate = 1.0 mL/min, λ = 210 nm, t_R = 20.7 min (major) and t_R = 30.5 min (minor).

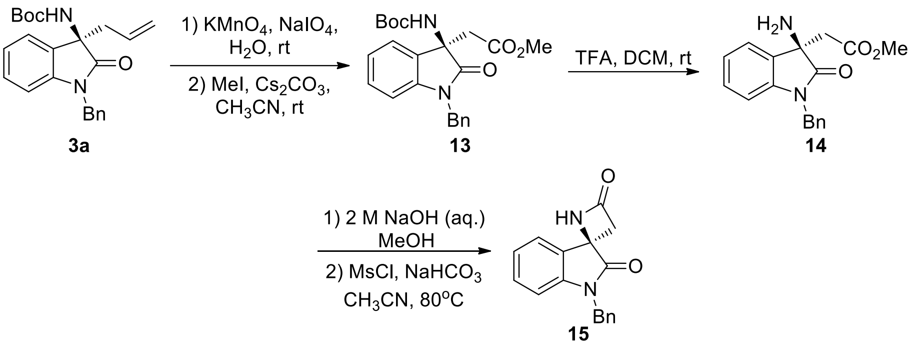

(*R*)-methyl 2-(1-benzyl-3-((tert-butoxycarbonyl)amino)-2-oxoindolin-3-yl)acetate (13) (Laschat and Kunz, 1991; Shao et al., 2017)

To a stirred solution of $KMnO_4$ (28.444 mg, 0.18 mmol) and $NaIO_4$ (1.80g, 8.40 mmol) in water (30 mL) was added compound **3a** (1.2 mmol) at room temperature, and the suspension was stirred until **3a** was completely consumed. The reaction mixture was extracted five times with ether (100 mL). The combined organic layers were dried with $MgSO_4$, filted and evaporated in vacuo. The residue was directly used for the next step without further purification.

To a stirred solution of the above residue in CH_3CN (30 mL) were added Cs_2CO_3 (782.4 mg, 2.4 mmol) and MeI (150 µL, 2.4 mmol) at room temperature. The reaction mixture was stirred for 8 h, and water was added. The reaction mixture was extracted with EtOAc (30 mL× 3). The combined organic layers were washed with brine, dried with Na_2SO_4, filtered and concentrated in vacuo. The residue was purified by silica gel column chromatography (ethyl acetate/petroleum ether = 1/5, v/v) to afford white solid **13** (340.6mg, 68% yield, 98.7:1.3 *er*); $[\alpha]_D^{27}$ = +46.6 (c = 1.0, $CHCl_3$); MP 53-54 °C; ^{1}H NMR (400 MHz, $CDCl_3$) δ 7.37 (d, *J* = 7.1 Hz, 2H), 7.32 (t, *J* = 7.3 Hz, 2H), 7.30 - 7.23 (m, 2H), 7.18 (t, *J* = 7.7 Hz, 1H), 7.00 (t, *J* = 7.5 Hz, 1H), 6.71 (d, *J* = 7.8 Hz, 1H), 6.35 (s, 1H), 5.08 (br, 1H), 4.82 (br, 1H), 3.69 (s, 3H), 2.96 (d, *J* = 15.0 Hz, 1H), 2.59 (d, *J* = 15.0 Hz, 1H), 1.29 (s, 9H); ^{13}C NMR (101 MHz, $CDCl_3$) δ 175.55, 170.19, 153.79, 142.29, 135.75, 129.56, 129.14, 128.76, 127.59, 127.37, 122.96, 122.80, 109.41, 80.40, 59.25, 52.20, 44.21, 40.90, 28.12; HRMS (ESI): *m/z* calcd for $C_{23}H_{27}N_2O_5$ $[M+H]^+$: 411.1920; found: 411.1912; HPLC: Daicel Chiralpak AD - H, *n*-hexane/*i*-PrOH = 3:2, Flow rate = 1.0 mL/min, λ = 210 nm, t_R = 8.0 min (major) and t_R = 23.6 min (minor).

(*R*)-methyl 2-(3-amino-1-benzyl-2-oxoindolin-3-yl)acetate (14) (Melchiorre et al., 2008)

To a stirred solution of compound **13** (271.50 mg, 0.66 mmol) in DCM (5 mL) was added TFA (983.00 μL, 13.20 mmol) at 0°C. After stirring for 2 h at room temperature, the mixture was cooled to 0°C and sat. $NaHCO_3$ (20 mL) was added. The aqueous layer was extracted with DCM (10 mL × 3) and the combined organic layers were dried over $MgSO_4$. Filtered and concentrated in vacuo, the residue was purified by silica gel column chromatography (ethyl acetate/petroleum ether = 1/1) to give compound **14** as white solid (191.9 mg, 94% yield, 99.0:1.0 *er*); $[\alpha]_D^{27}$ = +57.4 (c = 1.0, $CHCl_3$); MP 52 - 53 °C; ^1H NMR (400 MHz, $CDCl_3$) δ 7.39 (d, *J* = 7.4 Hz, 1H), 7.38 - 7.30 (m, 4H), 7.29 - 7.26 (m, 1H), 7.20 (td, *J* = 7.7, 1.3 Hz, 1H), 7.04 (t, *J* = 7.6 Hz, 1H), 6.74 (d, *J* = 7.8 Hz, 1H), 5.05- 4.81 (dd, *J* = 42.3, 15.6 Hz, 2H), 3.52 (s, 3H), 3.00 (s, 2H), 1.92 (s, 2H); ^{13}C NMR (101 MHz, $CDCl_3$) δ 179.18, 169.95, 142.72, 135.74, 130.81, 129.36, 128.80, 127.67, 127.41, 123.71, 122.93, 109.47, 58.47, 51.73, 44.01, 42.45; HRMS (ESI): *m/z* calcd for $C_{18}H_{19}N_2O_3$ $[M+H]^+$: 311.1396 found: 311.1393; HPLC: Daicel Chiralpak AD - H, *n*-hexane/*i*-PrOH = 9:1, Flow rate = 1.0 mL/min, λ = 210 nm, t_R = 28.0 min (major) and t_R = 30.2 min (minor).

(*R*)-1'-benzylspiro[azetidine-2,3'-indoline]-2',4-dione (15) (Shibasaki et al., 2010)

A mixture of compound **14** (91.50 mg, 0.295 mmol), 2 M *aq.* NaOH (0.45 mL) and MeOH (0.90 mL) was stirred for 2 h at room temperature. Acidified the reaction mixture by 1 M *aq.* HCl, and then the reaction mixture was evaporated at 50 °C to give a crude carboxylic acid. To a round bottom flask with the crude carboxylic acid were added $NaHCO_3$ (123.98 mg, 1.48 mmol), the MsCl (68.50 μL, 0.89 mmol) and CH_3CN (3 mL) were added in order under argon atmosphere. The reaction mixture was stirred for 18 h at 80 °C. After cooling to room temperature, the mixture was filtered and washed with 2.5% MeOH in EtOAc. The filtrate was concentrated under reduced pressure and the residue was purified by silica gel flash column chromatography (ethyl acetate/petroleum ether = 1/1) to afford compound **15** as white solid (54.9 mg, 67% yield, 98.8:1.2 *er*); $[\alpha]_D^{27}$ = +82.4 (c = 0.5, $CHCl_3$); MP 116 - 117 °C; ^1H NMR (400 MHz, $CDCl_3$) δ 7.46 (d, *J* = 7.3 Hz, 1H), 7.37 - 7.23 (m, 4H), 7.11 (t, *J* = 7.5 Hz, 1H), 6.79 (d, *J* = 7.9 Hz, 1H), 6.22 (s, 1H), 4.92 (dd, *J* = 33.4, 15.5 Hz, 2H), 3.41 (dd, *J* = 105.7, 14.5 Hz, 2H); ^{13}C NMR (101 MHz, $CDCl_3$) δ 175.38, 166.49, 142.70, 135.22, 130.32, 128.93, 127.94, 127.44, 126.76, 123.49, 109.76, 55.99, 51.11, 44.30; HRMS (ESI): *m/z* calcd for $C_{17}H_{15}N_2O_2$ $[M+H]^+$: 279.1134; found: 279.1131; HPLC: Daicel Chiralpak AD - H, *n*-hexane/*i*-PrOH = 4:1, Flow rate = 1.0 mL/min, λ = 210 nm, t_R = 11.1 min (major) and t_R = 13.1 min (minor).

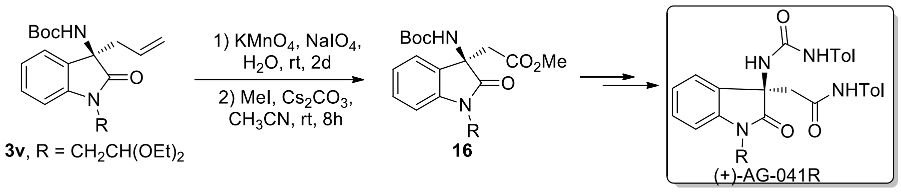

(*R*)-methyl 2-(3-((*tert*-butoxycarbonyl)amino)-1-(2,2-diethoxyethyl)-2-oxoindolin-3- yl) acetate (16) (Laschat and Kunz, 1991; Shao et al., 2017)

According to the approach to compound **13**, compound **16** could be afforded as colorless liquid (54.1 mg, 62% yield, 99.6:0.4 *er*); $[\alpha]_D^{27}$ = +51.3 (c = 0.3, $CHCl_3$); ^1H NMR (400 MHz, $CDCl_3$) δ 7.28 - 7.22 (m, 2H), 7.05 (d, *J* = 7.9 Hz, 1H), 7.00 (t, *J* = 7.5 Hz, 1H), 6.31 (s, 1H), 4.74 (t, *J* = 5.4 Hz, 1H), 4.01 (s, 1H), 3.77- 3.72 (m, 2H), 3.69 (s, 3H), 3.60 - 3.50 (m, 2H), 2.70 (dd, *J* = 147.9, 15.1 Hz, 2H), 1.25 (s, 9H), 1.20 - 1.04 (m, 6H); ^{13}C NMR (101 MHz, $CDCl_3$) δ 175.66, 170.32, 153.70, 142.85, 129.33, 128.90, 122.62, 122.52, 109.92, 100.46, 80.23, 63.53, 63.23, 58.98, 52.15, 43.73, 40.71, 28.06, 15.28, 15.23; HRMS (ESI): *m/z* calcd for $C_{22}H_{32}N_2NaO_7$ $[M+Na]^+$: 459.2107; found: 459.2099; HPLC: Daicel Chiralpak AD - H, *n*-hexane/*i*-PrOH = 4:1, Flow rate = 1.0 mL/min, λ = 210 nm, t_R = 8.1 min (major) and t_R = 17.4 min (minor).

References.

Andrae, D., Häußermann, U., Dolg, M., Stoll, H. and Preuß, H. (1990). Energy-adjustedab initio pseudopotentials for the second and third row transition elements. Theoretica. Chimica. Acta. *77*, 123-141.

Aslam, N.A., Babu, S.A., Rani, S., Mahajan, S., Solanki, J., Yasuda, M. and Baba, A. (2015). Diastereoselective construction of 3-aminooxindoles with adjacent stereocenters: stereocontrolled addition of γ-substituted allylindiums to isatin ketimines. Eur. J. Org. Chem. 4168-4189.

Bittner, S., Assaf, Y., Krief, P., Pomerantz, M., Ziemnicka, B. T. and Smith, C.G. (1985). Synthesis of *N*-acyl-, *N*-sulfonyl-, and *N*-phosphinylphospha-λ^5-azenes by a redox-condensation reaction using amides, triphenylphosphine, and diethyl azodicarboxylate. J. Org. Chem. *50*, 1712-1718.

Chung, L.W., Sameera, W.M.C., Ramozzi, R., Page, A.J., Hatanaka, M., Petrova, G.P., Harris, T.V., Li, X., Ke, Z., Liu, F., Li, H.-B., Ding, L. and Morokuma, K. (2015). The ONIOM Method and Its Applications. Chem. Rev. *115*, 5678-5796.

Dai, J., Xiong, D., Yuan, T., Liu, J., Chen, T. and Shao, Z. (2017). Chiral primary amine catalysis for asymmetric Mannich reactions of aldehydes with ketimines: stereoselectivity and reactivity. Angew. Chem. Int. Ed. *56*, 12697-12701.

Frisch, M.J., et al. (2009). Gaussian 09, revision B.01; Gaussian, Inc.: Wallingford, CT.

Gianelli, C., Sambri, L., Carlone, A., Bartoli, G. and Melchiorre, P. (2008). Aminocatalytic enantioselective *anti*-Mannich reaction of aldehydes with in situ generated *N*-Cbz and *N*-Boc imines. Angew. Chem., Int. Ed. *47*, 8700-8702.

Hay, P.J. and Wadt, W.R. (1985). Ab initio effective core potentials for molecular calculations. Potentials for K to Au including the outermost core orbitals. J. Chem. Phys. *82*, 299-310.

Hepburn, H.B., Chotsaeng, N., Luo, Y. and Lam, H.W. (2013). Enantioselective rhodium-catalyzed allylation of cyclic imines with potassium allyltrifluoroborates. Synthesis *45*, 2649-2661.

Hu, F.-L., Wei, Y., Shi, M., Pindic, S. and Li, G. (2013). Asymmetric catalytic aza-Morita–Baylis–Hillman reaction for the synthesis of 3-substituted-3- aminooxindoles with chiral quaternary carbon centers. Org. Biomol. Chem. *11*, 1921-1924.

Laschat, S. and Kunz, H. (1991). Carbohydrates as chiral templates: diastereoselective synthesis of *N*-Glycosyl-*N*-homoallylamines and β-amino acids from imines. J. Org. Chem. *56*, 5883-5889.

Mouri, S., Chen, Z., Mitsunuma, H., Furutachi, M., Matsunaga, S. and Shibasaki, M. (2010). Catalytic asymmetric synthesis of 3-aminooxindoles: enantiofacial selectivity switch in bimetallic vs monometallic schiff base catalysis. J. Am. Chem. Soc. *132*, 1255-1257.

Nakamura, S. and Takahashi, S. (2015). Organocatalytic enantioselective peroxidation of ketimines derived from isatins. Org. Lett. *17*, 2590-2593.

Nakamura, S., Hyodo, K., Nakamura, M., Nakane, D. and Masuda, H. (2013). Catalytic enantioselective allylation of ketimines by using palladium pincer complexes with chiral bis(imidazoline)s. Chem. - Eur. J. *19*, 7304-7309.

Niyomchon, S., Audisio, D., Luparia, M. and Maulide, N. (2013). Regio- and enantioselective cyclobutene allylations. Org. Lett. *15*, 2318-2321.

Ohshima, T., Gnanadesikan, V., Shibuguchi, T., Fukuta, Y., Nemoto, T. and Shibasaki, M. (2003). Enantioselective syntheses of aeruginosin 298-A and its analogues using a catalytic asymmetric phase-transfer reaction and epoxidation. J. Am. Chem. Soc. *125*, 11206-11207.

Qu, W., Zha, Z., Ploessl, K., Lieberman, B.P., Zhu, L., Wise, D.R., Thompson, C.B. and Kung, H.F. (2011). Synthesis of optically pure 4-fluoro-glutamines as potential metabolic imaging agents for tumors. J. Am. Chem. Soc. 133, 1122-1133.

Rappe, A.K., Casewit, C.J., Colwell, K.S., Goddard III, W.A. and Skiff, W.M. (1992). UFF, a full periodic table force field for molecular mechanics and molecular dynamics simulations. J. Am. Chem. Soc. *114,* 10024-10035.

Simón, L. and Goodman J.M. (2010). DFT Study on the Factors Determining the Enantioselectivity of Friedel−Crafts Reactions of Indole with N-Acyl and N-Tosylimines Catalyzed by BINOL−Phosphoric Acid Derivatives. J. Org. Chem. *75*, 589-597.

Simón, L. and Goodman, J.M. (2008). Theoretical Study of the Mechanism of Hantzsch Ester Hydrogenation of Imines Catalyzed by Chiral BINOL-Phosphoric Acids. J. Am. Chem. Soc. *130*, 8741-8747.

Simón, L. and Goodman, J.M. (2011). A Model for the Enantioselectivity of Imine Reactions Catalyzed by BINOL−Phosphoric Acid Catalysts. J. Org. Chem. *76*, 1775-1788.

Simón, L. and Goodman, J.M. (2012). Mechanism of Amination of β-Keto Esters by Azadicarboxylates Catalyzed by an Axially Chiral Guanidine: Acyclic Keto Esters React through an E Enolate. J. Am. Chem. Soc. *134*, 16869-16876.

Simón, L. and Paton, R.S. (2015). Origins of Asymmetric Phosphazene Organocatalysis: Computations Reveal a Common Mechanism for Nitro- and Phospho-Aldol Additions. J. Org. Chem. *80*, 2756-2766.

Simón, L. and Paton, R.S. (2016). QM/MM study on the enantioselectivity of spiroacetalization catalysed by an imidodiphosphoric acid catalyst: how confinement works. Org. Biomol. Chem. *14*, 3031-3039.

Simón, L. and Paton, R.S. (2017). Phosphazene Catalyzed Addition to Electron-Deficient Alkynes: The Importance of Nonlinear Allenyl Intermediates upon Stereoselectivity. J. Org. Chem. *82*, 3855-3863.

Simón, L. and Paton, R.S. (2018). The True Catalyst Revealed: The Intervention of Chiral Ca and Mg Phosphates in Brønsted Acid Promoted Asymmetric Mannich Reactions. J. Am. Chem. Soc. *140*, 5412-5420.

Svensson, M., Humbel, S. and Morokuma, K. (1996). Energetics using the single point IMOMO (integrated molecular orbital+molecular orbital) calculations: Choices of computational levels and model system. J. Chem. Phys. *105*, 3654-3661.

The PyMOL Molecular Graphics System, Version 1.3, Schrödinger, LLC, (2009–2010.).

Vreven, T. and Morokuma, K. (2000). On the application of the IMOMO (integrated molecular orbital + molecular orbital) method. J. Comput. Chem. *21*, 1419-1432.

Yan, W.J., Wang, D., Feng, J.C., Li, P., Zhao, D.P. and Wang, R. (2012). Synthesis of *N*-alkoxycarbonyl ketimines derived from isatins and their application in enantioselective synthesis of 3-aminooxindoles. Org. Lett. *14*, 2512-2515.

Yua, J.-S. and Zhou, J. (2015). A highly efficient Mukaiyama–Mannich reaction of *N*-Boc isatin ketimines and other active cyclic ketimines using difluoroenol silyl ethers catalyzed by $Ph_3PAuOTf$. Org. Biomol. Chem. *13*, 10968-10972.

Zhao, P., Li, Y., Gao, G., Wang, S., Yan, Y., Zhan, X., Liu, Z., Mao, Z., Chen, S. and Wang, L. (2014). Design, synthesis and biological evaluation of *N*-alkyl or aryl substituted isoindigo derivatives as potential dual cyclin-dependent kinase 2 (CDK2)/glycogen synthase kinase 3β (GSK-β) phosphorylation inhibitors. Eur. J. Med. Chem. *86*, 165-174.

Zhao, Y. and Truhlar, D.G. (2008). Density Functionals with Broad Applicability in Chemistry. Acc. Chem. Res. *41*, 157-167.
